# A General Entry to *Ganoderma* Meroterpenoids: Synthesis of Applanatumol E, H, and I, Lingzhilactone B, Meroapplanin B, and Lingzhiol

**DOI:** 10.1021/acs.orglett.4c03192

**Published:** 2024-10-11

**Authors:** Alexander Rode, Nicolas Müller, Ondřej Kováč, Klaus Wurst, Thomas Magauer

**Affiliations:** † Department of Organic Chemistry and Center for Molecular Biosciences, University of Innsbruck, 6020 Innsbruck, Austria; ‡ Department of Organic Chemistry, Palacký University Olomouc, 77900 Olomouc, Czech Republic; § Department of General Inorganic and Theoretical Chemistry, University of Innsbruck, 6020 Innsbruck, Austria

## Abstract

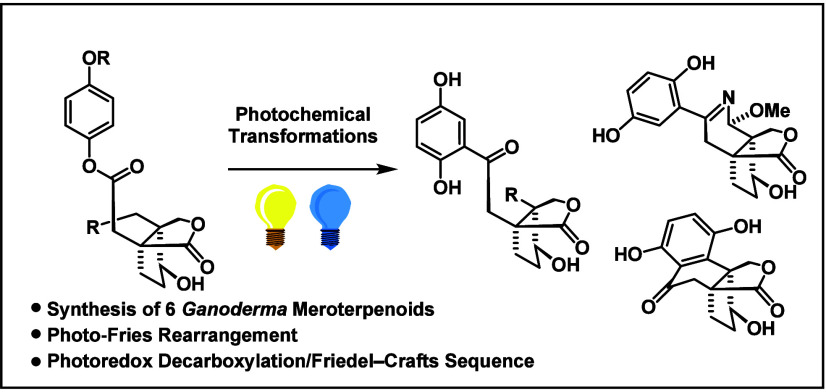

*Ganoderma* meroterpenoids are fungal derived hybrid natural product class containing a 1,2,4-trisubstituted benzene ring and a polycyclic terpenoid part. The representatives applanatumol E, H and I, lingzhilactone B, and meroapplanin B share the same bicyclic lactone moiety connected to the arene. Employing photo-Fries rearrangements as the key step enabled a general entry to these natural products. For the synthesis of the tetracyclic framework of lingzhiol, we made use of a powerful photoredox oxidative decarboxylation/Friedel–Crafts sequence.

*Ganoderma* is genus of wood decay fungi that has been used in traditional Chinese medicine to treat a variety of medical conditions such as hypertension, chronic bronchitis and diabetes.^1−3^ The secondary metabolites from these fungi cover the classes of polysaccharides, (mero)terpenoids, steroids and fatty acids of which polysaccharides were found to be the main bioactive component. To date, more than 100 meroterpenoids belonging to this natural product class have been isolated including applanatumol A (**1**),^4^ applanatumol E (**2**),^5^ lingzhilactone B (**3**),^6^ meroapplanin B (**4**),^7^ lingzhiol (**5**),^8^ and ganoapplanin^9^ (**6**, Figure 1).

**Figure 1:**
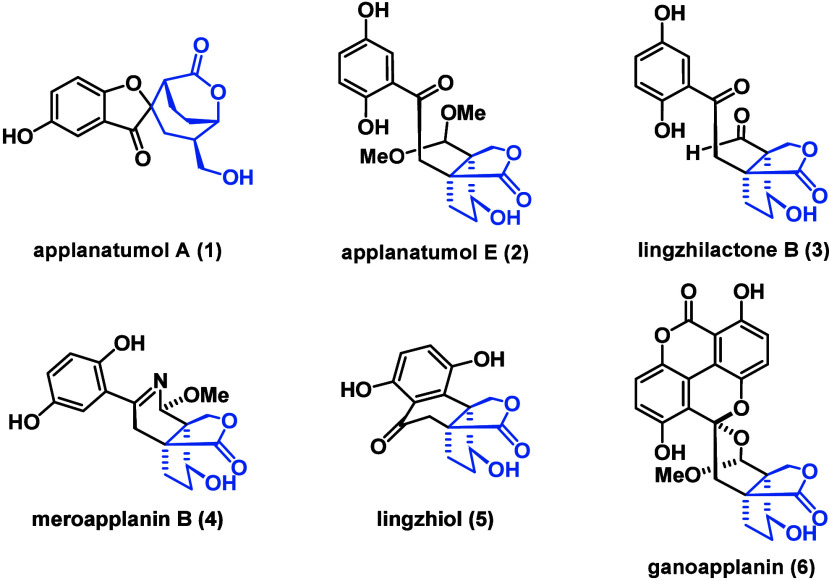
Selected polycyclic *Ganoderma* meroterpenoids

These natural products have a common bicyclic lactone moiety (highlighted in blue), connected to a 1,2,4-trisubstituted benzene ring. In the case of lingzhiol (**5**), the aromatic core is further linked to the bicycle to give a unique tetralone subunit. Bioactivity assays revealed lingzhilactone B (**3**) and lingzhiol (**5**) to protect against renal injuries by increasing the activity of antioxidants and hence might be beneficial for antikidney disease drug design.^6,8^ Ganoapplanin (**6**) was reported to be an inhibitor for T-type voltage-gated calcium channels (IC_50_ = 36.6 μM), positioning it as a potential lead compound for the development of treatments for neurodegenerative diseases.^10,11^

Owing to their structural complexity and medicinal relevance, meroterpenoids from the *Ganoderma* genus constitute an attractive target for total synthesis. While syntheses for lingzhilactone B (**3**),^12^ lingzhiol (**5**)^13−18^ and ganoapplanin (**6**)^19^ were previously reported, synthetic strategies to access the applanatumol natural product family and meroapplanin B (**4**) are still unknown.

Here, we report a general entry to this natural product class involving two photochemical reactions as the key steps.^20^ We began our endeavor with the retrosynthetic analysis of applanatumol E (**2**) for which a photo-Fries retron was found (Scheme 1). Disconnecting the ketone from the arene revealed ester **7** as the required precursor. Further dissection gave commercially available hydroquinone (not shown) and the corresponding acid **8**. The functional group pattern of **8** was derived from bicyclic lactone **9** via Krapcho decarboxylation of the methyl ester followed by allylation and sequential oxidation. For the installation of the crucial bicyclic lactone component **9**, we identified a highly diastereoselective iodocarbocyclization employing malonate **10**.

**Scheme 1:**
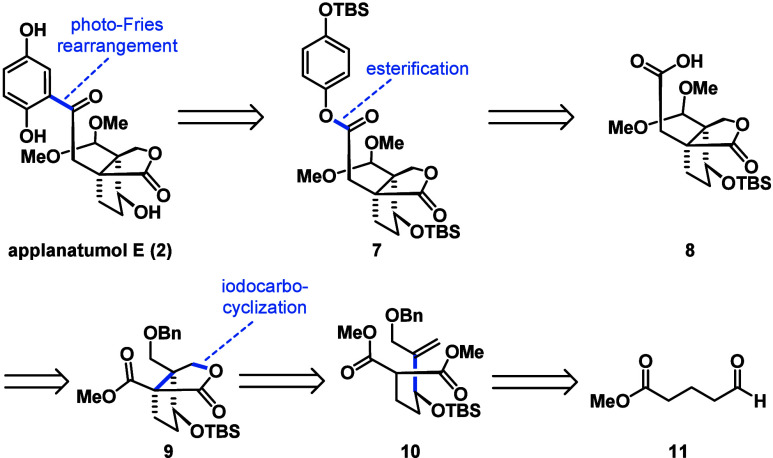
Retrosynthetic Analysis

As depicted in Scheme 2A, our synthesis commenced with the lithiation and 1,2-addition of vinyl iodide **12**(21,22) to commercially available aldehyde **11** to give the corresponding allylic alcohol. Silylation (TBSCl, imidazole, DMAP) provided ester **13** in 53% over two steps. Sequential treatment of ester **13** with LDA and methyl chloroformate at −78 °C gave the prerequisite malonate **10** in 56% yield.^23^ Alternatively, **10** can also be accessed via a one-pot Nozaki–Hiyama–Kishi (NHK) reaction between aldehyde **14** and vinyl iodide **12** to form the corresponding secondary alcohol, which was protected in-situ to give TBS ether **10**.^19^

**Scheme 2:**
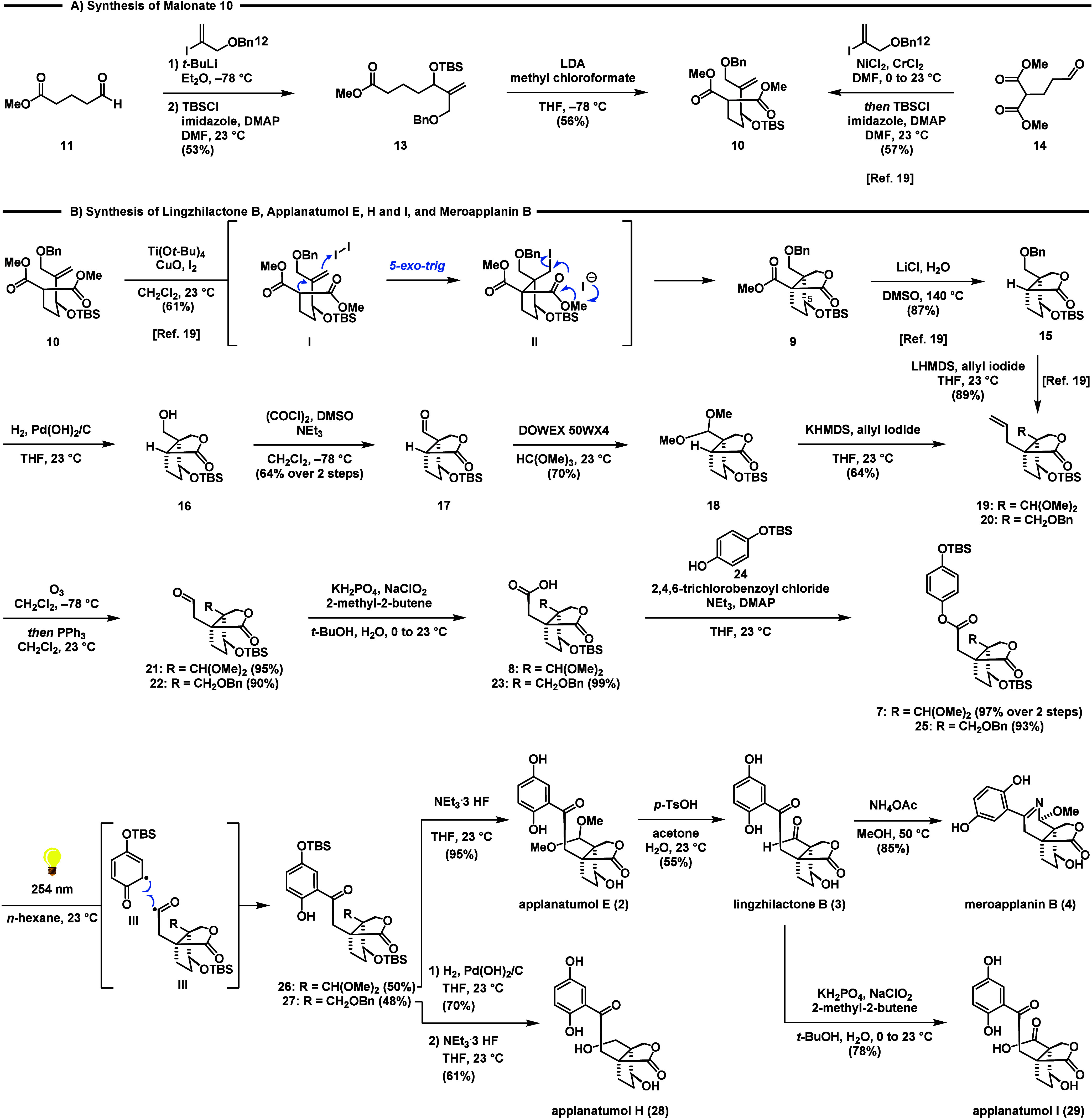
Synthesis of Lingzhilactone B (**3**), Applanatumol E (**2**), H (**28**), and I (**29**), and Meroapplanin B (**4**)

For the following iodocarbocyclization reaction we relied on the conditions reported in seminal work of Taguchi and recently employed by us for the synthesis of ganoapplanin (**6**) (Scheme 2B).^19,24,25^ Following the established protocol, **10** was converted via a 5-*exo*-trig cyclization/lactonization sequence to the bicyclic lactone **9** as a single diastereomer on decagram scale in 61% yield. The two quaternary centers are set in the 5-*exo*-trig cyclization of I to II with the desired relative configuration at C5.^26^ A Krapcho decarboxylation^27^ using LiCl in DMSO/water at elevated temperature (140 °C) allowed for clean removal of the ester group of **9** to furnish **15** in 87% yield. The use of NaCl instead of LiCl under otherwise similar reaction conditions gave **15** in slightly lower yields (68%). For the subsequent debenzylation of **15** Pearlman’s catalyst^28^ and a hydrogen pressure of 40 bar proved to be the conditions of choice. At lower pressure or when employing Pd/C, only slow conversion of **15** to alcohol **16** was observed. Oxidation of **16** was accomplished under Swern conditions^29,30^ to provide aldehyde **17** in 64% yield over two steps. Surprisingly, for the subsequent conversion of **17** to dimethyl acetal **18** most standard conditions failed (see Supporting Information for details). After extensive experimentation, we found that the use of acidic Dowex resin in combination with trimethyl orthoformate was highly effective to give **18** in 70% yield. Subsequent treatment with KHMDS and allyl iodide at 23 °C completed the installation of the vicinal quaternary stereocenters in 64% yield. Noteworthy, at standard cryogenic temperatures either no reaction took place, or an intermolecular Claisen-type addition was observed.^31^ Finally, aldehyde **21** was obtained in 95% yield by oxidative cleavage (O_3_, then PPh_3_) of remote alkene **19**. With robust access to aldehyde **21**, we continued our synthesis by first performing a Pinnick–Lindgren–Kraus oxidation^32^ to access acid **8**. Subsequent treatment of **8** with Yamaguchi’s reagent,^33^ NEt_3_ and TBS-hydroquinone **24** afforded ester **7** in 97% yield over 2 steps. To access the 1,2,4-trisubsituted phenyl group inherent to the *Ganoderma* meroterpenoids we resorted to a Fries rearrangement.^34^ Since the use of standard conditions involving Lewis acids (e.g., AlCl_3_, BF_3_·OEt, TiCl_4_) was considered to be too harsh for both the silyl and the acetal protecting groups we opted for the rare photochemical variant.^35−37^ To our delight, irradiation of **7** at 254 nm in *n*-hexane (see Supporting Information for details) afforded the rearranged product **26** in 50% yield despite competing substrate decomposition. To complete the synthesis of applanatumol E (**2**), **26** was treated with NEt_3_·3HF to give **2** in 95% yield. The analytical data for **2** (^1^H NMR, ^13^C NMR, HRMS) fully matched those reported for the natural compound.^5^ We were also able to convert applanatumol E (**2**) to lingzhilactone B (**3**) in 55% yield by means of acetal removal employing *p*-TsOH in the presence of aqueous acetone. Lingzhilactone B (**3**) was further oxidized under Pinnick–Lindgren–Kraus conditions to deliver applanatumol I (**29**) in 78% yield. To access applanatumol H (**28**) we performed a direct allylation of lactone **15** to give alkene **20**, followed by ozonolysis to afford aldehyde **22**.^19^ Following the established conditions ester **25** was obtained in two additional steps. The photo-Fries reaction of **25** to **27** and the subsequent deprotection sequence proceeded with the same efficiency and high yields, ultimately enabling the synthesis of applanatumol H (**28**). In addition, meroapplanin B (**4**) was accessible in 85% yield when a solution of **3** in methanol was treated with NH_4_OAc at 50 °C. It might be noteworthy, that attempts to form the meroapplanin B (**4**) scaffold from **26** failed under those conditions.

With access to applanatumol I (**29**), we wondered if transformation to lingzhiol (**5**) would be feasible by means of an oxidative decarboxylation/Friedel–Crafts sequence. For the investigation of this transformation in the chemical laboratory, we first prepared phenol **32** from acid **8** (49% yield over two steps) through the well-established esterification/photo-Fries sequence (Scheme 3). To reduce the risk of overoxidation of the delicate phenol during the key-step, we protected the remaining phenolic hydroxy group as a methyl ether (K_2_CO_3_, MeI, 96%) to give **33**. Acetal removal with *p*-TsOH gave aldehyde **34** which was further oxidized to acid **35** (81%). Based on recent work by Doyle on the photocatalytic fluorination of redoxactive esters,^38^ the intermediate acid **35** was converted to the *N*-hydroxyphthalimide ester **36** (92%). Fortunately, by employing the Ir(dFppy)_3_ catalyst (10 mol %) in combination with a catalytic amount of NEt_3_•3 HF at 419 nm (blue light), **36** was cleanly converted to tetralone **37** in 71% yield. According to the mechanistic proposal, an initial single electron reduction forms an intermediate carboxyl radical **IV**. After extrusion of carbon dioxide, a single electron oxidation gives a stabilized tertiary carbocation **V**. This is then attacked by the arene in a Friedel–Crafts reaction to give tetralone **37**.

**Scheme 3:**
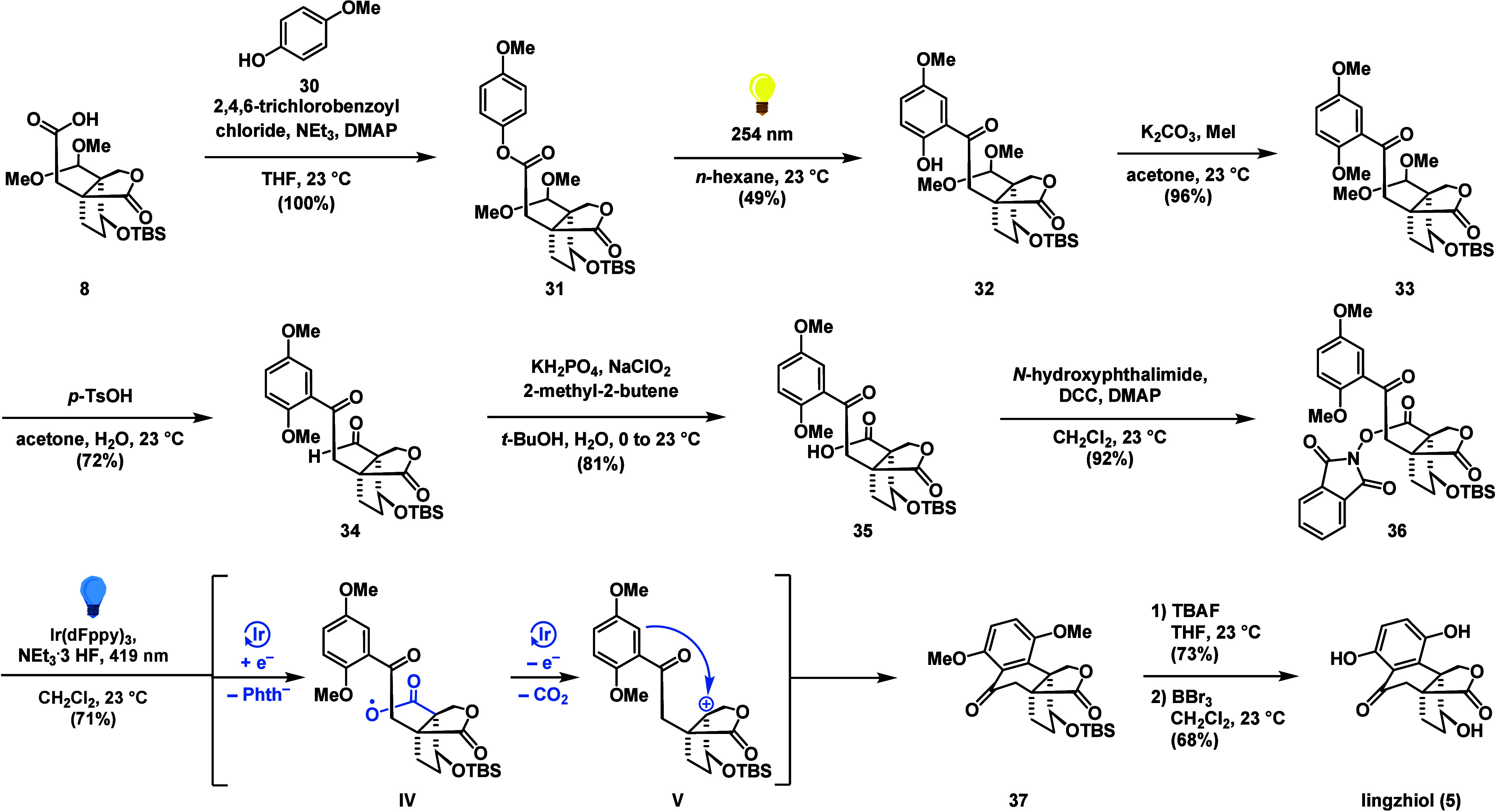
Synthesis of Lingzhiol (**5**) via a Photo-Fries Rearrangement and an Oxidative Decarboxylation/Friedel–Crafts Sequence

Global deprotection of the silyl protecting group (TBAF) and the methyl ethers (BBr_3_) afforded the natural product lingzhiol (**5**) in 50% yield over 2 steps. The spectroscopic data (^1^H and ^13^C NMR, HRMS) for **5** were in full agreement with those reported for the naturally occurring substance.^8^

In conclusion, we accomplished the total synthesis of six *Ganoderma* meroterpenoids. The robust route features the formation of the 1,2,4-trisubstituted benzene ring by employing a powerful, yet rare photo-Fries rearrangement. The natural product lingzhiol (**5**) was synthesized by photoredox catalysis that enabled an efficient oxidative decarboxylation/Friedel–Crafts sequence. The realization of this sequence highlights the synthetic potential of oxidative decarboxylation processes and constitutes a valuable starting point to access related *Ganoderma* natural products. Studies in this direction are currently underway in our laboratories and will be reported in due course.

## Data Availability

The data underlying this study are available in the published article and its Supporting Information.

## Supporting Information Available

Experimental procedures and characterization data for all new compounds as well as optimization tables and X-ray data (PDF)
