# Supplementary material for: A General Entry to Ganoderma Meroterpenoids: Synthesis of Applanatumol E, H, and I, Lingzhilactone B, Meroapplanin B, and Lingzhiol
Source: Org Lett. 2024 Oct 11;26(42):9017–21. doi: 10.1021/acs.orglett.4c03192 (PMC7616716; doi:10.1021/acs.orglett.4c03192)
Supplement: Supplementary file 1 — ol4c03192_si_001.pdf [file ol4c03192_si_001.pdf]

A General Entry to Ganoderma Meroterpenoids: Synthesis of Applanatumol E, H and I, Lingzhilactone B, Meroapplanin B and Lingzhiol

Alexander Rode,[a] Nicolas Müller,[a] Ondřej Kováč,[a,b] Klaus Wurst,[c] and Thomas Magauer[a,*]

[a]Department of Organic Chemistry and Center for Molecular Biosciences, University of Innsbruck, Innrain 80–82, 6020 Innsbruck, Austria

[b]Department of Organic Chemistry, Palacký University Olomouc, Tr. 17. Listopadu 12, 77900 Olomouc, Czech Republic

[c]Department of General Inorganic and Theoretical Chemistry, University of Innsbruck, 6020 Innsbruck, Austria

[*]E-mail: Thomas.magauer@uibk.ac.at

Table of Contents

1. General Experimental Details 4
2. Experimental Part 6
2.1. Synthesis of Unsuccessful Cyclization Precursors 6
Aldehyde S1 7
Acetal S2 8
Aldehyde 11 9
Allyl alcohol S3 10
Silyl ether S4 11
Malonate S5 12
Vinyl iodide S7 14
Vinyl iodide S8 15
Silyl ether S10 16
Malonate S11 18
Propellane S12 19
2.2. Synthesis of Aldehyde 19 20
Bundle's reagent S13 20
Benzyl ether 12 21
Allyl alcohol S14 22
Silyl ether 13 23
Malonate 10 24
Malonate 10 25
Ester 9 26
Lactone 15 27
Alcohol 16 28
Aldehyde 17 29
Ferrocene ester S15 30
Screening of Aldehyde Protection 31
Acetal 18 32

Lactone 19 33

Aldehyde 21 35

2.3. Total Syntheses of Applanatumol E, I, Lingzhilactone B and Meroapplanin B 36

Acid 8 36

Propellane S16 37

Phenol 24 38

Ester 7 39

UV-VIS Spectrum of Photo-Fries Precursor 7 40

Screening of Photo-Fries rearrangement 41

Ketone 26 42

Applanatumol E (2) 43

Lingzhilactone B (3) 46

Meroapplanin B (4) 49

Applanatumol I (29) 52

2.4. Total Synthesis of Applanatumol H (28) 55

Lactone 20 56

Aldehyde 22 57

Acid 23 58

Ester 25 59

Ketone 27 60

Alcohol S17 61

Applanatumol H (28) 62

2.5. Total Synthesis of Lingzhiol 65

Ester 31 65

Ketone 32 66

1,4-Dimethylhydroquinone 33 67

Aldehyde 34 68

Acid 35 69

N-(Acyloxy)phthalimide 36 70

Tetralone 37 ... 71

Alcohol S18 ... 72

Lingzhiol (5) ... 73

3. NMR Spectra ... 76

4. X-Ray Data ... 120

4.1. Ferrocene ester S15 ... 120

1. General Experimental Details

All reactions were carried out with magnetic stirring and, if moisture or air sensitive, under nitrogen or argon atmosphere using standard Schlenk techniques in oven-dried glassware (100 °C oven temperature). If required glassware was further dried under vacuum with a heat-gun at 650 °C. External bath thermometers were used to record all reaction temperatures. Low temperature reactions were carried out in a Dewar vessel filled with acetone and dry ice (–78 °C) or equipped with an electronically regulated cryostat in acetone (between –78 °C and 0 °C) or with distilled water and ice (0 °C). High temperature reactions were conducted in reaction vessels equipped with a reflux condenser or in a pressure tube using a heated silicon oil bath or a metal block. Tetrahydrofuran (THF) and diethyl ether were dried over molecular sieve (4 Å) prior to use. All other solvents were purchased from Acros Organics as 'extra dry' reagents. If required solvents were degassed by bubbling argon through the solvent with a balloon under sonication. All other reagents with a purity > 95% were obtained from commercial sources (Sigma Aldrich, Acros, Alfa Aesar and others) and used without further purification unless otherwise stated.

Flash column chromatography (FCC) was carried out with Merck silica gel 60 (0.040-0.063 mm). Analytical thin layer chromatography (TLC) was carried out using Merck silica gel 60 F254 aluminum foils and visualized under UV light at 254 nm. Staining was performed with ceric ammonium molybdate (CAM) or by staining with an aqueous potassium permanganate solution and subsequent heating.

NMR spectra (^{1}H NMR and ^{13}C NMR) were recorded in deuterated chloroform (chloroform-*d*), deuterated dichloromethane (dichloromethane-d_2) or deuterated acetone (acetone-d_6) on a Bruker Avance Neo 400 MHz spectrometer, or a Bruker Avance II 600 MHz spectrometer and are reported as follows: chemical shift δ in ppm (multiplicity, coupling constant *J* in Hz, number of protons) for ^{1}H NMR spectra and chemical shift δ in ppm for ^{13}C NMR spectra. Multiplicities are abbreviated as follows: s = singlet, d = doublet, t = triplet, q = quartet, br = broad, m = multiplet, or combinations thereof. For ^{1}H NMR the residual protic solvent peak served as internal reference (chloroform-*d*: 7.26 ppm, dichloromethane-d_2: 5.32 ppm, methanol-d_4: 3.31 ppm for the signal with the lowest shift and acetone-d_6: 2.05 ppm for the signal with the lowest shift). For ^{13}C NMR the central carbon resonance of chloroform-*d* (77.16 ppm), dichloromethane-d_2 (54.00 ppm), methanol-d_4 (49.00 ppm) or acetone-d_6 (206.26 ppm for the signal with the highest shift) served as internal reference. NMR spectra were assigned using information ascertained from COSY, HMBC, HSQC and NOESY experiments.

High resolution mass spectra (HRMS) were recorded on a Thermo Scientific™ LTQ Orbitrap XL™ Hybrid Ion Trap-Orbitrap Mass Spectrometer at the Institute of Organic Chemistry and Center for Molecular Biosciences, University of Innsbruck.

Infrared spectra (IR) were recorded from 4000 cm^{-1} to 450 cm^{-1} on a Bruker™ ALPHA FT-IR Spectrometer from Bruker. Samples were prepared as a neat film or a film by evaporation of a solution

in chloroform-*d*, dichloromethane. IR data in frequency of absorption (cm^{-1}) is reported as follows: w = weak, m = medium, s = strong, br = broad or combinations thereof.

Melting points were measured with an SRS MPA120 EZ-Melt Melting Point Apparatus in open glass capillaries.

X-Ray diffraction analysis was carried out by Dr. Klaus Wurst at the Institute of Inorganic and Theoretical Chemistry and Center for Molecular Biosciences, University of Innsbruck. The data collections were performed on a Bruker D8 Quest diffractometer (Photon 100 detector) equipped with a microfocus source generator (Incoatec GmbH, Geesthacht, Germany) combined with multi-layer optics (monochromatized Mo Kα radiation, λ = 71.073 pm). The Bruker Apex III software was applied for the integration, scaling and multi-scan absorption correction of the data. The structure was solved with SHELXS[1] (version 2013/1). Structure refinement (full-matrix least-squares against F^2) with SHELXL[2] (version 2014/7). All nonhydrogen atoms were refined anisotropically. The hydrogen atoms were placed in ideal geometry riding on their parent atoms. Relevant details of the data collection and evaluation are listed in chapter 4. Supplementary crystallographic data for **S15** may be obtained free of charge from the Cambridge Crystallographic Data Centre CCDC deposition service via www.ccdc.cam.ac.uk/structures on quoting the deposition number CCDC 2055903. Plotting of thermal ellipsoids in this document and in the main text was carried out using MERCURY for Windows at 50% probability level.

All yields are isolated, unless otherwise specified.

[1] G. M. Sheldrick, *Acta Crystallogr. Sect. Found. Adv.* **2015**, *71*, 3–8.
[2] G. M. Sheldrick, *Acta Crystallogr. Sect. C Struct. Chem.* **2015**, *71*, 3–8.

2. Experimental Part

2.1. Synthesis of Unsuccessful Cyclization Precursors

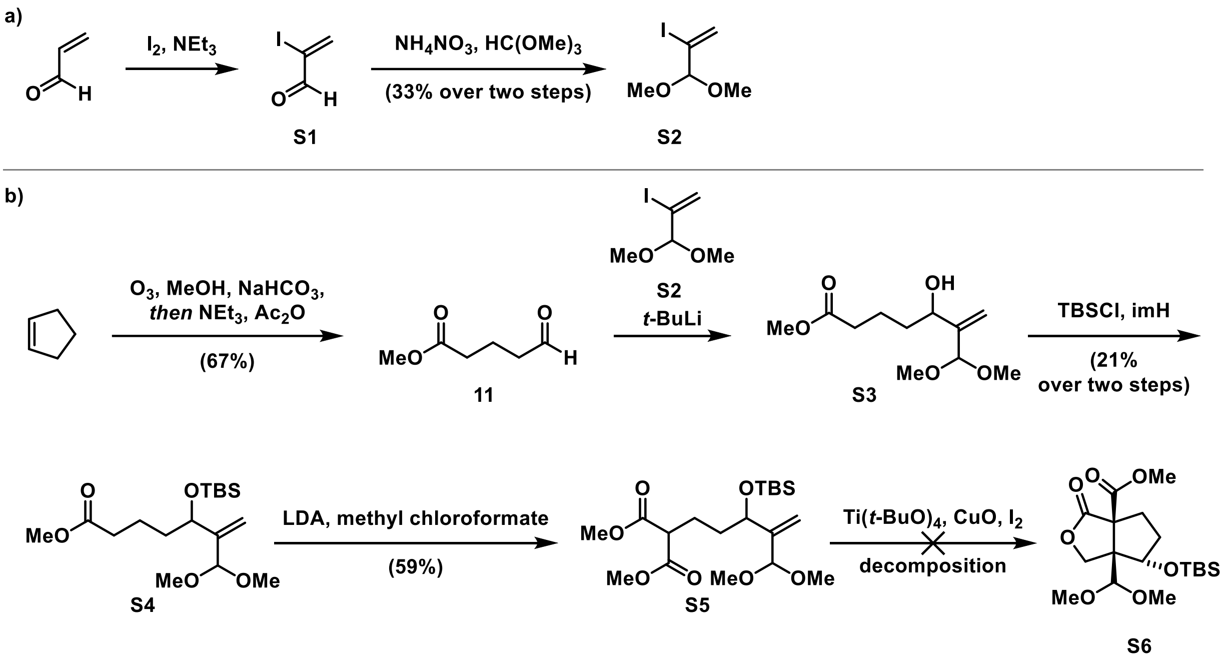

Scheme 1: Synthesis of vinyl iodide **S2** (a) and attempted iodo-carbocyclization to access bicycle **S6** (b).

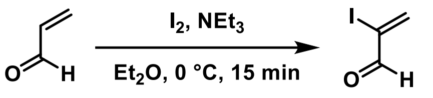

S1

Aldehyde S1

To a solution of acrolein (2.00 g, 35.7 mmol, 2.00 equiv) in diethyl ether (50 mL) was added triethylamine (2.48 mL, 17.8 mmol, 1 equiv) at 0 °C. Iodine (4.57 g, 17.8 mmol, 1 equiv) was added portion wise to the solution at 0 °C. After 15 minutes, the reaction mixture was filtered through a pad of Celite and the pad was washed with diethyl ether (20 mL). The filtrate was concentrated at 23 °C to yield aldehyde **S1** (2.18 g, 12.0 mmol) as a slightly yellow oil. The crude product was used in the next step without further purification. The obtained analytical data were in full agreement with those reported in the literature.[3]

[3] B. V. D. Vijaykumar, P. Mallesham, S. Chandrasekhar, *Eur. J. Org. Chem.* **2012**, *2012*, 988–994.

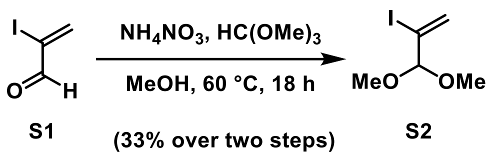

Acetal S2

To a solution of aldehyde **S1** (2.18 g, 12.0 mmol, 1 equiv) in methanol (3 mL) was added ammonium nitrate (47.9 mg, 599 µmol, 0.0500 equiv) and trimethyl orthoformate (1.34 mL, 12.0 mmol, 1 equiv) and the reaction mixture was heated to 60 °C. After 18 hours, the reaction mixture was allowed to cool to 23 °C and was then concentrated. Sodium carbonate (0.5 g) was added to the residue and the mixture was purified by bulb-to-bulb distillation (0.1 mmHg, 125 °C, the product was collected in a flask cooled to –78 °C and protected from light) to yield acetal **S2** (1.35 g, 5.92 mmol, 33% over two steps) as a slightly yellow oil.

TLC (20% ethyl acetate in cyclohexane): R_f = 0.58 ($KMnO_4$).

^{1}H NMR (400 MHz, chloroform-*d*) δ 6.57 (dd, *J* = 1.5, 1.1 Hz, 1H), 6.09 (dd, *J* = 1.5, 0.6 Hz, 1H), 4.38 (t, *J* = 0.9 Hz, 1H), 3.35 (s, 6H).

^{13}C NMR (101 MHz, chloroform-*d*) δ 128.9, 107.9, 105.4, 53.6.

IR (Diamond-ATR, neat) $\tilde{\nu}_{max}$: 2929 (s), 2865 (m), 1698 (m), 1464 (w), 1090 (m), 884 (w), 860 (w), 572 (w), 542 (w) cm^{-1}.

HRMS (ESI) no mass found.

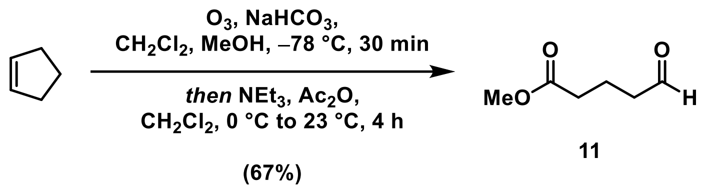

11

Aldehyde 11

To a solution of cyclopentene (6.60 mL, 72.0 mmol, 1 equiv) in dichloromethane (250 ml) and methanol (50 mL) was added sodium bicarbonate (1.94 g, 23.0 mmol, 0.320 equiv) and the mixture was cooled to −78 °C. Ozone was sparged through the reaction mixture until a blue colour appeared. After 30 minutes, oxygen was sparged through the mixture until the blue colour disappeared. The reaction mixture was filtered and to the filtrate was added benzene (80 mL). The solution was concentrated until approximately 50 mL of solvent remained. Dichloromethane (250 mL) was added and the mixture was cooled to 0 °C. To the ice-cold mixture was added triethylamine (15.0 mL, 108 mmol, 1.50 equiv) and acetic anhydride (22.1 g, 216 mmol, 3.00 equiv). After 15 minutes, the reaction mixture was allowed to warm to 23 °C. After four hours at 23 °C, aqueous hydrochloric acid (0.1 M, 500 mL) was added to the reaction mixture and the layers were separated. The aqueous layer was extracted with ethyl acetate (3 × 150 mL) and the combined organic layers were dried over sodium sulfate. The dried solution was filtered and the filtrate was concentrated. The residue was purified by flash column chromatography on silica gel (15% ethyl acetate in hexanes) to yield aldehyde **11** (6.30 g, 48.4 mmol, 67%) as a colourless liquid. The obtained analytical data were in full agreement with those reported in the literature.[4]

[4] J. Chen, J. Chen, Y. Xie, H. Zhang, *Angew. Chem. Int. Ed.* **2012**, *51*, 1024–1027.

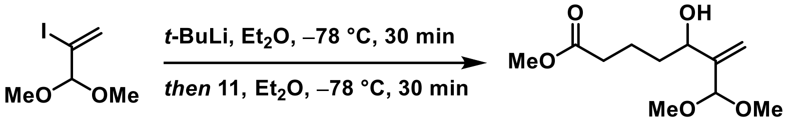

Allyl alcohol S3

To a solution of *tert*-butyllithium (1.50 M in pentane, 1.11 mL, 1.67 mmol, 2.40 equiv) in diethyl ether (2 mL) was added a solution of vinyl iodide **S2** (190 mg, 835 µmol, 1.20 equiv) in diethyl ether (2 mL) dropwise over the course of 15 minutes at −78 °C. 30 minutes after the addition of **S2** was completed, a solution of aldehyde **11** (90.6 mg, 696 µmol, 1 equiv) in diethyl ether (2 mL) was added to the yellow mixture over the course of ten minutes. 15 minutes after the addition of **12** was completed, saturated aqueous solution of ammonium chloride (20 mL) was added to the reaction mixture and the solution was allowed to warm to 23 °C. The layers were separated and the aqueous layer was extracted with ethyl acetate (3 × 10 mL). The combined organic layers were dried over sodium sulfate, the dried solution was filtered and the filtrate was concentrated to obtain **S3** as a colourless oil (88 mg). The residue was used crude in the next reaction.

Note: The allylic alcohol is slightly unstable on silica gel. A small sample was purified on silica gel (30% ethyl acetate in cyclohexane) to obtain an analytical pure product.

TLC (30% ethyl acetate in cyclohexane): R_f = 0.11 ($KMnO_4$).

^{1}H NMR (400 MHz, chloroform-*d*) δ 5.27 – 5.23 (m, 2H), 4.72 (s, 1H), 4.17 – 4.12 (m, 1H), 3.62 (s, 3H), 3.32 – 3.27 (m, 6H), 2.65 (d, *J* = 4.7 Hz, 1H), 2.33 – 2.28 (m, 2H), 1.77 – 1.70 (m, 1H), 1.66 – 1.58 (m, 3H).

^{13}C NMR (101 MHz, chloroform-*d*) δ 174.1, 146.2, 114.6, 104.7, 71.2, 54.0, 53.6, 51.5, 35.0, 33.8, 21.3.

IR (Diamond-ATR, neat) $\tilde{\upsilon}_{max}$: 3485 (br, w), 2929 (m), 1737 (s), 1438 (m), 1367 (w), 1195 (m), 1112 (m), 1074 (m), 983 (w) cm^{-1}.

HRMS (ESI) calc. for $C_{11}H_{20}NaO_5$ $[M+Na]^+$: 255.1203 found: 255.1198; calc. for $C_{11}H_{20}KO_5$ $[M+K]^+$: 271.0942 found: 271.0937.

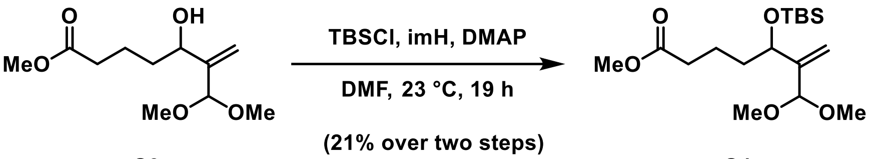

S3 S4

Silyl ether S4

To a solution of crude allyl alcohol **S3** (88.0 mg, 379 µmol, 1 equiv) in *N*,*N*-dimethylformamide (3 mL) was added *tert*-butyldimethylchlorosilane (68.5 mg, 455 µmol, 1.20 equiv), imidazole (61.9 mg, 909 µmol, 2.40 equiv) and 4-(dimethylamino)pyridine (4.68 mg, 38.0 µmol, 0.100 equiv) at 23 °C. After 19 hours, water (5 mL) and diethyl ether (5 mL) were added to the reaction mixture and the layers were separated. The aqueous layer was extracted with diethyl ether (3 × 3 mL), the combined organic layers were washed with an aqueous solution of lithium chloride (10%, 5 mL) and the washed organic layers were dried over sodium sulfate. The dried solution was filtered and the filtrate was concentrated. The residue was purified by flash column chromatography on silica gel (10% ethyl acetate in cyclohexane) to yield silyl ether **S4** (50.5 mg, 146 µmol, 21% over two steps) as a colourless oil.

TLC (20% ethyl acetate in cyclohexane): R_f = 0.24 ($KMnO_4$).

^{1}H NMR (600 MHz, chloroform-*d*) δ 5.35 (t, *J* = 1.7 Hz, 1H), 5.29 – 5.26 (m, 1H), 4.70 (s, 1H), 4.25 – 4.21 (m, 1H), 3.66 (s, 3H), 3.34 (s, 3H), 3.25 (s, 3H), 2.33 – 2.27 (m, 2H), 1.68 – 1.57 (m, 3H), 1.55 – 1.49 (m, 1H), 0.90 (s, 9H), 0.06 – –0.01 (m, 6H).

^{13}C NMR (151 MHz, chloroform-*d*) δ 174.3, 147.0, 113.9, 103.2, 71.5, 54.4, 52.4, 51.6, 36.5, 34.2, 26.0, 20.5, 18.3, –4.5, –4.9.

IR (Diamond-ATR, neat) $\tilde{\upsilon}_{max}$: 2953 (m), 2930 (m), 2857 (w), 1741 (s), 1463 (w), 1253 (m), 1193 (w), 1091 (s), 994 (w), 837 (m), 776 (m) cm^{-1}.

HRMS (ESI) calc. for $C_{17}H_{34}NaO_5Si$ $[M+Na]^+$: 369.2068 found: 369.2061.

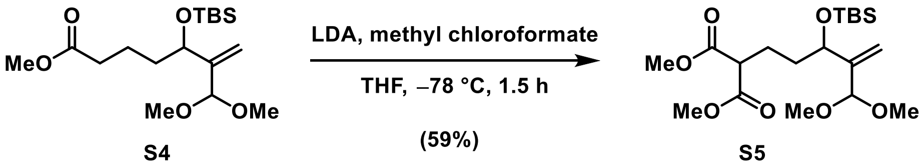

Malonate S5

To a solution of silyl ether **S4** (50.5 mg, 146 µmol, 1 equiv) in THF (0.8 mL) was added dropwise a solution of lithium diisopropylamide (1.00 M in tetrahydrofuran, 146 µL, 87.0 µmol, 1.00 equiv) at −78 °C. After 30 minutes, to the yellow mixture was added a solution of lithium diisopropylamide (1.00 mM in tetrahydrofuran, 160 µL, 160 µmol, 1.10 equiv). After ten minutes, methyl chloroformate (23.6 µL, 291 µmol, 2.00 equiv) was added to the reaction mixture. After one hour, a saturated aqueous solution of ammonium chloride (5 mL) and diethyl ether (5 mL) were added to the reaction mixture and the layers were separated. The aqueous layer was extracted with ethyl acetate (3 × 3 mL), the combined organic layers were dried over sodium sulfate. The dried solution was filtered and the filtrate was concentrated. The residue was purified by flash column chromatography on silica gel (30% ethyl acetate in cyclohexane) to yield malonate **S5** (34.8 mg, 86.0 µmol, 59%) as a colourless oil.

TLC (30% ethyl acetate in cyclohexane): R_f = 0.26 ($KMnO_4$).

^{1}H NMR (700 MHz, chloroform-*d*) δ 5.36 – 5.35 (m, 1H), 5.28 (dt, *J* = 2.0, 1.0 Hz, 1H), 4.67 (s, 1H), 4.27 – 4.24 (m, 1H), 3.71 (m, 6H), 3.36 (t, *J* = 7.6 Hz, 1H), 3.33 (s, 3H), 3.24 (s, 3H), 1.92 (ddt, *J* = 9.8, 7.4, 5.4 Hz, 2H), 1.64 – 1.58 (m, 1H), 1.57 – 1.52 (m, 1H), 0.90 (s, 9H), 0.05 – –0.02 (m, 6H).

^{13}C NMR (176 MHz, chloroform-*d*) δ 170.1, 170.0, 146.6, 114.3, 103.3, 71.0, 54.4, 52.6, 52.5, 52.5, 51.7, 34.3, 26.0, 24.2, 18.3, –4.6, –4.9.

IR (Diamond-ATR, neat) $\tilde{\upsilon}_{max}$: 2954 (m), 2930 (m), 2857 (w), 1739 (s), 1437 (w), 1254 (m), 1156 (m), 1092 (s), 991 (w), 836 (m), 777 (m) cm^{-1}.

HRMS (ESI) calc. for $C_{19}H_{36}NaO_7Si$ $[M+Na]^+$: 427.2123 found: 427.2113.

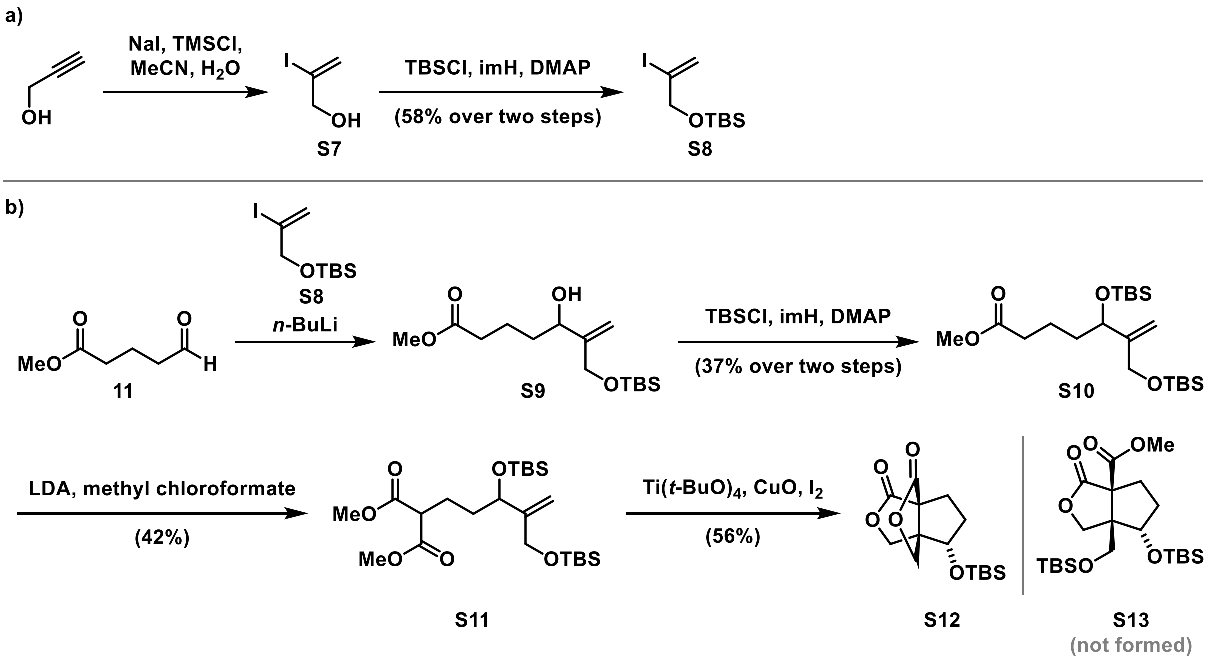

Scheme 2*:* Synthesis of vinyl iodide **S8** and iodo-carbocyclization of malonate **S11** formed propellane **S12**.

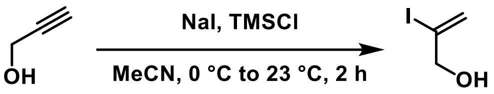

S7

Vinyl iodide S7

To a suspension of sodium iodide (121 g, 804 mmol, 1.20 equiv) in acetonitrile (1 L) was added trimethylsilyl chloride (105 mL, 804 mmol, 1.20 equiv) at 0 °C. After 15 minutes, water (7.25 mL, 402 mmol, 0.600 equiv) and propargyl alcohol (30 mL, 503 mmol, 1 equiv) were added and the reaction was allowed to warm to 23 °C. After two hours at 23 °C, a saturated aqueous solution of sodium hydrogen carbonate (1 L) was added to the reaction mixture and the layers were separated. The aqueous layer was extracted with ethyl acetate (3 × 300 mL). The combined organic layers were washed with a saturated aqueous solution of sodium thiosulfate (500 mL) and a saturated aqueous solution of sodium chloride (500 mL) in sequence. The washed organic layers were dried over sodium sulfate. The dried solution was filtered and the filtrate was concentrated to yield vinyliodide **S7** (123 g, 670 mmol) as a slightly yellow liquid. The crude reaction mixture was used in the next step without further purification. The obtained analytical data were in full agreement with those reported in the literature.[5]

[5] M. Kurosu, M.-H. Lin, Y. Kishi, *J. Am. Chem. Soc.* **2004**, *126*, 12248–12249.

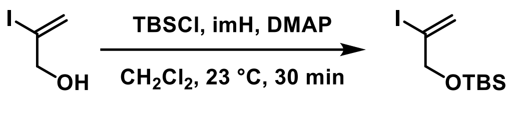

S7 S8

Vinyl iodide S8

To a solution of crude vinyl iodide **S7** (3.04 g, 16.5 mmol, 1 equiv) in dichloromethane (160 mL) was added *tert*-butyldimethylchlorosilane (3.05 g, 19.8 mmol, 1.20 equiv), imidazole (1.36 g, 19.8 mmol, 1.20 equiv) and 4-dimethylaminopyridine (204 mg, 1.65 mmol, 0.100 equiv) at 23 °C. After 30 minutes, water (100 mL) was added to the reaction mixture and the layers were separated. The aqueous layer was extracted with dichloromethane (3 × 30 mL), the combined organic layers were dried over sodium sulfate. The dried solution was filtered and the filtrate was concentrated. The residue was purified by flash column chromatography on silica gel (cyclohexane) to yield vinyl iodide **S8** (2.87 g, 9.62 mmol, 58% over two steps) as a colourless oil. The obtained analytical data were in full agreement with those reported in the literature.[6]

[6] T. Smeilus, F. Mousavizadeh, J. Krieger, X. Tu, M. Kaiser, A. Giannis, *Beilstein J. Org. Chem.* **2019**, *15*, 567–570.

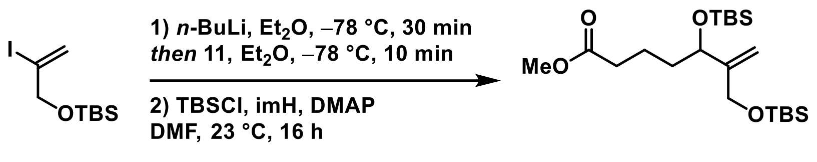

S8 (37% over two steps) S10

Silyl ether S10

To a solution of *n*-butyllithium (2.44 M in hexane, 1.05 mL, 2.57 mmol, 1.15 equiv) in diethyl ether (7 mL) was added a solution of vinyl iodide **S8** (767 mg, 2.57 mmol, 1.15 equiv) in diethyl ether (4 mL) dropwise over the course of 30 minutes at −78 °C. 30 minutes after the addition of **S8** was completed, a solution of aldehyde **11** (291 mg, 2.24 mmol, 1 equiv) in diethyl ether (2 mL) was added to the reaction mixture over the course of ten minutes. 30 minutes after the addition of **12** was completed, a saturated aqueous solution of ammonium chloride (20 mL) was added to the mixture and the solution was allowed to warm to 23 °C. The layers were separated and the aqueous layer was extracted with ethyl acetate (3 × 10 mL). The combined organic layers were dried over sodium sulfate, the dried solution was filtered and the filtrate was concentrated to yield allylic alcohol **S9** (291 mg, 962 µmol) as a colourless oil.

To a solution of the crude allylic alcohol **S9** (291 mg, 962 µmol, 1 equiv) in *N*,*N*-dimethylformamide (10 mL) was added *tert*-butyldimethylchlorosilane (222 mg, 1.44 mmol, 1.50 equiv), imidazole (99.2 mg, 1.44 mmol, 1.50 equiv) and 4-(dimethylamino)pyridine (11.9 mg, 96.0 µmol, 0.100 equiv) at 23 °C. After six hours, water (20 mL) and diethyl ether (20 mL) were added to the reaction mixture and the layers were separated. The aqueous layer was extracted with diethyl ether (3 × 10 mL), the combined organic layers were washed with a 10% aqueous solution of lithium chloride (20 mL) and the washed organic layers were dried over sodium sulfate. The dried solution was filtered and the filtrate was concentrated. The residue was purified by flash column chromatography on silica gel (4% grading to 10% ethyl acetate in cyclohexane) to yield silyl ether **S10** (349 mg, 837 µmol, 37% over two steps) as a colourless oil.

TLC (10% ethyl acetate in cyclohexane): R_f = 0.56 (CAM).

^{1}H NMR (400 MHz, chloroform-*d*) δ 5.11 – 5.07 (m, 1H), 5.03 – 5.00 (m, 1H), 4.20 (t, *J* = 5.8 Hz, 1H), 4.18 – 4.11 (m, 2H), 3.66 (s, 3H), 2.30 (t, *J* = 7.3 Hz, 2H), 1.71 – 1.63 (m, 1H), 1.62 – 1.50 (m, 3H), 0.92 – 0.87 (m, 18H), 0.06 (s, 6H), 0.05 – 0.00 (m, 6H).

^{13}C NMR (101 MHz, chloroform-*d*) δ174.2, 150.6, 109.6, 74.0, 62.8, 51.6, 36.4, 34.1, 26.1, 26.0, 21.1, 18.5, 18.3, –4.6, –4.9, –5.2, –5.3.

IR (Diamond-ATR, neat) $\tilde{\upsilon}_{max}$: 2954 (m), 2930 (m), 2857 (m), 1743 (m), 1463 (w), 1253 (m), 1158 (w), 1079 (m), 1005 (m), 836 (s), 776 (s).

HRMS (ESI) calc. for $C_{21}H_{44}NaO_4Si_2$ $[M+Na]^+$: 439.2670 found: 439.2648.

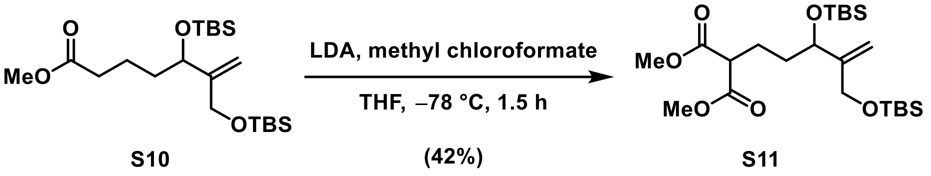

Malonate S11

To a solution of silyl ether **S10** (349 mg, 837 µmol, 1 equiv) in tetrahydrofuran (8 mL) was added dropwise a solution of lithium diisopropylamide (1.00 M in tetrahydrofuran, 837 µL, 837 µmol, 1.00 equiv) at −78 °C. After 30 minutes, a solution of lithium diisopropylamide (1 M in tetrahydrofuran, 921 µL, 921 µmol, 1.10 equiv) was added to the yellow mixture. After ten minutes, methyl chloroformate (132 µL, 1.67 mmol, 2.00 equiv) was added to the reaction mixture. After one hour, a saturated aqueous solution of ammonium chloride (20 mL) was added to the reaction mixture and the layers were separated. The aqueous layer was extracted with diethyl ether (3 × 10 mL), the combined organic layers were dried over sodium sulfate. The dried solution was filtered and the filtrate was concentrated. The residue was purified by flash column chromatography on silica gel (3% ethyl acetate in cyclohexane) to yield malonate **S11** (165 mg, 348 µmol, 42%) as a slightly yellow oil.

TLC (3% ethyl acetate in cyclohexane): R_f = 0.16 (CAM).

^{1}H NMR (600 MHz, chloroform-*d*) δ 5.11 – 5.09 (m, 1H), 5.05 – 5.03 (m, 1H), 4.23 (t, *J* = 5.9 Hz, 1H), 4.14 (dt, *J* = 8.7, 1.6 Hz, 2H), 3.73 (s, 6H), 3.36 (t, *J* = 7.5 Hz, 1H), 1.97 – 1.91 (m, 1H), 1.90 – 1.84 (m, 1H), 1.55 – 1.54 (m, 2H), 0.91 (s, 9H), 0.89 (s, 9H), 0.06 (s, 6H), 0.04 – 0.01 (m, 6H).

^{13}C NMR (151 MHz, chloroform-*d*) δ 170.0, 169.9, 150.1, 110.0, 73.5, 62.8, 52.6, 51.7, 34.3, 26.1, 26.0, 24.9, 18.5, 18.3, –4.6, –5.0, –5.2, –5.3.

IR (Diamond-ATR, neat) $\tilde{\upsilon}_{max}$: 2930 (m), 2857 (m), 1739 (m), 1463 (w), 1252 (m), 1152 (m), 1079 (m), 1006 (w), 834 (s), 774 (s) cm^{-1}.

HRMS (ESI) calc. for $C_{23}H_{46}NaO_6Si_2$ $[M+Na]^+$: 497.2725 found: 497.2718.

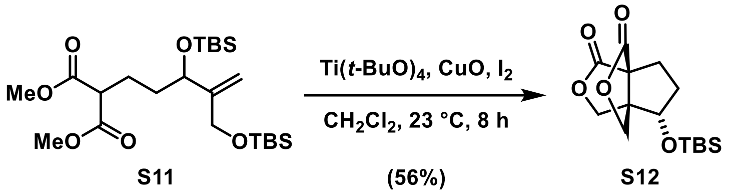

Propellane S12

To a solution of malonate **S11** (42.0 mg, 89.0 µmol, 1 equiv) in dichloromethane (1 mL) was added Ti(*t*-BuO)$_4$ (41.0 µL, 106 µmol, 1 equiv) at 23 °C. After 15 minutes, iodine (90.7 mg, 354 µmol, 4.00 equiv) and cupric oxide (10.1 g, 124 µmol, 1.40 equiv) were added. After eight hours, a saturated aqueous solution of sodium thiosulfate (5 mL) was added to the red suspension and the layers were separated. The aqueous layer was extracted with dichloromethane (3 × 2 mL) and the combined organic layers were dried over sodium sulfate. The dried solution was filtered over a pad of Celite and the filtrate was concentrated. The residue was purified by flash column chromatography on silica gel (40% ethyl acetate in cyclohexane) to yield propellane **S12** (15.5 mg, 50.0 µmol, 56%) as a slightly yellow oil.

TLC (50% ethyl acetate in cyclohexane): R_f = 0.32 (CAM).

^{1}H NMR (600 MHz, chloroform-*d*) δ 4.75 (d, *J* = 9.8 Hz, 1H), 4.40 (d, *J* = 10.1 Hz, 1H), 4.26 (t, *J* = 4.3 Hz, 1H), 4.21 (d, *J* = 9.8 Hz, 1H), 4.13 (d, *J* = 10.1 Hz, 1H), 2.53 – 2.48 (m, 1H), 2.39 – 2.35 (m, 1H), 2.06 – 2.03 (m, 1H), 1.98 – 1.94 (m, 1H), 0.88 (s, 9H), 0.10 (s, 6H).

^{13}C NMR (151 MHz, chloroform-*d*) δ 172.0, 171.9, 78.8, 74.5, 71.5, 60.8, 36.0, 32.3, 25.7, 18.0, –4.3, –4.9.

IR (Diamond-ATR, neat) $\tilde{\upsilon}_{max}$: 2955 (w), 2929 (w), 2856 (w), 1782 (s), 1746 (m), 1254 (m), 1206 (m), 1143 (m), 1071 (w), 1020 (m), 836 (m), 778 (m) cm^{-1}.

HRMS (ESI) calc. for $C_{15}H_{24}NaO_5Si$ [M+Na]$^+$: 335.1285 found: 335.1282.

2.2. Synthesis of Aldehyde 19

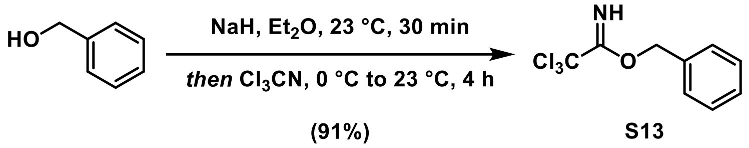

Bundle's reagent S13

To a solution of benzyl alcohol (24.7 mL, 238 mmol, 1 equiv) in diethyl ether (500 mL) was added sodium hydride (6.41 g, 262 mmol, 1.10 equiv) at 23 °C. After 30 minutes, the reaction mixture was cooled to 0 °C and trichloroacetonitrile (26.8 mL, 262 mmol, 1.10 equiv) was added to the solution. After 15 minutes, the reaction mixture was allowed to warm to 23 °C. After four hours at 23 °C, water (500 mL) was slowly added to the reaction mixture and the layers were separated. The aqueous layer was extracted with diethyl ether (3 × 150 mL) and the combined organic layers were dried over sodium sulfate. The dried solution was filtered and the filtrate was concentrated to yield Bundle's reagent **S13** (54.4 g, 215 mmol, 91%) as a brownish oil. The obtained analytical data were in full agreement with those reported in the literature.[7]

[7] C. Li, W. Li, J. Wang, *Tetrahedron Lett.* **2009**, *50*, 2533–2535.

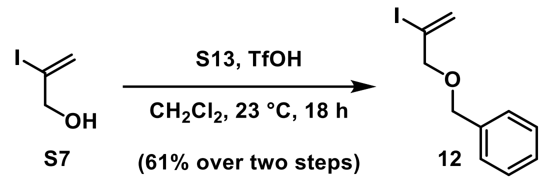

Benzyl ether 12

To a solution of crude vinyl iodide **S7** (37.4 g, 203 mmol, 1 equiv) in dichloromethane (500 mL) was added Bundle´s reagent **S13** (56.5 g, 224 mmol, 1.10 equiv) at 23 °C. To this reaction mixture was added trifluoromethanesulfonic acid (1.81 mL, 20.3 mmol, 0.100 equiv). After 18 hours, a saturated aqueous solution of sodium bicarbonate (500 mL) was added to the reaction mixture and the layers were separated. The aqueous layer was extracted with diethyl ether (3 × 150 mL) and the combined organic layers were dried over sodium sulfate. The dried solution was filtered and the filtrate was concentrated. The residue was purified by flash column chromatography on silica gel (10% grading to 20% ethyl acetate in cyclohexane) to yield benzyl ether **12** (33.8 g, 123 mmol, 61% over two steps) as a slightly yellow oil. The obtained analytical data were in full agreement with those reported in the literature.[8]

[8] M. T. Riaz, I. Pohorilets, J. J. Hernandez, J. Rios, N. I. Totah, *Tetrahedron Lett.* **2018**, *59*, 2809–2812.

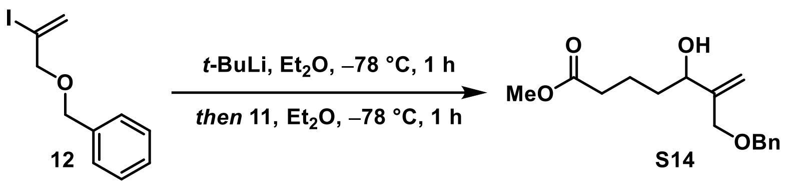

Allyl alcohol S14

To a solution of *tert*-butyllithium (1.60 M in pentane, 63.1 mL, 97.8 mmol, 2.00 equiv) in diethyl ether (400 mL) was added a solution of vinyl iodide **12** (13.4 g, 48.9 mmol, 1 equiv) in diethyl ether (100 mL) dropwise over the course of 30 minutes at −78 °C. 30 minutes after the addition of **12** was completed, a solution of aldehyde **11** (6.36 g, 48.9 mmol, 1 equiv) in diethyl ether (100 mL) was added to the yellow solution over the course of 30 minutes. 30 minutes after the addition of **11** was completed, a saturated aqueous solution of ammonium chloride (600 mL) was added to the reaction mixture and the solution was allowed to warm to 23 °C. The layers were separated and the aqueous layer was extracted with ethyl acetate (3 × 200 mL). The combined organic layers were dried over sodium sulfate, the dried solution was filtered and the filtrate was concentrated to yield allyl alcohol **S14** (13.6 g, 48.9 mmol) as a colourless oil. The crude reaction mixture was used in the next step without further purification.

Note: The product was found to undergo partial decomposition upon purification on silica gel. To obtain analytical data, only a small aliquot of the crude reaction mixture was purified by flash column chromatography on silica gel (30% ethyl acetate in cyclohexane).

TLC (30% ethyl acetate in cyclohexanes): R_f = 0.29 (CAM).

^{1}H NMR (400 MHz, chloroform-*d*) δ 7.37 – 7.31 (m, 4H), 5.19 (t, *J* = 1.2 Hz, 1H), 5.16 – 5.15 (m, 1H), 4.52 (s, 2H), 4.19 (t, *J* = 6.1 Hz, 1H), 4.16 – 4.12 (m, 1H), 4.08 – 4.03 (m, 1H), 3.66 (s, 3H), 2.36 – 2.32 (m, 2H), 1.79 – 1.72 (m, 1H), 1.68 – 1.59 (m, 3H).

^{13}C NMR (101 MHz, chloroform-*d*) δ 174.2, 147.1, 137.9, 128.6, 127.9, 127.9, 114.1, 73.7, 72.6, 71.5, 51.6, 35.1, 33.9, 21.2.

IR (Diamond-ATR, neat) $\tilde{\nu}_{max}$: 3468 (br, w), 2950 (w), 1735 (s), 1454 (w), 1365 (w), 1241 (m), 1072 (m), 738 (w), 699 (w) cm^{-1}.

HRMS (ESI) calc. for $C_{16}H_{22}NaO_4$ $[M+Na]^+$: 301.1410 found: 301.1391.

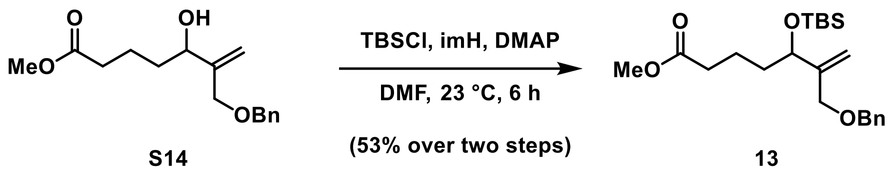

Silyl ether 13

To a solution of crude allyl alcohol **S14** (25.0 g, 89.8 mmol, 1 equiv) in *N*,*N*-dimethylformamide (900 mL) was added *tert*-butyldimethylsilyl chloride (14.9 g, 98.8 mmol, 1.10 equiv), imidazole (7.41 g, 108 mmol, 1.20 equiv) and 4-(dimethylamino)pyridine (1.11 g, 8.98 mmol, 0.100 equiv) at 23 °C. After six hours, water (450 mL) and diethyl ether (100 mL) were added to the reaction mixture and the layers were separated. The aqueous layer was extracted with diethyl ether (3 × 300 mL), the combined organic layers were washed with a 10% aqueous solution of lithium chloride (300 mL) and the washed organic layers were dried over sodium sulfate. The dried solution was filtered and the filtrate was concentrated. The residue was purified by flash column chromatography on silica gel (4% grading to 10% ethyl acetate in cyclohexane) to yield silyl ether **13** (18.7 g, 47.6 mmol, 53% over two steps) as a slightly yellow oil.

TLC (10% ethyl acetate in cyclohexane): R_f = 0.38 (CAM).

^{1}H NMR (400 MHz, chloroform-*d*) δ 7.37 – 7.33 (m, 5H), 5.18 – 5.15 (m, 2H), 4.54 – 4.47 (m, 2H), 4.23 (t, *J* = 5.6 Hz, 1H), 4.07 – 3.97 (m, 2H), 3.66 (s, 3H), 2.29 (t, *J* = 7.2 Hz, 2H), 1.70 – 1.60 (m, 2H), 1.58 – 1.54 (m, 2H), 0.88 (s, 9H), 0.05 – 0.00 (m, 6H).

^{13}C NMR (101 MHz, chloroform-*d*) δ 174.2, 147.9, 138.6, 128.5, 127.7, 127.7, 112.3, 73.9, 72.3, 70.0, 51.6, 36.1, 34.1, 26.0, 20.9, 18.3, –4.6, –4.9.

IR (Diamond-ATR, neat) $\tilde{\upsilon}_{max}$: 2953 (w), 2929 (w), 2856 (w), 1739 (s), 1454 (w), 1251 (m), 1087 (s), 835 (s), 776 (s), 698 (m) cm^{-1}.

HRMS (ESI) calc. for $C_{22}H_{36}NaO_4Si$ $[M+Na]^+$: 415.2275 found: 415.2250.

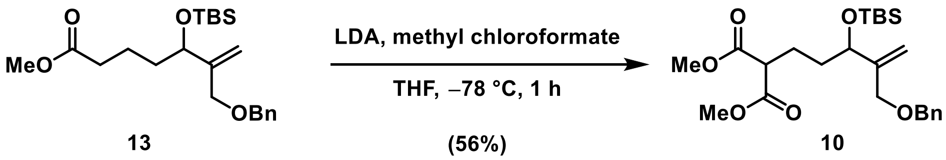

Malonate 10

To a solution of silyl ether **13** (18.7 g, 47.6 mmol, 1 equiv) in THF (150 mL) was added dropwise a solution of lithium diisopropylamide (1.00 M in tetrahydrofuran, 47.6 mL, 47.6 mmol, 1.00 equiv) at −78 °C over the course of 30 minutes. 30 minutes after the addition of the solution of lithium diisopropylamide was completed, a solution of lithium diisopropylamide (1.00 M in tetrahydrofuran, 52.4 mL, 52.4 mmol, 1.10 equiv) was added to the yellow mixture over the course of five minutes. Ten minutes after the addition of the lithium diisopropylamide solution was completed, methyl chloroformate (95.3 mmol, 7.36 mL, 2.00 equiv) was added to the reaction mixture. After one hour, a saturated aqueous solution of ammonium chloride (200 mL) was added to the reaction mixture and the layers were separated. The aqueous layer was extracted with diethyl ether (3 × 100 mL), the combined organic layers were dried over sodium sulfate. The dried solution was filtered and the filtrate was concentrated. The residue was purified by flash column chromatography on silica gel (10% ethyl acetate in cyclohexane) to yield malonate **10** (13.2 g, 29.3 mmol, 56%) as a slightly yellow oil.

TLC (30% ethyl acetate in cyclohexane): R_f = 0.50 (CAM).

^{1}H NMR (400 MHz, chloroform-*d*) δ 7.38 – 7.30 (m, 5H), 5.21 – 5.15 (m, 2H), 4.56 – 4.44 (m, 2H), 4.26 (t, *J* = 5.7 Hz, 1H), 4.07 – 3.96 (m, 2H), 3.71 (s, 6H), 3.35 (t, *J* = 7.5 Hz, 1H), 1.98 – 1.84 (m, 2H), 1.59 – 1.53 (m, 2H), 0.89 (s, 9H), 0.06 – 0.00 (m, 6H).

^{13}C NMR (101 MHz, chloroform-*d*) δ 169.9, 169.9, 147.4, 138.4, 128.5, 127.7, 127.6, 112.8, 73.4, 72.3, 70.1, 52.5, 52.5, 51.6, 34.0, 26.0, 25.9, 24.6, 18.3, –4.6, –5.0.

IR (Diamond-ATR, neat) $\tilde{\nu}_{max}$: 2953 (w), 2929 (w), 2856 (w), 1737 (s), 1435 (w), 1251 (s), 1073 (s), 835 (s), 775 (s), 698 (m) cm^{-1}.

HRMS (ESI) calc. for $C_{24}H_{38}NaO_6Si$ $[M+Na]^+$: 473.2330 found: 473.2297.

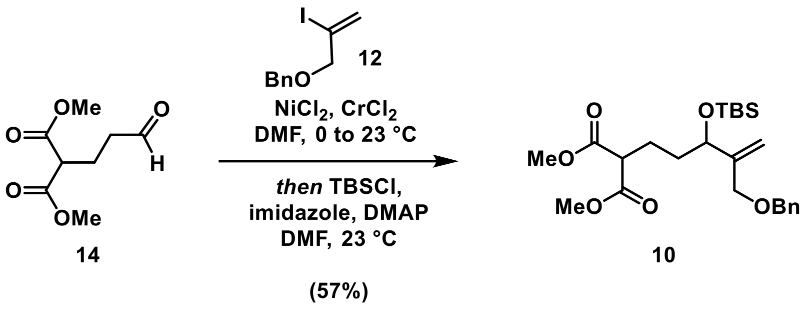

Malonate 10

Note: N,N-dimethylformamide was degassed via freeze-pump thaw (three cycles) prior to use. The NHK reaction was conducted in four parallel runs (each 26.6 mmol), which were combined after the work-up.

A solution of aldehyde **14** (5.00 g, 26.6 mmol, 1 equiv) and vinyl iodide **12** (10.9 g, 39.9 mmol, 1.50 equiv) in degassed N,N-dimethylformamide (30.0 mL) was added to a suspension of chromium dichloride (10.8 g, 87.7 mmol, 3.30 equiv) and nickel dichloride (344 mg, 2.66 mmol, 0.100 equiv) in degassed *N,N*-dimethylformamide (130 mL) at 0 °C, which was then warmed to 23 °C. After 6 h, *tert*-butyldimethylsilyl chloride (8.01 g, 53.2 mmol, 2.00 equiv), imidazole (7.24 g, 106 mmol, 4.00 equiv) and 4-dimethylaminopyridine (325 mg, 2.66 mmol, 0.100 equiv) were added to the reaction mixture at 23 °C. After 12 h, 1 M aqueous hydrochloric acid (250 mL) and diethyl ether (250 mL) were added to the reaction mixture. The layers were separated, and the aqueous phase was extracted with diethyl ether (2 × 250 mL). The combined organic phases were washed with 1 M aqueous lithium chloride solution (500 mL) and water (500 mL). The washed organic layers were dried over magnesium sulfate and the dried solution was filtered. The filtrate was concentrated under reduced pressure and the combined residues were purified by flash column chromatography on silica gel (10% diethyl ether in pentane) to give malonate **10** (27.1 g, 60.0 mmol, 57%) as a yellow oil.

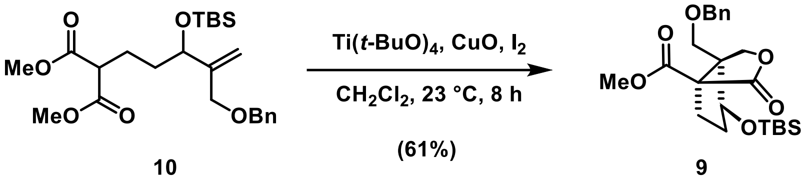

Ester 9

To a solution of malonate **10** (13.2 g, 29.3 mmol, 1 equiv) in dichloromethane (300 mL) was added titanium(IV) *tert*-butoxide (12.3 mL, 32.2 mmol, 1.10 equiv) at 23 °C. After 15 minutes, iodine (29.7 g, 117 mmol, 4.00 equiv) and cupric oxide (2.85 g, 35.1 mmol, 1.20 equiv) were added. After eight hours, a saturated aqueous solution of sodium thiosulfate (200 mL) was added to the red suspension and the layers were separated. The aqueous layer was extracted with dichloromethane (3 × 100 mL) and the combined organic layers were dried over sodium sulfate. The dried solution was filtered over a pad of Celite and the filtrate was concentrated. The residue was purified by flash column chromatography on silica gel (10% ethyl acetate in cyclohexane) to yield ester **9** (7.72 g, 17.8 mmol, 61%) as a slightly yellow oil.

TLC (30% ethyl acetate in cyclohexane): R_f = 0.50 (CAM).

^{1}H NMR (600 MHz, chloroform-*d*) δ 7.36 – 7.33 (m, 2H), 7.31 – 7.28 (m, 1H), 7.26 – 7.24 (m, 2H), 4.63 (d, *J* = 9.3 Hz, 1H), 4.45 – 4.39 (m, 2H), 4.22 (dd, *J* = 8.5, 5.7 Hz, 1H), 4.01 (d, *J* = 9.3 Hz, 1H), 3.59 (s, 3H), 3.48 (d, *J* = 9.3 Hz, 1H), 3.35 (d, *J* = 9.2 Hz, 1H), 2.39 (ddd, *J* = 13.4, 11.4, 6.9 Hz, 1H), 2.24 (ddd, *J* = 13.5, 6.9, 3.3 Hz, 1H), 1.99 (dddd, *J* = 12.7, 6.8, 5.7, 3.3 Hz, 1H), 1.49 (dddd, *J* = 12.7, 11.3, 8.5, 6.9 Hz, 1H), 0.86 (s, 9H), 0.05 – 0.00 (m, 6H).

^{13}C NMR (151 MHz, chloroform-*d*) δ 176.9, 169.2, 137.3, 128.4, 127.9, 127.6, 76.0, 73.5, 69.8, 68.7, 61.3, 58.0, 52.7, 32.7, 30.3, 25.6, 17.9, –4.5, –5.1.

IR (Diamond-ATR, neat) $\tilde{\upsilon}_{max}$: 2954 (w), 2929 (w), 2856 (w), 1778 (s), 1743 (m), 1252 (m), 1147 (m), 1036 (m), 838 (m), 778 (w) cm^{-1}.

HRMS (ESI) calc. for $C_{23}H_{34}KO_6Si$ $[M+K]^+$: 473.1756 found: 473.2299.

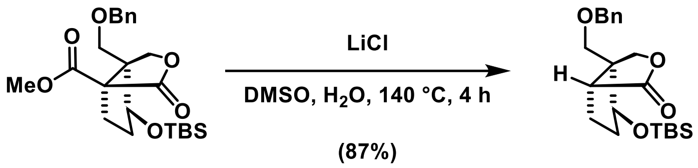

9 15

Lactone 15

To a solution of ester **9** (7.60 g, 17.5 mmol, 1 equiv) in dimethyl sulfoxide (70 mL) was added lithium chloride (5.99 g, 140 mmol, 8.00 equiv) and water (2.52 mL, 140 mmol, 8.00 equiv). The suspension was heated to 140 °C. After four hours, the reaction mixture was allowed to cool to 23 °C. When 23 °C was reached, water (70 mL) and diethyl ether (70 mL) were added to the reaction mixture and the layers were separated. The aqueous layer was extracted with diethyl ether (3 × 25 mL) and the combined organic layers were dried over sodium sulfate. The dried solution was filtered and the filtrate was concentrated. The residue was purified by flash column chromatography on silica gel (20% ethyl acetate in cyclohexane) to yield lactone **15** (5.72 g, 15.2 mmol, 87%) as a colourless oil.

TLC (20% ethyl acetate in cyclohexane): R_f = 0.26 (CAM).

^{1}H NMR (400 MHz, chloroform-*d*) δ 7.40 – 7.28 (m, 5H), 4.55 (d, *J* = 9.7 Hz, 1H), 4.53 (s, 2H), 4.15 (dd, *J* = 9.1, 5.3 Hz, 1H), 3.92 (d, *J* = 9.7 Hz, 1H), 3.42 – 3.37 (m, 2H), 2.88 – 2.84 (m, 1H), 2.07 – 1.99 (m, 1H), 1.96 – 1.85 (m, 2H), 1.64 – 1.57 (m, 1H), 0.86 (s, 9H), 0.05 – 0.01 (m, 6H).

^{13}C NMR (101 MHz, chloroform-*d*) δ 180.8, 137.8, 128.6, 128.0, 127.8, 76.1, 73.6, 72.3, 69.9, 54.3, 46.0, 33.7, 26.1, 25.8, 18.0, –4.4, –5.0.

IR (Diamond-ATR, neat) $\tilde{\upsilon}_{max}$: 2954 (w), 2929 (w), 2856 (w), 1770 (s), 1463 (w), 1362 (w), 1253 (m), 1147 (m), 1113 (s), 1028 (m), 866 (m), 837 (m), 777 (m), 698 (m) cm^{-1}.

HRMS (ESI) calc. for $C_{21}H_{32}NaO_4Si$ $[M+Na]^+$: 399.1962 found: 399.1934.

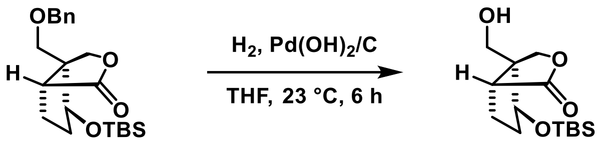

15 **16**

Alcohol 16

To a solution of lactone **15** (5.68 g, 15.1 mmol, 1 equiv) in tetrahydrofuran (16 mL) was added $Pd(OH)_2$/C (20 wt%, 5.30 g, 7.54 mmol, 0.500 equiv). The reaction vessel was placed in a high-pressure autoclave and exposed to hydrogen pressure of 40 bar. After six hours, the gas was released and the autoclave was purged with nitrogen for one minute. The reaction mixture was filtered through a pad of Celite and the pad washed with dichloromethane (20 mL). The filtrate was concentrated to yield alcohol **16** (4.43 g, 15.5 mmol) as a colourless oil. The crude reaction mixture was used in the next step without further purification. To obtain analytical data a small aliquot of the crude reaction mixture was purified by flash column chromatography on silica gel (50% ethyl acetate in cyclohexane).

TLC (50% ethyl acetate in cyclohexane): R_f = 0.29 (CAM).

^{1}H NMR (600 MHz, chloroform-*d*) δ 4.57 (d, *J* = 9.8 Hz, 1H), 4.14 (dd, *J* = 9.1, 5.3 Hz, 1H), 4.00 (d, *J* = 9.8 Hz, 1H), 3.69 – 3.62 (m, 2H), 2.85 – 2.80 (m, 1H), 2.07 – 2.02 (m, 1H), 1.96 – 1.90 (m, 2H), 1.78 (t, *J* = 4.6 Hz, 1H), 1.63 – 1.58 (m, 1H), 0.87 (s, 9H), 0.08 – 0.05 (m, 6H).

^{13}C NMR (151 MHz, chloroform-*d*) δ 180.8, 76.3, 69.7, 65.3, 55.0, 45.4, 33.6, 26.1, 25.8, –4.3, –5.0.

IR (Diamond-ATR, neat) $\tilde{\upsilon}_{max}$: 3478 (br, w), 2955 (m), 2930 (m), 2858 (m), 1752 (s), 1471 (w), 1254 (m), 1197 (m), 1149 (m), 1112 (m), 1031 (m), 865 (s), 838 (s), 778 (m) cm^{-1}.

HRMS (ESI) calc. for $C_{14}H_{26}NaO_4Si$ $[M+Na]^+$: 309.1493 found: 309.1469.

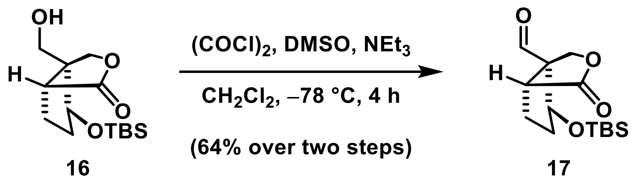

Aldehyde 17

To a solution of oxalylchloride (1.65 mL, 17.0 mmol, 1.10 equiv) in dichloromethane (100 mL) was added dimethyl sulfoxide (2.42 mL, 34.0 mmol, 2.20 equiv) at −78 °C. After ten minutes, a solution of crude alcohol **16** (4.43 g, 15.5 mmol, 1 equiv) in dichloromethane (40 mL) was added dropwise to the reaction mixture over the course of five minutes. Ten minutes after the addition of the alcohol **17** was completed, triethylamine (10.7 mL, 77.3 mmol, 5.00 equiv) was added to the reaction mixture. After 20 minutes, the reaction mixture was allowed to warm to 23 °C. After four hours at 23 °C, water (150 mL) was added to the reaction mixture and the layers were separated. The aqueous layer was extracted with dichloromethane (3 × 50 mL) and the combined organic layers were dried over sodium sulfate. The dried solution was filtered and the filtrate was concentrated. The residue was purified by flash column chromatography on silica gel (30% ethyl acetate in cyclohexane) to yield aldehyde **17** (2.80 g, 9.84 mmol, 64% over two steps) as a colourless oil.

TLC (30% ethyl acetate in cyclohexane): R_f = 0.22 (CAM).

^{1}H NMR (600 MHz, chloroform-*d*) δ 9.67 (s, 1H), 4.63 (dd, *J* = 10.2, 1.1 Hz, 1H), 4.57 – 4.53 (m, 2H), 3.11 (dt, *J* = 8.6, 1.9 Hz, 1H), 2.19 – 2.15 (m, 1H), 2.04 – 1.98 (m, 2H), 1.73 – 1.66 (m, 1H), 0.86 (s, 9H), 0.08 – 0.03 (m, 6H).

^{13}C NMR (151 MHz, chloroform-*d*) δ 198.6, 178.2, 75.7, 65.6, 64.6, 45.5, 34.1, 26.5, 25.7, 18.0, –4.5, –5.0.

IR (Diamond-ATR, neat) $\tilde{\upsilon}_{max}$: 3010 (br, w), 2955 (m), 2931 (m), 2887 (w), 2858 (m), 1777 (s), 1745 (s), 1471 (w), 1387 (w), 1254 (m), 1198 (m), 1129 (m), 1028 (m), 861 (s), 839 (s), 779 (m) cm^{-1}.

HRMS (ESI) calc. for $C_{14}H_{24}NaO_4Si$ $[M+Na]^+$: 307.1336 found: 307.1314.

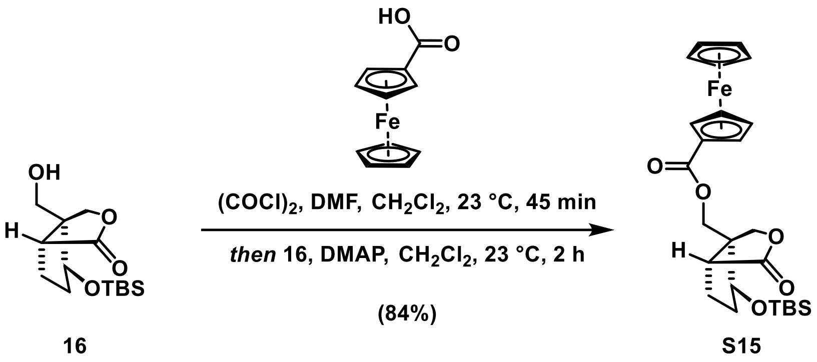

Ferrocene ester S15

To suspension of ferrocene carboxylic acid (8.25 mg, 35.0 µmol, 2.00 equiv) in dichloromethane (800 µL) was added a solution of oxalyl chloride (2.00 M, 19.1 µL, 38.0 µmol, 2.20 equiv) followed by a microsyringe drop of *N*,*N*-dimethylformamide at 23 °C upon which gas formation was observed. The reaction mixture turned red. After 45 minutes, toluene (800 µL) was added and the mixture was concentrated. To a solution of alcohol **16** (4.98 mg, 17.4 µmol, 1 equiv) and 4-dimethylaminopyridine (21.5 mg, 174 µmol, 10.0 equiv) in dichloromethane (300 µL) was added a solution of the freshly prepared ferrocene acid chloride in dichloromethane (300 µL) at 23 °C. After 2 hours, the reaction mixture was concentrated and the residue was purified by flash column chromatography on silica gel (10% ethyl acetate in cyclohexane) to yield ferrocene ester **S15** (7.30 mg, 15.0 µmol, 84%) as a yellow solid.

*Note: Crystals of suitable quality and size for X-ray crystallography were obtained by slowly evaporating a solution of ferrocene ester **S15** in diethyl ether at room temperature.*

TLC (10% ethyl acetate in cyclohexane): R_f = 0.20 (CAM, UV).

^{1}H NMR (400 MHz, chloroform-*d*) δ 4.79 – 4.76 (m, 2H), 4.66 (d, *J* = 9.9 Hz, 1H), 4.45 – 4.42 (m, 2H), 4.28 (d, *J* = 11.1 Hz, 1H), 4.22 – 4.19 (m, 5H), 4.16 – 4.11 (m, 2H), 4.08 (d, *J* = 9.9 Hz, 1H), 2.91 – 2.86 (m, 1H), 2.18 – 2.09 (m, 1H), 2.06 – 1.96 (m, 2H), 1.76 – 1.64 (m, 1H), 0.90 (s, 9H), 0.11 – 0.07 (m, 6H).

^{13}C NMR (101 MHz, chloroform-*d*) δ 180.1, 171.8, 76.6, 71.9, 70.3, 70.2, 70.2, 70.0, 69.8, 66.6, 53.7, 46.1, 33.6, 26.0, 25.8, 18.0, –4.2, –4.9.

IR (Diamond-ATR, neat) $\tilde{\nu}_{max}$: 2955 (w), 2857 (w), 1773 (s), 1717 (s), 1460 (w), 1385 (s), 1273 (s), 1212 (w), 1132 (s), 1028 (m), 1002 (w), 864 (m), 838 (m), 776 (m) cm^{-1}.

HRMS (ESI) calc. for $C_{25}H_{34}FeNaO_5Si$ [M+Na]$^+$: 521.1417 found: 521.1394.

Crystal structure: *see chapter 4 for more details*

Screening of Aldehyde Protection

Table 1: Screening of the acetalization conditions to furnish aldehyde **18**.

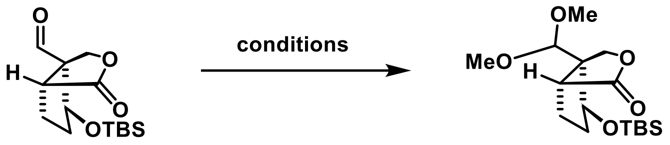

17 **18**

entry	conditions	result
1	*p*-TsOH, MeOH	decomposition
2	$TiCl_4$, MeOH, NEt_3	no reaction
3	NH_4NO_3, MeOH	no reaction
4[9]	Eosin Y, MeCN/ MeOH<br>green LEDs	no reaction
5[10]	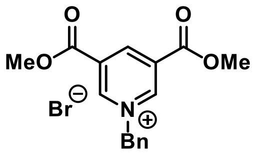 + MeOH	no reaction
6	Dowex 50WX4, $HC(OMe)_3$	70%

[9] H. Yi, L. Niu, S. Wang, T. Liu, A. K. Singh, A. Lei, *Org. Lett.* **2017**, *19*, 122–125.
[10] B. Procuranti, S. J. Connon, *Org. Lett.* **2008**, *10*, 4935–4938.

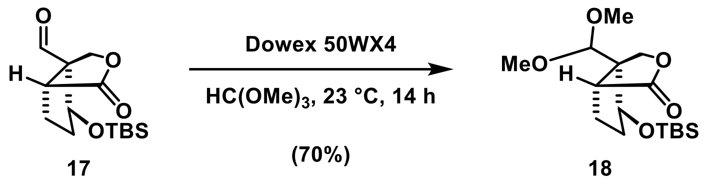

Acetal 18

To a solution of aldehyde **17** (2.80 g. 9.84 mmol, 1 equiv) in trimethyl orthoformate (18 mL) was added Dowex 50WX4 (4.20 g) at 23 °C. After 14 hours, the reaction mixture was filtered through a pad of Celite and the pad was washed with ethyl acetate (20 mL). The filtrate was concentrated and the residue was purified by flash column chromatography on silica gel (20% ethyl acetate in cyclohexane) to yield acetal **18** (2.26 g, 6.84 mmol, 70%) as a colourless oil.

TLC (20% ethyl acetate in cyclohexane): R_f = 0.26 (CAM).

^{1}H NMR (600 MHz, chloroform-*d*) δ 4.53 (d, *J* = 9.6 Hz, 1H), 4.27 (dd, *J* = 9.1, 5.7 Hz, 1H), 4.20 (s, 1H), 4.09 (d, *J* = 9.7 Hz, 1H), 3.52 – 3.49 (m, 6H), 2.92 (dd, *J* = 9.6, 1.8 Hz, 1H), 2.02 – 1.98 (m, 1H), 1.94 – 1.90 (m, 1H), 1.89 – 1.82 (m, 1H), 1.52 (tdd, *J* = 11.1, 6.6, 4.0 Hz, 1H), 0.87 (s, 9H), 0.06 – 0.04 (m, 6H).

^{13}C NMR (151 MHz, chloroform-*d*) δ 180.8, 108.3, 75.9, 68.8, 58.8, 58.2, 44.8, 34.0, 26.3, 25.8, 18.0, –4.3, –5.0.

IR (Diamond-ATR, neat) $\tilde{\upsilon}_{max}$: 2954 (m), 2930 (m), 2857 (m), 1770 (s), 1471 (w), 1385 (w), 1253 (m), 1195 (m), 1150 (m), 1073 (s), 1029 (m), 867 (m), 837 (s), 777 (m), 673 (w) cm^{-1}.

HRMS (ESI) calc. for $C_{16}H_{30}NaO_5Si$ $[M+Na]^+$: 353.1755 found: 353.1753.

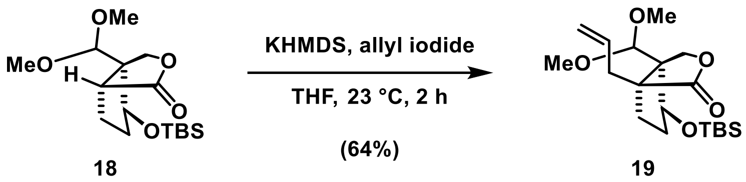

Lactone 19

To a solution of acetal **18** (2.26 g, 6.84 mmol, 1 equiv) and allyl iodide (2.60 mL, 27.9 mmol, 4.00 equiv) in tetrahydrofuran (70 mL) was added a potassium bis(trimethylsilyl)amide solution (1.00 M in tetrahydrofuran, 13.7 mL, 13.7 mmol, 2.00 equiv) at 23 °C. After two hours, water (70 mL) was added to the reaction mixture and the layers were separated. The aqueous layer was extracted with diethyl ether (3 × 20 mL) and the combined organic layers were dried over sodium sulfate. The dried solution was filtered and the filtrate was concentrated. The residue was purified by flash column chromatography on silica gel (10% ethyl acetate in cyclohexane) to yield lactone **19** (1.63 g, 4.40 mmol, 64%) as a colourless oil.

TLC (30% ethyl acetate in cyclohexane): R_f = 0.49 (CAM).

^{1}H NMR (400 MHz, chloroform-*d*) δ 5.89 (ddt, *J* = 17.4, 10.4, 7.3 Hz, 1H), 5.18 – 5.10 (m, 2H), 4.61 (d, *J* = 9.2 Hz, 1H), 4.55 (dd, *J* = 8.2, 6.7 Hz, 1H), 4.47 (s, 1H), 3.84 (d, *J* = 9.2 Hz, 1H), 3.52 – 3.47 (m, 6H), 2.47 (dd, *J* = 7.2, 1.5 Hz, 2H), 2.07 – 1.96 (m, 2H), 1.61 – 1.55 (m, 1H), 1.35 – 1.28 (m, 1H), 0.87 (s, 9H), 0.06 – 0.03 (m, 6H).

^{13}C NMR (151 MHz, chloroform-*d*) δ 181.9, 134.1, 118.6, 108.5, 74.7, 68.0, 59.3, 59.0, 57.4, 56.2, 37.4, 34.0, 33.5, 25.9, 18.1, –4.4, –5.0.

IR (Diamond-ATR, neat) $\tilde{\upsilon}_{max}$: 2954 (m), 2930 (m), 2856 (m), 1768 (s), 1471 (w), 1362 (w), 1251 (m), 1149 (s), 1107 (s), 1070 (s), 1027 (s), 866 (m), 837 (s), 777 (m) cm^{-1}.

HRMS (ESI) calc. for $C_{19}H_{34}NaO_5Si$ $[M+Na]^+$: 393.2068 found: 393.2060.

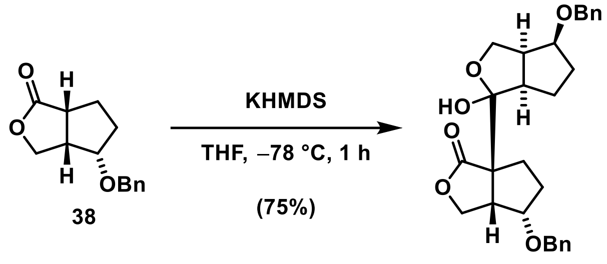

Lactone dimer 39

To a solution of potassium bis(trimethylsilyl)amide (1.00 M in tetrahydrofuran, 38.7 µL, 39.0 µmol, 1.50 equiv) was added a solution of lactone **38** (6.00 mg, 26.0 µmol, 1 equiv) in tetrahydrofuran (400 µL) at –78 °C. After one hour, water (5 mL) and ethyl acetate (5 mL) were added to the reaction mixture. The layers were separated, the aqueous layer was extracted with ethyl acetate (3 × 2 mL) and the combined organic layers were dried over sodium sulfate. The dried solution was filtered and the filtrate was concentrated. The residue was purified by flash column chromatography on silica gel (20% grading to 30% ethyl acetate in cyclohexane) to yield lactone dimer **39** (9.00 mg, 19.0 µmol, 75%) as a colourless oil.

TLC (20% ethyl acetate in cyclohexane): R_f = 0.52 (CAM).

^{1}H NMR (400 MHz, chloroform-*d*) δ 7.37 – 7.28 (m, 10H), 5.22 (s, 1H), 4.71 (d, *J* = 11.9 Hz, 1H), 4.53 – 4.45 (m, 3H), 4.41 (d, *J* = 11.9 Hz, 1H), 4.23 (t, *J* = 8.8 Hz, 1H), 4.06 – 3.99 (m, 2H), 3.85 (d, *J* = 8.9 Hz, 1H), 3.82 – 3.79 (m, 1H), 3.75 – 3.68 (m, 1H), 3.46 – 3.41 (m, 1H), 2.88 – 2.81 (m, 1H), 2.29 – 2.23 (m, 1H), 2.00 – 1.95 (m, 1H), 1.93 – 1.89 (m, 1H), 1.86 – 1.82 (m, 1H), 1.79 – 1.73 (m, 1H), 1.70 – 1.63 (m, 1H), 1.62 – 1.56 (m, 1H).

^{13}C NMR (201 MHz, chloroform-*d*) δ 180.3, 138.3, 137.1, 128.8, 128.6, 128.3, 128.1, 127.8, 127.5, 105.3, 81.2, 79.7, 71.6, 71.0, 65.9, 64.6, 61.1, 50.1, 47.2, 43.4, 34.4, 30.9, 29.1, 23.4.

IR (Diamond-ATR, neat) $\tilde{\upsilon}_{max}$: 2926 (m), 2855 (w), 1737 (s), 1459 (w), 1370 (m), 1218 (s), 1049 (m), 605 (w) cm^{-1}.

HRMS (ESI) calc. for $C_{28}H_{36}NO_6$ $[M+NH_4]^+$: 482.2537 found: 482.2541.

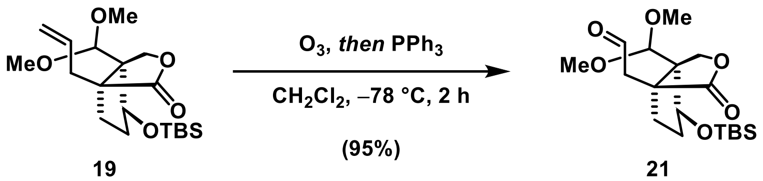

19 21

Aldehyde 21

Through a solution of lactone **19** (1.63 g, 4.40 mmol, 1 equiv) in dichloromethane (44 mL) was sparged a stream of ozone at −78 °C. After ten minutes, the reaction mixture turned blue and then oxygen was sparged through the blue reaction mixture until the colour disappeared. Triphenylphosphine (3.50 g, 13.2 mmol, 3.00 equiv) was added to the reaction mixture. The reaction mixture was allowed to warm to 23° C. After two hours at 23 °C, the mixture was concentrated and the residue was purified by flash column chromatography on silica gel (30% ethyl acetate in cyclohexane) to yield aldehyde **21** (1.55 g, 4.16 mmol, 95%) as a colourless solid.

TLC (40% ethyl acetate in cyclohexane): R_f = 0.35 (CAM).

mp: (63-64) °C

^{1}H NMR (600 MHz, chloroform-*d*) δ 9.67 (dd, *J* = 1.9, 0.9 Hz, 1H), 4.71 (d, *J* = 9.2 Hz, 1H), 4.48 (dd, *J* = 9.8, 6.6 Hz, 1H), 4.17 (s, 1H), 4.09 (d, *J* = 9.2 Hz, 1H), 3.47 – 3.44 (m, 6H), 2.97 (dd, *J* = 18.2, 1.9 Hz, 1H), 2.86 (dd, *J* = 18.1, 1.0 Hz, 1H), 2.08 (ddd, *J* = 13.1, 7.2, 1.8 Hz, 1H), 1.98 – 1.93 (m, 1H), 1.69 (td, *J* = 12.9, 6.6 Hz, 1H), 1.46 – 1.40 (m, 1H), 0.89 (s, 9H), 0.06 (s, 6H).

^{13}C NMR (151 MHz, chloroform-*d*) δ 200.2, 181.8, 108.6, 75.3, 68.4, 59.1, 58.8, 57.9, 51.8, 47.6, 34.7, 32.6, 25.9, 18.1, –4.2, –4.9.

IR (Diamond-ATR, neat) $\tilde{\upsilon}_{max}$: 2955 (m), 2930 (m), 2957 (m), 1766 (s), 1724 (m), 1471 (w), 1388 (w), 1253 (m), 1188 (m), 1152 (m), 1114 (s), 1071 (s), 1028 (s), 870 (m), 837 (m), 777 (m), 670 (w) cm^{-1}.

HRMS (ESI) calc. for $C_{18}H_{32}NaO_6Si$ $[M+Na]^+$: 395.1860 found: 395.1852.

2.3. Total Syntheses of Applanatumol E, I, Lingzhilactone B and Meroapplanin B

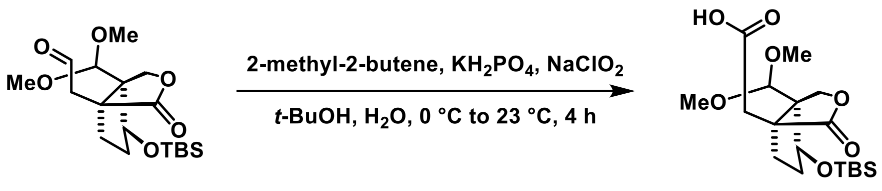

Acid 8

To a solution of aldehyde **21** (209 mg, 561 µmol, 1 equiv) and potassium dihydrogen phosphate (153 mg, 1.12 mmol, 2.00 equiv) in *tert*-butanol (3 mL), 2-methyl-2-butene (1 mL) and water (1 mL) was added sodium chlorite (190 mg, 1.68 mmol, 3.00 equiv) at 0 °C. After four hours, saturated aqueous solution of ammonium chloride (10 mL) and ethyl acetate (5 mL) were added to the reaction mixture and the layers were separated. The aqueous layer was extracted with ethyl acetate (3 × 5 mL) and the combined organic layers were dried over sodium sulfate. The dried solution was filtered and the filtrate was concentrated to yield acid **8** (271 mg) as a colourless solid. The crude reaction mixture was used in the next step without further purification. To obtain analytical data a small aliquot of the crude reaction mixture was purified by flash column chromatography on silica gel (5% methanol in dichloromethane).

TLC (5% methanol in dichloromethane): R_f = 0.18 (CAM).

mp: (89-91) °C

^{1}H NMR (600 MHz, chloroform-*d*) δ 4.76 (d, *J* = 8.9 Hz, 1H), 4.51 (dd, *J* = 9.8, 6.7 Hz, 1H), 4.26 (s, 1H), 4.07 (d, *J* = 9.0 Hz, 1H), 3.49 – 3.47 (m, 6H), 2.91 (q, *J* = 17.7 Hz, 2H), 2.03 – 1.98 (m, 1H), 1.96 – 1.91 (m, 1H), 1.65 – 1.61 (m, 1H), 1.36 (ddt, *J* = 12.5, 5.7, 2.8 Hz, 1H), 0.88 (s, 9H), 0.06 (s, 6H).

^{13}C NMR (151 MHz, chloroform-*d*) δ 182.6, 176.4, 108.5, 75.1, 68.6, 59.1, 58.3, 57.7, 52.9, 38.3, 34.7, 32.7, 25.9, 18.1, –4.3, –5.0.

IR (Diamond-ATR, neat) $\tilde{\nu}_{max}$: 3011 (br, w), 2955 (m), 2929 (m), 2856 (m), 1767 (m), 1739 (s), 1390 (m), 1362 (m), 1253 (m), 1186 (m), 1153 (s), 1135 (s), 1116 (s), 1070 (s), 1026 (s), 906 (m), 865 (m), 837 (s), 776 (m), 724 (w) cm^{-1}.

HRMS (ESI) calc. for $C_{18}H_{32}NaO_7Si$ $[M+Na]^+$: 411.1810 found: 411.1817, calc. for $C_{18}H_{31}O_7Si$ $[M–H]^-$: 387.1845 found: 387.1842.

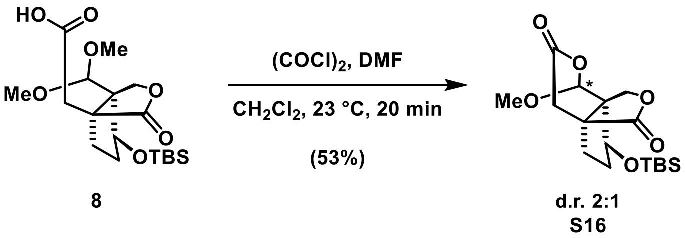

Propellane S16

To a solution of acid **8** (4.50 mg, 12.0 µmol, 1 equiv) in dichloromethane (300 µL) was added a solution of oxalyl chloride (2.00 M in dichloromethane, 23.0 µL, 46.0 µmol, 4.00 equiv) and a microsyringe drop of *N*,*N*-dimethylformamide at 23 °C. After 20 minutes, the reaction mixture was concentrated and the residue was purified by flash column chromatography on silica gel (30% ethyl acetate in cyclohexane) to yield tricyclic lactone **S16** (2:1 ratio of diastereomers, 2.20 mg, 6.00 µmol, 53%) as a colourless oil.

TLC (30% ethyl acetate in cyclohexane): R_f = 0.30 (double spot) (CAM, UV).

Product was obtained in a diastereomeric ratio of 2:1. The ^{1}H and ^{13}C NMR signals are shown for the major diastereomer.

^{1}H NMR (600 MHz, chloroform-*d*) δ 5.05 (d, *J* = 5.0 Hz, 1H), 4.57 (d, *J* = 10.1 Hz, 1H), 4.31 (d, *J* = 10.1 Hz, 1H), 4.23 – 4.18 (m, 1H), 3.57 (s, 3H), 2.90 (d, *J* = 15.7 Hz, 1H), 2.69 (d, *J* = 15.7 Hz, 1H), 2.29 – 2.26 (m, 1H), 1.95 – 1.91 (m, 1H), 1.62 – 1.58 (m, 2H), 0.88 (s, 9H), 0.08 (s, 6H).

^{13}C NMR (151 MHz, chloroform-*d*) δ 179.9, 168.4, 103.9, 76.3, 67.5, 57.8, 55.6, 48.8, 36.7, 34.1, 31.8, 25.7, 18.0, –4.3, –4.9.

IR (Diamond-ATR, neat) $\tilde{\upsilon}_{max}$: 2957 (w), 2929 (w), 2856 (w), 1782 (s), 1748 (w), 1463 (w), 1397 (w), 1256 (m), 1206 (m), 1166 (m), 1142 (m), 1071 (m), 1047 (m), 1021 (m), 988 (m), 902 (w), 838 (m), 778 (m) cm^{-1}.

HRMS (ESI) calc. for $C_{17}H_{28}NaO_6Si$ $[M+Na]^+$: 379.1547 found: 379.1522.

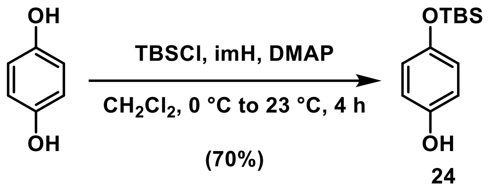

24

Phenol 24

To a solution of hydroquinone (581 mg, 5.22 mmol, 1 equiv) in dichloromethane (52 mL) was added imidazole (898 mg, 13.1 mmol, 2.50 equiv) and *tert*-butyldimethylsilyl chloride (956 mg, 6.22 mmol, 1.20 equiv) at 23 °C. After four hours, the solution was concentrated and the residue was purified by flash column chromatography on silica gel (10% ethyl acetate in cyclohexane) to yield phenol **24** (821 mg, 3.66 mmol, 70%) as a slightly yellow oil. The obtained analytical data were in full agreement with those reported in the literature.[11]

[11] W. Li, Y. Gao, Q. Li, Z.-J. Li, *Org. Biomol. Chem.* **2018**, *16*, 4720–4727.

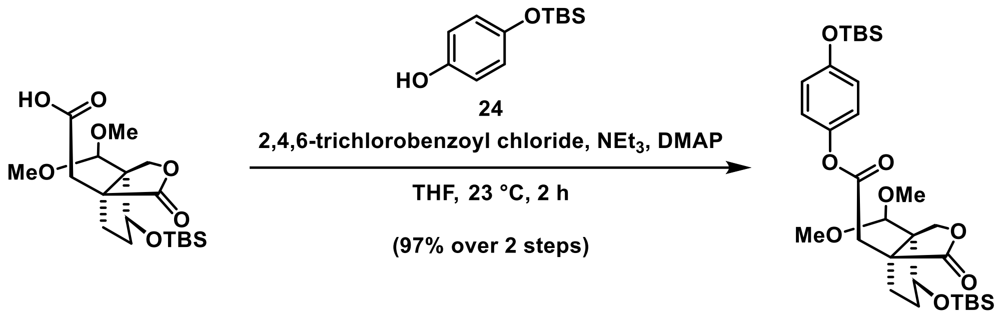

8 7

Ester 7

To a solution of acid **8** (271 mg, 698 µmol, assumed pure) and phenol **24** (203 mg, 907 µmol, 1.30 equiv) in tetrahydrofuran (7 mL) was added triethylamine (776 µl, 5.58 mmol, 8.00 equiv) followed by 2,4,6-trichlorobenzoyl chloride (556 µl, 3.49 mmol, 5.00 equiv) at 23 °C. After 15 minutes, 4-(dimethylamino)pyridine (8.61 mg, 70.0 µmol, 0.100 equiv) was added. After two hours, the mixture was concentrated and the residue was purified by flash column chromatography on silica gel (5% ethyl acetate in cyclohexane) to yield ester **7** (325 mg, 546 µmol, 97% over 2 steps) as a colourless solid.

TLC (10% ethyl acetate in cyclohexane): R_f = 0.37 (CAM, UV).

mp: (95-96) °C

^{1}H NMR (600 MHz, chloroform-*d*) δ 6.96 – 6.93 (m, 2H), 6.81 – 6.78 (m, 2H), 4.76 (d, *J* = 8.9 Hz, 1H), 4.55 (dd, *J* = 9.8, 6.7 Hz, 1H), 4.34 (s, 1H), 4.10 (d, *J* = 9.0 Hz, 1H), 3.51 – 3.48 (m, 6H), 3.11 – 3.03 (m, 2H), 2.06 (ddd, *J* = 12.7, 7.0, 1.7 Hz, 1H), 1.95 (ddd, *J* = 11.3, 7.4, 5.7 Hz, 1H), 1.70 (td, *J* = 12.9, 6.5 Hz, 1H), 1.41 – 1.35 (m, 1H), 0.97 (s, 9H), 0.88 (s, 9H), 0.18 (s, 6H), 0.06 (s, 6H).

^{13}C NMR (151 MHz, chloroform-*d*) δ 181.8, 170.5, 153.5, 144.5, 122.1, 120.6, 108.8, 75.2, 68.1, 59.2, 58.2, 57.7, 53.2, 38.4, 34.9, 32.8, 25.9, 25.8, 18.3, 18.1, –4.3, –4.4, –5.0.

IR (Diamond-ATR, neat) $\tilde{\upsilon}_{max}$: 2930 (w), 2857 (w), 1765 (m), 1502 (s), 1471 (w), 1254 (m), 1188 (m), 1145 (m), 1069 (m), 1027 (m), 906 (s), 835 (s), 776 (s), 733 (w), 670 (w), 526 (w) cm^{-1}.

HRMS (ESI) calc. for $C_{30}H_{50}NaO_8Si_2$ $[M+Na]^+$: 617.2936 found: 617.2958, calc. for $C_{30}H_{50}KO_8Si_2$ $[M+K]^+$: 633.2676 found: 633.2669.

UV-VIS Spectrum of Photo-Fries Precursor 7

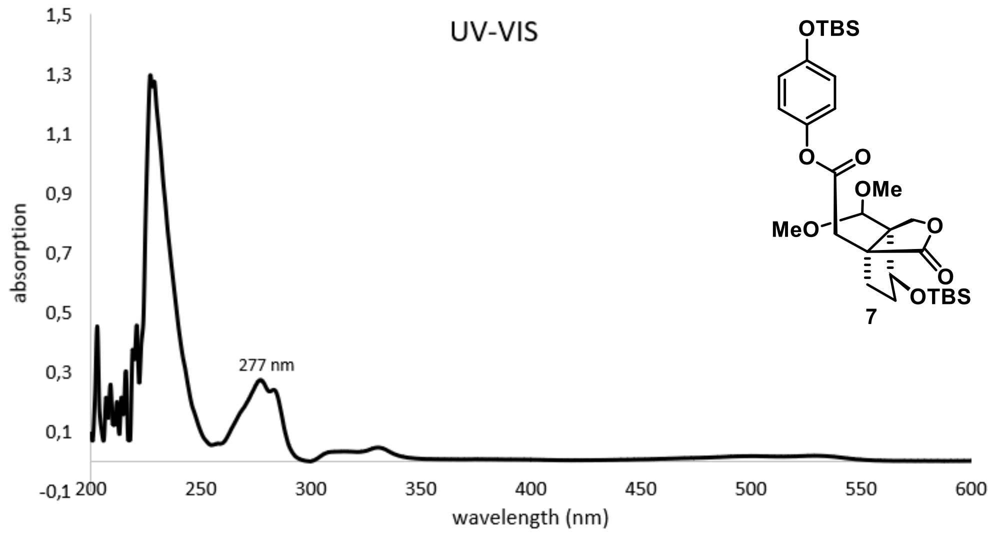

Screening of Photo-Fries rearrangement

Table 2: Optimization of the photo-Fries rearrangement.

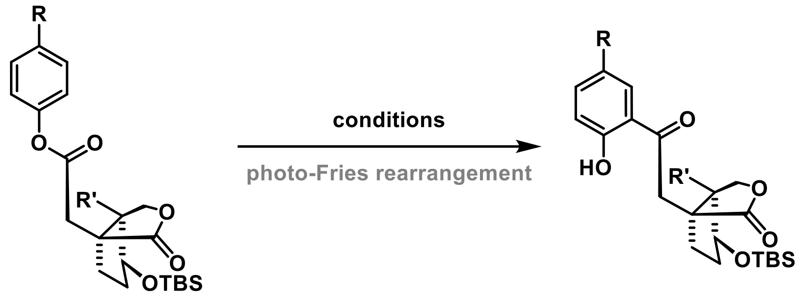

entry	**R =**	**R' =**	conditions	result
1	**H**	**CH_2OBn**	methanol, 254 nm (Rayonet)	decomposition
2	**OH**	**CH_2OBn**	methanol, 254 nm (Rayonet)	decomposition
3	**OBn**	**CH_2OBn**	acetonitrile, 254 nm (Rayonet)	decomposition + isolation of 4-benzyloxyphenol
4	**OMe**	**CH_2OBn**	methanol, 254 nm (Rayonet)	36% + isolation of 4-methoxyphenol
5	**OMe**	**CH_2OBn**	methanol, 300 nm (Rayonet)	31%
6	**OMe**	**CH_2OBn**	*n*-hexane, 50 °C (Xenon lamp)	very slow conversion
7	**OTBS**	**CH_2OBn**	*n*-hexane, 254 nm (Rayonet)	**27**, 48%
8	**OTBS**	**$CH(OMe)_2$**	methanol, 254 nm (Rayonet)	**26**, 42% + isolation of 4-(*tert*-butyldimethylsiloxy)phenol
9	**OTBS**	**$CH(OMe)_2$**	*n*-hexane, 254 nm (Rayonet)	**26**, 50%
10	**OMe**	**$CH(OMe)_2$**	*n*-hexane, 254 nm (Rayonet)	**32**, 49%

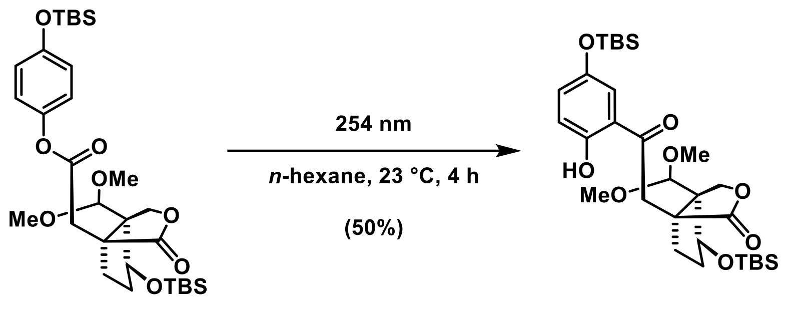

Ketone 26

Reaction setup: The reaction was carried out in the Rayonet RPR-200 Photochemical Reactor, with the reaction mixture placed in the center of the 25 cm wide reaction chamber (approximately 12 cm away from the light source), above a cooling fan and surrounded by a circular array of 16 light tubes.

A solution of ester **7** (60.0 mg, 101 µmol, 1 equiv) in degassed hexane (6 ml) was irradiated at 254 nm (Southern New England Ultraviolet Company, RPR-2537A° lamps) in a quartz tube. *Note: The reaction was set up in three parallel batches (20.0 mg and 2 ml n-hexane each) and irradiated at the same time.* After four hours, the reaction mixture was concentrated and the residue was purified by flash column chromatography on silica gel (5% ethyl acetate in cyclohexane) to yield ketone **26** (30.2 mg, 51.0 µmol, 50%) as a slightly yellow solid.

TLC (20% ethyl acetate in cyclohexane): R_f = 0.49 (CAM, UV).

mp: (160-161) °C

^{1}H NMR (600 MHz, chloroform-*d*) δ 11.51 (s, 1H), 7.13 (d, *J* = 2.9 Hz, 1H), 7.02 (dd, *J* = 8.9, 2.9 Hz, 1H), 6.87 (d, *J* = 8.9 Hz, 1H), 4.87 (d, *J* = 8.8 Hz, 1H), 4.55 (dd, *J* = 10.1, 6.6 Hz, 1H), 4.19 (d, *J* = 8.8 Hz, 1H), 4.11 (s, 1H), 3.69 (d, *J* = 18.6 Hz, 1H), 3.42 (d, *J* = 18.6 Hz, 1H), 3.38 (s, 3H), 3.25 (s, 3H), 2.07 – 2.03 (m, 1H), 1.94 (ddd, *J* = 12.6, 6.3, 4.7 Hz, 1H), 1.71 (dt, *J* = 12.8, 6.4 Hz, 1H), 1.42 (ddd, *J* = 13.0, 6.4, 3.7 Hz, 1H), 0.99 (s, 9H), 0.90 (s, 9H), 0.20 – 0.18 (m, 6H), 0.07 – 0.06 (m, 6H).

^{13}C NMR (151 MHz, chloroform-*d*) δ 203.6, 182.5, 157.0, 147.4, 129.7, 119.3, 118.9, 118.5, 108.7, 75.0, 68.1, 59.3, 57.6, 57.2, 52.5, 43.3, 35.1, 32.5, 25.8, 25.7, 18.2, 18.0, –4.4, –4.4, –4.5, –5.1.

IR (Diamond-ATR, neat) $\tilde{\nu}_{max}$: 2928 (m), 2857 (w), 1769 (s), 1640 (w), 1481 (m), 1388 (m), 1278 (m), 1174 (m), 1149 (m), 1115 (m), 1077 (m), 1003 (m), 947 (m), 886 (m), 836 (s), 783 (m), 642 (w) cm^{-1}.

HRMS (ESI) calc. for $C_{30}H_{50}NaO_8Si_2$ $[M+Na]^+$: 617.2936 found: 617.2958, calc. for $C_{30}H_{50}KO_8Si_2$ $[M+K]^+$: 633.2676 found: 633.2669.

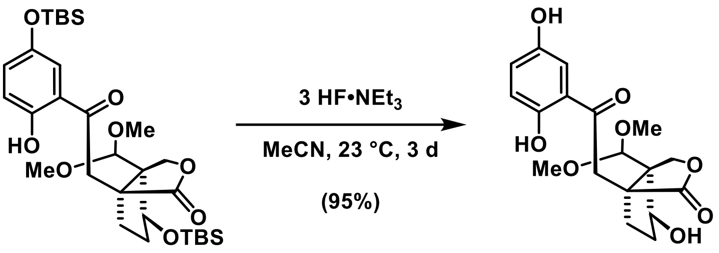

26 **applanatumol E (2)**

Applanatumol E (2)

To a solution of ketone **26** (57.4 mg, 97.0 µmol, 1 equiv) in acetonitrile (650 µL) was added hydrogen fluoride triethylamine (802 µL, 4.82 mmol, 50.0 equiv) at 23 °C. After three days, a saturated aqueous solution of sodium bicarbonate (5 mL) and ethyl acetate (5 mL) were added to the reaction mixture and the layers were separated. The aqueous layer was extracted with ethyl acetate (3 × 5 mL), the combined organic layers were dried over sodium sulfate. The dried solution was filtered and the filtrate was concentrated. The residue was purified by flash column chromatography on silica gel (5% methanol in dichloromethane) to yield applanatumol E (**2**) (33.4 mg, 91.2 µmol, 95%) as a yellow solid.

TLC (5% methanol in dichloromethane): R_f = 0.33 (CAM, UV).

mp: (130-133) °C

^{1}H NMR (600 MHz, acetone-d_6) δ 11.30 (s, 1H), 8.21 (s, 1H), 7.38 (d, J = 2.9 Hz, 1H), 7.12 (dd, J = 8.9, 3.0 Hz, 1H), 6.84 (d, J = 8.9 Hz, 1H), 4.81 (d, J = 8.8 Hz, 1H), 4.59 (t, J = 8.2 Hz, 1H), 4.39 (s, 1H), 4.22 (d, J = 8.8 Hz, 1H), 3.84 (d, J = 19.0 Hz, 1H), 3.68 (d, J = 3.9 Hz, 1H), 3.48 (d, J = 19.0 Hz, 1H), 3.43 (s, 3H), 3.34 (s, 3H), 1.97 – 1.88 (m, 2H), 1.79 – 1.71 (m, 1H), 1.31 (dddd, J = 14.0, 12.2, 10.9, 6.6 Hz, 1H).

^{13}C NMR (151 MHz, acetone-d_6) δ 205.2, 182.4, 156.5, 150.3, 126.2, 119.7, 119.6, 115.3, 110.2, 75.4, 68.0, 59.4, 57.8, 57.4, 53.5, 44.3, 35.7, 31.6.

IR (Diamond-ATR, neat) $\tilde{\upsilon}_{max}$: 3389 (br, w), 2963 (w), 2834 (w), 1743 (m), 1662 (m), 1485 (m), 1280 (m), 1235 (m), 1177 (s), 1093 (m), 1066 (s), 1027 (m), 785 (m), 647 (m) cm^{-1}.

HRMS (ESI) calc. for $C_{18}H_{22}NaO_8$ [M+Na]$^+$: 389.1207 found: 389.1206.

Table 3: Comparison of ^{1}H-NMR shifts for natural[12] and synthetic applanatumol E (**2**).

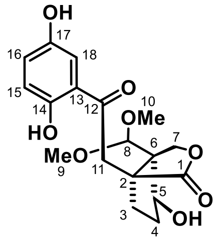

applanatumol E (2)

No	^{1}H-NMR (400 MHz, acetone-d_6) isolated applanatumol E **ppm**	^{1}H-NMR (600 MHz, acetone-d_6) synthetic applanatumol E **ppm**	Δ ppm
1	-	-	-
2	-	-	-
3	1.89 (overlap)	1.97 – 1.88 (m, overlap)	+0.04
	1.72 (m)	1.79 – 1.71 (m)	+0.03
4	1.89 (overlap)	1.97 – 1.88 (m, overlap)	+0.04
	1.28 (m)	1.31 (dddd, *J* = 14.0, 12.2, 10.9, 6.6 Hz)	+0.03
5	4.55 (dd, *J* = 10.3, 6.6 Hz)	4.59 (t, *J* = 8.2 Hz)	+0.04
5-OH	3.68 (s)	3.68 (d, *J* = 3.9 Hz)	±0
6	-	-	-
7	4.78 (d, *J* = 8.8 Hz)	4.81 (d, *J* = 8.8 Hz)	+0.03
	4.19 (d, *J* = 8.8 Hz)	4.22 (d, *J* = 8.8 Hz)	+0.03
8	4.36 (s)	4.39 (s)	+0.03
9	3.39 (s)	3.34 (s)	–0.05
10	3.31 (s)	3.43 (s)	+0.12
11	3.77 (d, *J* = 18.9 Hz)	3.84 (d, *J* = 19.0 Hz)	+0.07
	3.45 (d, *J* = 18.9 Hz)	3.48 (d, *J* = 19.0 Hz)	+0.03
12	-	-	-
13	-	-	-
14-OH	11.26 (s)	11.30 (s)	+0.04
15	6.81 (d, *J* = 8.9 Hz)	6.84 (d, *J* = 8.9 Hz)	+0.03
16	7.10 (dd, *J* = 8.9, 2.9 Hz)	7.12 (dd, *J* = 8.9, 3.0 Hz)	+0.02
17-OH	8.19 (br, s)	8.21 (s)	+0.02
18	7.34 (d, *J* = 2.9 Hz)	7.38 (d, *J* = 2.9 Hz)	+0.04

[12] Q. Luo, X.-H. Yang, Z.-L. Yang, Z.-C. Tu, Y.-X. Cheng, *Tetrahedron* **2016**, *72*, 4564–4574.

Table 4: Comparison of ^{13}C-NMR shifts for natural and synthetic applanatumol E (**2**).

No	^{13}C-NMR (150 MHz, acetone-d_6) isolated applanatumol E **ppm**	^{13}C-NMR (151 MHz, acetone-d_6) synthetic applanatumol E **ppm**	Δ ppm
1	182.4	182.4	±0
2	53.4	53.5	–0.1
3	35.7	35.7	±0
4	31.5	31.6	+0.1
5	75.2	75.4	+0.2
6	57.4	57.4	±0
7	68.0	68.0	±0
8	110.2	110.2	±0
9	59.3	59.4	+0.1
10	57.8	57.8	±0
11	44.3	44.3	±0
12	205.1	205.2	+0.1
13	119.5	119.6	+0.1
14	156.1	156.5	+0.4
15	119.6	119.7	+0.1
16	126.0	126.2	+0.2
17	150.3	150.3	±0
18	115.3	115.3	±0

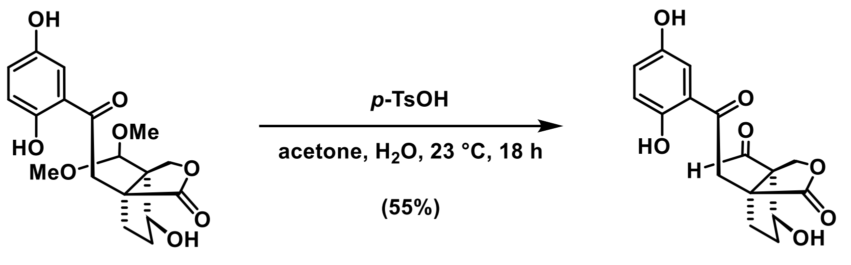

Lingzhilactone B (3)

To a solution of applanatumol E (**2**) (4.60 mg, 12.6 µmol, 1 equiv) in acetone (100 µL) was added water (1 µL) and *p*-toluenesulfonic acid monohydrate (0.200 mg, 1.26 µmol, 0.100 equiv) at 23 °C. After 18 hours, the reaction mixture was concentrated and the residue was purified by flash column chromatography (5% methanol in dichloromethane) to yield lingzhilactone B (**3**) (2.20 mg, 6.87 µmol, 55%) as a slightly yellow oil.

TLC (5% methanol in dichloromethane): R_f = 0.17 (CAM, UV).

mp: (138-139) °C

^{1}H NMR (400 MHz, acetone-d_6) δ 10.99 (s, 1H), 9.68 (s, 1H), 7.33 (d, *J* = 2.9 Hz, 1H), 7.12 (dd, *J* = 9.0, 3.0 Hz, 1H), 6.81 (d, *J* = 9.0 Hz, 1H), 4.94 (d, *J* = 9.7 Hz, 1H), 4.89 (d, *J* = 9.7 Hz, 1H), 4.83 – 4.70 (m, 1H), 4.69 – 4.64 (m, 1H), 3.78 (s, 2H), 2.04 – 1.97 (m, 3H), 1.65 – 1.55 (m, 1H).

^{13}C NMR (101 MHz, acetone-d_6) δ 205.2, 203.2, 181.2, 156.4, 150.4, 126.7, 119.7, 119.4, 115.4, 77.9, 66.9, 63.7, 54.4, 44.5, 35.5, 32.0.

IR (Diamond-ATR, neat) $\tilde{\upsilon}_{max}$: 3414 (br, m), 2965 (br, w), 1747 (s), 1719 (m), 1642 (m), 1621 (m), 1485 (m), 1383 (m), 1278 (s), 1174 (s), 1032 (m) cm^{-1}.

HRMS (ESI) calc. for $C_{16}H_{15}O_7$ [M–H]$^-$: 319.0823 found: 319.0823.

Table 5: Comparison of ^{1}H-NMR shifts for natural[13] and synthetic lingzhilactone B (**3**).

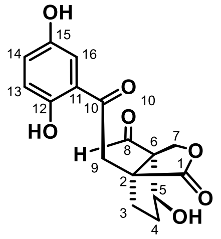

lingzhilactone B (3)

No	^{1}H-NMR (600 MHz, acetone-d_6) isolated lingzhilactone B **ppm**	^{1}H-NMR (400 MHz, acetone-d_6) synthetic lingzhilactone B **ppm**	Δ ppm
1	-	-	-
2	-	-	-
3	2.03 (m)	2.04 – 1.97 (m)	+0.01
4	2.06 (overlap)	2.04 – 1.97 (m)	–0.02
	1.58 (m)	1.65 – 1.55 (m)	–0.03
5	4.66 (dd, J = 11.0, 5.4 Hz)	4.69 – 4.64 (m)	–0.02
5-OH	-	4.83 – 4.70 (m)	-
6	-	-	-
7	4.94 (d, J = 9.7 Hz)	4.94 (d, J = 9.7 Hz)	±0
	4.88 (d, J = 9.7 Hz)	4.89 (d, J = 9.7 Hz)	+0.01
8	9.68 (s)	9.68 (s)	±0
9	3.77 (s)	3.78 (s)	+0.01
10	-	-	-
11	-	-	-
12-OH	11.00 (s)	10.99 (s)	–0.01
13	6.81 (d, J = 8.9 Hz)	6.81 (d, J = 9.0 Hz)	±0
14	7.12 (dd, J = 8.9, 2.9 Hz)	7.12 (dd, J = 9.0, 3.0 Hz)	±0
15-OH	8.35 (br, s)	-	-
16	7.33 (d, J = 2.9 Hz)	7.33 (d, J = 2.9 Hz)	±0

[13] Y.-M. Yan, X.-L. Wang, L.-L. Zhou, F.-J. Zhou, R. Li, Y. Tian, Z.-L. Zuo, P. Fang, A. C. K. Chung, F.-F. Hou, Y.-X. Cheng, *J. Ethnopharmacol.* **2015**, *176*, 385–393.

Table 6: Comparison of ^{13}C-NMR shifts for natural and synthetic lingzhilactone B (**3**).

No	^{13}C-NMR (150 MHz, acetone-d_6) isolated lingzhilactone B **ppm**	^{13}C-NMR (100 MHz, acetone-d_6) synthetic lingzhilactone B **ppm**	Δ ppm
1	181.2	181.2	±0
2	54.3	54.4	+0.1
3	35.4	35.5	+0.1
4	31.9	32.0	+0.1
5	77.8	77.9	+0.1
6	63.6	63.7	+0.1
7	66.9	66.9	±0
8	203.3	203.2	–0.1
9	44.4	44.5	+0.1
10	205.2	205.2	±0
11	119.4	119.4	±0
12	156.3	156.4	+0.1
13	126.7	126.7	±0
14	119.7	119.7	±0
15	150.4	150.4	±0
16	115.3	115.4	+0.1

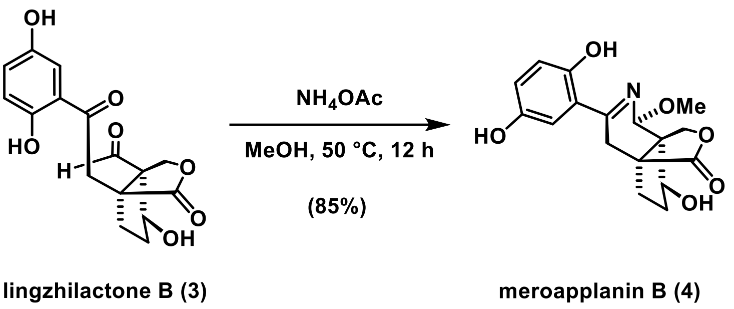

Meroapplanin B (4)

To a solution of lingzhilactone B (**3**) (12.0 mg, 38.0 µmol, 1 equiv) in methanol (100 µL) was added ammonium acetate (14.4 mg, 187 µmol, 5.00 equiv) and the mixture was heated to 50 °C. After 12 hours, the reaction mixture was allowed to cool to 23 °C and then the mixture was concentrated. The residue was purified by flash column chromatography (3% methanol in dichloromethane) to yield meroapplanin B (**4**) (10.6 mg, 32.0 µmol, 85%) as a slightly yellow solid.

TLC (3% methanol in dichloromethane): R_f = 0.31 (CAM, UV).

mp: (134-135) °C

^{1}H NMR (400 MHz, pyridine-d_5) δ 13.82 (s, 1H), 7.71 (d, J = 2.9 Hz, 1H), 7.31 (dd, J = 8.8, 2.8 Hz, 1H), 7.19 (d, J = 8.8 Hz, 2H), 5.16 (d, J = 9.8 Hz, 1H), 4.71 (s, 1H), 4.63 (dd, J = 7.1, 5.0 Hz, 1H), 4.47 (d, J = 9.8 Hz, 1H), 3.95 (d, J = 15.4 Hz, 1H), 3.51 (s, 3H), 2.51 – 2.46 (m, 1H), 2.40 (d, J = 15.4 Hz, 1H), 2.14 – 2.08 (m, 1H), 1.91 – 1.85 (m, 1H), 1.84 – 1.78 (m, 1H).

^{13}C NMR (151 MHz, pyridine-d_5) δ 182.1, 173.6, 155.8, 151.4, 123.1, 119.6, 119.2, 115.3, 95.1, 78.1, 68.3, 58.3, 56.8, 54.0, 36.0, 34.3, 33.3.

IR (Diamond-ATR, neat) $\tilde{\upsilon}_{max}$: 3420 (br, w), 2924 (s), 2853 (m), 1744 (s), 1575 (w), 1456 (w), 1375 (m), 1221 (s), 1178 (m), 1051 (m), 1018 (m) cm^{-1}.

HRMS (ESI) calc. for $C_{17}H_{20}NO_6$ $[M+H]^+$: 334.1285 found: 334.1269; calc. for $C_{17}H_{19}NNaO_6$ $[M+Na]^+$: 356.1105 found: 356.1088; calc. for $C_{17}H_{19}KNO_6$ $[M+K]^+$: 372.0844 found: 372.0827.

Table 7: Comparison of ^{1}H-NMR shifts for natural[14] and synthetic meroapplanin B (**4**).

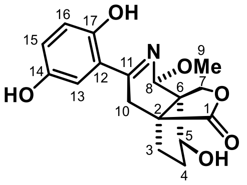

meroapplanin B (4)

No	^{1}H-NMR (600 MHz, pyridine-d_5) isolated meroapplanin B **ppm**	^{1}H-NMR (400 MHz, pyridine-d_5) synthetic meroapplanin B **ppm**	Δ ppm
1	-	-	-
2	-	-	-
3	2.44 (m)	2.51 – 2.46 (m)	+0.02
	1.78 (m)	1.91 – 1.85 (m)	+0.07
4	2.05 (m)	2.14 – 2.08 (m)	+0.03
	1.81 (m)	1.84 – 1.78 (m)	+0.03
5	4.59 (br, s)	4.63 (dd, *J* = 7.1, 5.0 Hz)	+0.04
5-OH	-	-	-
6	-	-	-
7	5.13 (d, *J* = 9.9 Hz)	5.16 (d, *J* = 9.8 Hz)	+0.03
	4.44 (d, *J* = 9.9 Hz)	4.47 (d, *J* = 9.8 Hz)	+0.03
8	4.68 (s)	4.71 (s)	+0.04
9	3.48 (s)	3.51 (s)	+0.03
10	3.92 (d, *J* = 15.4 Hz)	3.95 (d, *J* = 15.4 Hz)	+0.03
	2.34 (d, *J* = 15.4 Hz)	2.40 (d, *J* = 15.4 Hz)	+0.06
11	-	-	-
12	-	-	-
13	7.68 (d, *J* = 2.8 Hz)	7.71 (d, *J* = 2.9 Hz)	+0.03
14-OH	-	-	-
15	7.15 (d, *J* = 8.8 Hz)	7.19 (d, *J* = 8.8 Hz)	+0.04
16	7.28 (dd, *J* = 8.8, 2.8 Hz)	7.31 (dd, *J* = 8.8, 2.8 Hz)	+0.03
17-OH	-	13.82 (s)	-

[14] X.-R. Peng, Q.-Q. Shi, J. Yang, H.-G. Su, L. Zhou, M.-H. Qiu, *J. Org. Chem.* **2020**, *85*, 7446–7451.

Table 8: Comparison of ^{13}C-NMR shifts for natural and synthetic meroapplanin B (**4**).

No	^{13}C-NMR (150 MHz, pyridine-d_5) isolated meroapplanin B **ppm**	^{13}C-NMR (150 MHz, pyridine-d_5) synthetic meroapplanin B **ppm**	Δ ppm
1	181.5	182.1	+0.6
2	57.6	58.3	+0.7
3	35.4	36.0	+0.6
4	33.7	34.3	+0.6
5	77.5	78.1	+0.6
6	53.4	54.0	+0.6
7	67.7	68.3	+0.6
8	94.5	95.1	+0.6
9	56.2	56.8	+0.6
10	32.7	33.3	+0.6
11	173.0	173.6	+0.6
12	118.6	119.2	+0.6
13	114.6	115.3	+0.7
14	150.8	151.4	+0.6
15	122.5	123.1	+0.6
16	119.0	119.6	+0.6
17	155.2	155.8	+0.6

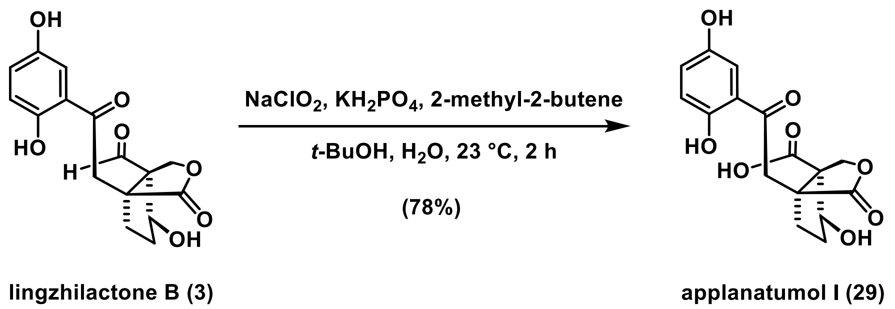

Applanatumol I (29)

To a solution of lingzhilactone B (**3**) (2.20 mg, 6.87 µmol, 1 equiv) and potassium dihydrogen phosphate (1.87 mg, 13.7 µmol, 2.00 equiv) in *tert*-butanol (180 µL), 2-methyl-2-butene (60 µL) and water (60 µL) was added sodium chlorite (2.33 mg, 20.6 µmol, 3.00 equiv) at 0 °C. After one hour, the reaction mixture was allowed to warm to 23 °C. After one hour at 23 °C, a saturated aqueous solution of ammonium chloride (5 mL) and ethyl acetate (5 mL) were added to the reaction mixture and the layers were separated. The aqueous layer was extracted with ethyl acetate (3 × 2 mL) and the combined organic layers were dried over sodium sulfate. The dried solution was filtered and the filtrate was concentrated to yield applanatumol I (**29**) (1.80 mg, 5.35 µmol, 78%) as a colourless solid.

Note: The product is instable on silica gel and analytical data of crude applanatumol I were obtained.

TLC (20% methanol in dichloromethane): R_f = 0.20 (CAM).

^{1}H NMR (600 MHz, methanol-d_4) δ 7.23 (d, *J* = 3.0, Hz, 1H), 7.01 (dd, *J* = 9.0, 2.9 Hz, 1H), 6.78 (d, *J* = 9.0 Hz, 1H), 4.93 (d, *J* = 9.7 Hz, 1H), 4.83 (d, *J* = 9.7 Hz, 1H), 4.54 (dd, *J* = 11.2, 5.8 Hz, 1H), 4.06 (d, *J* = 18.8 Hz, 1H), 3.59 (d, *J* = 18.8 Hz, 1H), 2.07 – 2.03 (m, 1H), 2.03 – 1.99 (m, 1H), 1.94 (td, *J* = 12.8, 5.4 Hz, 1H), 1.55 – 1.46 (m, 1H).

^{13}C NMR (151 MHz, methanol-d_4) δ 205.2, 184.0, 176.2, 156.5, 150.8, 126.4, 120.0, 119.8, 115.3, 80.9, 68.8, 60.4, 55.3, 46.3, 36.0, 32.1.

IR (Diamond-ATR, neat) $\tilde{\upsilon}_{max}$: 3381 (br, w), 2927 (m), 2856 (w), 1734 (s), 1643 (m), 1485 (w), 1381 (m), 1227 (s), 1178 (m), 1098 (w), 1023 (m), 834 (w), 782 (w) cm^{-1}.

HRMS (ESI) calc. for $C_{16}H_{16}NaO_8$ $[M+Na]^+$: 359.0737 found: 359.0728.

Table 9: Comparison of ^{1}H-NMR shifts for natural[12] and synthetic applanatumol I (**29**).

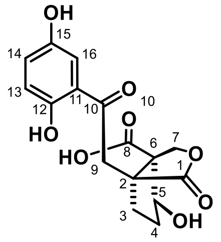

lingzhilactone I (29)

No	^{1}H-NMR (600 MHz, methanol-d_4) isolated applanatumol I **ppm**	^{1}H-NMR (600 MHz, methanol-d_4) synthetic applanatumol I **ppm**	Δ ppm
1	-	-	-
2	-	-	-
3	2.06 (m)	2.07 – 2.03 (m)	–0.1
	1.96 (m)	1.94 (td, *J* = 12.8, 5.4 Hz)	–0.2
4	2.02 (m)	2.03 – 1.99 (m)	–0.1
	1.51 (m)	1.55 – 1.46 (m)	–0.1
5	4.54 (dd, *J* = 11.2, 5.8 Hz)	4.54 (dd, *J* = 11.2, 5.8 Hz)	±0
5-OH	-	-	-
6	-	-	-
7	4.93 (d, *J* = 9.7 Hz)	4.93 (d, *J* = 9.7 Hz)	±0
	4.83 (d, *J* = 9.7 Hz)	4.83 (d, *J* = 9.7 Hz)	±0
8	-	-	-
9	4.05 (d, *J* = 18.8 Hz)	4.06 (d, *J* = 18.8 Hz)	+0.1
	3.60 (d, *J* = 18.8 Hz)	3.59 (d, *J* = 18.8 Hz)	–0.1
10	-	-	-
11	-	-	-
12-OH	-	-	-
13	6.79 (d, *J* = 8.9 Hz)	6.78 (d, *J* = 9.0 Hz)	–0.1
14	7.02 (dd, *J* = 8.9, 2.9 Hz)	7.01 (dd, *J* = 9.0, 3.0 Hz)	–0.1
15-OH	-	-	-
16	7.24 (d, *J* = 2.9 Hz)	7.23 (d, *J* = 3.0 Hz)	–0.1

Table 10: Comparison of ^{13}C-NMR shifts for natural and synthetic applanatumol I (**29**).

No	^{13}C-NMR (150 MHz, methanol-d_4) isolated applanatumol I **ppm**	^{13}C-NMR (151 MHz, methanol-d_4) synthetic applanatumol I **ppm**	Δ ppm
1	183.9	184.0	+0.1
2	55.3	55.3	±0
3	36.0	36.0	±0
4	32.0	32.1	+0.1
5	80.8	80.9	+0.1
6	60.4	60.4	±0
7	68.7	68.8	+0.1
8	176.1	176.2	+0.1
9	46.3	46.3	±0
10	205.1	205.2	+0.1
11	119.9	120.0	+0.1
12	156.5	156.5	±0
13	119.8	119.8	±0
14	126.4	126.4	±0
15	150.8	150.8	±0
16	115.3	115.3	±0

2.4. Total Synthesis of Applanatumol H (28)

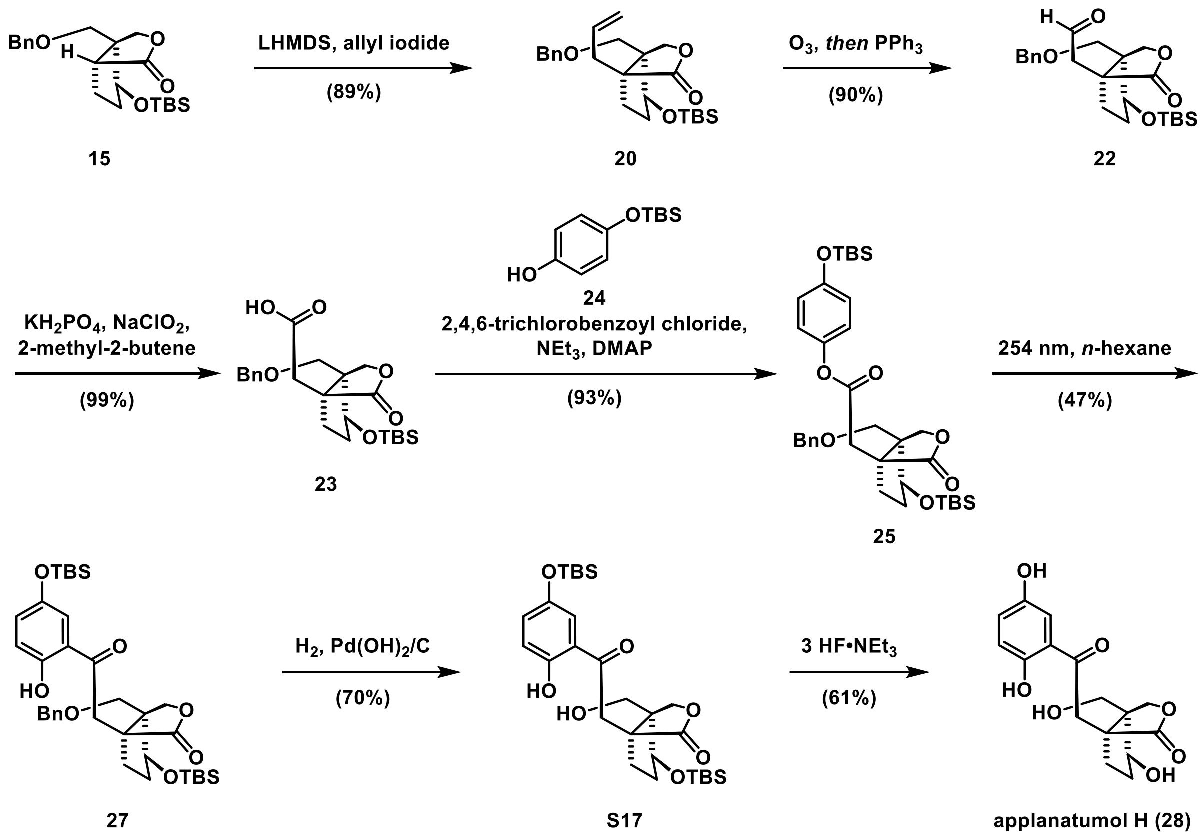

Scheme 3: Total synthesis of applanatumol H (**28**).

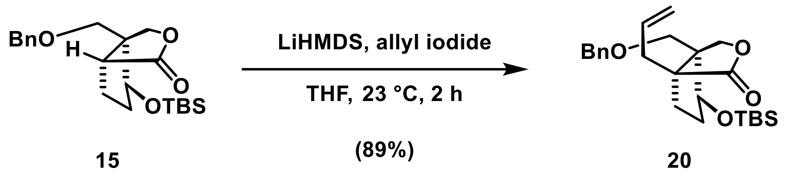

Lactone 20

To a solution of lactone **15** (442 mg, 1.17 mmol, 1 equiv) and allyl iodide (1.10 mL, 11.7 mmol, 10.0 equiv) in tetrahydrofuran (12 mL) was added a lithium bis(trimethylsilyl)amide solution (1.00 M in tetrahydrofuran, 3.52 mL, 3.52 mmol, 3.00 equiv) at 23 °C. After two hours, water (20 mL) was added to the reaction mixture and the layers were separated. The aqueous layer was extracted with diethyl ether (3 × 10 mL) and the combined organic layers were dried over sodium sulfate. The dried solution was filtered and the filtrate was concentrated. The residue was purified by flash column chromatography on silica gel (5% ethyl acetate in cyclohexane) to yield lactone **20** (435 mg, 1.04 mmol, 89%) as a slightly yellow oil.

TLC (30% ethyl acetate in cyclohexane): R_f = 0.65 (CAM).

^{1}H NMR (600 MHz, chloroform-*d*) δ 7.36 (dd, *J* = 8.3, 6.2 Hz, 2H), 7.33 – 7.28 (m, 3H), 5.87 (ddt, *J* = 17.2, 10.2, 7.2 Hz, 1H), 5.08 – 5.03 (m, 2H), 4.56 (d, *J* = 9.4 Hz, 1H), 4.49 (s, 2H), 4.27 (dd, *J* = 9.0, 6.2 Hz, 1H), 3.75 (d, *J* = 9.4 Hz, 1H), 3.52 (d, *J* = 9.4 Hz, 1H), 3.41 (d, *J* = 9.4 Hz, 1H), 2.55 (dd, *J* = 14.4, 7.4 Hz, 1H), 2.44 (ddt, *J* = 14.4, 7.1, 1.7 Hz, 1H), 2.07 (ddd, *J* = 13.2, 7.3, 2.5 Hz, 1H), 1.91 (dtd, *J* = 13.0, 6.6, 2.5 Hz, 1H), 1.62 (ddd, *J* = 13.2, 11.6, 6.8 Hz, 1H), 1.46 – 1.39 (m, 1H), 0.87 – 0.83 (m, 9H), 0.03 – 0.01 (m, 6H).

^{13}C NMR (151 MHz, chloroform-*d*) δ 182.2, 137.7, 133.9, 128.6, 128.0, 127.9, 118.5, 76.3, 73.6, 70.3, 68.6, 55.2, 54.3, 37.3, 33.5, 32.6, 25.8, 18.0, –4.3, –5.0.

IR (Diamond-ATR, neat) $\tilde{\upsilon}_{max}$: 2954 (w), 2929 (w), 2856 (w), 1765 (s), 1463 (w), 1362 (w), 1252 (m), 1144 (s), 1124 (s), 1029 (m), 837 (s), 698 (m), 673 (m) cm^{-1}.

HRMS (ESI) calc. for $C_{24}H_{36}NaO_4Si$ $[M+Na]^+$: 439.2275 found: 439.2240.

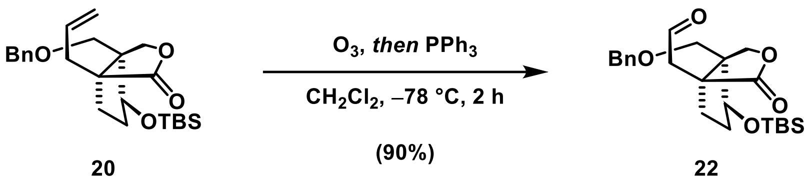

Aldehyde 22

Through a solution of lactone **20** (342 mg, 821 µmol, 1 equiv) in dichloromethane (8 mL) was sparged a stream of ozone at −78 °C. After ten minutes, the reaction mixture turned blue and then oxygen was sparged through the blue reaction mixture until the blue colour disappeared. Triphenylphosphine (652 mg, 2.46 mmol, 3.00 equiv) was added to the reaction mixture. The reaction mixture was allowed to warm to 23° C. After two hours at 23 °C, the mixture was concentrated and the residue was purified by flash column chromatography on silica gel (10% ethyl acetate in cyclohexane) to yield aldehyde **22** (308 mg, 736 µmol, 90%) as a colourless solid.

TLC (30% ethyl acetate in cyclohexane): R_f = 0.38 (CAM).

mp: (111-114) °C

^{1}H NMR (600 MHz, chloroform-*d*) δ 9.52 (s, 1H), 7.38 – 7.34 (m, 2H), 7.33 – 7.31 (m, 1H), 7.28 – 7.26 (m, 2H), 4.66 (d, *J* = 9.4 Hz, 1H), 4.46 (d, *J* = 11.7 Hz, 1H), 4.35 (dd, *J* = 11.1, 6.2 Hz, 2H), 3.82 (d, *J* = 9.4 Hz, 1H), 3.35 (d, *J* = 9.7 Hz, 1H), 3.29 (d, *J* = 9.7 Hz, 1H), 3.08 (dd, *J* = 18.7, 1.1 Hz, 1H), 2.90 (d, *J* = 18.7 Hz, 1H), 2.06 (dd, *J* = 13.0, 6.8 Hz, 1H), 1.91 – 1.86 (m, 1H), 1.64 (td, *J* = 13.2, 6.2 Hz, 1H), 1.50 – 1.43 (m, 1H), 0.87 (s, 9H), 0.06 – 0.01 (m, 6H).

^{13}C NMR (151 MHz, chloroform-*d*) δ 199.9, 182.2, 137.3, 128.7, 128.3, 128.2, 75.3, 73.7, 69.7, 68.5, 54.0, 51.0, 48.2, 34.6, 31.5, 25.9, 18.1, –4.3, –5.0.

IR (Diamond-ATR, neat) $\tilde{\upsilon}_{max}$: 2954 (w), 2928 (w), 2855 (w), 1765 (s), 1721 (m), 1386 (w), 1252 (m), 1153 (m), 1130 (m), 1034 (m), 871 (m), 837 (s), 777 (m), 699 (m) cm^{-1}.

HRMS (ESI) calc. for $C_{23}H_{34}NaO_5Si$ $[M+Na]^+$: 441.2068 found: 441.2080.

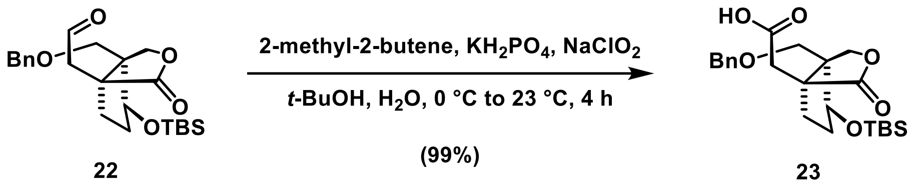

22 23

Acid 23

To a solution of aldehyde **22** (221 mg, 528 µmol, 1 equiv) and potassium dihydrogen phosphate (144 mg, 1.06 mmol, 2.00 equiv) in *tert*-butanol (3 mL), 2-methyl-2-butene (1 mL) and water (1 mL) was added sodium chlorite (179 mg, 1.58 mmol, 3.00 equiv) at 0 °C. After four hours, a saturated aqueous solution of ammonium chloride (10 mL) and ethyl acetate (5 mL) were added to the reaction mixture and the layers were separated. The aqueous layer was extracted with ethyl acetate (3 × 5 mL) and the combined organic layers were dried over sodium sulfate. The dried solution was filtered and the filtrate was concentrated. The residue was purified by flash column chromatography on silica gel (5% methanol in dichloromethane) to yield acid **23** (228 mg, 525 µmol, 99 %) as a colourless solid.

TLC (3% methanol in dichloromethane): R_f = 0.24 (CAM).

mp: (100-101) °C

^{1}H NMR (400 MHz, chloroform-*d*) δ 7.37 – 7.28 (m, 5H), 4.68 (d, *J* = 9.3 Hz, 1H), 4.48 – 4.42 (m, 2H), 4.35 (dd, *J* = 10.7, 6.3 Hz, 1H), 3.78 (d, *J* = 9.4 Hz, 1H), 3.50 (d, *J* = 9.8 Hz, 1H), 3.35 (d, *J* = 9.8 Hz, 1H), 3.08 (d, *J* = 17.8 Hz, 1H), 2.90 (d, *J* = 17.8 Hz, 1H), 2.07 (dd, *J* = 12.9, 6.7 Hz, 1H), 1.88 (dt, *J* = 12.2, 6.1 Hz, 1H), 1.65 (dt, *J* = 13.2, 6.7 Hz, 1H), 1.51 – 1.42 (m, 1H), 0.86 (s, 9H), 0.06 – 0.00 (m, 6H).

^{13}C NMR (151 MHz, chloroform-*d*) δ 183.0, 174.5, 137.3, 128.7, 128.2, 75.1, 73.9, 69.6, 68.8, 53.9, 51.7, 38.0, 34.6, 31.4, 25.8, 18.1, –4.3, –5.0.

IR (Diamond-ATR, neat) $\tilde{\upsilon}_{max}$: 3008 (br, w), 2927 (m), 2855 (m), 1769 (s), 1743 (s), 1463 (w), 1362 (w), 1252 (m), 1154 (s), 1132 (s), 1034 (s), 894 (m), 862 (m), 837 (m), 777 (m), 699 (w) cm^{-1}.

HRMS (ESI) calc. for $C_{23}H_{33}O_6Si$ $[M–H]^-$: 433.2052 found: 433.2056.

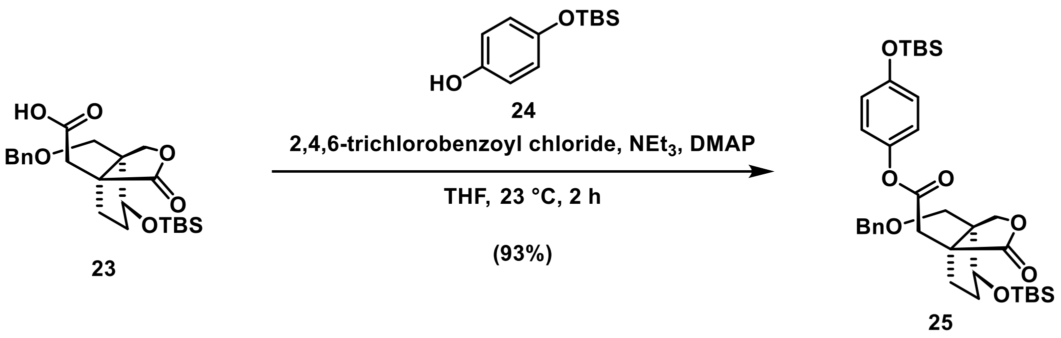

Ester 25

To a solution of acid **23** (66 mg, 152 µmol, 1 equiv) and phenol **24** (44.3 mg, 197 µmol, 1.30 equiv) in tetrahydrofuran (1.5 mL) was added triethylamine (169 µl, 1.21 mmol, 8.00 equiv) followed by 2,4,6-trichlorobenzoyl chloride (121 µl, 759 µmol, 5.00 equiv) at 23 °C. After 15 minutes, 4-(dimethylamino)pyridine (1.87 mg, 15.0 µmol, 0.100 equiv) was added. After two hours, the reaction mixture was concentrated and the residue was purified by flash column chromatography on silica gel (10% ethyl acetate in cyclohexane) to yield ester **25** (90.0 mg, 140 µmol, 93%) as a colourless solid.

TLC (30% ethyl acetate in cyclohexane): R_f = 0.62 (CAM, UV).

mp: (78-79) °C

^{1}H NMR (400 MHz, chloroform-*d*) δ 7.41 – 7.31 (m, 5H), 6.86 – 6.82 (m, 2H), 6.79 – 6.74 (m, 2H), 4.68 (d, *J* = 9.3 Hz, 1H), 4.50 – 4.46 (m, 2H), 4.43 (dd, *J* = 10.9, 6.3 Hz, 1H), 3.75 (d, *J* = 9.3 Hz, 1H), 3.57 (d, *J* = 9.8 Hz, 1H), 3.39 (d, *J* = 9.8 Hz, 1H), 3.26 (d, *J* = 18.0 Hz, 1H), 3.13 (d, *J* = 18.0 Hz, 1H), 2.10 (dd, *J* = 12.9, 6.5 Hz, 1H), 1.88 (dt, *J* = 12.0, 6.0 Hz, 1H), 1.69 (td, *J* = 13.2, 6.0 Hz, 1H), 1.51 – 1.40 (m, 1H), 0.97 (s, 9H), 0.87 (s, 9H), 0.18 (s, 6H), 0.06 – 0.01 (m, 6H).

^{13}C NMR (101 MHz, chloroform-*d*) δ 182.3, 153.5, 144.4, 137.5, 128.8, 128.5, 128.2, 128.1, 122.2, 120.6, 75.2, 74.0, 69.9, 68.3, 53.6, 38.5, 34.8, 31.4, 25.9, 25.8, 18.1, –4.3, –4.3, –5.0.

IR (Diamond-ATR, neat) $\tilde{\upsilon}_{max}$: 2955 (w), 2929 (w), 2857 (w), 1767 (m), 1502 (s), 1254 (m), 1189 (m), 1145 (s), 1035 (m), 913 (m), 837 (s), 778 (s), 698 (w) cm^{-1}.

HRMS (ESI) calc. for $C_{35}H_{52}NaO_7Si_2$ $[M+Na]^+$: 663.3144 found: 663.3157.

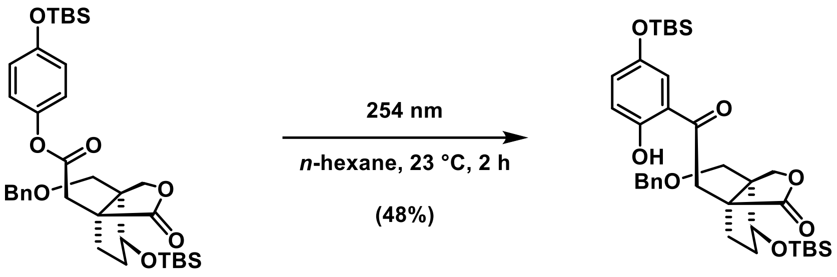

25 27

Ketone 27

Reaction setup: The reaction was carried out in the Rayonet RPR-200 Photochemical Reactor, with the reaction mixture placed in the center of the 25 cm wide reaction chamber (approximately 12 cm away from the light source), above a cooling fan and surrounded by a circular array of 16 light tubes.

A solution of ester **25** (93.9 mg, 146 µmol, 1 equiv) in degassed *n*-hexane (6 mL) was irradiated at 254 nm (Southern New England Ultraviolet Company, RPR-2537A° lamps) in a quartz tube. *Note: The reaction was set up in three parallel batches (31.3 mg and 2 mL n-hexane each) and irradiated at the same time.* After two hours, the reaction mixture was concentrated and the residue was purified by flash column chromatography on silica gel (5% ethyl acetate in cyclohexane) to yield ketone **27** (45.0 mg, 70.0 µmol, 48%) as a slightly yellow solid.

TLC (10% ethyl acetate in cyclohexane): R_f = 0.39 (CAM, UV).

mp: (78-79) °C

^{1}H NMR (400 MHz, chloroform-*d*) δ 11.39 (s, 1H), 7.20 – 7.14 (m, 3H), 7.07 – 7.01 (m, 4H), 6.87 (d, *J* = 8.8 Hz, 1H), 4.75 (d, *J* = 9.2 Hz, 1H), 4.46 (dd, *J* = 11.1, 6.1 Hz, 1H), 4.29 (d, *J* = 11.5 Hz, 1H), 4.13 (d, *J* = 11.5 Hz, 1H), 3.87 – 3.78 (m, 2H), 3.47 (d, *J* = 18.8 Hz, 1H), 3.36 (d, *J* = 9.7 Hz, 1H), 3.27 (d, *J* = 9.7 Hz, 1H), 2.07 (dd, *J* = 12.8, 6.3 Hz, 1H), 1.88 (dt, *J* = 11.9, 5.9 Hz, 1H), 1.72 (td, *J* = 13.3, 5.8 Hz, 1H), 1.54 – 1.45 (m, 1H), 0.99 (s, 9H), 0.89 (s, 9H), 0.17 (s, 6H), 0.08 – 0.01 (m, 6H).

^{13}C NMR (101 MHz, chloroform-*d*) δ 203.7, 183.0, 157.2, 147.5, 137.0, 129.8, 128.6, 128.1, 119.5, 119.2, 118.6, 75.0, 73.9, 69.8, 68.4, 53.4, 51.5, 43.9, 35.1, 31.1, 25.9, 25.8, 18.3, 18.1, –4.2, –4.3, –4.9.

IR (Diamond-ATR, neat) $\tilde{\upsilon}_{max}$: 2955 (m), 2930 (m), 2857 (m), 1768 (s), 1644 (w), 1614 (w), 1483 (s), 1363 (w), 1275 (m), 1255 (s), 1176 (m), 1154 (m), 1132 (m), 1037 (m), 953 (m), 839 (s), 752 (m), 699 (w) cm^{-1}.

HRMS (ESI) calc. for $C_{35}H_{52}NaO_7Si_2$ $[M+Na]^+$: 663.3144 found: 663.3157.

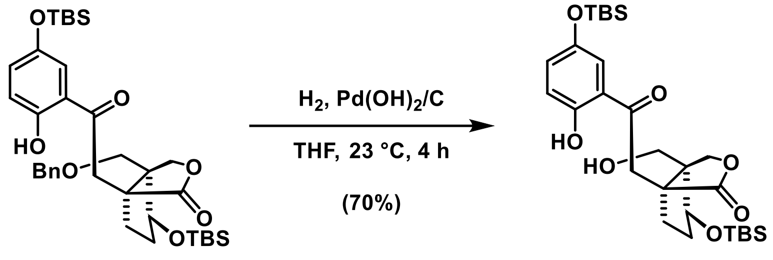

Alcohol S17

To a solution of ketone **27** (43.0 mg, 67.0 µmol, 1 equiv) in tetrahydrofuran (4 mL) was added $Pd(OH)_2/C$ (20 wt%, 31.4 mg, 34.0 µmol, 0.500 equiv). The reaction vessel was placed in a high-pressure autoclave and exposed to hydrogen pressure of 40.0 bar. After four hours, the gas was released and the autoclave was purged with nitrogen for one minute. The reaction mixture was filtered through a pad of Celite and the pad washed with dichloromethane (5 mL). The filtrate was concentrated and the residue was purified by flash column chromatography on silica gel (5% grading to 20% ethyl acetate in cyclohexane) to yield alcohol **S17** (26.0 mg, 47.0 µmol, 70%) as a colourless solid.

TLC (20% ethyl acetate in cyclohexane): R_f = 0.20 (CAM, UV).

mp: (169-170) °C

^{1}**H NMR** (600 MHz, chloroform-*d*) δ 11.43 (s, 1H), 7.14 (d, *J* = 2.9 Hz, 1H), 7.02 (dd, *J* = 9.0, 2.9 Hz, 1H), 6.86 (d, *J* = 8.9 Hz, 1H), 4.77 (d, *J* = 9.3 Hz, 1H), 4.45 (dd, *J* = 11.1, 6.1 Hz), 3.89 – 3.84 (m, 2H), 3.62 (dd, *J* = 10.5, 3.0 Hz, 1H), 3.55 – 3.49 (m, 2H), 2.11 (dd, *J* = 12.8, 6.4 Hz, 1H), 1.92 (dt, *J* = 12.0, 5.9 Hz, 1H), 1.75 (td, *J* = 13.3, 5.8 Hz, 1H), 1.54 – 1.49 (m, 1H), 0.99 (s, 9H), 0.90 (s, 9H), 0.19 – 0.18 (m, 6H), 0.10 – 0.07 (m, 6H).

^{13}**C NMR** (151 MHz, chloroform-*d*) δ 204.0, 183.0, 157.2, 147.5, 130.0, 119.5, 119.3, 118.7, 75.1, 68.4, 62.8, 53.8, 51.5, 43.6, 35.1, 31.2, 25.9, 25.8, 18.3, 18.1, –4.2, –4.3, –4.3, –4.9.

IR (Diamond-ATR, neat) $\tilde{\nu}_{max}$: 3457 (br, w), 2955 (m), 2929 (m), 2858 (m), 1747 (s), 1484 (s), 1255 (s), 1174 (m), 954 (m), 838 (s), 778 (m), 728 (w) cm^{-1}.

HRMS (ESI) calc. for $C_{28}H_{46}NaO_7Si_2$ $[M+Na]^+$: 573.2674 found: 573.2665.

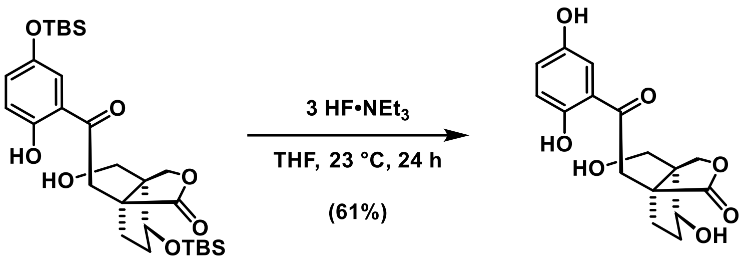

S17 applanatumol H (28)

Applanatumol H (28)

To a solution of alcohol **S17** (7.80 mg, 14.0 µmol, 1 equiv) in tetrahydrofuran (100 µL) was added hydrogen fluoride triethylamine (118 µL, 708 µmol, 50.0 equiv) at 23 °C. After 24 hours, a saturated aqueous solution of sodium bicarbonate (5 mL) and ethyl acetate (5 mL) were added to the reaction mixture and the layers were separated. The aqueous layer was extracted with ethyl acetate (3 × 2 mL) and the combined organic layers were dried over sodium sulfate. The dried solution was filtered and the filtrate was concentrated. The residue was purified by flash column chromatography on silica gel (5% grading to 20% methanol in dichloromethane) to yield applanatumol H (**28**) (2.80 mg, 8.69 µmol, 61%) as a colourless solid.

TLC (10% methanol in dichloromethane): R_f = 0.33 (CAM, UV).

^{1}H NMR (400 MHz, methanol-d_4) δ 7.24 (d, *J* = 2.9 Hz, 1H), 7.02 (dd, *J* = 8.9, 2.9 Hz, 1H), 6.80 (d, *J* = 9.0 Hz, 1H), 4.75 (d, *J* = 9.0 Hz, 1H), 4.38 (dd, *J* = 11.0, 6.2 Hz, 1H), 4.01 – 3.95 (m, 2H), 3.53 – 3.47 (m, 3H), 2.03 – 1.94 (m, 2H), 1.79 (td, *J* = 13.1, 5.8 Hz, 1H), 1.42 (dddd, *J* = 15.8, 13.3, 11.4, 6.6 Hz, 1H).

^{13}C NMR (151 MHz, methanol-d_4) δ 205.5, 186.1, 156.5, 150.8, 126.3, 120.2, 119.8, 115.5, 75.9, 70.0, 63.2, 54.3, 53.7, 44.6, 35.8, 31.8.

IR (Diamond-ATR, neat) $\tilde{\nu}_{max}$: 3389 (br, m), 2956 (m), 2921 (s), 2852 (m), 1739 (s), 1486 (m), 1277 (s), 1174 (s), 1020 (m), 783 (w) cm^{-1}.

HRMS (ESI) calc. for $C_{16}H_{18}NaO_7$ $[M+Na]^+$: 345.0945 found: 345.0933.

Table 11: Comparison of ^{1}H-NMR shifts for natural[12] and synthetic applanatumol H (**28**).

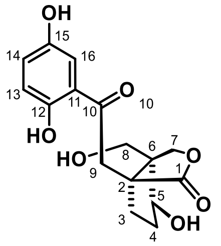

applanatumol H (28)

No	^{1}H-NMR (600 MHz, methanol-d_4) isolated applanatumol H **ppm**	^{1}H-NMR (400 MHz, methanol-d_4) synthetic applanatumol H **ppm**	Δ ppm
1	-	-	-
2	-	-	-
3	1.98 (overlap)	2.03 – 1.94 (m, overlap)	+0.01
	1.78 (m)	1.79 (td, *J* = 13.1, 5.8 Hz)	+0.01
4	1.98 (overlap)	2.03 – 1.94 (m, overlap)	+0.01
	1.42 (m)	1.42 (dddd, *J* = 15.8, 13.3, 11.4, 6.6 Hz)	±0
5	4.38 (dd, *J* = 10.8, 6.1 Hz)	4.38 (dd, *J* = 11.0, 6.2 Hz)	±0
5-OH	-	-	-
6	-	-	-
7	4.75 (d, *J* = 9.1 Hz)	4.75 (d, *J* = 9.0 Hz)	±0
	4.00 (d, *J* = 9.1 Hz)	4.01 – 3.95 (m, overlap)	–0.02
8	3.51 (s)	3.53 – 3.47 (m, overlap)	–0.01
9	3.97 (d, *J* = 15.9 Hz)	4.01 – 3.95 (m, overlap)	+0.01
	3.48 (d, *J* = 15.9 Hz)	3.53 – 3.47 (m, overlap)	+0.02
10	-	-	-
11	-	-	-
12-OH	-	-	-
13	6.80 (d, *J* = 8.9 Hz)	6.80 (d, *J* = 9.0 Hz)	±0
14	7.02 (dd, *J* = 8.9, 2.9 Hz)	7.02 (dd, *J* = 8.9, 2.9 Hz)	±0
15-OH	-	-	-
16	7.24 (d, *J* = 2.9 Hz)	7.24 (d, *J* = 2.9 Hz)	±0

Table 12: Comparison of ^{13}C-NMR shifts for natural and synthetic applanatumol H (**28**).

No	^{13}C-NMR (150 MHz, methanol-d_4) isolated applanatumol H **ppm**	^{13}C-NMR (151 MHz, methanol-d_4) synthetic applanatumol H **ppm**	Δ ppm
1	186.1	186.1	±0
2	53.8	53.7	–0.1
3	35.8	35.8	±0
4	31.7	31.8	+0.1
5	76.0	75.9	–0.1
6	54.3	54.3	±0
7	70.0	70.0	±0
8	63.2	63.2	±0
9	44.8	44.6 (low intensity due to deuteration in methanol-d_4)	–0.2
10	205.4	205.5	+0.1
11	120.0	120.2	+0.2
12	156.4	156.5	+0.1
13	119.8	119.8	±0
14	126.3	126.3	±0
15	150.6	150.8	+0.2
16	115.4	115.5	+0.1

2.5. Total Synthesis of Lingzhiol

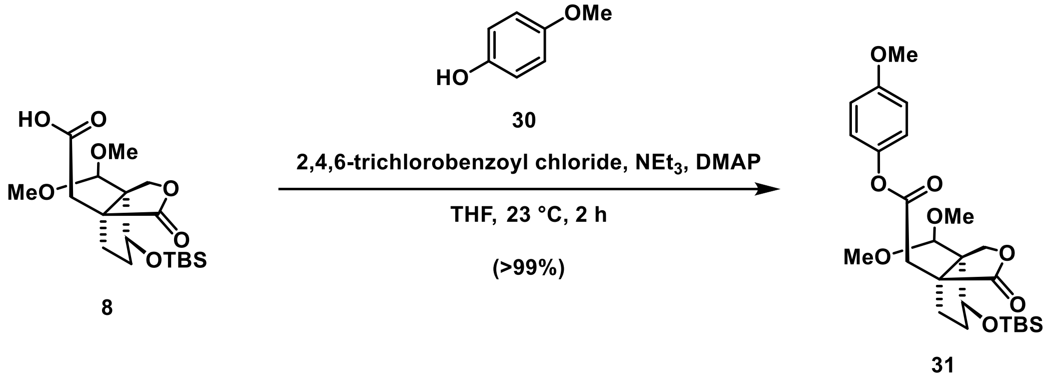

Ester 31

To a solution of acid **8** (97.4 mg, 251 µmol, 1 equiv) and 4-methoxyphenol (45.0 mg, 326 µmol, 1.30 equiv) in tetrahydrofuran (2.5 mL) was added triethylamine (279 µl, 2.01 mmol, 8.00 equiv) followed by 2,4,6-trichlorobenzoyl chloride (200 µl, 1.25 mmol, 5.00 equiv) at 23 °C. After 15 minutes, 4-(dimethylamino)pyridine (3.09 mg, 25.0 µmol, 0.100 equiv) was added. After two hours, the reaction mixture was concentrated and the residue was purified by flash column chromatography on silica gel (5% ethyl acetate in cyclohexane) to yield ester **31** (124 mg, 251 µmol, >99%) as a colourless solid.

TLC (20% ethyl acetate in cyclohexane): R_f = 0.28 (CAM, UV).

mp: (123-124) °C

^{1}H NMR (700 MHz, chloroform-*d*) δ 7.01 (d, *J* = 9.1 Hz, 1H), 6.88 (d, *J* = 9.1 Hz, 1H), 4.76 (d, *J* = 9.0 Hz, 1H), 4.55 (dd, *J* = 9.7, 6.7 Hz, 1H), 4.34 (s, 1H), 4.10 (d, *J* = 9.0 Hz, 1H), 3.79 (s, 3H), 3.51 (s, 3H), 3.49 (s, 3H), 3.13 – 3.04 (m, 2H), 2.09 – 2.05 (m, 1H), 1.96 (dtd, *J* = 12.8, 6.6, 1.7 Hz, 1H), 1.71 (td, *J* = 12.9, 6.5 Hz, 1H), 1.41 – 1.37 (m, 1H).

^{13}C NMR (176 MHz, chloroform-*d*) δ 181.8, 170.7, 157.5, 144.1, 122.3, 114.6, 108.8, 75.2, 68.2, 59.2, 58.2, 57.7, 55.8, 53.3, 38.4, 34.9, 32.9, 25.9, 18.1, –4.3, –4.9.

IR (Diamond-ATR, neat) $\tilde{\upsilon}_{max}$: 2955 (m), 2929 (m), 2855 (w), 1767 (s), 1506 (s), 1251 (m), 1195 (s), 1149 (s), 1070 (m), 1028 (m), 839 (m), 777 (w) cm^{-1}.

HRMS (ESI) calc. for $C_{25}H_{38}NaO_8Si$ $[M+Na]^+$: 517.2228 found: 517.2226.

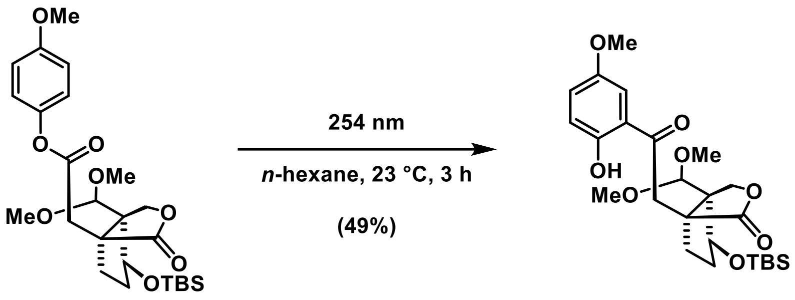

31 32

Ketone 32

Reaction setup: The reaction was carried out in the Rayonet RPR-200 Photochemical Reactor, with the reaction mixture placed in the center of the 25 cm wide reaction chamber (approximately 12 cm away from the light source), above a cooling fan and surrounded by a circular array of 16 light tubes.

A solution of ester **31** (100 mg, 202 µmol, 1 equiv) in degassed *n*-hexane (12 mL) was irradiated at 254 nm (Southern New England Ultraviolet Company, RPR-2537A° lamps) in a quartz tube. *Note: The reaction was set up in three parallel batches (33.3 mg and 4 mL n-hexane each) and irradiated at the same time.* After three hours, the reaction mixture was concentrated and the residue was purified by flash column chromatography on silica gel (5% ethyl acetate in cyclohexane) to yield ketone **32** (49.0 mg, 99.0 µmol, 49%) as a slightly yellow solid.

TLC (20% ethyl acetate in cyclohexane): R_f = 0.29 (CAM, UV).

mp: (158-159) °C

^{1}H NMR (700 MHz, chloroform-*d*) δ 11.53 (s, 1H), 7.16 (d, *J* = 3.0 Hz, 1H), 7.13 (dd, *J* = 9.0, 3.0 Hz, 1H), 6.94 (d, *J* = 9.1 Hz, 1H), 4.87 (d, *J* = 8.8 Hz, 1H), 4.55 (dd, *J* = 10.0, 6.6 Hz, 1H), 4.20 (d, *J* = 8.8 Hz, 1H), 4.13 (s, 1H), 3.81 (s, 3H), 3.72 (d, *J* = 18.6 Hz, 1H), 3.49 (d, *J* = 18.6 Hz, 1H), 3.38 (s, 3H), 3.25 (s, 3H), 2.05 (ddd, *J* = 12.6, 6.6, 1.7 Hz, 1H), 1.95 (ddd, *J* = 12.7, 6.3, 1.7 Hz, 1H), 1.70 (td, *J* = 12.8, 6.2 Hz, 1H), 1.45 – 1.40 (m, 1H), 0.90 (s, 9H), 0.07 – 0.06 (m, 6H).

^{13}C NMR (176 MHz, chloroform-*d*) δ 203.8, 182.6, 156.9, 151.9, 124.5, 119.7, 118.5, 112.6, 108.9, 75.2, 68.3, 59.4, 57.8, 57.3, 56.3, 52.7, 43.4, 35.3, 32.7, 25.9, 18.2, –4.3, –5.0.

IR (Diamond-ATR, neat) $\tilde{\nu}_{max}$: 2955 (m), 2849 (w), 1759 (s), 1676 (w), 1500 (s), 1393 (w), 1258 (m), 1185 (s), 1032 (s), 837 (m), 720 (w) cm^{-1}.

HRMS (ESI) calc. for $C_{25}H_{38}NaO_8Si$ [M+Na]$^+$: 517.2228 found: 517.2226.

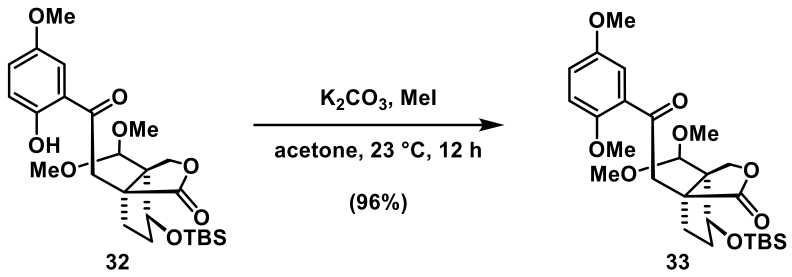

1,4-Dimethylhydroquinone 33

To a solution of ketone **32** (39.0 mg, 79.0 µmol, 1 equiv) in acetone (1 mL) was added potassium carbonate (21.8 mg, 158 µmol, 2.00 equiv) and iodomethane (6.00 µL, 95.0 µmol, 1.20 equiv) in sequence at 23 °C. After 12 hours, the mixture was filtered and the filtrate was concentrated. The residue was purified by flash column chromatography on silica gel (20% ethyl acetate in cyclohexane) to yield 1,4-dimethylhydroquinone **33** (38.3 mg, 75.0 µmol, 96%) as a slightly yellow solid.

TLC (10% ethyl acetate in cyclohexane): R_f = 0.11 (CAM, UV).

mp: (127-128) °C

^{1}**H NMR** (400 MHz, chloroform-*d*) δ 7.33 (d, *J* = 3.3 Hz, 1H), 7.05 (dd, *J* = 9.0, 3.3 Hz, 1H), 6.92 (d, *J* = 9.0 Hz, 1H), 4.90 (d, *J* = 8.7 Hz, 1H), 4.61 (dd, *J* = 10.0, 6.5 Hz, 1H), 4.28 (d, *J* = 8.7 Hz, 1H), 4.20 (s, 1H), 3.89 (s, 3H), 3.81 – 3.74 (m, 4H), 3.52 (d, *J* = 19.8 Hz, 1H), 3.40 (s, 3H), 3.33 (s, 3H), 1.99 (ddd, *J* = 12.3, 6.5, 1.6 Hz, 1H), 1.89 (dtd, *J* = 12.4, 6.2, 1.5 Hz, 1H), 1.61 (ddd, *J* = 13.4, 12.5, 5.9 Hz, 1H), 1.42 – 1.32 (m, 1H), 0.89 (d, *J* = 3.2 Hz, 9H), 0.06 (s, 6H).

^{13}**C NMR** (101 MHz, chloroform-*d*) δ 199.9, 183.2, 153.7, 127.0, 121.5, 113.8, 113.4, 109.4, 74.7, 68.5, 59.4, 57.3, 57.1, 56.2, 56.0, 53.6, 49.5, 35.2, 32.9, 25.9, 18.2, –4.4, –5.0.

IR (Diamond-ATR, neat) $\tilde{\nu}_{max}$: 2955 (m), 2929 (m), 2855 (w), 1764 (s), 1670 (w), 1496 (s), 1465 (m), 1278 (m), 1255 (m), 1152 (s), 1028 (s), 905 (w), 837 (m), 721 (w) cm^{-1}.

HRMS (ESI) calc. for $C_{26}H_{40}NaO_8Si$ $[M+Na]^+$: 531.2385 found: 531.2378.

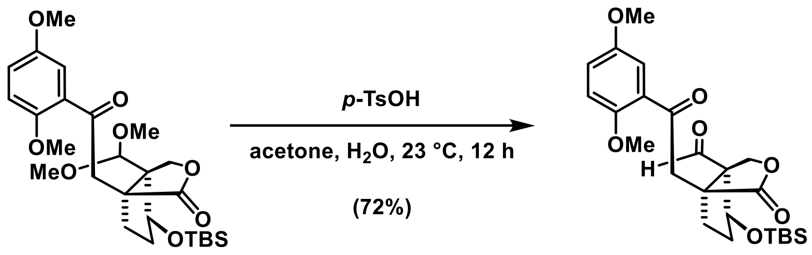

33 34

Aldehyde 34

To a solution of 1,4-dimethylhydroquinone **33** (38.0 mg, 75.0 µmol, 1 equiv) in acetone (1 mL) was added *p*-toluenesulfonic acid monohydrate (1.42 mg, 7.00 µmol, 0.100 equiv) at 23 °C. After 12 hours, the reaction mixture was concentrated and the residue was purified by flash column chromatography (10% ethyl acetate in cyclohexane) to yield aldehyde **34** (24.9 mg, 54.0 µmol, 72%) as slightly yellow oil.

TLC (20% ethyl acetate in cyclohexane): R_f = 0.50 (CAM, UV).

^{1}H NMR (400 MHz, chloroform-*d*) δ 9.59 (s, 1H), 7.28 (d, *J* = 3.3 Hz, 1H), 7.05 (dd, *J* = 9.0, 3.3 Hz, 1H), 6.89 (d, *J* = 9.1 Hz, 1H), 5.04 – 4.97 (m, 2H), 4.62 (dd, *J* = 11.1, 5.9 Hz, 1H), 3.87 (s, 3H), 3.76 (s, 3H), 3.72 (d, *J* = 19.6 Hz, 1H), 3.44 (d, *J* = 19.6 Hz, 1H), 2.12 (dd, *J* = 13.2, 6.6 Hz, 1H), 1.94 (dt, *J* = 11.9, 5.8 Hz, 1H), 1.78 (dd, *J* = 13.4, 5.9 Hz, 1H), 1.60 (m, 1H), 0.85 (s, 9H), 0.01 (m, 6H).

^{13}C NMR (101 MHz, chloroform-*d*) δ 203.5, 199.4, 181.3, 154.4, 153.6, 125.7, 122.5, 113.5, 113.3, 78.8, 67.5, 63.7, 56.1, 55.9, 54.7, 49.6, 34.8, 31.9, 25.7, 18.0, –4.5, –5.0.

IR (Diamond-ATR, neat) $\tilde{\upsilon}_{max}$: 2954 (m), 2930 (m), 2857 (w), 1765 (s), 1720 (m), 1661 (w), 1496 (s), 1464 (m), 1254 (m), 1154 (s), 1133 (s), 1035 (m), 838 (m), 779 (m), 725 (w) cm^{-1}.

HRMS (ESI) calc. for $C_{24}H_{34}NaO_7Si$ $[M+Na]^+$: 485.1966 found: 485.1956.

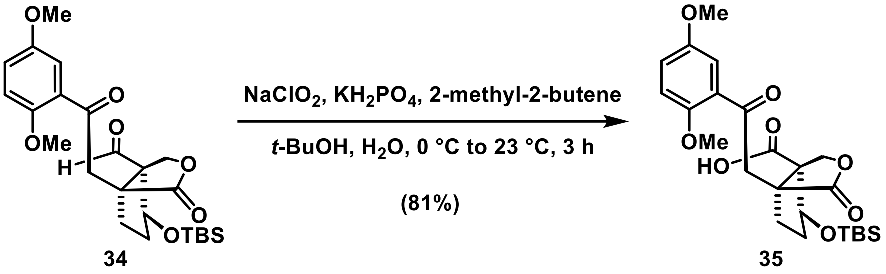

Acid 35

To a solution of aldehyde **34** (24.0 mg, 52.0 µmol, 1 equiv) and potassium dihydrogen phosphate (14.1 mg, 104 µmol, 2.00 equiv) in *tert*-butanol (600 µL), 2-methyl-2-butene (200 µL) and water (200 µL) was added sodium chlorite (17.6 mg, 156 µmol, 3.00 equiv) at 0 °C. After one hour, the mixture was allowed to warm to 23 °C. After two hours at 23 °C, a saturated aqueous solution of ammonium chloride (5 mL) and ethyl acetate (5 mL) were added to the reaction mixture and the layers were separated. The aqueous layer was extracted with ethyl acetate (3 × 2 mL) and the combined organic layers were dried over sodium sulfate. The dried solution was filtered and the filtrate was concentrated. The residue was purified by flash column chromatography on silica gel (30% ethyl acetate in hexane) to yield acid **35** (20.0 mg, 42.0 µmol, 81%) as a colourless solid.

TLC (30% ethyl acetate in cyclohexane): R_f = 0.14 (CAM, UV).

mp: (207-208) °C

^{1}H NMR (400 MHz, chloroform-*d*) δ 7.27 (d, *J* = 3.3 Hz, 1H), 7.01 (dd, *J* = 9.0, 3.3 Hz, 1H), 6.87 (d, *J* = 9.0 Hz, 1H), 4.97 (d, *J* = 9.7 Hz, 1H), 4.83 (d, *J* = 9.7 Hz, 1H), 4.43 (dd, *J* = 11.0, 5.7 Hz, 1H), 3.92 (d, *J* = 19.5 Hz, 1H), 3.87 (s, 3H), 3.74 (s, 3H), 3.66 (d, *J* = 19.5 Hz, 1H), 2.10 (dd, *J* = 12.7, 6.3 Hz, 1H), 1.92 – 1.85 (m, 1H), 1.79 (dd, *J* = 13.3, 5.7 Hz, 1H), 1.52 (dtd, *J* = 12.7, 11.3, 10.9, 6.1 Hz, 1H), 0.77 (s, 9H), –0.15 (m, 6H).

^{13}C NMR (101 MHz, chloroform-*d*) δ 199.9, 181.4, 154.3, 153.5, 126.2, 121.9, 113.8, 113.3, 81.1, 66.9, 56.2, 55.9, 54.4, 50.1, 34.9, 31.9, 25.9, 25.6, 17.9, –5.0, –5.3.

IR (Diamond-ATR, neat) $\tilde{\nu}_{max}$: 3000 (br, w), 2930 (m), 2857 (m), 1767 (s), 1741 (s), 1702 (s), 1496 (s), 1465 (m), 1278 (s), 1223 (s), 1166 (s), 1127 (s), 1029 (m), 839 (s), 779 (m) cm^{-1}.

HRMS (ESI) calc. for $C_{24}H_{33}O_8Si$ $[M–H]^-$: 477.1950 found: 477.1941.

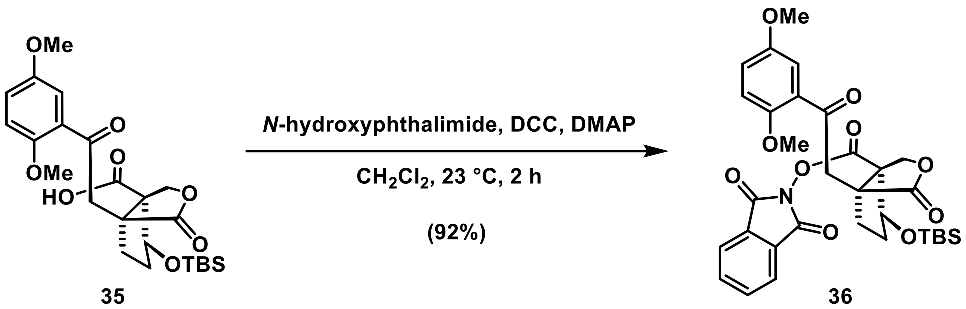

N-(Acyloxy)phthalimide 36

To a solution of acid **35** (20.0 mg, 42.0 µmol, 1 equiv) in dichloromethane (200 µL) was added 4-(dimethylamino)pyridine (0.500 mg, 4.18 µmol, 0.100 equiv), *N*-hydroxyphthalimide (9.14 mg, 54.0 µmol, 1.30 equiv) and a solution of *N,N*'-dicyclohexylcarbodiimide (9.58 mg, 46.0 µmol, 1.10 equiv) in dichloromethane (100 µL) in sequence at 23 °C. After two hours, the mixture was diluted with diethyl ether (2 mL) and filtered through a pad of Celite, the filter cake was washed with diethyl ether (2 mL) and the filtrated was concentrated. The residue was purified by flash column chromatography on silica gel (30% ethyl acetate in hexane) to yield *N*-(acyloxy)phthalimide **36** (24.0 mg, 39.0 µmol, 92%) as a colourless solid.

TLC (30% ethyl acetate in cyclohexane): R_f = 0.17 (CAM, UV).

mp: (177-178) °C

^{1}H NMR (400 MHz, chloroform-*d*) δ 7.80 – 7.77 (m, 2H), 7.73 (dd, *J* = 5.5, 3.1 Hz, 2H), 7.46 (d, *J* = 3.3 Hz, 1H), 7.04 (dd, *J* = 9.0, 3.3 Hz, 1H), 6.89 (d, *J* = 9.0 Hz, 1H), 5.19 – 5.10 (m, 2H), 4.78 (dd, *J* = 10.5, 5.9 Hz, 1H), 4.00 – 3.88 (m, 2H), 3.87 (s, 3H), 3.82 (s, 3H), 2.28 – 2.20 (m, 1H), 2.10 – 2.02 (m, 1H), 1.94 (dd, *J* = 13.3, 5.9 Hz, 1H), 1.63 – 1.59 (m, 1H), 0.93 (s, 9H), 0.19 – 0.16 (m, 6H).

^{13}C NMR (101 MHz, chloroform-*d*) δ 198.6, 180.8, 169.3, 161.3, 154.4, 153.5, 134.9, 129.0, 126.3, 124.1, 122.3, 113.8, 113.3, 80.6, 66.5, 59.3, 56.2, 56.0, 55.3, 50.6, 35.2, 32.9, 25.9, 18.1, –4.5, –5.1.

IR (Diamond-ATR, neat) $\tilde{\upsilon}_{max}$: 2929 (w), 2856 (w), 1769 (m), 1746 (s), 1496 (w), 1184 (m), 1048 (m), 877 (m), 696 (m) cm^{-1}.

HRMS (ESI) calc. for $C_{32}H_{37}NNaO_{10}Si$ [M+Na]$^+$: 646.2079 found: 646.2067.

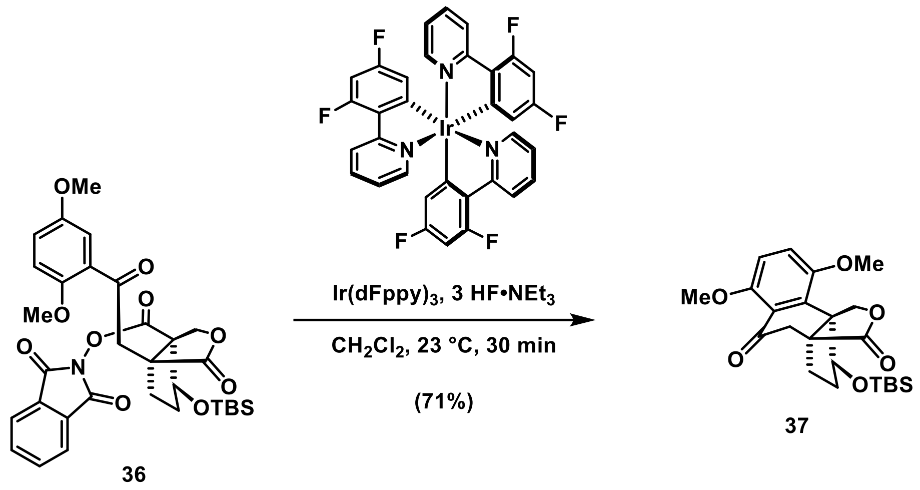

Tetralone 37

Reaction setup: The reaction was carried out in the Rayonet RPR-200 Photochemical Reactor, with the reaction mixture placed in the center of the 25 cm wide reaction chamber (approximately 12 cm away from the light source), above a cooling fan and surrounded by a circular array of 16 light tubes.

A solution of *N*-(acyloxy)phthalimide **36** (5.90 mg, 9.46 µmol, 1 equiv), tris[2-(2,4-difluorophenyl)pyridine]iridium(III) (0.700 mg, 0.946 µmol, 0.100 equiv) and hydrogen fluoride triethylamine (1.50 µL, 0.946 µmol, 0.100 equiv) in degassed dichloromethane (300 µL) was irradiated at 419 nm (Southern New England Ultraviolet Company, RPR-4190A° lamps) at 23 °C. After 30 minutes, the mixture was concentrated and the residue was purified by flash column chromatography on silica gel (20% grading to 30% ethyl acetate in hexane) to yield tetralone **37** (2.90 mg, 6.70 µmol, 71%) as a yellow solid.

TLC (30% ethyl acetate in cyclohexane): R_f = 0.17 (CAM, UV).

mp: (192-193) °C

^{1}H NMR (600 MHz, chloroform-*d*) δ 7.06 (d, *J* = 9.1 Hz, 1H), 6.89 (d, *J* = 9.1 Hz, 1H), 5.07 (d, *J* = 9.7 Hz, 1H), 4.39 (d, *J* = 2.7 Hz, 1H), 4.24 (d, *J* = 9.7 Hz, 1H), 3.86 – 3.83 (m, 6H), 2.87 – 2.77 (m, 2H), 2.57 – 2.50 (m, 1H), 1.91 (dd, *J* = 13.8, 9.2 Hz, 1H), 1.62 (dd, *J* = 13.4, 7.8 Hz, 1H), 1.40 (dddd, *J* = 13.5, 12.1, 9.4, 2.9 Hz, 1H), 0.92 (s, 9H), 0.18 – 0.05 (m, 6H).

^{13}C NMR (151 MHz, chloroform-*d*) δ 195.2, 180.0, 152.3, 150.2, 131.7, 123.7, 116.7, 112.0, 80.3, 71.7, 57.5, 56.6, 55.8, 52.2, 45.3, 34.3, 32.1, 25.7, 18.0, –4.4, –4.9.

IR (Diamond-ATR, neat) $\tilde{\nu}_{max}$: 2927 (s), 2854 (m), 1771 (m), 1701 (m), 1464 (m), 1272 (m), 1181 (w), 1107 (m), 1018 (m), 834 (m), 777 (w) cm^{-1}.

HRMS (ESI) calc. for $C_{23}H_{32}NaO_6Si$ $[M+Na]^+$: 455.1860 found: 455.1855.

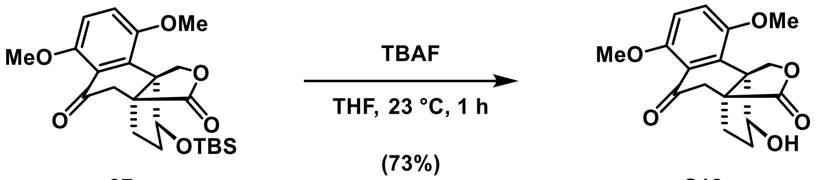

37 S18

Alcohol S18

To a solution of tetralone **37** (8.60 mg, 20.0 µmol, 1 equiv) in tetrahydrofuran (200 µL) was added a solution of tetrabutylammonium fluoride (1.00 M in tetrahydrofuran, 9.94 µL, 9.94 µmol, 5.00 equiv) at 23 °C. After one hour, a saturated aqueous solution of sodium bicarbonate (5 mL) and ethyl acetate (5 mL) were added to the reaction mixture and the layers were separated. The aqueous layer was extracted with ethyl acetate (3 × 2 mL) and the combined organic layers were dried over sodium sulfate. The dried solution was filtered and the filtrate was concentrated. The residue was purified by flash column chromatography on silica gel (30% ethyl acetate in cyclohexane to 5% methanol in dichloromethane) to yield alcohol **S18** (4.60 mg, 15.0 µmol, 73%) as a colourless oil.

TLC (5% methanol in dichloromethane): R_f = 0.31 (CAM, UV).

^{1}H NMR (600 MHz, chloroform-*d*) δ 7.10 (d, *J* = 9.1 Hz, 1H), 6.89 (d, *J* = 9.2 Hz, 1H), 5.31 (d, *J* = 9.9 Hz, 1H), 4.26 (d, *J* = 9.9 Hz, 1H), 4.23 (dd, *J* = 7.5, 5.8 Hz, 1H), 3.88 (s, 3H), 3.85 (s, 3H), 2.87 (d, *J* = 13.1 Hz, 1H), 2.80 (d, *J* = 13.1 Hz, 1H), 2.68 (s, 1H), 2.38 (ddd, *J* = 13.5, 7.0, 4.3 Hz, 1H), 1.93 (dddd, *J* = 13.1, 7.1, 5.7, 4.2 Hz, 1H), 1.79 (ddd, *J* = 13.4, 10.0, 7.1 Hz, 1H), 1.58 – 1.51 (m, 1H).

^{13}C NMR (151 MHz, chloroform-*d*) δ 194.9, 179.7, 152.7, 150.3, 134.2, 122.6, 117.1, 111.7, 81.8, 70.3, 56.7, 56.5, 53.6, 53.6, 44.3, 32.4, 31.6.

IR (Diamond-ATR, neat) $\tilde{\upsilon}_{max}$: 3476 (br, w), 2925 (m), 2853 (w), 1747 (s), 1694 (m), 1585 (w), 1476 (m), 1270 (s), 1180 (m), 1107 (m), 1013 (s), 814 (w) cm^{-1}.

HRMS (ESI) calc. for $C_{23}H_{32}NaO_6Si$ $[M+Na]^+$: 455.1860 found: 455.1855.

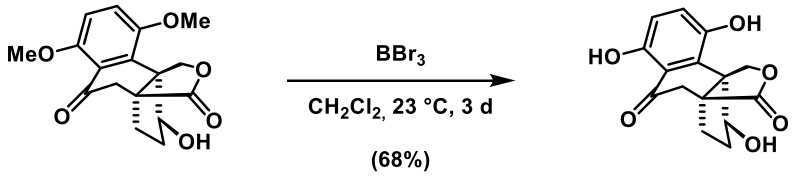

S18 lingzhiol (5)

Lingzhiol (5)

To a solution of alcohol **S18** (4.10 mg, 13.0 µmol, 1 equiv) in dichloromethane (0.5 mL) was added a solution of boron tribromide (1.00 M in dichloromethane, 64.0 µL, 64.0 µmol, 5.00 equiv) at 23 °C upon which the solution turned quickly dark brown. After three days, a saturated aqueous solution of sodium bicarbonate (5 mL) and dichloromethane (5 mL) were added to the reaction mixture and the layers were separated. The aqueous layer was extracted with dichloromethane (3 × 2 mL) and the combined organic layers were dried over sodium sulfate. The dried solution was filtered and the filtrate was concentrated. The residue was purified by flash column chromatography on silica gel (60% ethyl acetate in cyclohexane) to yield lingzhiol (**5**) (2.55 mg, 8.78 µmol, 68%) as a slightly yellow solid.

TLC (60% ethyl acetate in cyclohexane): R_f = 0.14 (CAM, UV).

^{1}H NMR (700 MHz, acetone-d_6) δ 7.22 (d, J = 8.9 Hz, 1H), 6.77 (d, J = 8.9 Hz, 1H), 5.22 (d, J = 9.6 Hz, 1H), 4.63 (m, 1H), 4.45 (d, J = 9.6 Hz, 1H), 3.09 (d, J = 16.0 Hz, 1H), 2.81 – 2.78 (m, 1H), 2.47 – 2.44 (m, 1H), 1.85 – 1.82 (m, 1H), 1.80 – 1.76 (m, 1H), 1.72 – 1.69 (m, 1H).

^{13}C NMR (176 MHz, acetone-d_6) δ 202.4, 180.2, 156.5, 148.1, 129.8, 127.7, 118.0, 116.6, 80.8, 71.0, 56.2, 52.6, 42.4, 33.9, 33.4.

IR (Diamond-ATR, neat) $\tilde{\upsilon}_{max}$: 3298 (br, w), 2926 (m), 2856 (w), 1768 (m), 1650 (m), 1465 (s), 1332 (m), 1220 (m), 1181 (m), 1103 (w), 1014 (w) cm^{-1}.

HRMS (ESI) calc. for $C_{15}H_{14}NaO_6$ $(M+Na)^+$: 313.0683 found: 313.0680.

Table 13: Comparison of ^{1}H-NMR shifts for natural[15] and synthetic lingzhiol (**5**).

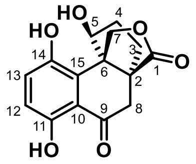

lingzhiol (5)

No	^{1}H-NMR (400 MHz, acetone-d_6) isolated lingzhiol **ppm**	^{1}H-NMR (700 MHz, acetone-d_6) synthetic lingzhiol **ppm**	Δ ppm
1	-	-	-
2	-	-	-
3	2.44 (m)	2.47 – 2.44 (m)	+0.02
	1.78 (m)	1.80 – 1.76 (m)	±0
4	1.83 (m)	1.85 – 1.82 (m)	+0.01
	1.70 (m)	1.72 – 1.69 (m)	+0.01
5	4.63 (t, *J* = 4.5 Hz)	4.63 (m)	±0
6	-	-	-
7	5.22 (d, *J* = 9.6 Hz)	5.22 (d, *J* = 9.6 Hz)	±0
	4.45 (d, *J* = 9.6 Hz)	4.45 (d, *J* = 9.6 Hz)	±0
8	3.09 (d, *J* = 16.0 Hz)	3.09 (d, *J* = 16.0 Hz)	±0
	2.79 (d, *J* = 16.0 Hz)	2.81 – 2.78 (m, overlap)	±0
9	-	-	-
10	-	-	-
11	-	-	-
12	6.77 (d, *J* = 8.9 Hz)	6.77 (d, *J* = 8.9 Hz)	±0
13	7.22 (d, *J* = 8.9 Hz)	7.22 (d, *J* = 8.9 Hz)	±0
14	-	-	-
15	-	-	-

[15] Y.-M. Yan, J. Ai, L. L. Zhou, A. C. K. Chung, R. Li, J. Nie, P. Fang, X.-L. Wang, J. Luo, Q. Hu, F.-F. Hou, Y.-X. Cheng, *Org. Lett.* **2013**, *15*, 5488–5491.

Table 14: Comparison of ^{13}C-NMR shifts for natural and synthetic lingzhiol (**5**).

No	^{13}C-NMR (100 MHz, acetone-d_6) isolated lingzhiol **ppm**	^{13}C-NMR (176 MHz, acetone-d_6) synthetic lingzhiol **ppm**	Δ ppm
1	180.1	180.2	+0.1
2	52.5	52.6	+0.1
3	33.3	33.4	+0.1
4	33.7	33.9	+0.2
5	80.6	80.8	+0.2
6	56.1	56.2	+0.1
7	70.9	71.0	+0.1
8	42.3	42.4	+0.1
9	202.3	202.4	+0.1
10	116.4	116.6	+0.2
11	156.3	156.5	+0.2
12	117.9	118.0	+0.1
13	127.5	127.7	+0.2
14	147.9	148.1 (low intensity)	+0.2
15	129.1	129.8 (low intensity)	+0.7

3. NMR Spectra

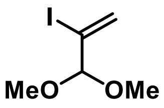

S2

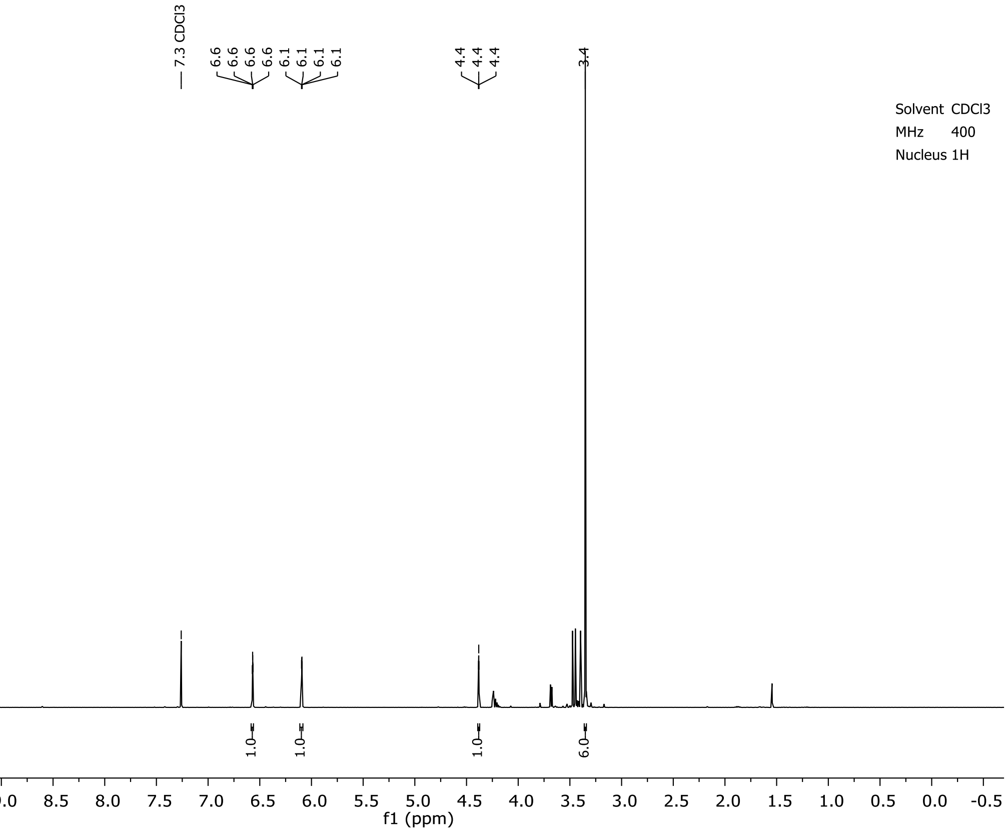

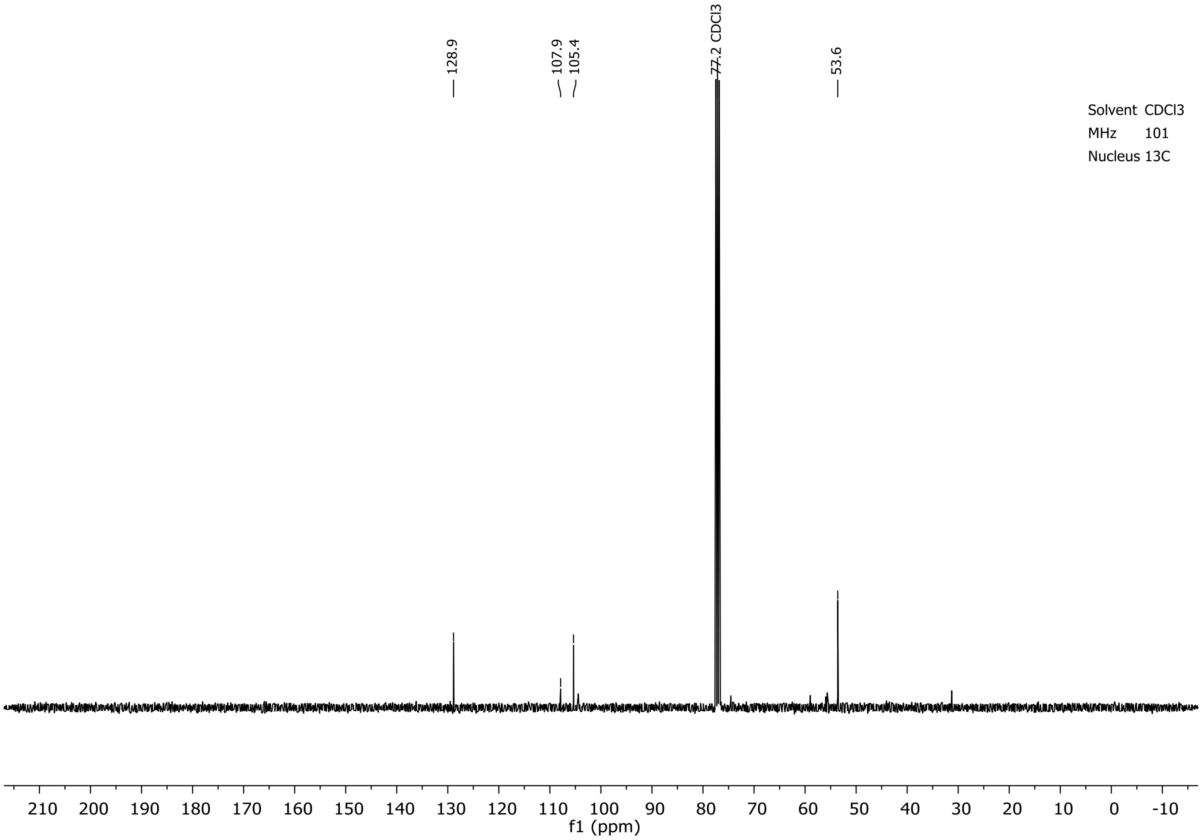

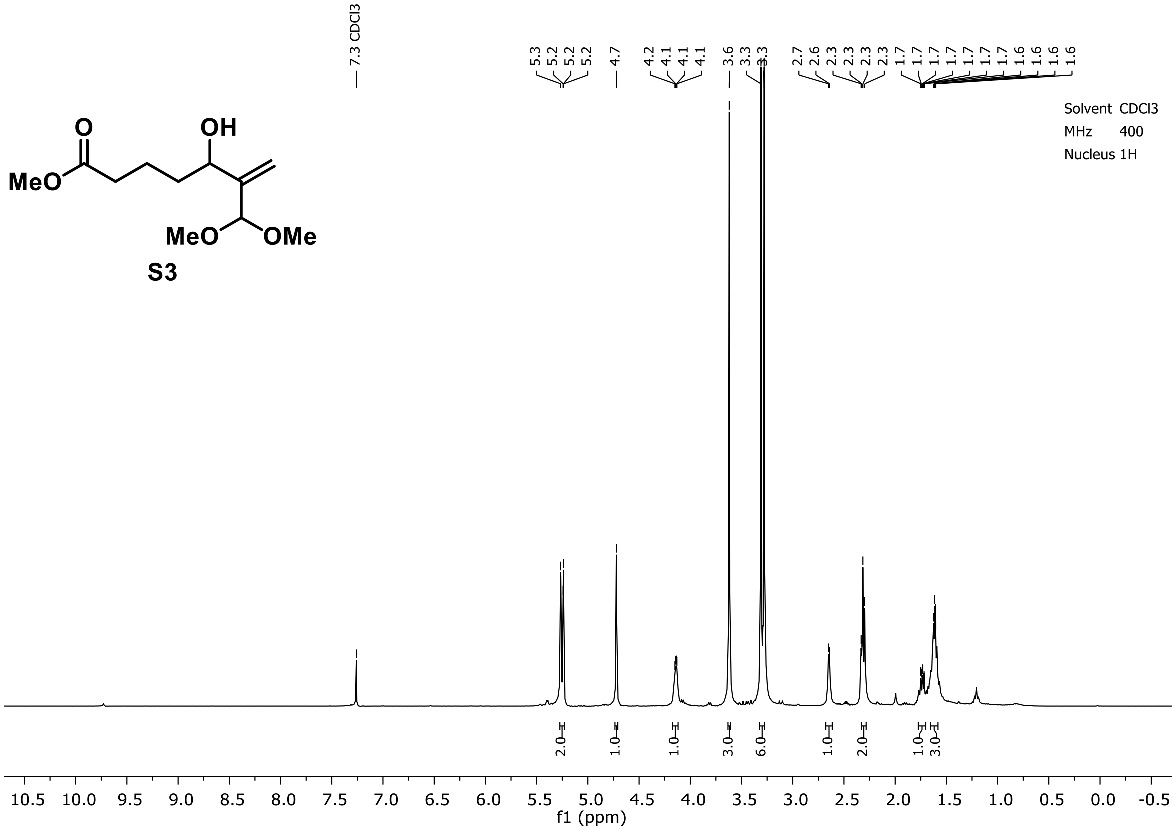
O
OH
MeO
MeO
OMe
S3
7.3 CDCl3
5.3 5.2 5.2 5.2
4.7
4.2 4.1 4.1 4.1
3.6 3.3 3.3
2.7 2.6 2.3 2.3 2.3 2.3
1.7 1.7 1.7 1.7 1.7 1.7 1.7 1.6 1.6 1.6
Solvent CDCl3
MHz 400
Nucleus 1H
2.0
1.0
1.0
3.0
6.0
1.0
2.0
1.0
3.0
10.5 10.0 9.5 9.0 8.5 8.0 7.5 7.0 6.5 6.0 5.5 5.0 4.5 4.0 3.5 3.0 2.5 2.0 1.5 1.0 0.5 0.0 -0.5
f1 (ppm)

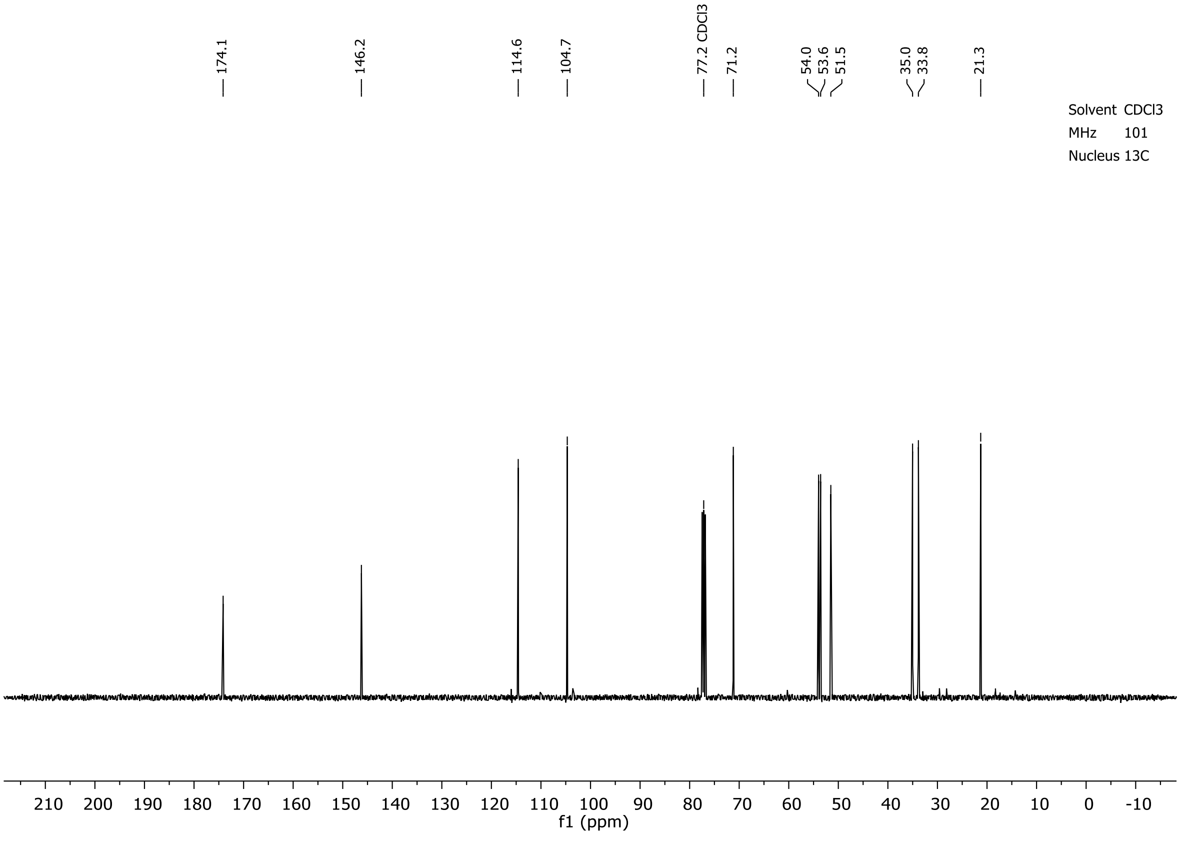
174.1
146.2
114.6
104.7
77.2 CDCl3
71.2
54.0 53.6 51.5
35.0 33.8
21.3
Solvent CDCl3
MHz 101
Nucleus 13C
210 200 190 180 170 160 150 140 130 120 110 100 90 80 70 60 50 40 30 20 10 0 -10
f1 (ppm)

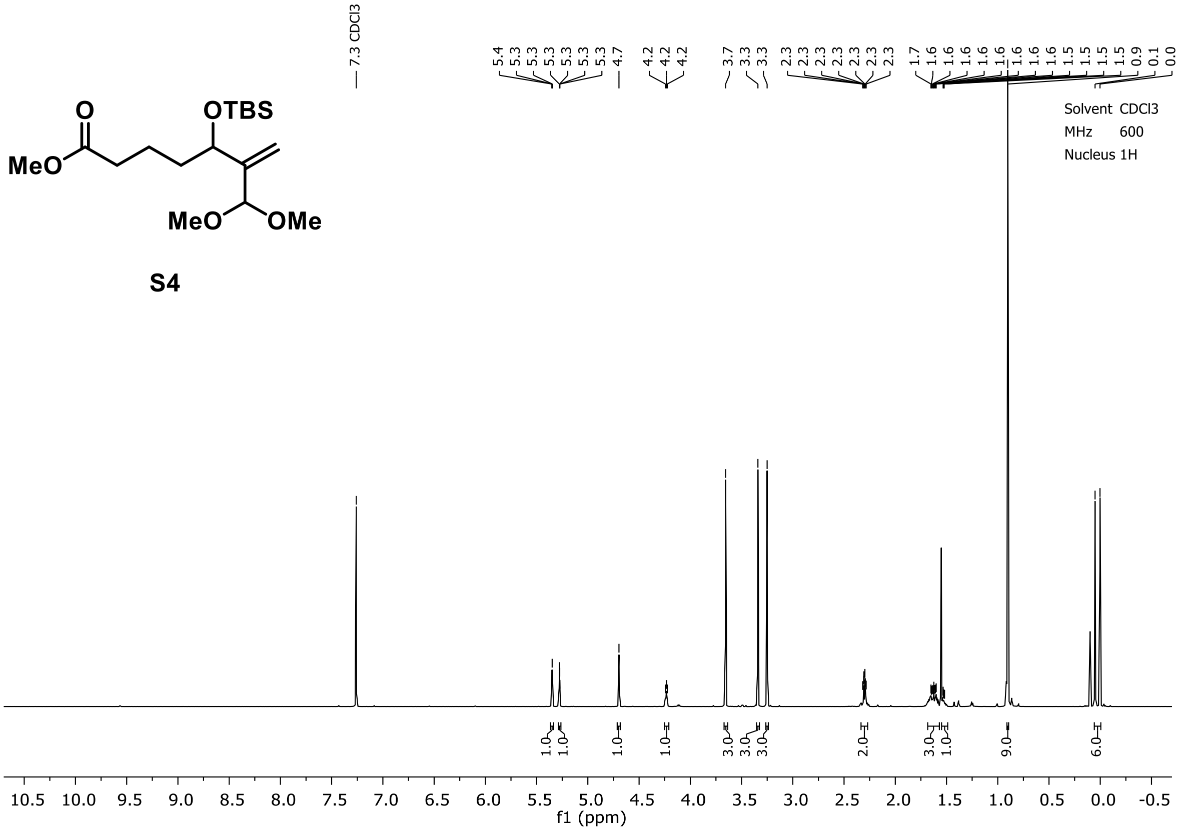
O
OTBS
MeO
MeO
OMe
S4
7.3 CDCl3
5.4
5.3
5.3
5.3
5.3
5.3
5.3
4.7
4.2
4.2
4.2
3.7
3.3
3.3
2.3
2.3
2.3
2.3
2.3
2.3
2.3
1.7
1.6
1.6
1.6
1.6
1.6
1.6
1.6
1.5
1.5
1.5
1.5
0.9
0.1
0.0
Solvent CDCl3
MHz 600
Nucleus 1H
1.0
1.0
1.0
1.0
3.0
3.0
3.0
2.0
3.0
1.0
9.0
6.0
10.5 10.0 9.5 9.0 8.5 8.0 7.5 7.0 6.5 6.0 5.5 5.0 4.5 4.0 3.5 3.0 2.5 2.0 1.5 1.0 0.5 0.0 -0.5
f1 (ppm)

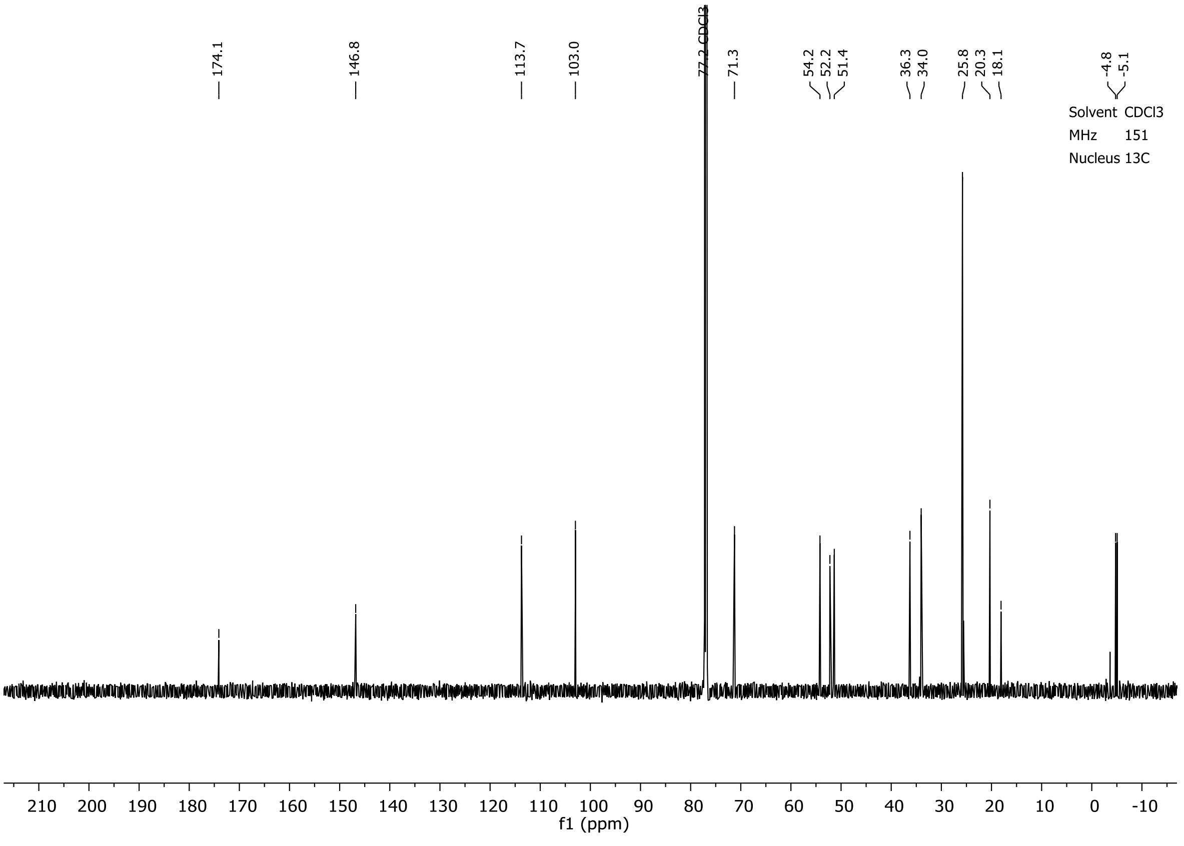
174.1
146.8
113.7
103.0
77.2 CDCl3
71.3
54.2
52.2
51.4
36.3
34.0
25.8
20.3
18.1
-4.8
-5.1
Solvent CDCl3
MHz 151
Nucleus 13C
210 200 190 180 170 160 150 140 130 120 110 100 90 80 70 60 50 40 30 20 10 0 -10
f1 (ppm)

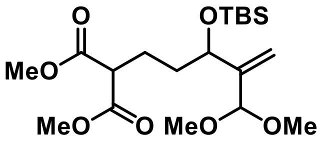
O
OTBS
MeO
MeO
O
MeO
OMe

S5

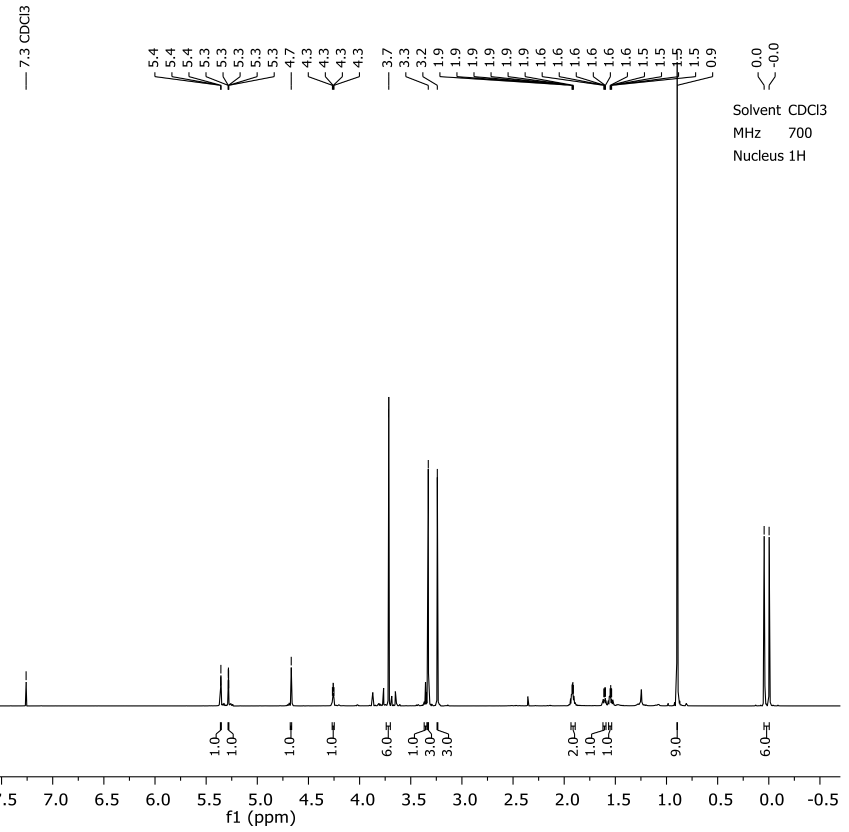
Solvent CDCl3
MHz 700
Nucleus 1H
f1 (ppm)

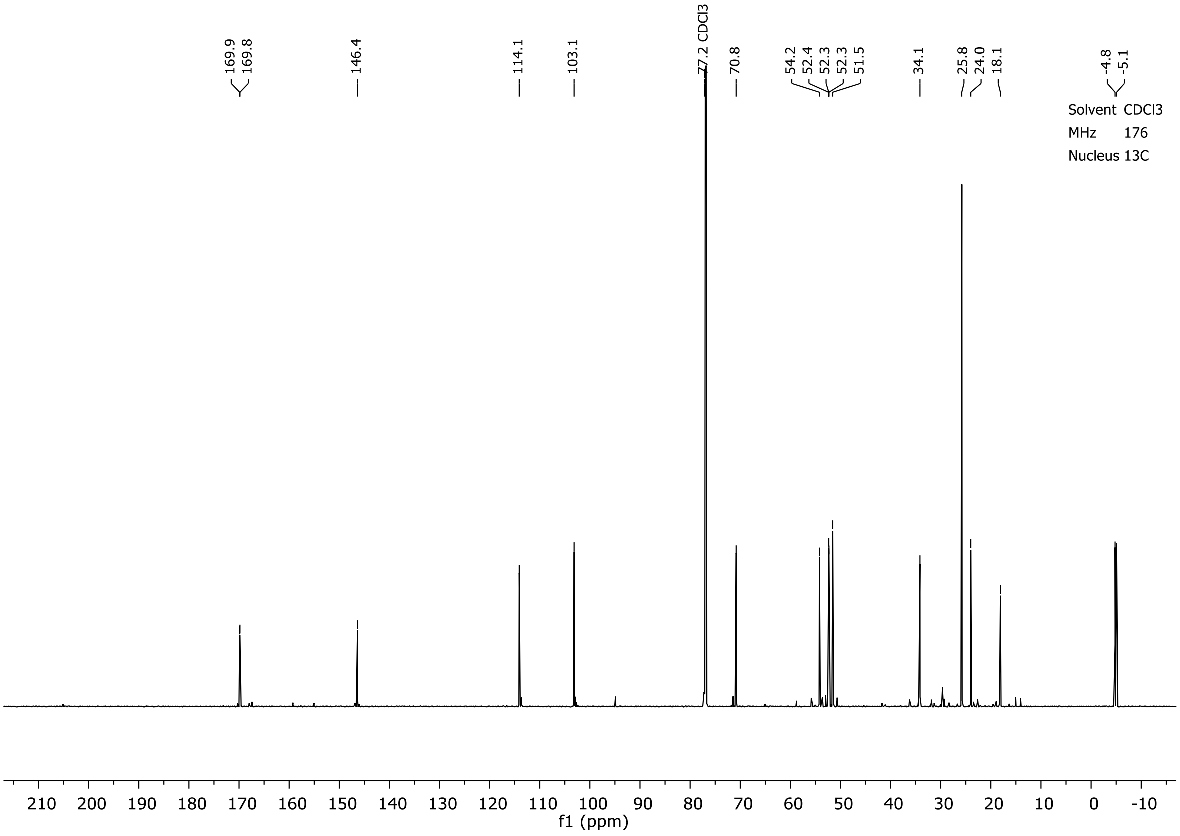
Solvent CDCl3
MHz 176
Nucleus 13C
f1 (ppm)

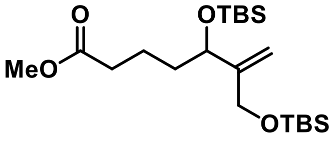
O
OTBS
MeO
OTBS

S10

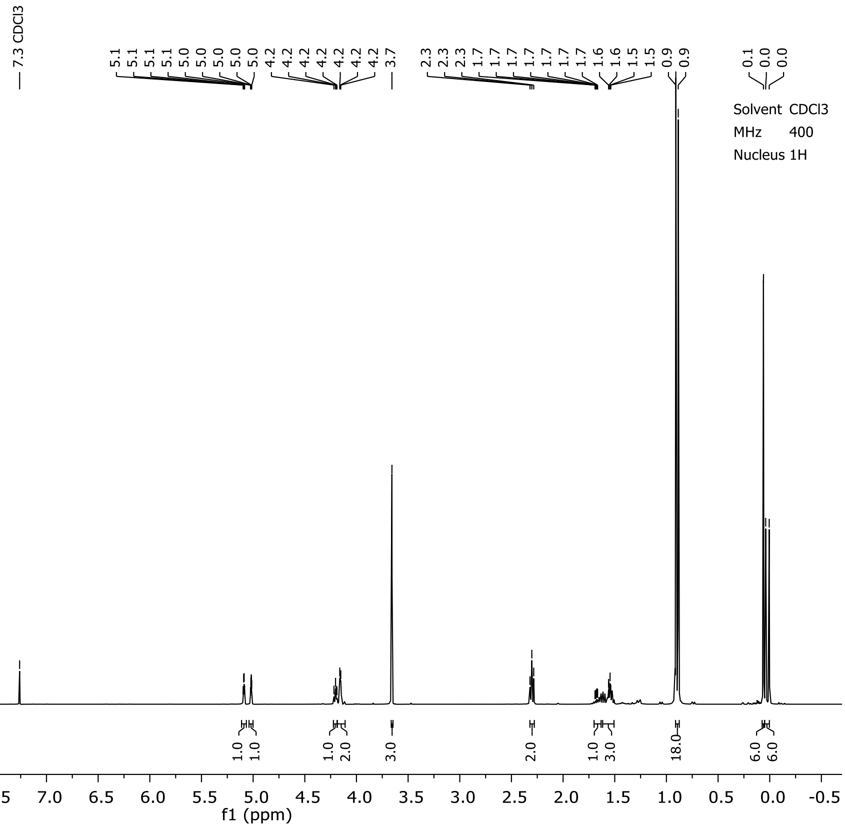
7.3 CDCl3
Solvent CDCl3
MHz 400
Nucleus 1H
f1 (ppm)

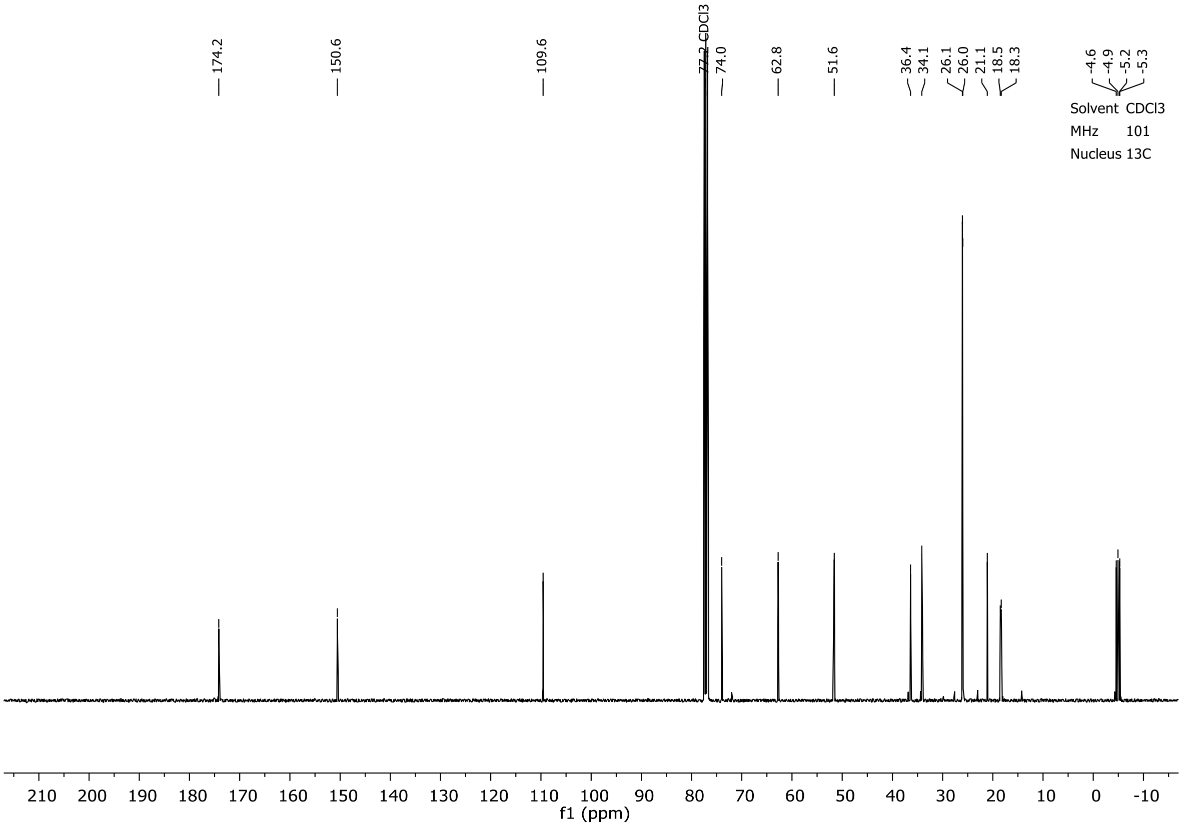
77.2 CDCl3
Solvent CDCl3
MHz 101
Nucleus 13C
f1 (ppm)

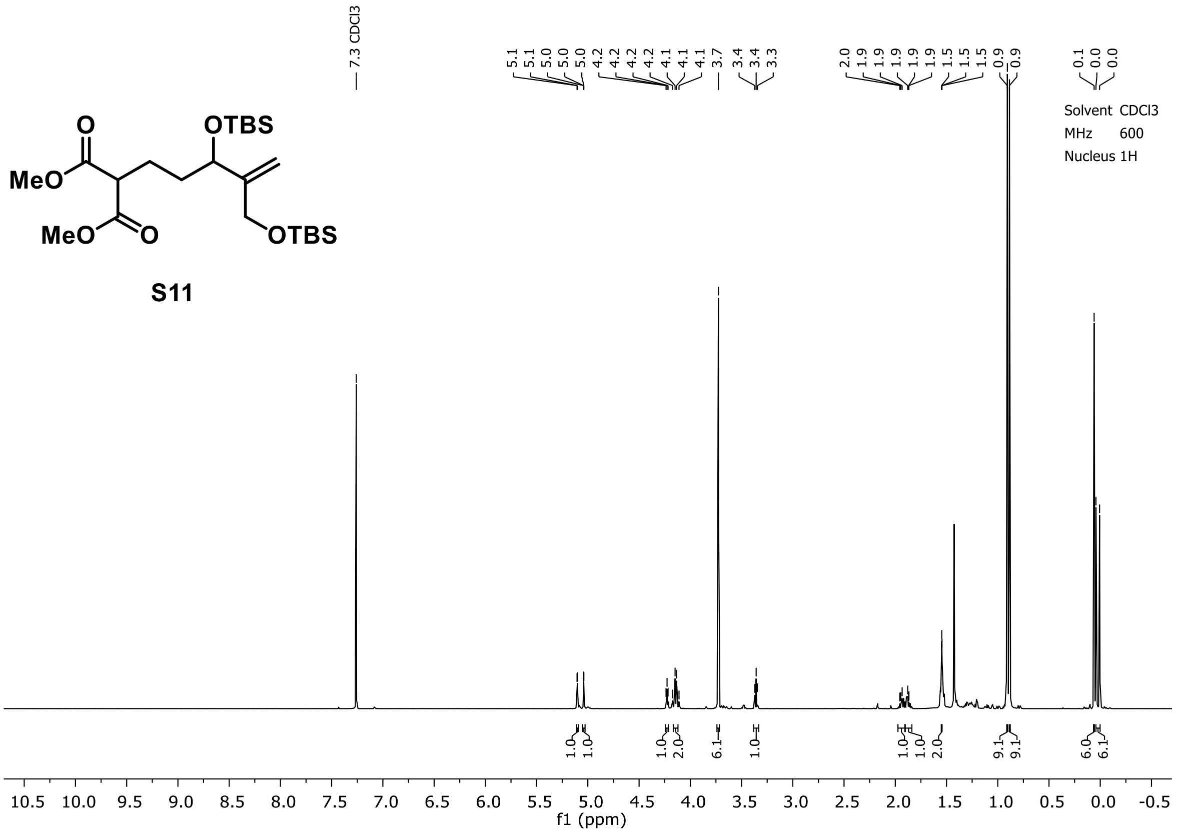
O
OTBS
MeO
MeO
O
OTBS
7.3 CDCl3
Solvent CDCl3
MHz 600
Nucleus 1H
f1 (ppm)

S11

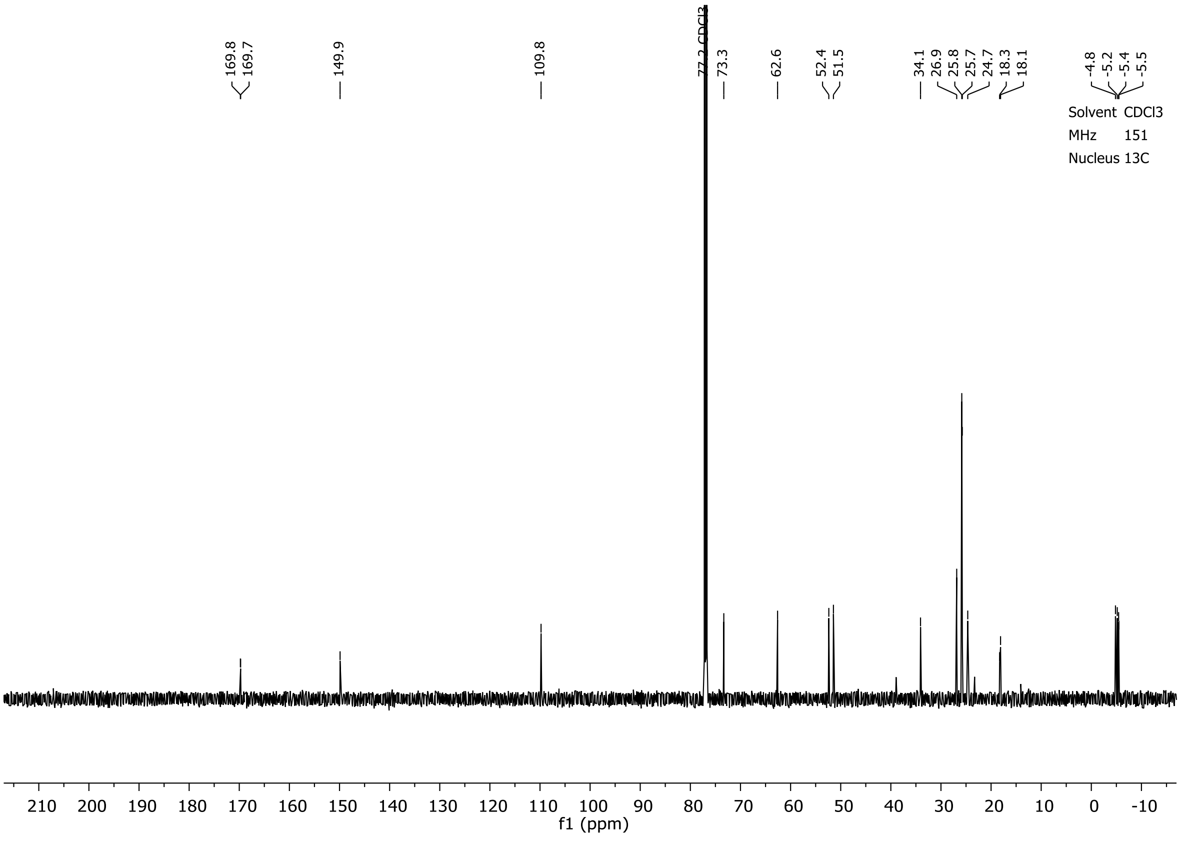
Solvent CDCl3
MHz 151
Nucleus 13C
f1 (ppm)

S12

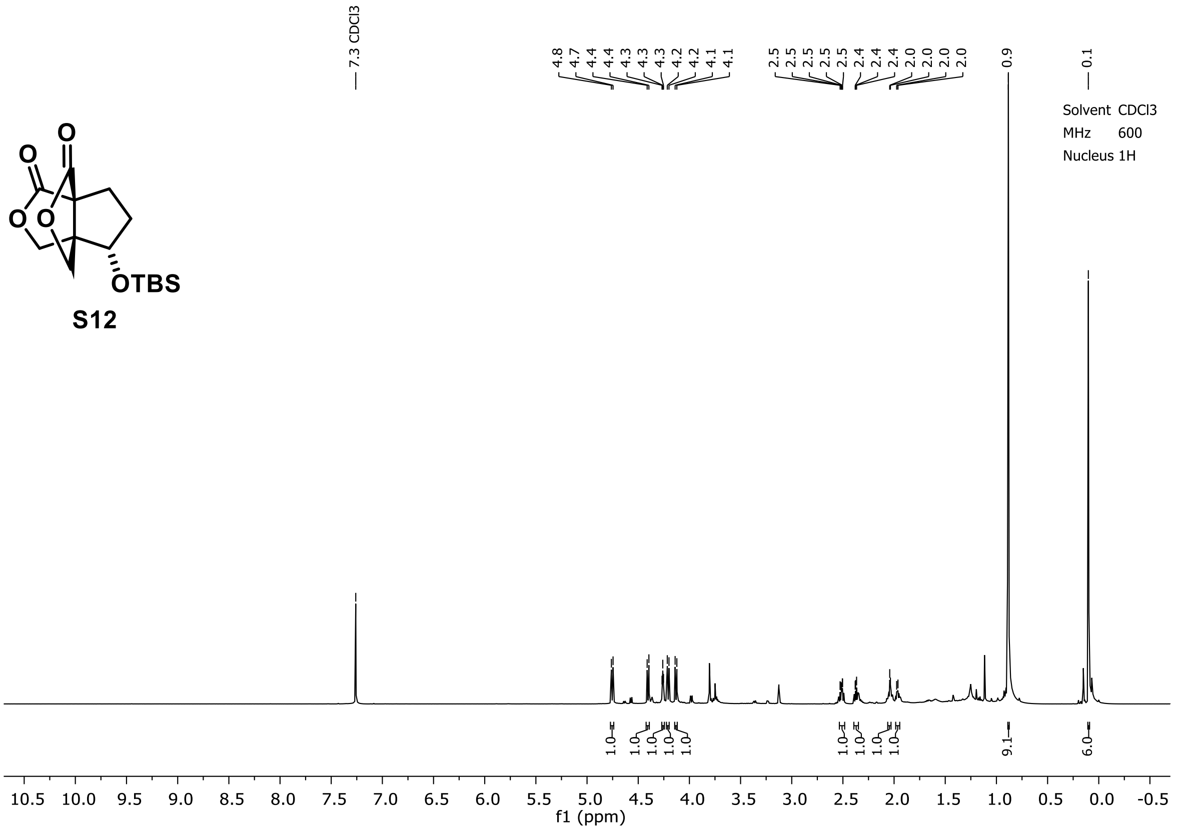
O
O
O O
OTBS
7.3 CDCl3
4.8
4.7
4.4
4.4
4.3
4.3
4.3
4.2
4.2
4.1
4.1
2.5
2.5
2.5
2.5
2.5
2.4
2.4
2.4
2.0
2.0
2.0
2.0
0.9
0.1
Solvent CDCl3
MHz 600
Nucleus 1H
1.0
1.0
1.0
1.0
1.0
1.0
1.0
1.0
1.0
9.1
6.0
10.5 10.0 9.5 9.0 8.5 8.0 7.5 7.0 6.5 6.0 5.5 5.0 4.5 4.0 3.5 3.0 2.5 2.0 1.5 1.0 0.5 0.0 -0.5
f1 (ppm)

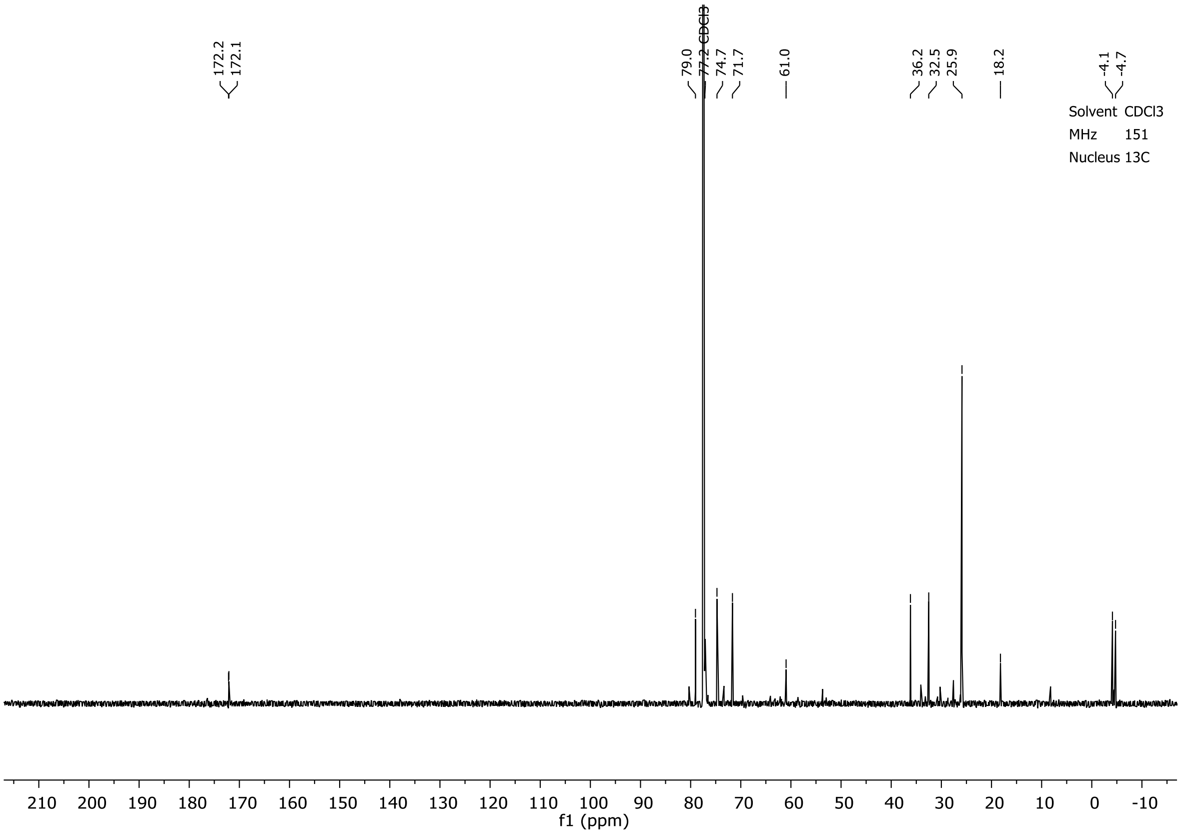
172.2
172.1
79.0
77.2 CDCl3
74.7
71.7
61.0
36.2
32.5
25.9
18.2
-4.1
-4.7
Solvent CDCl3
MHz 151
Nucleus 13C
210 200 190 180 170 160 150 140 130 120 110 100 90 80 70 60 50 40 30 20 10 0 -10
f1 (ppm)

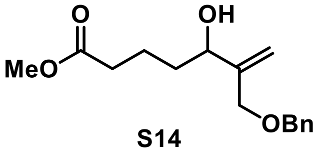
O
OH
MeO
OBn

S14

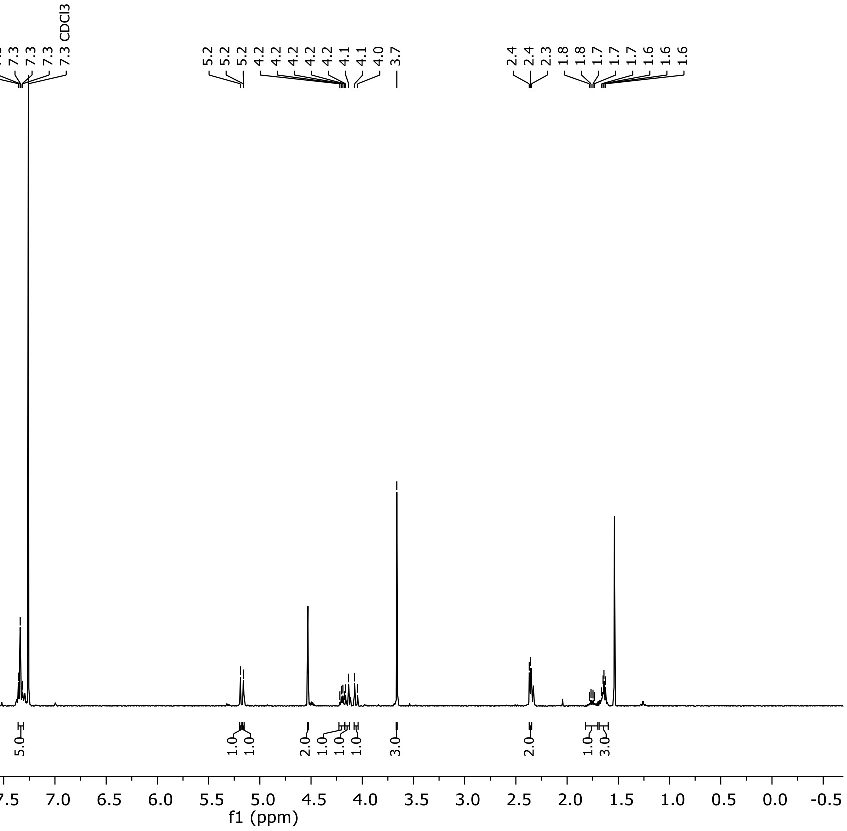
7.4
7.3
7.3
7.3
7.3 CDCl3
5.2
5.2
5.2
4.2
4.2
4.2
4.2
4.2
4.2
4.1
4.1
4.0
3.7
2.4
2.4
2.3
1.8
1.8
1.7
1.7
1.7
1.7
1.6
1.6
1.6
5.0
1.0
1.0
2.0
1.0
1.0
1.0
3.0
2.0
1.0
3.0
10.5 10.0 9.5 9.0 8.5 8.0 7.5 7.0 6.5 6.0 5.5 5.0 4.5 4.0 3.5 3.0 2.5 2.0 1.5 1.0 0.5 0.0 -0.5
f1 (ppm)

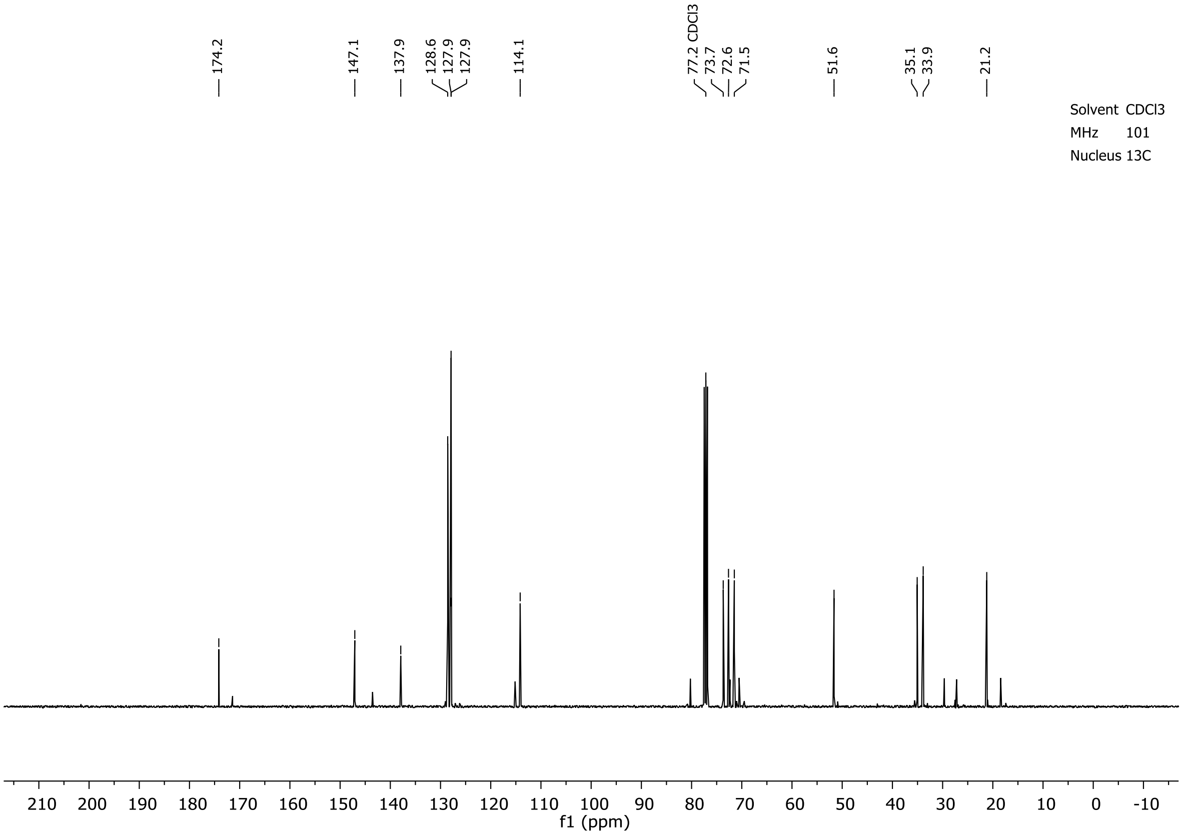
174.2
147.1
137.9
128.6
127.9
127.9
114.1
77.2 CDCl3
73.7
72.6
71.5
51.6
35.1
33.9
21.2
Solvent CDCl3
MHz 101
Nucleus 13C
210 200 190 180 170 160 150 140 130 120 110 100 90 80 70 60 50 40 30 20 10 0 -10
f1 (ppm)

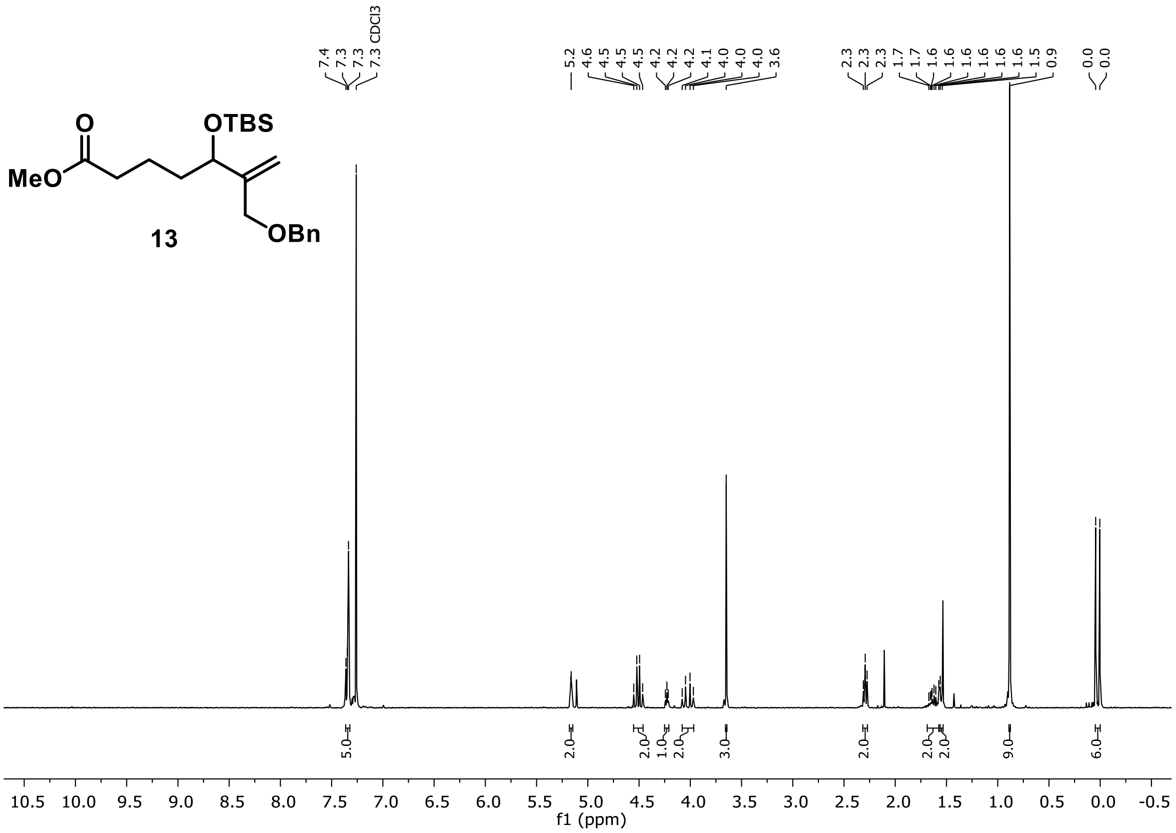
O
OTBS
MeO
13
OBn
7.4
7.3
7.3
7.3 CDCl3
5.2
4.6
4.5
4.5
4.5
4.2
4.2
4.2
4.1
4.0
4.0
4.0
3.6
2.3
2.3
2.3
1.7
1.7
1.6
1.6
1.6
1.6
1.6
1.5
0.9
0.0
0.0
5.0
2.0
2.0
1.0
2.0
3.0
2.0
2.0
2.0
9.0
6.0
10.5 10.0 9.5 9.0 8.5 8.0 7.5 7.0 6.5 6.0 5.5 5.0 4.5 4.0 3.5 3.0 2.5 2.0 1.5 1.0 0.5 0.0 -0.5
f1 (ppm)

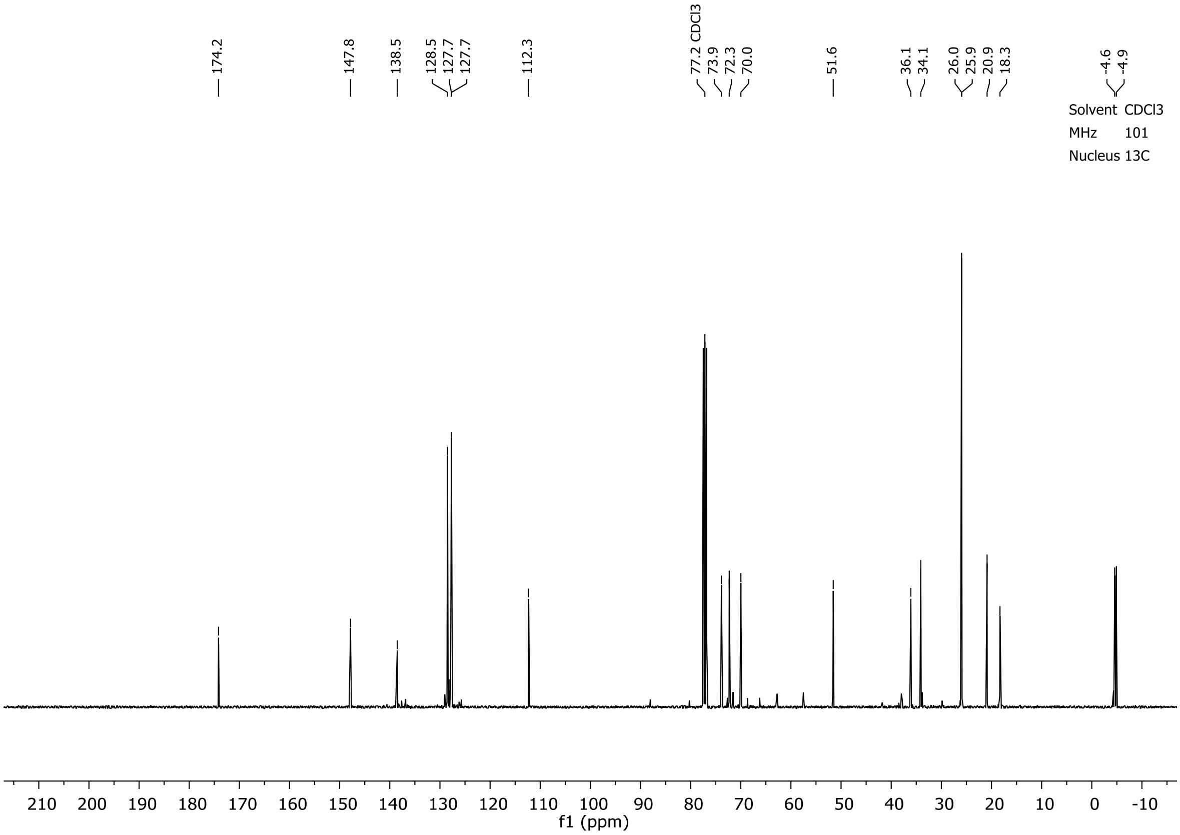
174.2
147.8
138.5
128.5
127.7
127.7
112.3
77.2 CDCl3
73.9
72.3
70.0
51.6
36.1
34.1
26.0
25.9
20.9
18.3
-4.6
-4.9
Solvent CDCl3
MHz 101
Nucleus 13C
210 200 190 180 170 160 150 140 130 120 110 100 90 80 70 60 50 40 30 20 10 0 -10
f1 (ppm)

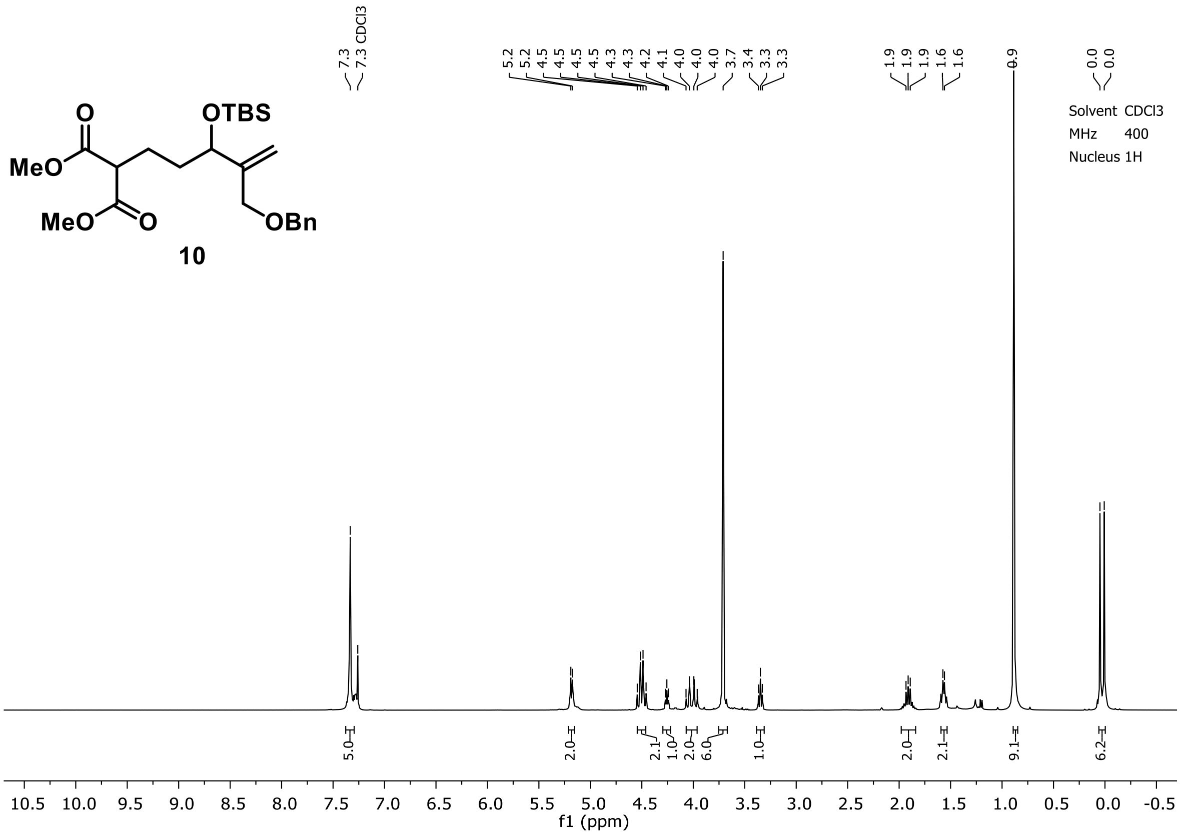
O
OTBS
MeO
MeO
O
OBn
10
7.3
7.3 CDCl3
5.2
5.2
4.5
4.5
4.5
4.5
4.3
4.3
4.2
4.1
4.0
4.0
4.0
3.7
3.4
3.3
3.3
1.9
1.9
1.9
1.6
1.6
0.9
0.0
0.0
Solvent CDCl3
MHz 400
Nucleus 1H
5.0
2.0
2.1
1.0
2.0
6.0
1.0
2.0
2.1
9.1
6.2
10.5 10.0 9.5 9.0 8.5 8.0 7.5 7.0 6.5 6.0 5.5 5.0 4.5 4.0 3.5 3.0 2.5 2.0 1.5 1.0 0.5 0.0 -0.5
f1 (ppm)

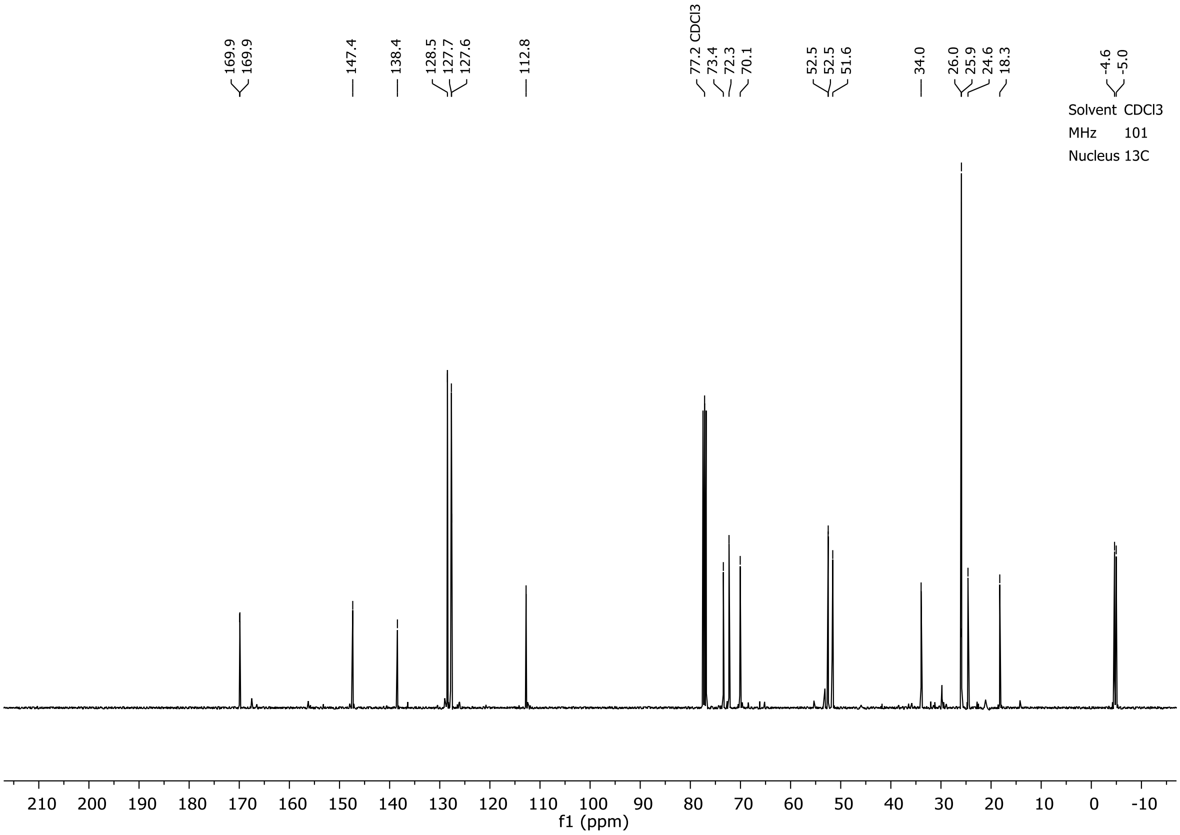
169.9
169.9
147.4
138.4
128.5
127.7
127.6
112.8
77.2 CDCl3
73.4
72.3
70.1
52.5
52.5
51.6
34.0
26.0
25.9
24.6
18.3
-4.6
-5.0
Solvent CDCl3
MHz 101
Nucleus 13C
210 200 190 180 170 160 150 140 130 120 110 100 90 80 70 60 50 40 30 20 10 0 -10
f1 (ppm)

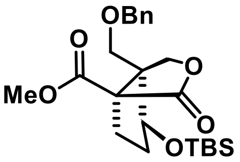
OBn
O
O
MeO
O
OTBS

9

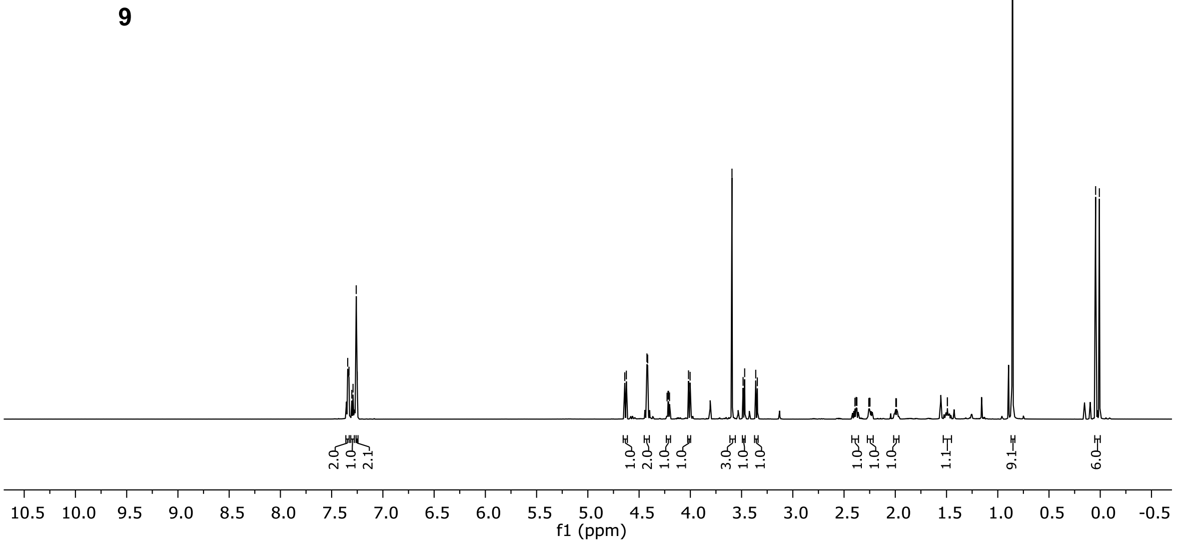
Solvent CDCl3
MHz 600
Nucleus 1H
f1 (ppm)

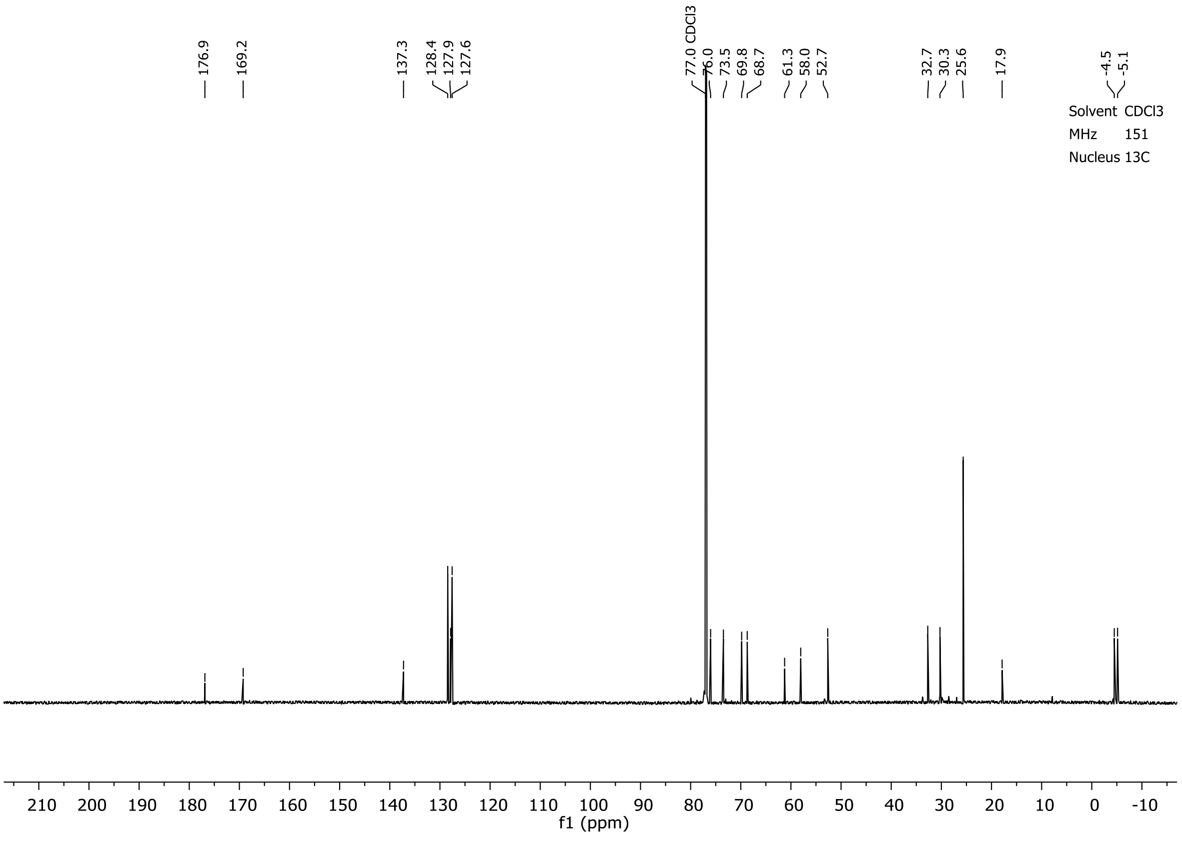
Solvent CDCl3
MHz 151
Nucleus 13C
f1 (ppm)

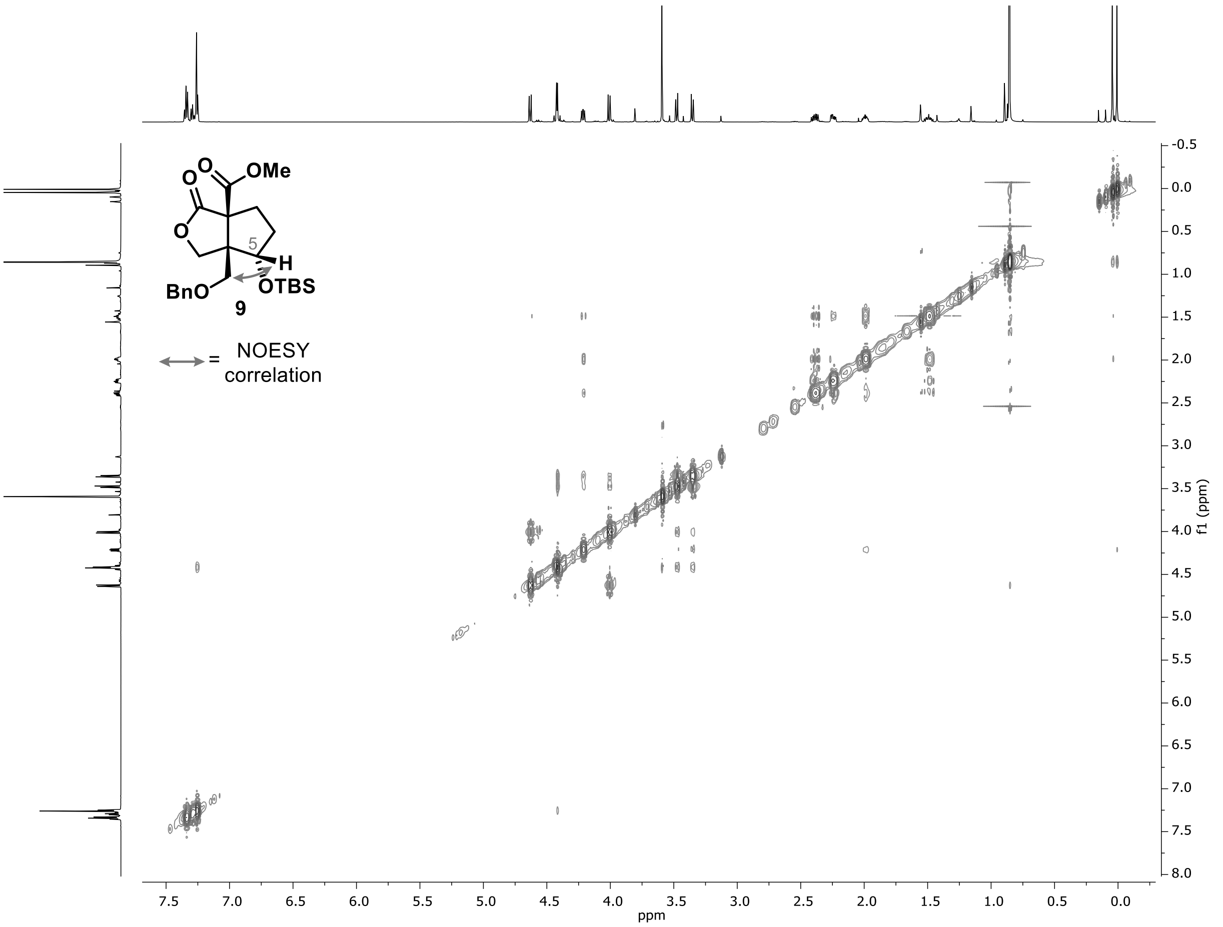
OMe
O
O
O
5
H
OTBS
BnO
9
= NOESY correlation
-0.5
0.0
0.5
1.0
1.5
2.0
2.5
3.0
3.5
4.0
4.5
5.0
5.5
6.0
6.5
7.0
7.5
8.0
f1 (ppm)
7.5
7.0
6.5
6.0
5.5
5.0
4.5
4.0
3.5
3.0
2.5
2.0
1.5
1.0
0.5
0.0
ppm

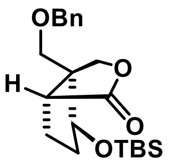
OBn
H
O
O
OTBS

15

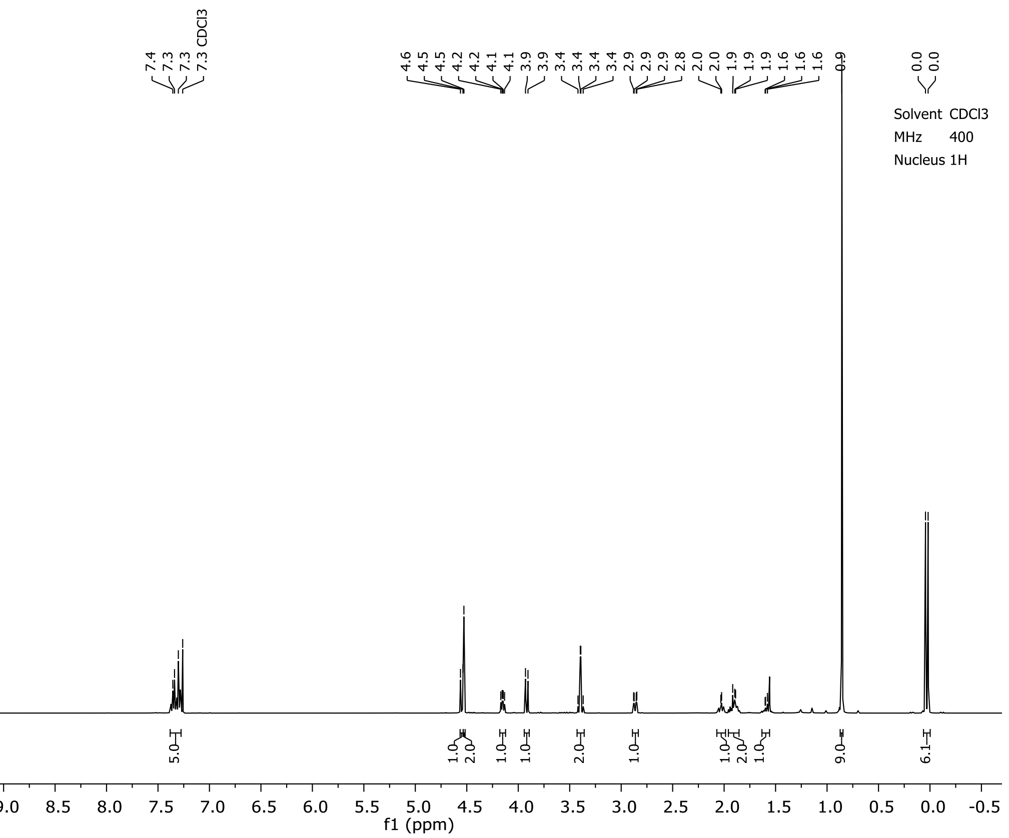
7.4
7.3
7.3
7.3 CDCl3
4.6
4.5
4.5
4.2
4.2
4.1
4.1
3.9
3.9
3.4
3.4
3.4
3.4
2.9
2.9
2.9
2.8
2.0
2.0
1.9
1.9
1.9
1.6
1.6
1.6
0.9
0.0
0.0
Solvent CDCl3
MHz 400
Nucleus 1H
5.0
1.0
2.0
1.0
1.0
2.0
1.0
1.0
2.0
1.0
9.0
6.1
10.5 10.0 9.5 9.0 8.5 8.0 7.5 7.0 6.5 6.0 5.5 5.0 4.5 4.0 3.5 3.0 2.5 2.0 1.5 1.0 0.5 0.0 -0.5
f1 (ppm)

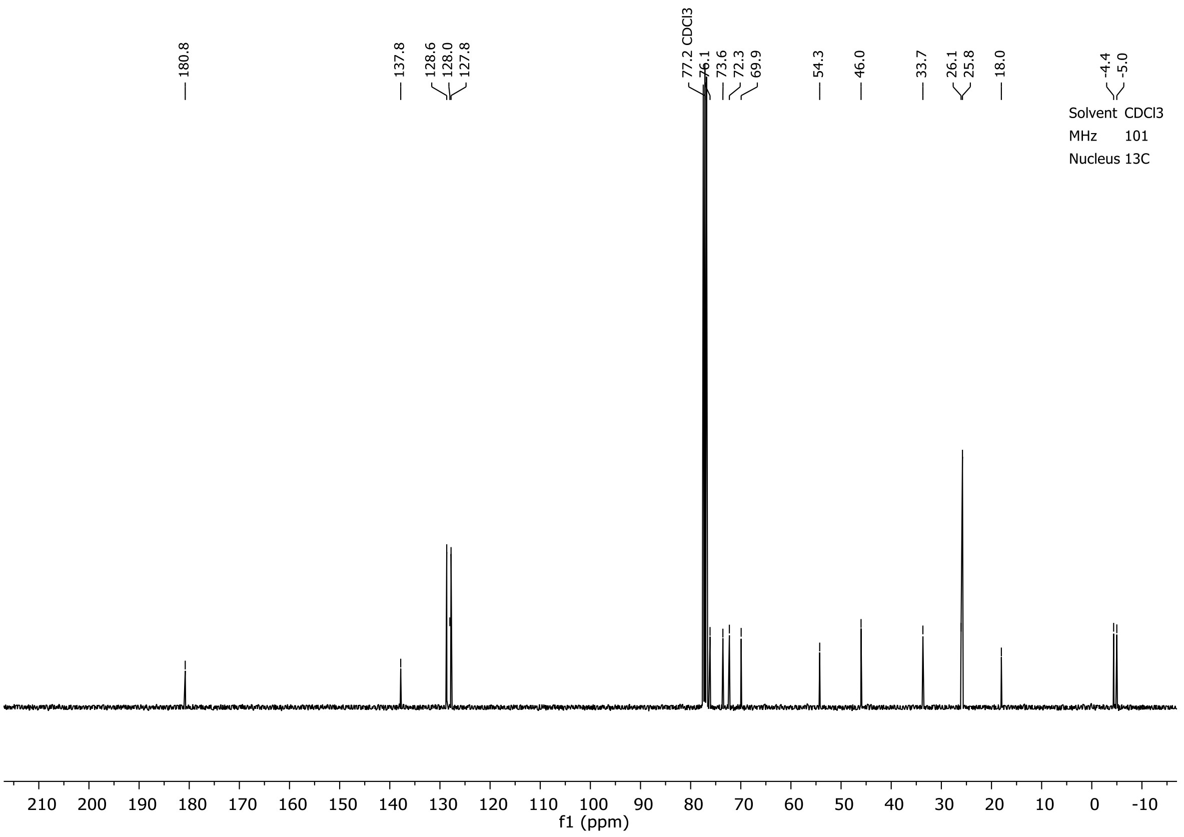
180.8
137.8
128.6
128.0
127.8
77.2 CDCl3
76.1
73.6
72.3
69.9
54.3
46.0
33.7
26.1
25.8
18.0
-4.4
-5.0
Solvent CDCl3
MHz 101
Nucleus 13C
210 200 190 180 170 160 150 140 130 120 110 100 90 80 70 60 50 40 30 20 10 0 -10
f1 (ppm)

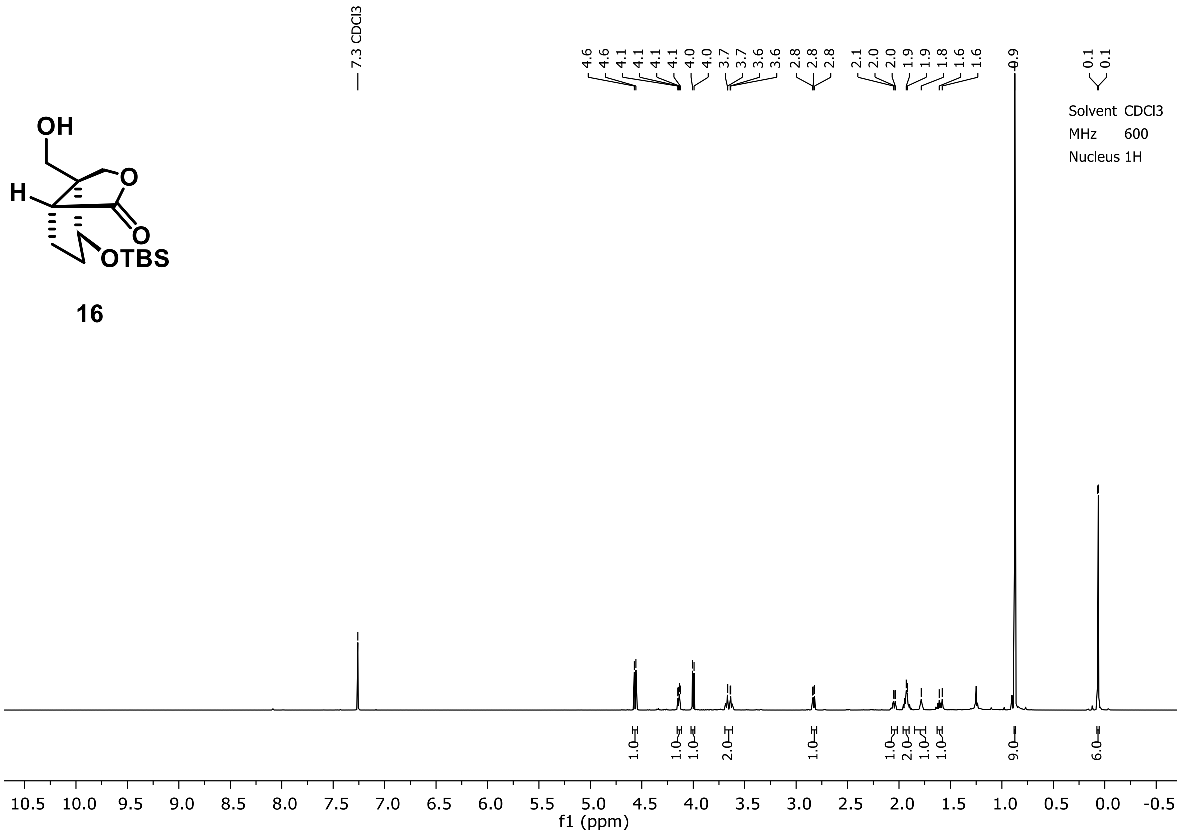
OH
H
O
O
OTBS
16
7.3 CDCl3
4.6
4.6
4.1
4.1
4.1
4.1
4.0
4.0
3.7
3.7
3.6
3.6
2.8
2.8
2.8
2.1
2.0
2.0
1.9
1.9
1.8
1.6
1.6
0.9
0.1
0.1
Solvent CDCl3
MHz 600
Nucleus 1H
1.0
1.0
1.0
2.0
1.0
1.0
2.0
1.0
1.0
9.0
6.0
10.5 10.0 9.5 9.0 8.5 8.0 7.5 7.0 6.5 6.0 5.5 5.0 4.5 4.0 3.5 3.0 2.5 2.0 1.5 1.0 0.5 0.0 -0.5
f1 (ppm)

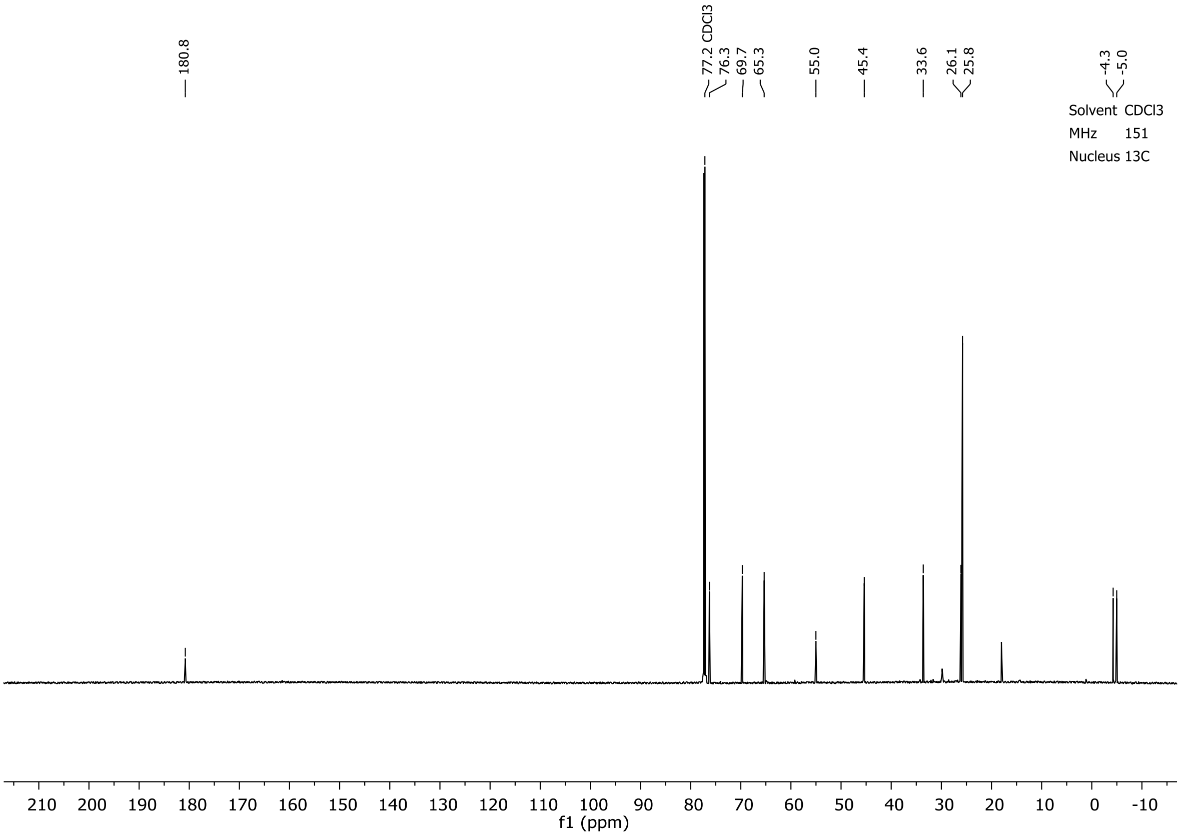
180.8
77.2 CDCl3
76.3
69.7
65.3
55.0
45.4
33.6
26.1
25.8
-4.3
-5.0
Solvent CDCl3
MHz 151
Nucleus 13C
210 200 190 180 170 160 150 140 130 120 110 100 90 80 70 60 50 40 30 20 10 0 -10
f1 (ppm)

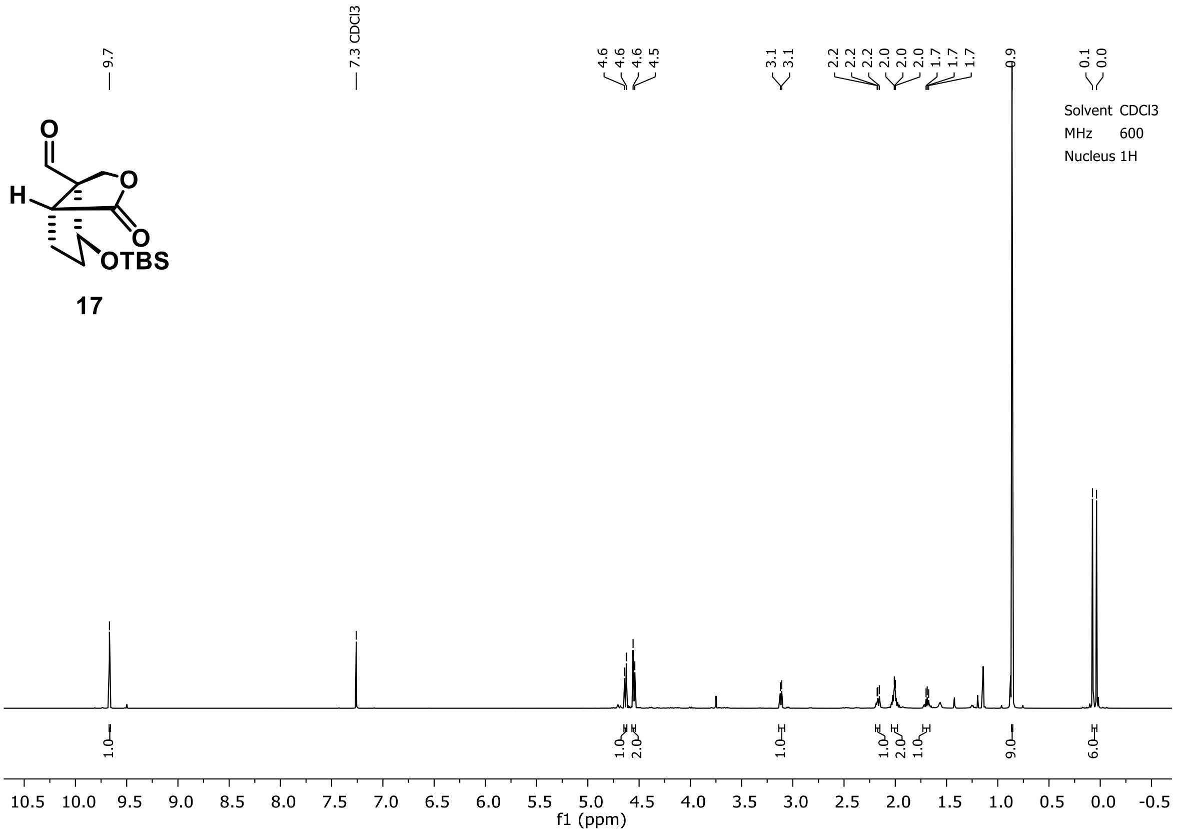
O
H
O
O
OTBS
17
9.7
7.3 CDCl3
4.6
4.6
4.6
4.5
3.1
3.1
2.2
2.2
2.2
2.2
2.0
2.0
2.0
2.0
1.7
1.7
1.7
1.7
0.9
0.1
0.0
Solvent CDCl3
MHz 600
Nucleus 1H
1.0
1.0
2.0
1.0
1.0
2.0
1.0
9.0
6.0
10.5 10.0 9.5 9.0 8.5 8.0 7.5 7.0 6.5 6.0 5.5 5.0 4.5 4.0 3.5 3.0 2.5 2.0 1.5 1.0 0.5 0.0 -0.5
f1 (ppm)

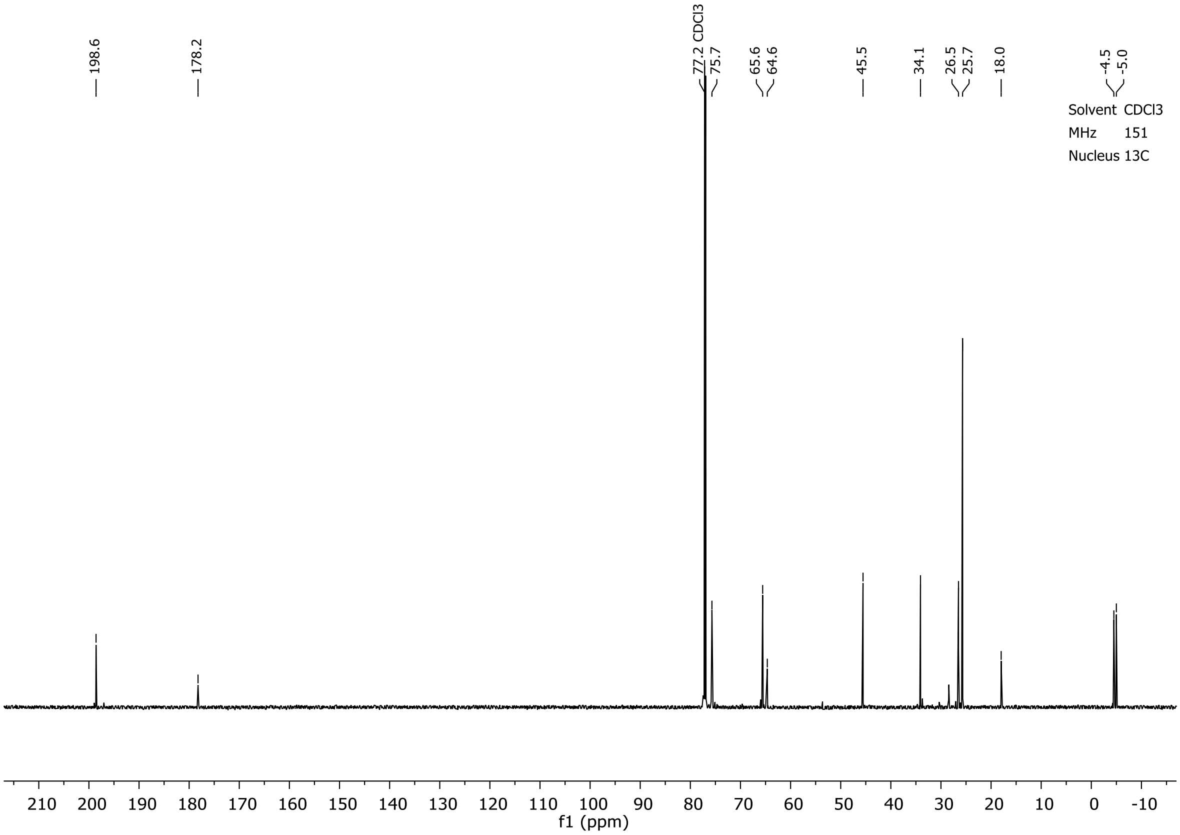
198.6
178.2
77.2 CDCl3
75.7
65.6
64.6
45.5
34.1
26.5
25.7
18.0
-4.5
-5.0
Solvent CDCl3
MHz 151
Nucleus 13C
210 200 190 180 170 160 150 140 130 120 110 100 90 80 70 60 50 40 30 20 10 0 -10
f1 (ppm)

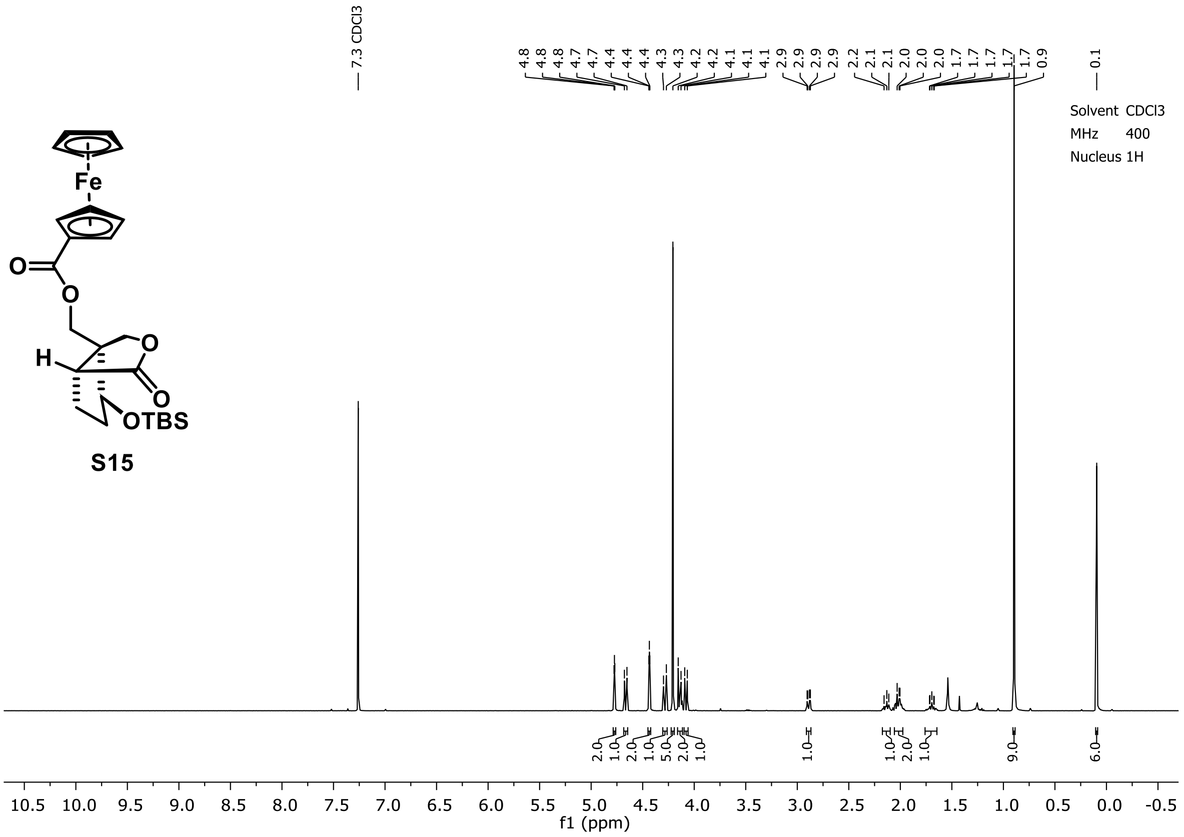
Fe
O
O
H
O
O
OTBS
S15
7.3 CDCl3
Solvent CDCl3
MHz 400
Nucleus 1H
f1 (ppm)

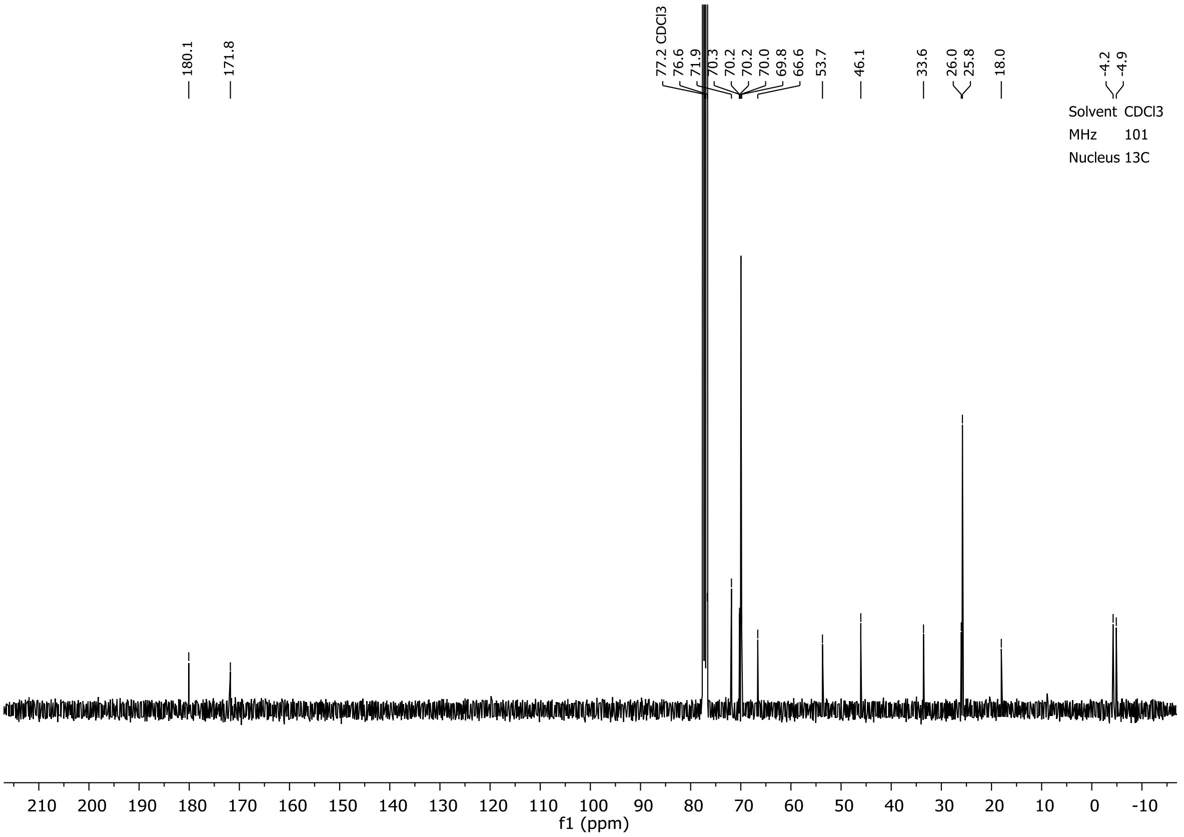
180.1
171.8
77.2 CDCl3
76.6
71.9
70.3
70.2
70.2
70.0
69.8
66.6
53.7
46.1
33.6
26.0
25.8
18.0
-4.2
-4.9
Solvent CDCl3
MHz 101
Nucleus 13C
f1 (ppm)

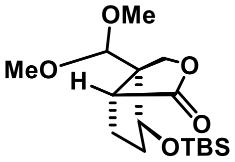
OMe
MeO
H
O
O
OTBS

18

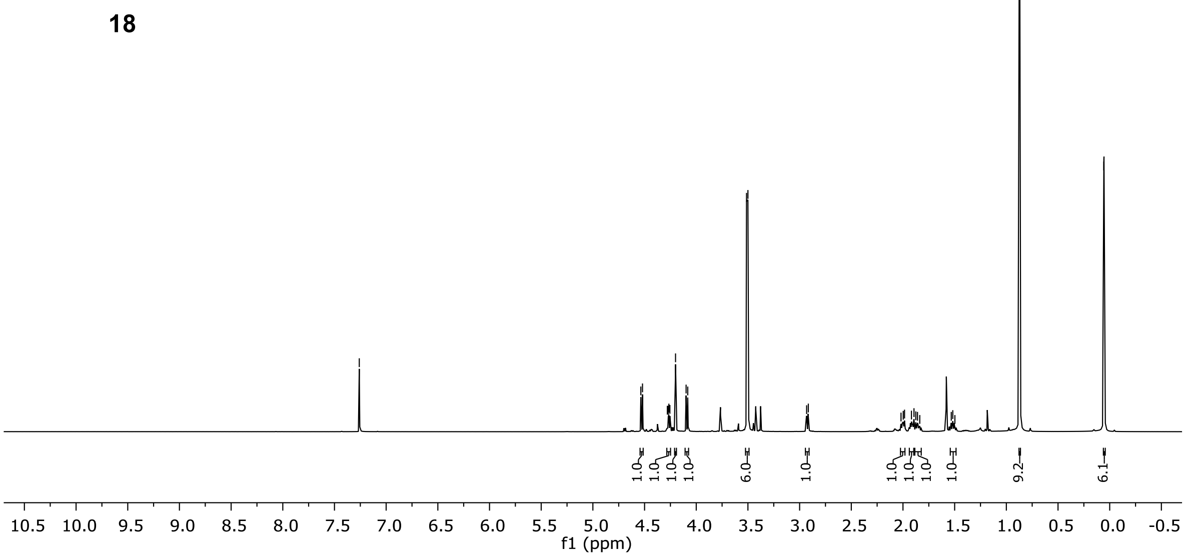
7.3 CDCl3
Solvent CDCl3
MHz 600
Nucleus 1H
f1 (ppm)

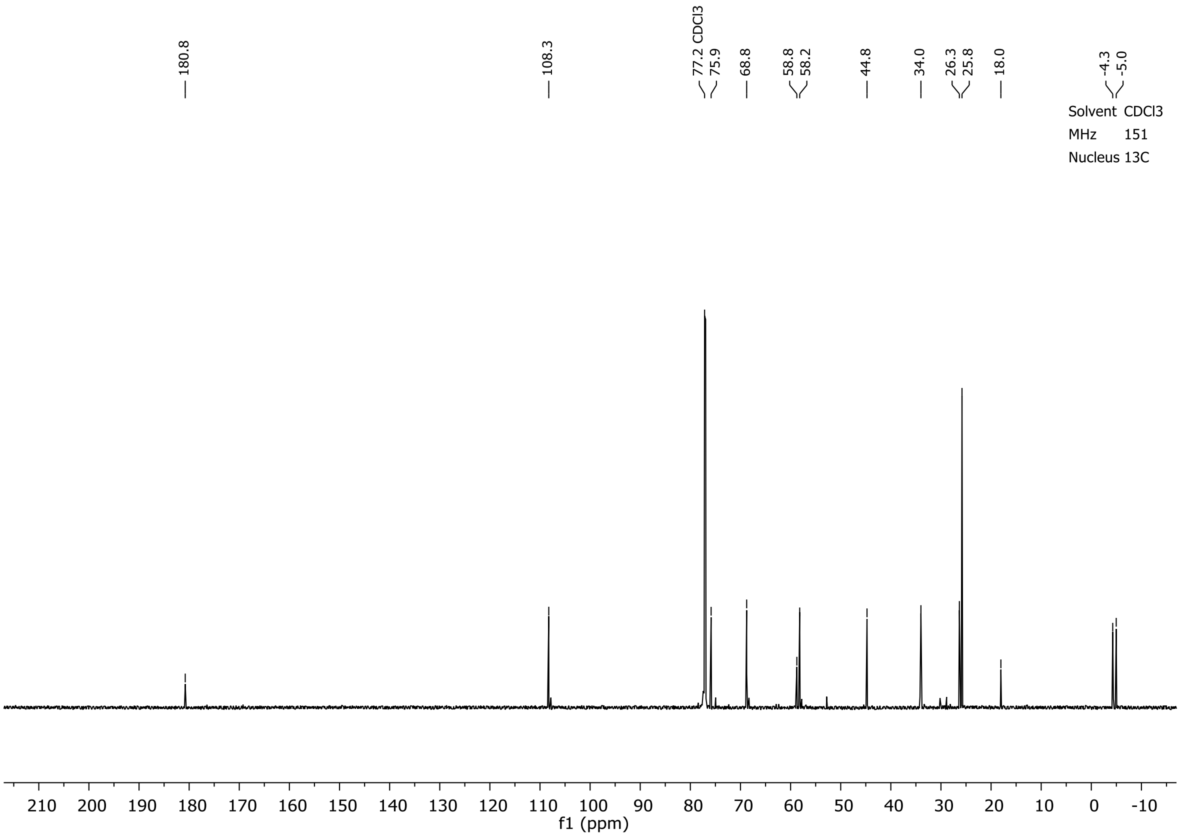
180.8
108.3
77.2 CDCl3
75.9
68.8
58.8
58.2
44.8
34.0
26.3
25.8
18.0
-4.3
-5.0
Solvent CDCl3
MHz 151
Nucleus 13C
f1 (ppm)

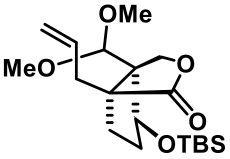
OMe
MeO
O
O
OTBS

19

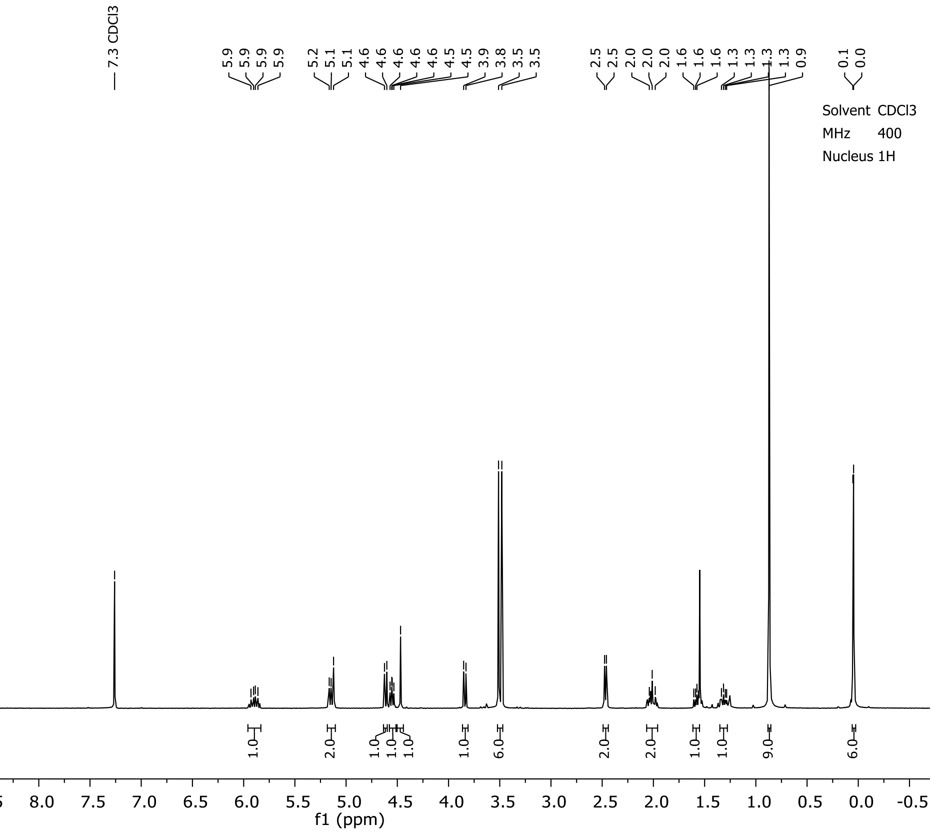
7.3 CDCl3
5.9
5.9
5.9
5.9
5.2
5.1
5.1
4.6
4.6
4.6
4.6
4.5
4.5
3.9
3.8
3.5
3.5
2.5
2.5
2.0
2.0
2.0
1.6
1.6
1.6
1.3
1.3
1.3
1.3
0.9
0.1
0.0
Solvent CDCl3
MHz 400
Nucleus 1H
1.0
2.0
1.0
1.0
1.0
1.0
6.0
2.0
2.0
1.0
1.0
9.0
6.0
10.5 10.0 9.5 9.0 8.5 8.0 7.5 7.0 6.5 6.0 5.5 5.0 4.5 4.0 3.5 3.0 2.5 2.0 1.5 1.0 0.5 0.0 -0.5
f1 (ppm)

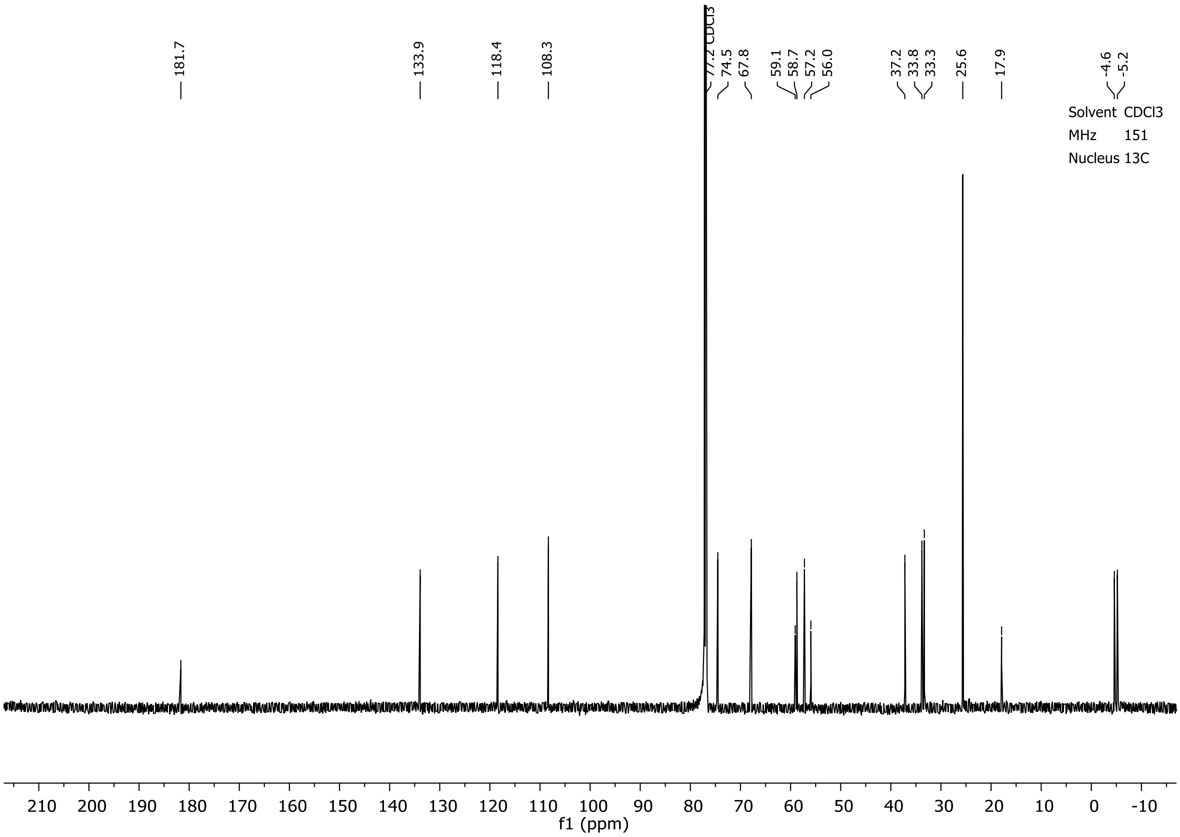
181.7
133.9
118.4
108.3
77.2 CDCl3
74.5
67.8
59.1
58.7
57.2
56.0
37.2
33.8
33.3
25.6
17.9
-4.6
-5.2
Solvent CDCl3
MHz 151
Nucleus 13C
210 200 190 180 170 160 150 140 130 120 110 100 90 80 70 60 50 40 30 20 10 0 -10
f1 (ppm)

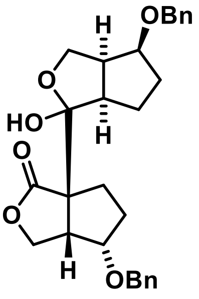
H
OBn
O
HO
H
O
O
H
OBn

39

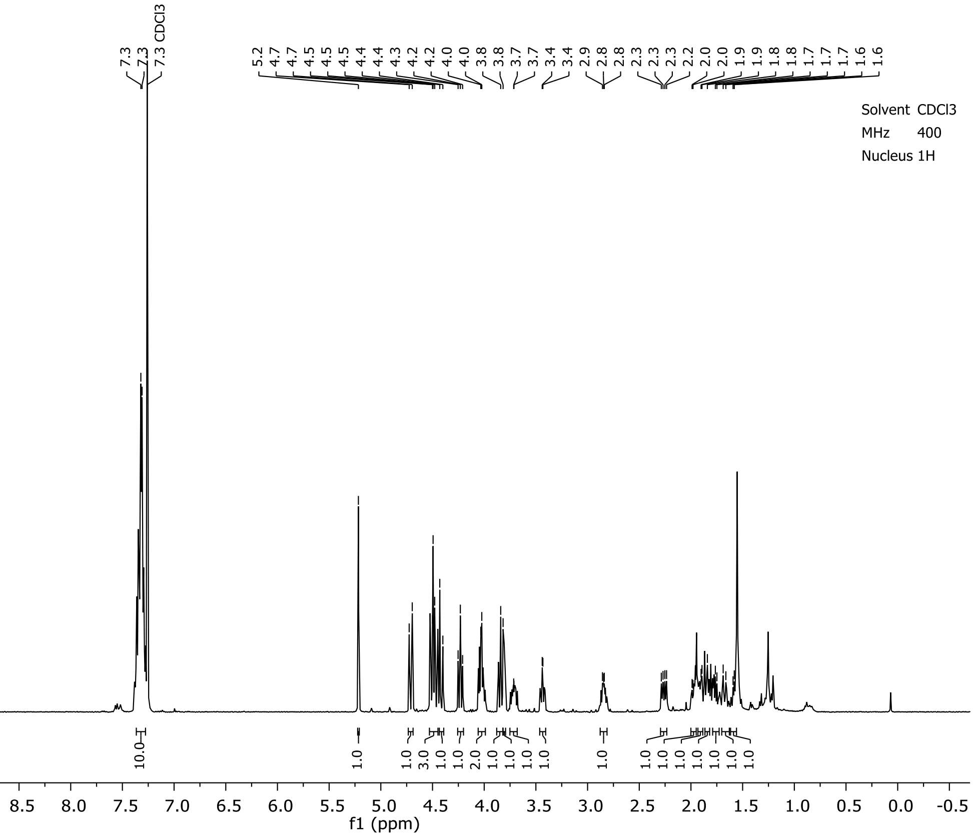
7.3
7.3
7.3 CDCl3
5.2 4.7 4.7 4.5 4.5 4.5 4.5 4.4 4.4 4.4 4.3 4.2 4.2 4.2 4.0 4.0 3.8 3.8 3.7 3.7 3.4 3.4 2.9 2.8 2.8 2.3 2.3 2.3 2.2 2.2 2.0 2.0 1.9 1.9 1.8 1.8 1.7 1.7 1.7 1.6 1.6
Solvent CDCl3
MHz 400
Nucleus 1H
10.0 1.0 1.0 3.0 1.0 1.0 2.0 1.0 1.0 1.0 1.0 1.0 1.0 1.0 1.0 1.0 1.0 1.0
10.5 10.0 9.5 9.0 8.5 8.0 7.5 7.0 6.5 6.0 5.5 5.0 4.5 4.0 3.5 3.0 2.5 2.0 1.5 1.0 0.5 0.0 -0.5
f1 (ppm)

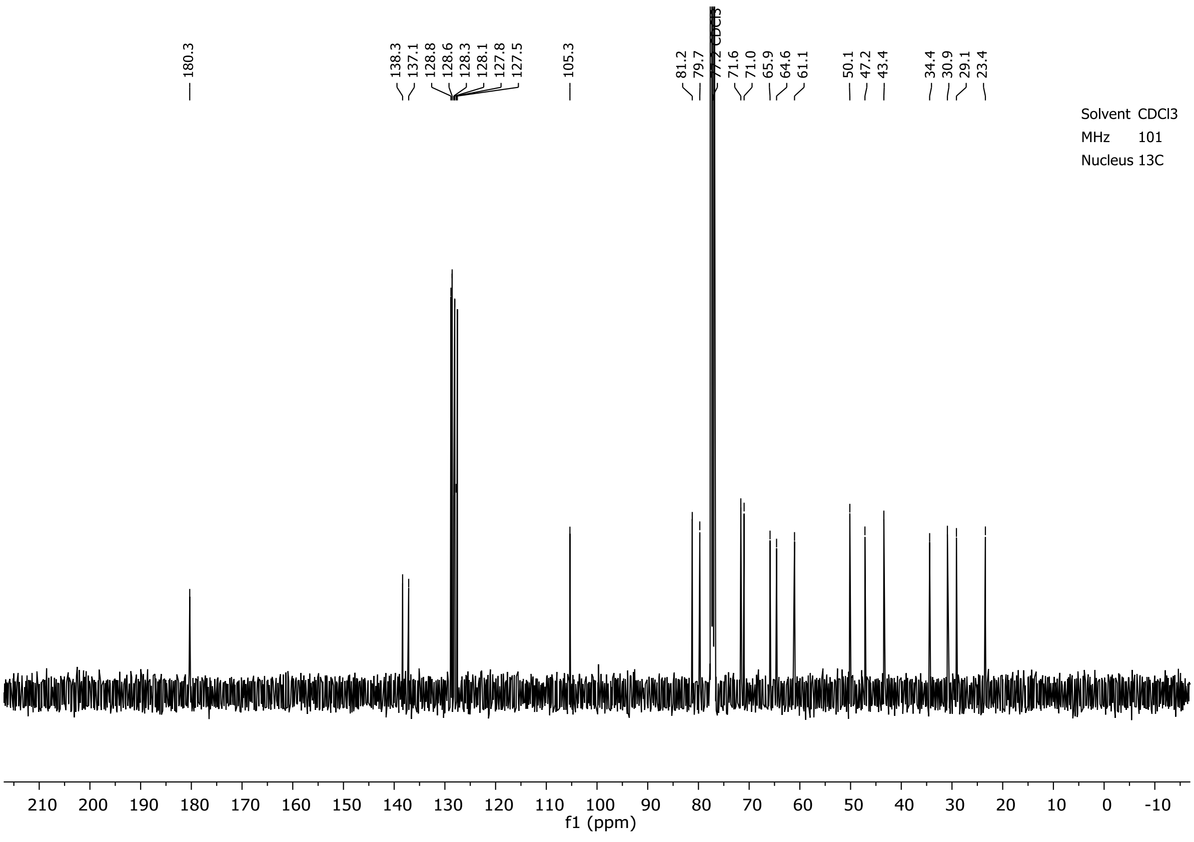
180.3
138.3 137.1 128.8 128.6 128.3 128.1 127.8 127.5
105.3
81.2 79.7 77.2 CDCl3 71.6 71.0 65.9 64.6 61.1
50.1 47.2 43.4
34.4 30.9 29.1 23.4
Solvent CDCl3
MHz 101
Nucleus 13C
210 200 190 180 170 160 150 140 130 120 110 100 90 80 70 60 50 40 30 20 10 0 -10
f1 (ppm)

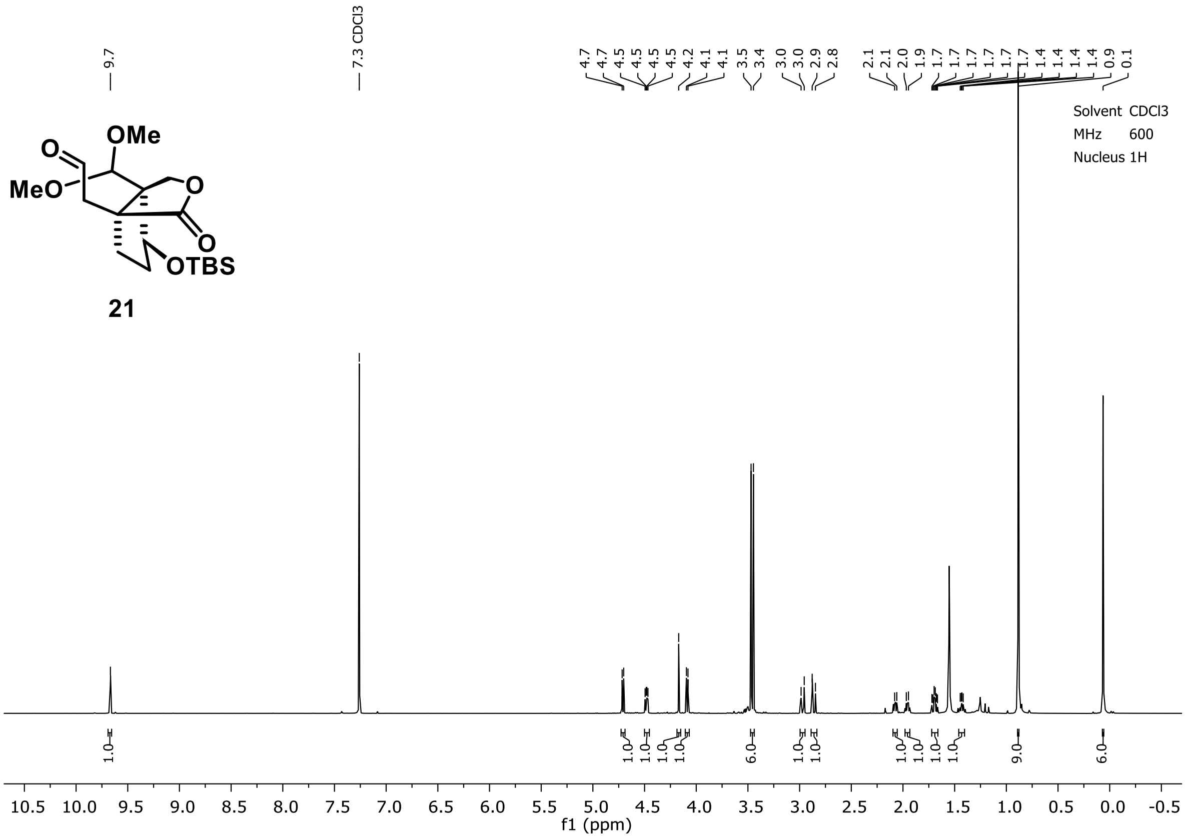
OMe
MeO
O
OTBS
21
9.7
7.3 CDCl3
Solvent CDCl3
MHz 600
Nucleus 1H
f1 (ppm)

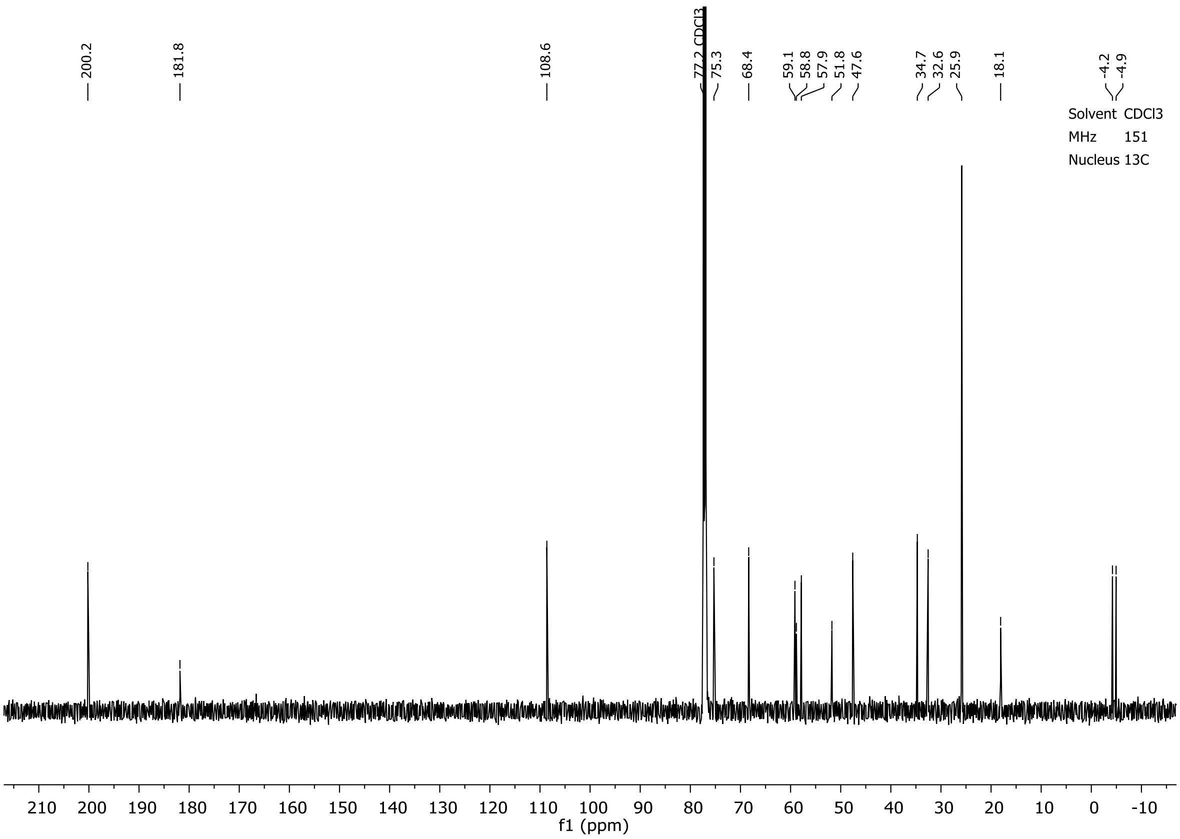
200.2
181.8
108.6
77.2 CDCl3
75.3
68.4
59.1
58.8
57.9
51.8
47.6
34.7
32.6
25.9
18.1
-4.2
-4.9
Solvent CDCl3
MHz 151
Nucleus 13C
f1 (ppm)

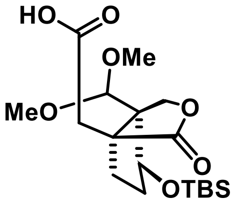
HO
O
OMe
MeO
O
O
OTBS

8

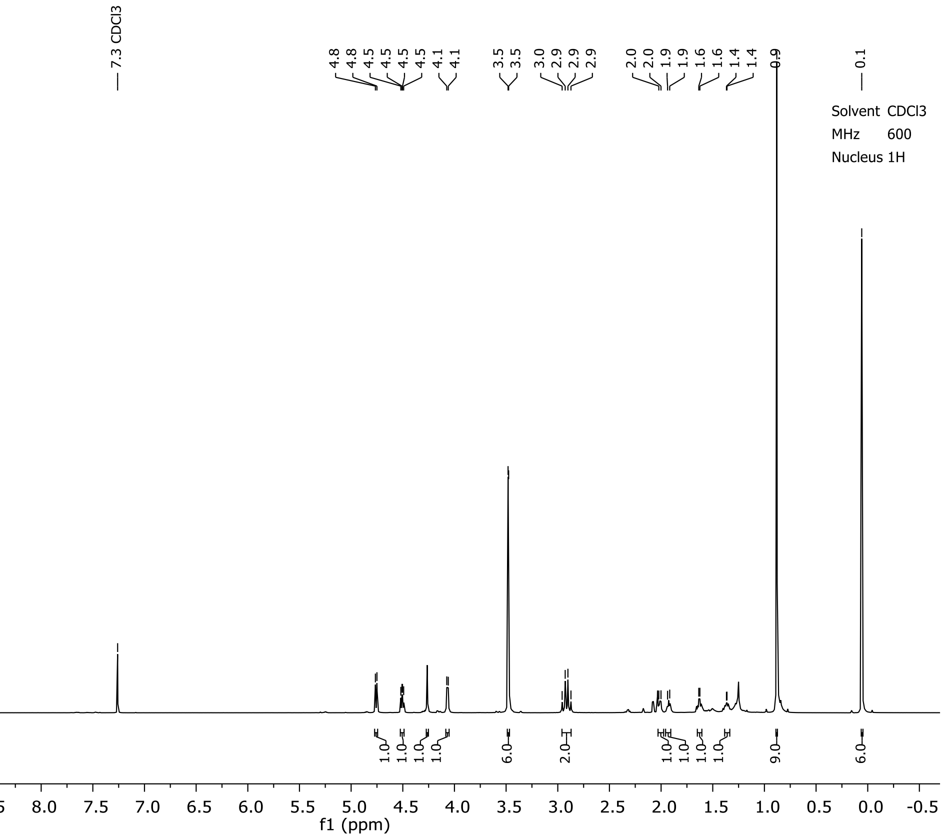
7.3 CDCl3
4.8
4.8
4.5
4.5
4.5
4.5
4.5
4.1
4.1
3.5
3.5
3.0
2.9
2.9
2.9
2.0
2.0
1.9
1.9
1.9
1.6
1.6
1.4
1.4
0.9
0.1
Solvent CDCl3
MHz 600
Nucleus 1H
1.0
1.0
1.0
1.0
6.0
2.0
1.0
1.0
1.0
1.0
9.0
6.0
10.5 10.0 9.5 9.0 8.5 8.0 7.5 7.0 6.5 6.0 5.5 5.0 4.5 4.0 3.5 3.0 2.5 2.0 1.5 1.0 0.5 0.0 -0.5
f1 (ppm)

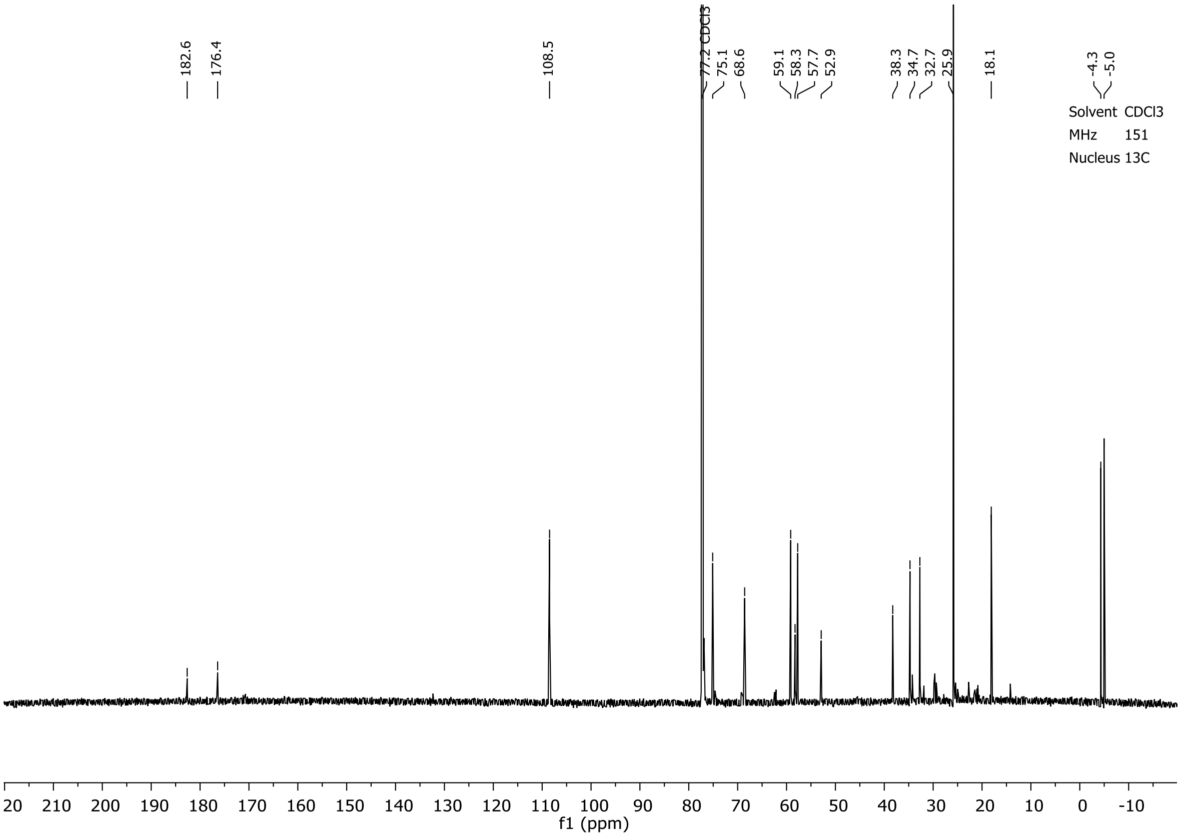
182.6
176.4
108.5
77.2 CDCl3
75.1
68.6
59.1
58.3
57.7
52.9
38.3
34.7
32.7
25.9
18.1
-4.3
-5.0
Solvent CDCl3
MHz 151
Nucleus 13C
20 210 200 190 180 170 160 150 140 130 120 110 100 90 80 70 60 50 40 30 20 10 0 -10
f1 (ppm)

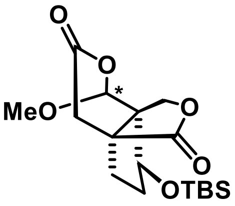
MeO
O
OTBS

d.r. 2:1
S16

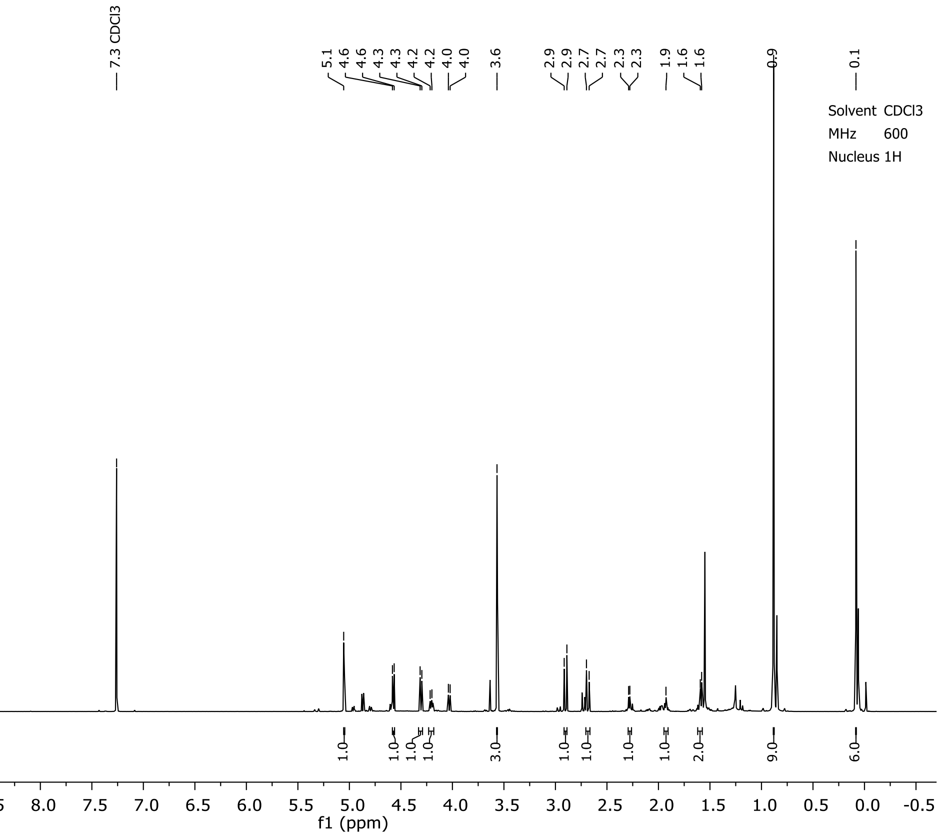
7.3 CDCl3
5.1
4.6
4.6
4.3
4.3
4.2
4.2
4.0
4.0
3.6
2.9
2.9
2.7
2.7
2.3
2.3
1.9
1.6
1.6
0.9
0.1
Solvent CDCl3
MHz 600
Nucleus 1H
1.0
1.0
1.0
1.0
3.0
1.0
1.0
1.0
1.0
2.0
9.0
6.0
10.5 10.0 9.5 9.0 8.5 8.0 7.5 7.0 6.5 6.0 5.5 5.0 4.5 4.0 3.5 3.0 2.5 2.0 1.5 1.0 0.5 0.0 -0.5
f1 (ppm)

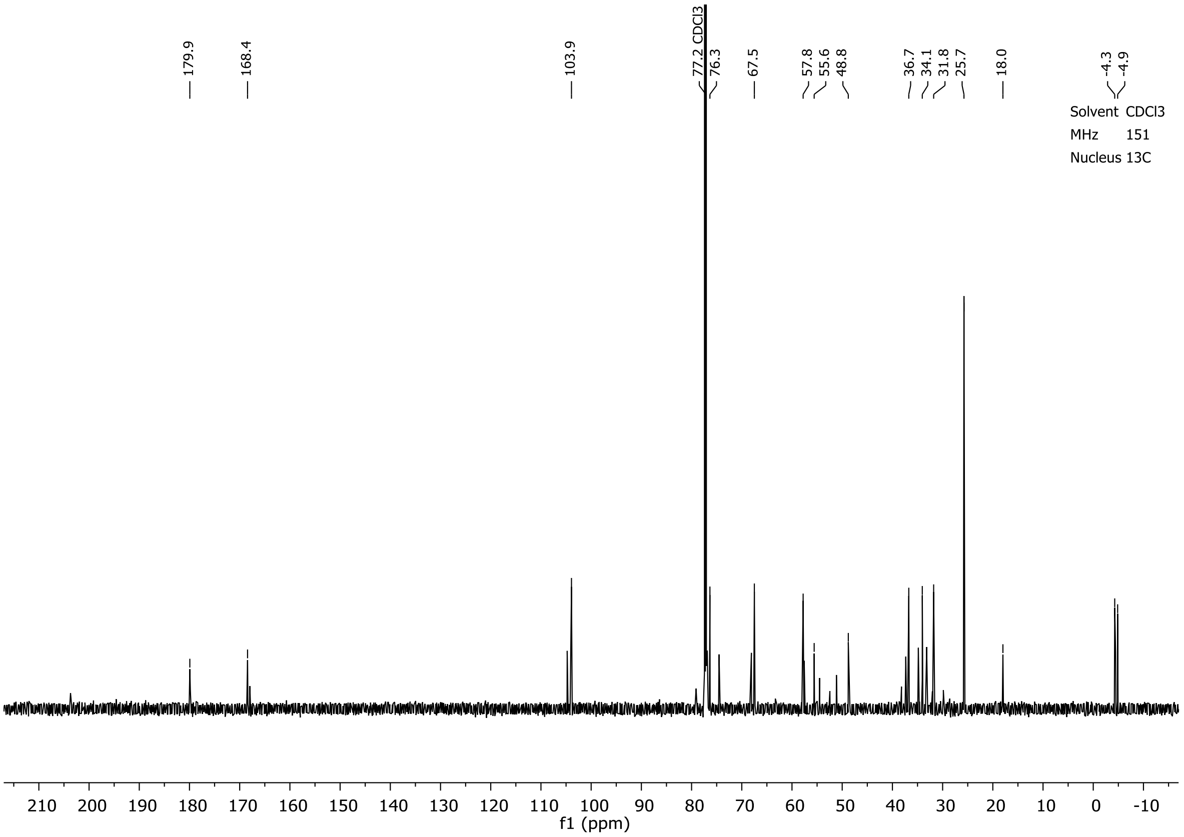
179.9
168.4
103.9
77.2 CDCl3
76.3
67.5
57.8
55.6
48.8
36.7
34.1
31.8
25.7
18.0
-4.3
-4.9
Solvent CDCl3
MHz 151
Nucleus 13C
210 200 190 180 170 160 150 140 130 120 110 100 90 80 70 60 50 40 30 20 10 0 -10
f1 (ppm)

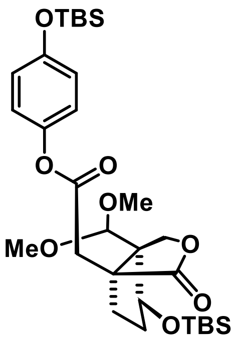
OTBS
O
OMe
MeO
O
O
OTBS

7

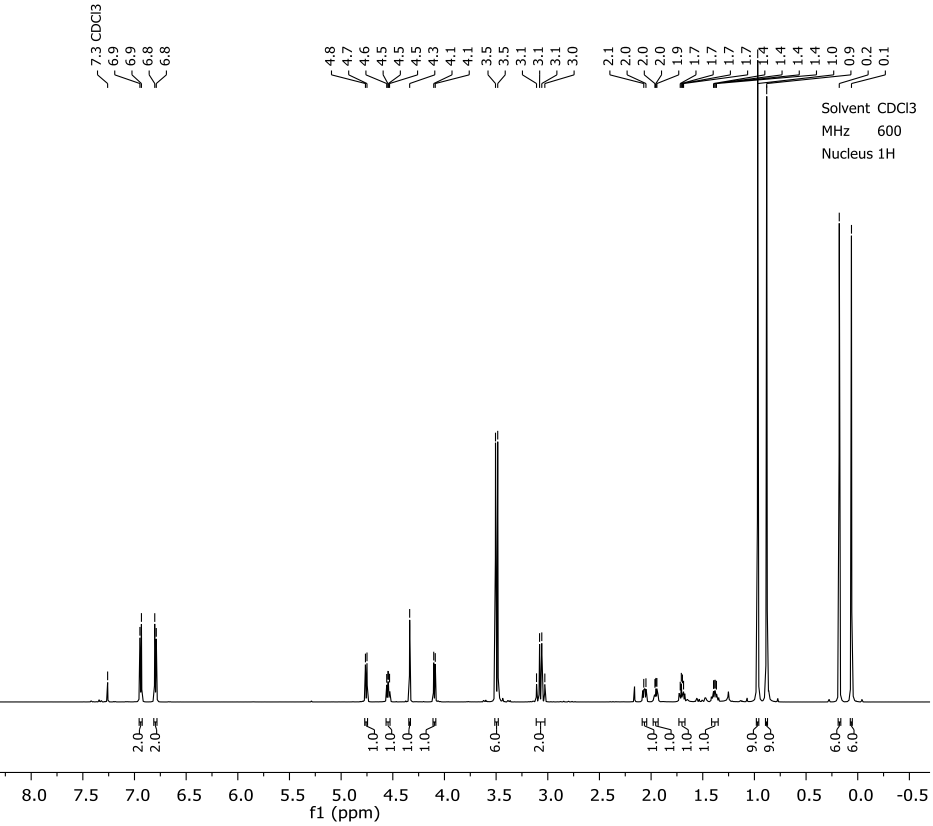
Solvent CDCl3
MHz 600
Nucleus 1H
f1 (ppm)

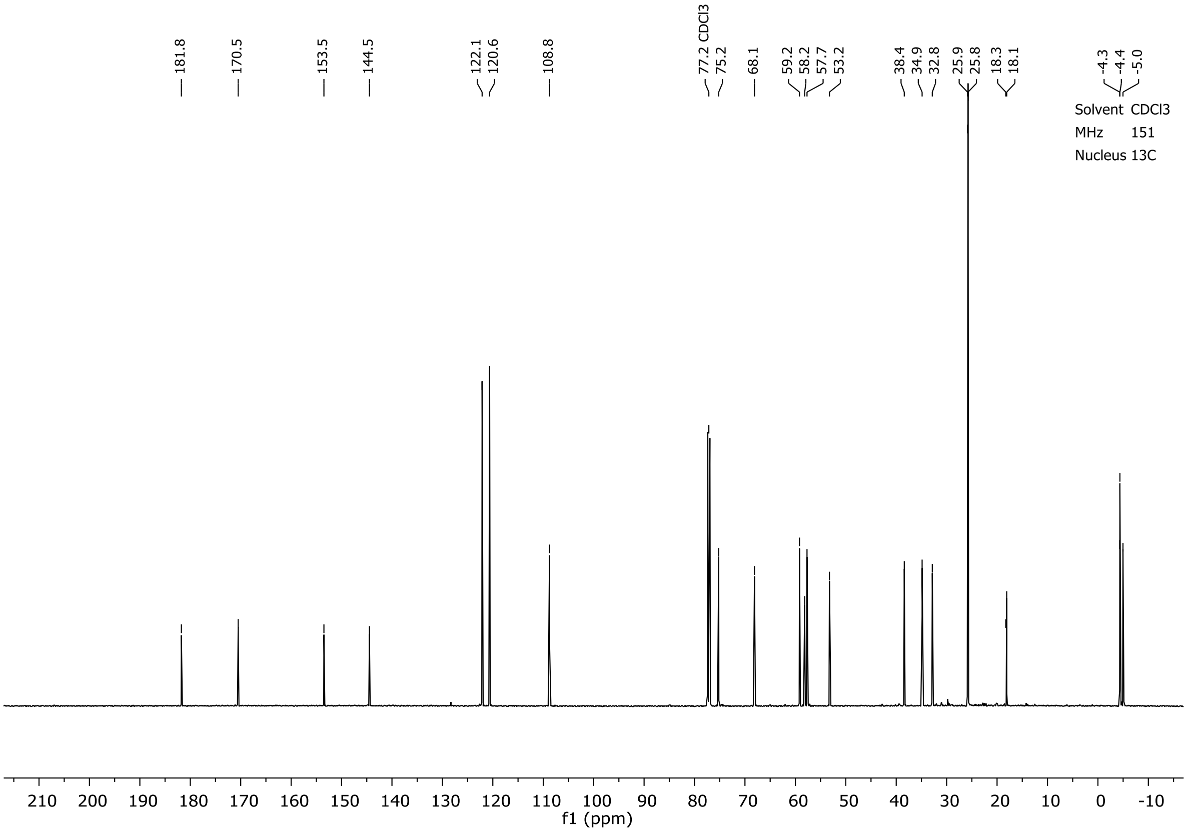
Solvent CDCl3
MHz 151
Nucleus 13C
f1 (ppm)

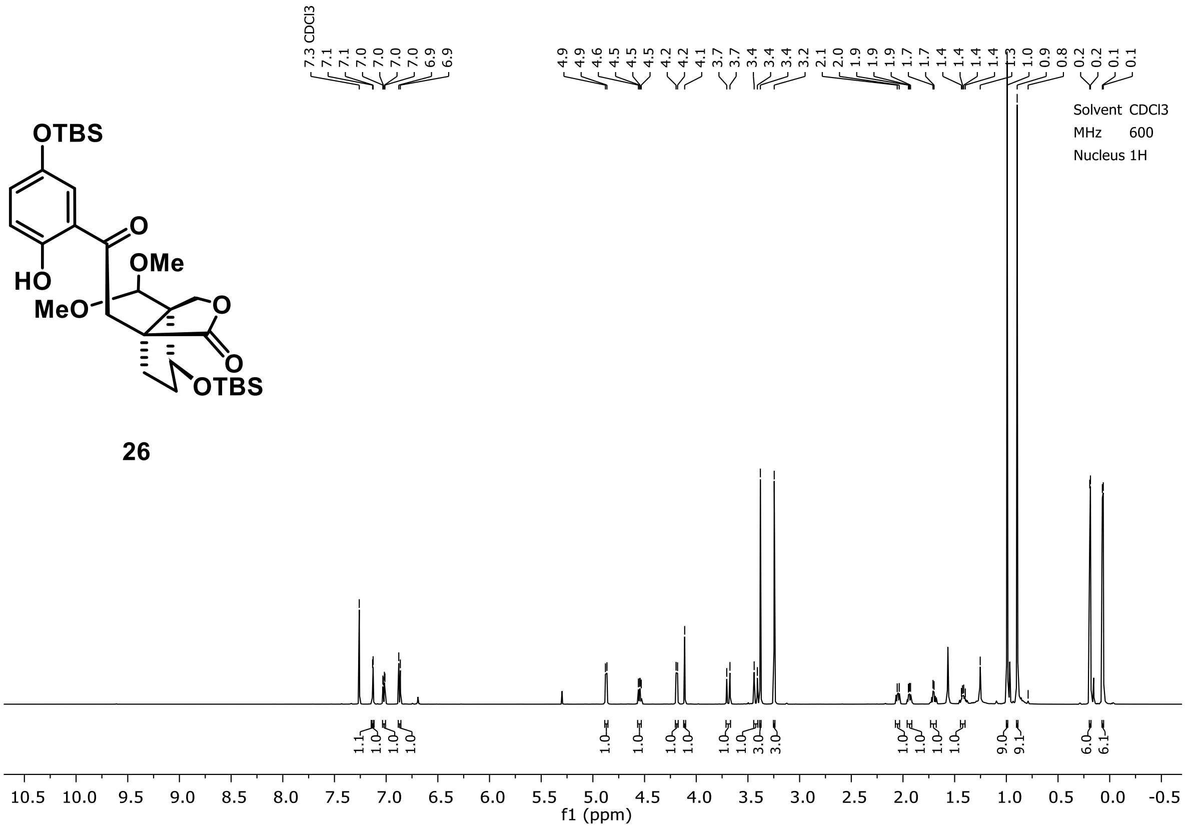
OTBS
O
OMe
HO
MeO
O
O
OTBS
Solvent CDCl3
MHz 600
Nucleus 1H
f1 (ppm)

26

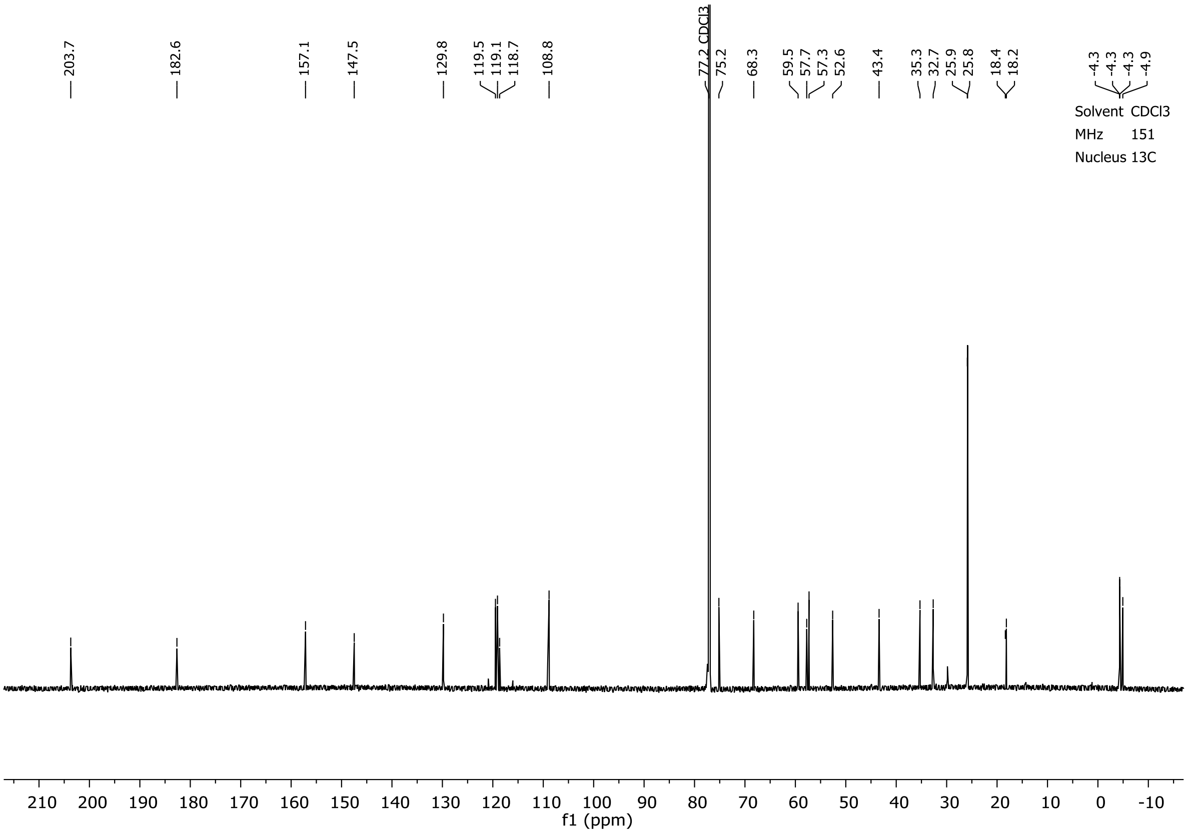
Solvent CDCl3
MHz 151
Nucleus 13C
f1 (ppm)

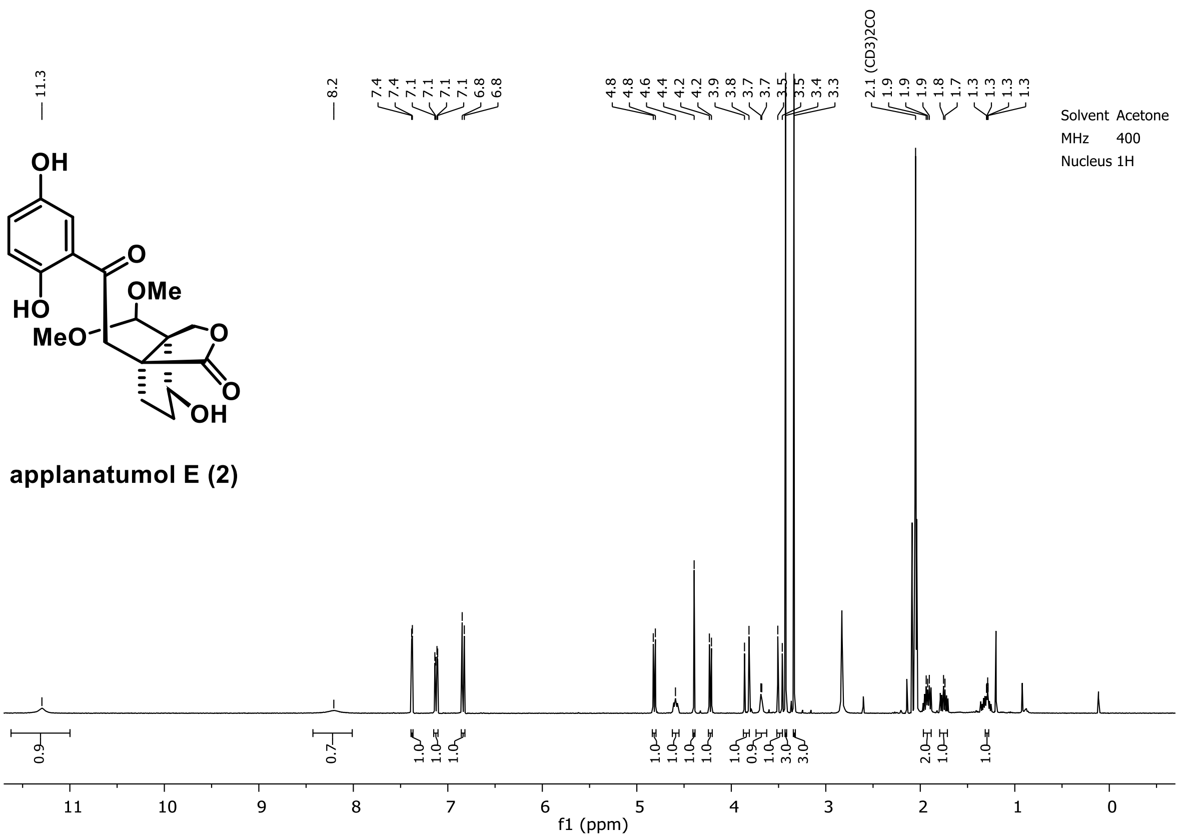

applanatumol E (2)

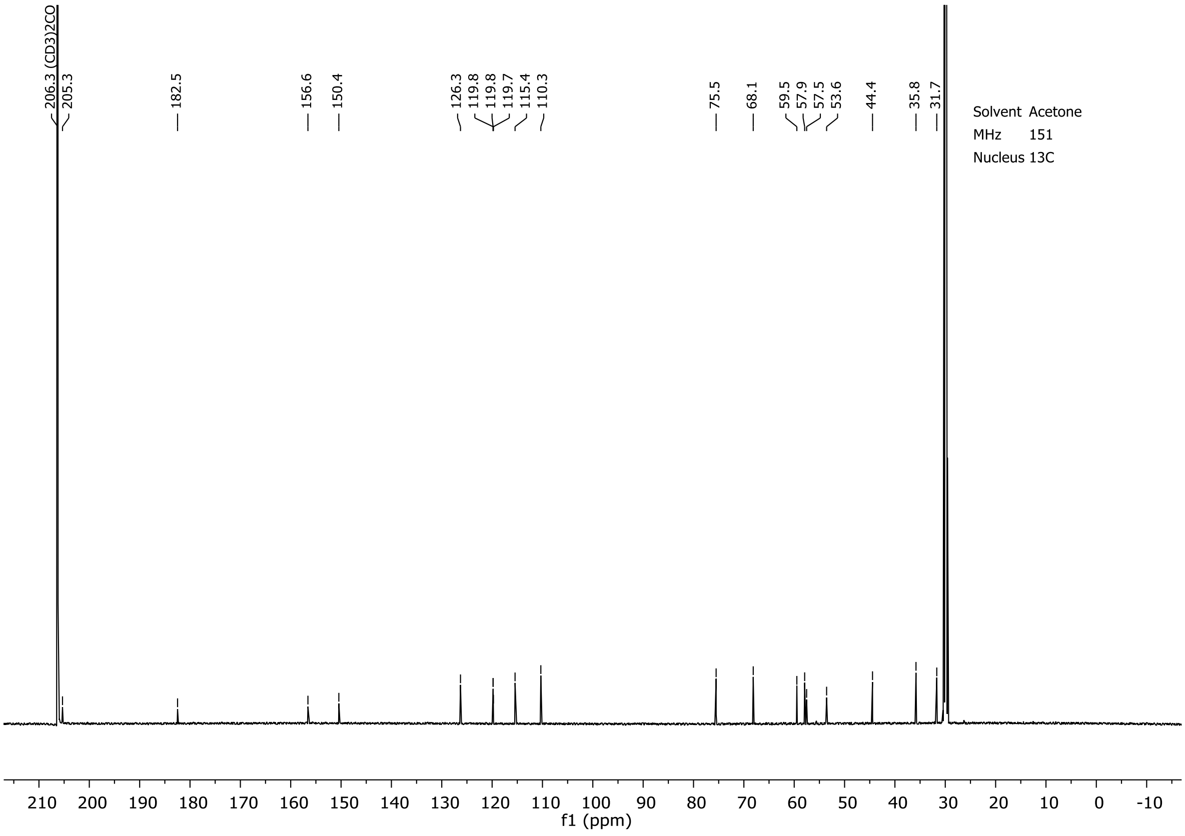

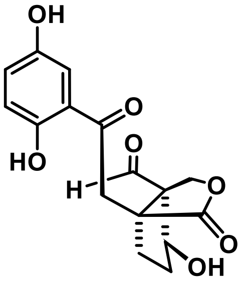

lingzhilactone B (3)

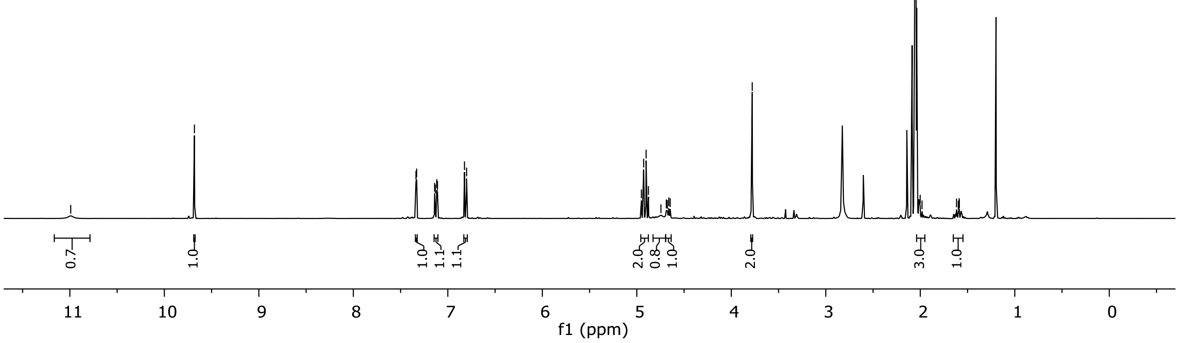

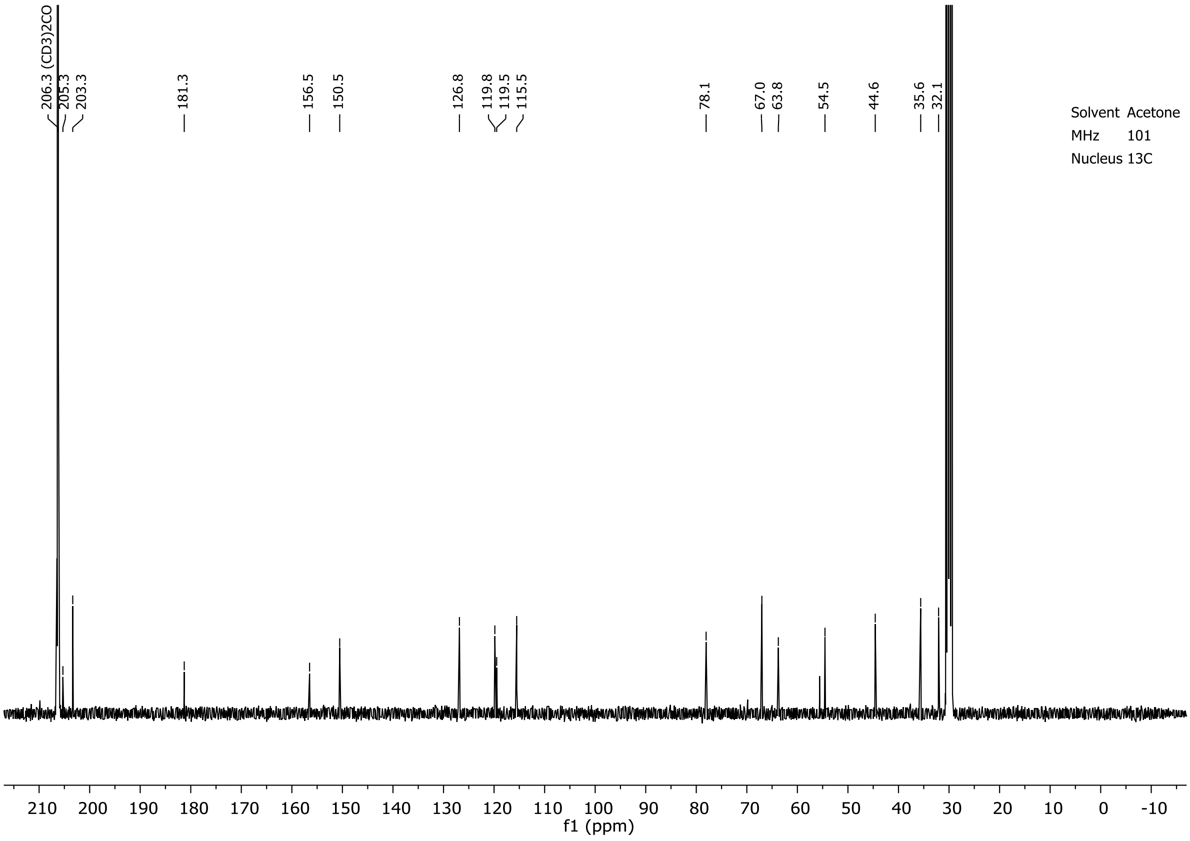

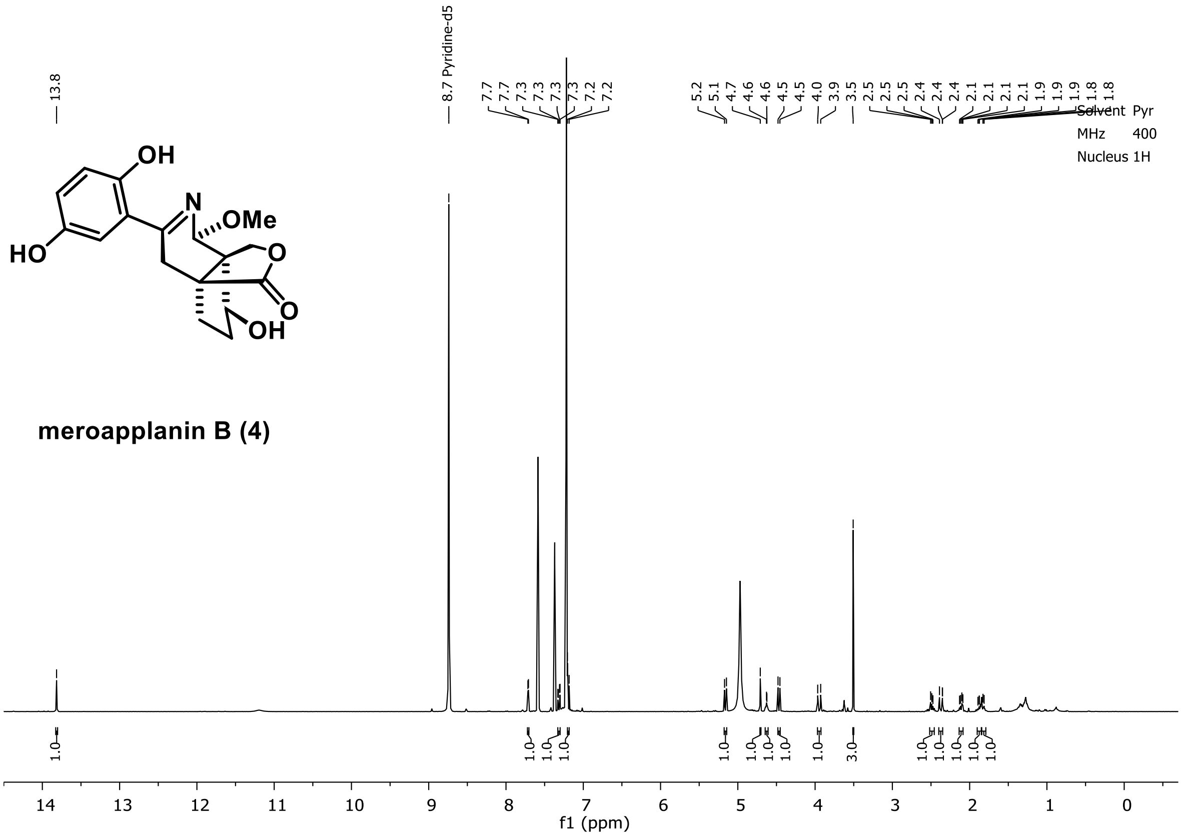

meroapplanin B (4)

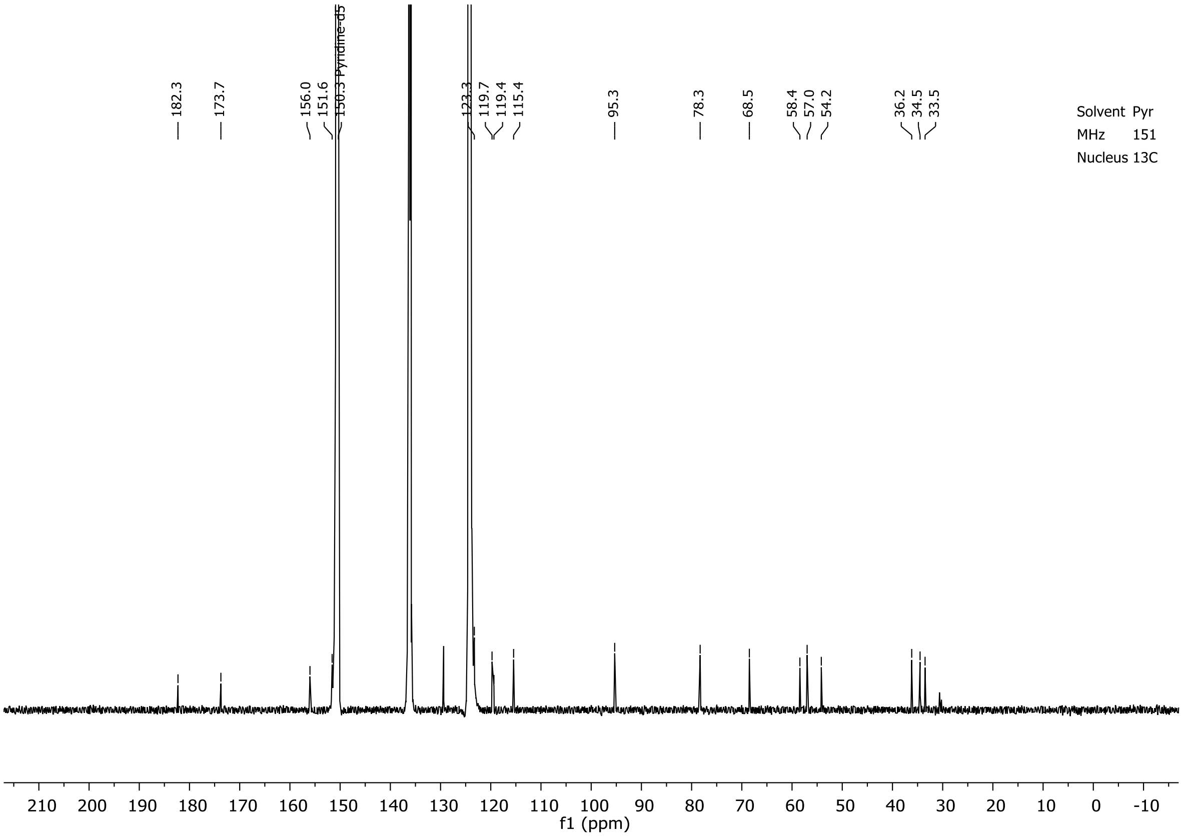

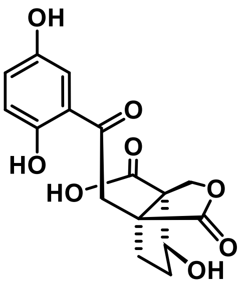

applanatumol I (29)

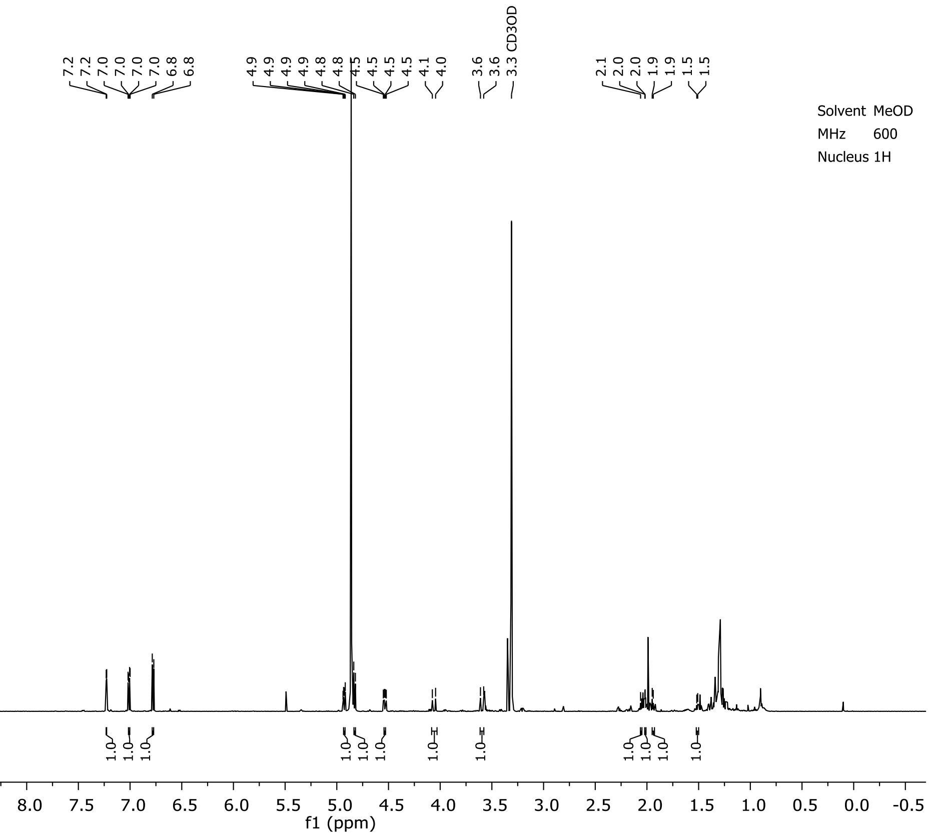

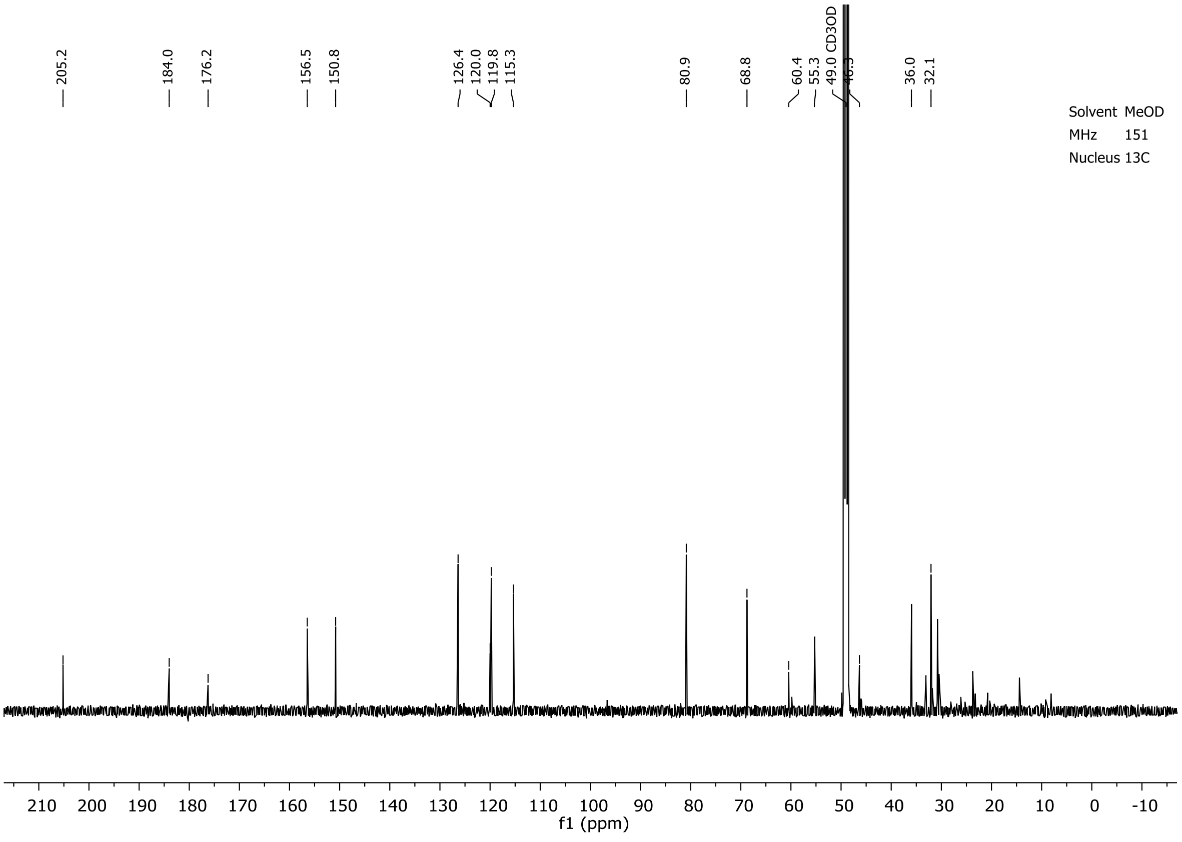

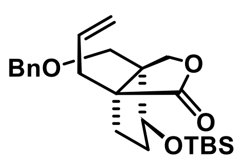
BnO
O
O
OTBS

20

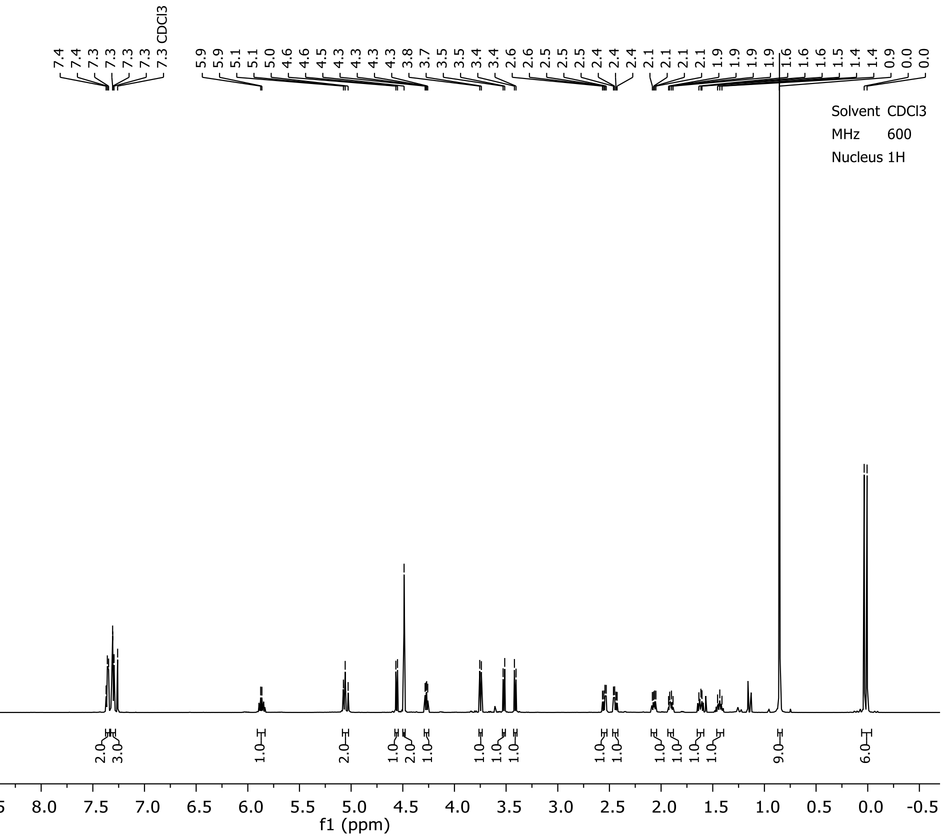
7.4 7.4 7.3 7.3 7.3 7.3 7.3 CDCl3
5.9 5.9 5.1 5.1 5.0 4.6 4.6 4.5 4.3 4.3 4.3 4.3 3.8 3.7 3.5 3.5 3.4 3.4 2.6 2.5 2.5 2.5 2.4 2.4 2.4 2.1 2.1 2.1 2.1 1.9 1.9 1.9 1.9 1.6 1.6 1.6 1.5 1.4 1.4 0.9 0.0 0.0
Solvent CDCl3
MHz 600
Nucleus 1H
2.0 3.0 1.0 2.0 1.0 2.0 1.0 1.0 1.0 1.0 1.0 1.0 1.0 1.0 1.0 1.0 1.0 9.0 6.0
10.5 10.0 9.5 9.0 8.5 8.0 7.5 7.0 6.5 6.0 5.5 5.0 4.5 4.0 3.5 3.0 2.5 2.0 1.5 1.0 0.5 0.0 -0.5
f1 (ppm)

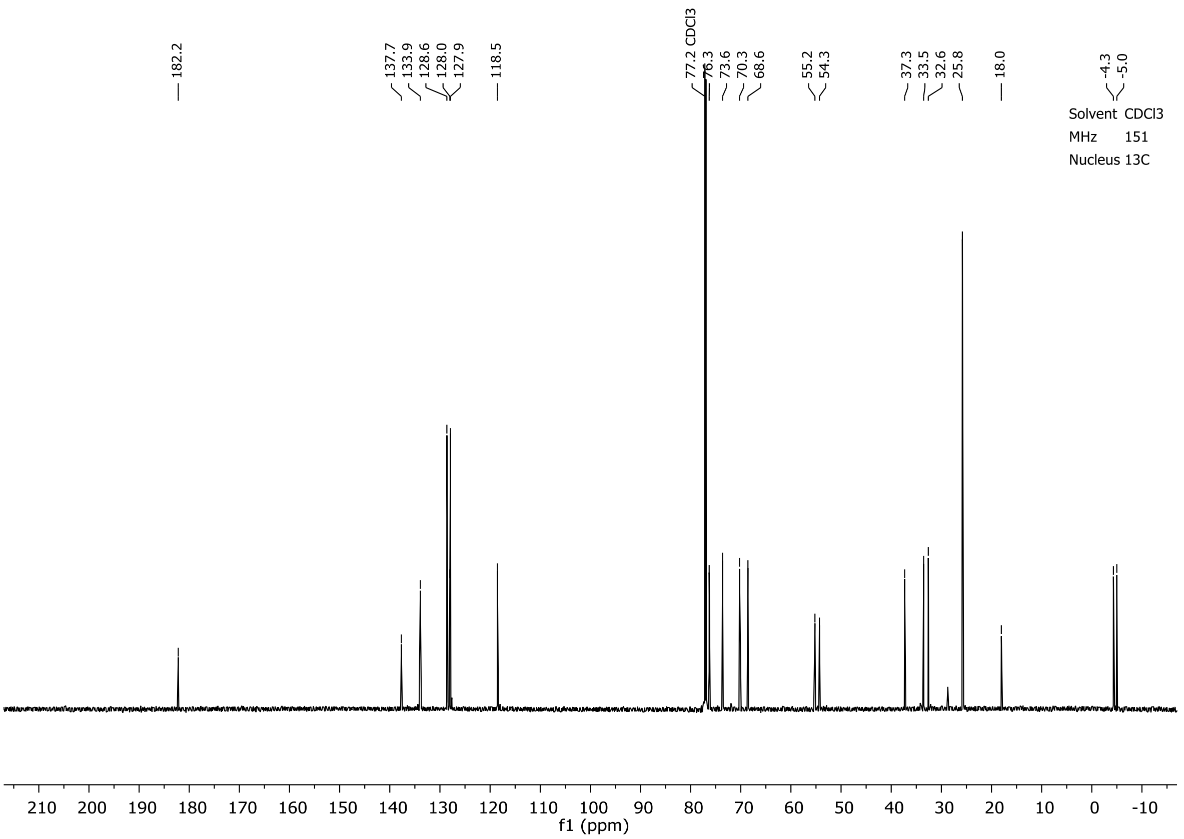
182.2
137.7 133.9 128.6 128.0 127.9
118.5
77.2 CDCl3
76.3 73.6 70.3 68.6
55.2 54.3
37.3 33.5 32.6 25.8
18.0
-4.3 -5.0
Solvent CDCl3
MHz 151
Nucleus 13C
210 200 190 180 170 160 150 140 130 120 110 100 90 80 70 60 50 40 30 20 10 0 -10
f1 (ppm)

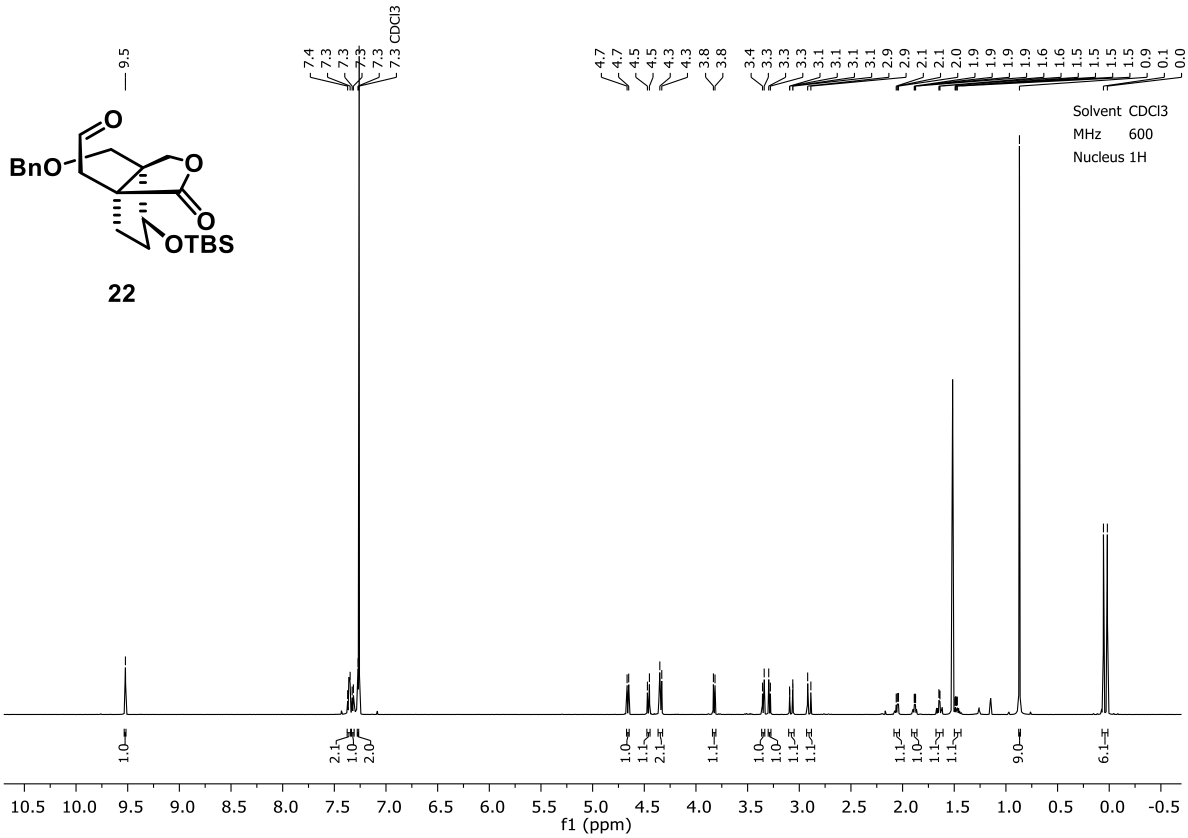
Solvent CDCl3
MHz 600
Nucleus 1H
f1 (ppm)

22

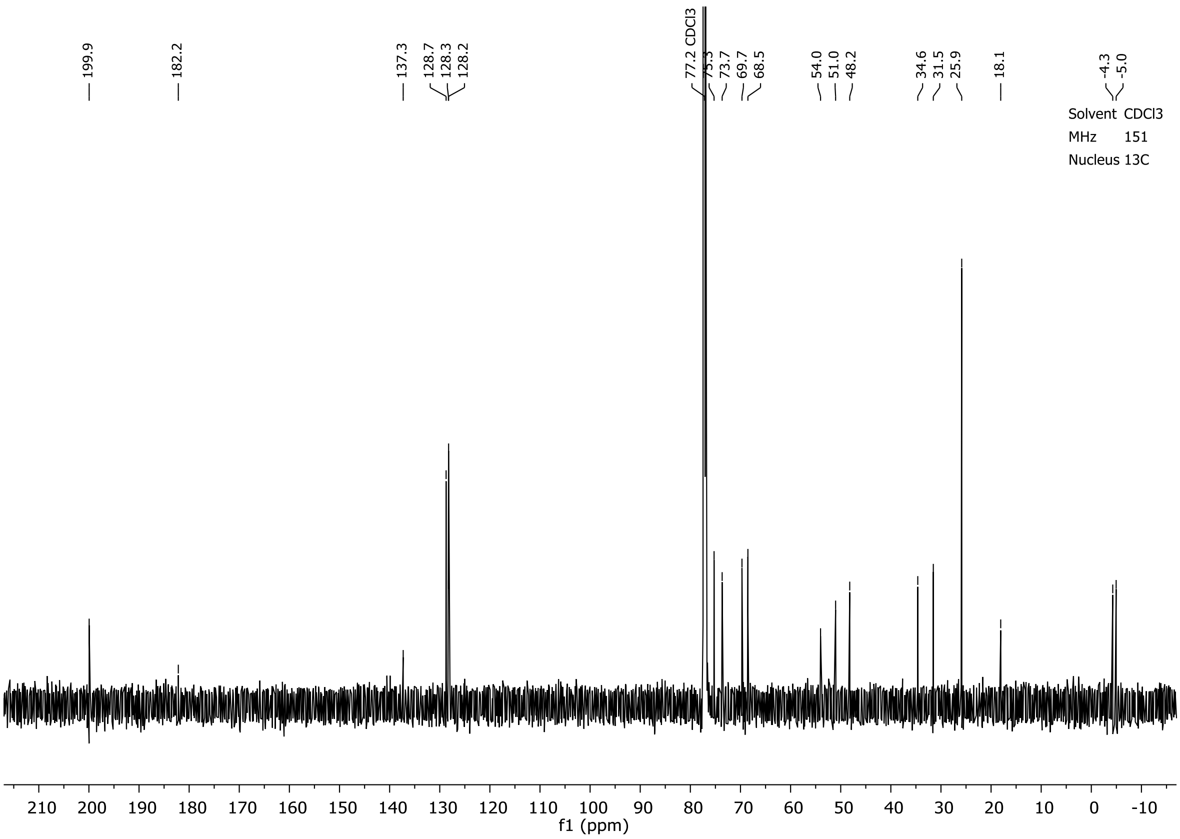
Solvent CDCl3
MHz 151
Nucleus 13C
f1 (ppm)

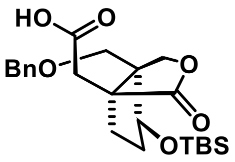
HO
O
BnO
O
O
OTBS

23

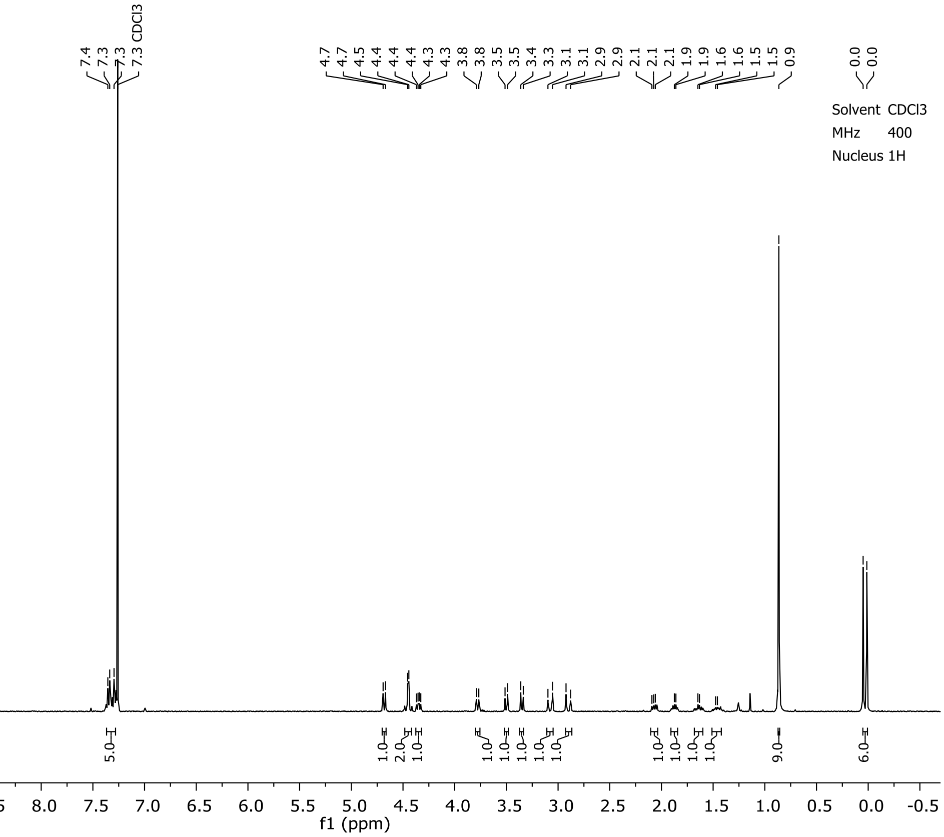
7.4
7.3
7.3
7.3 CDCl3
4.7
4.7
4.5
4.4
4.4
4.4
4.4
4.3
4.3
3.8
3.8
3.5
3.5
3.4
3.3
3.3
3.1
3.1
2.9
2.9
2.1
2.1
2.1
1.9
1.9
1.6
1.6
1.5
1.5
0.9
0.0
0.0
Solvent CDCl3
MHz 400
Nucleus 1H
5.0
1.0
2.0
1.0
1.0
1.0
1.0
1.0
1.0
1.0
1.0
1.0
1.0
1.0
9.0
6.0
10.5 10.0 9.5 9.0 8.5 8.0 7.5 7.0 6.5 6.0 5.5 5.0 4.5 4.0 3.5 3.0 2.5 2.0 1.5 1.0 0.5 0.0 -0.5
f1 (ppm)

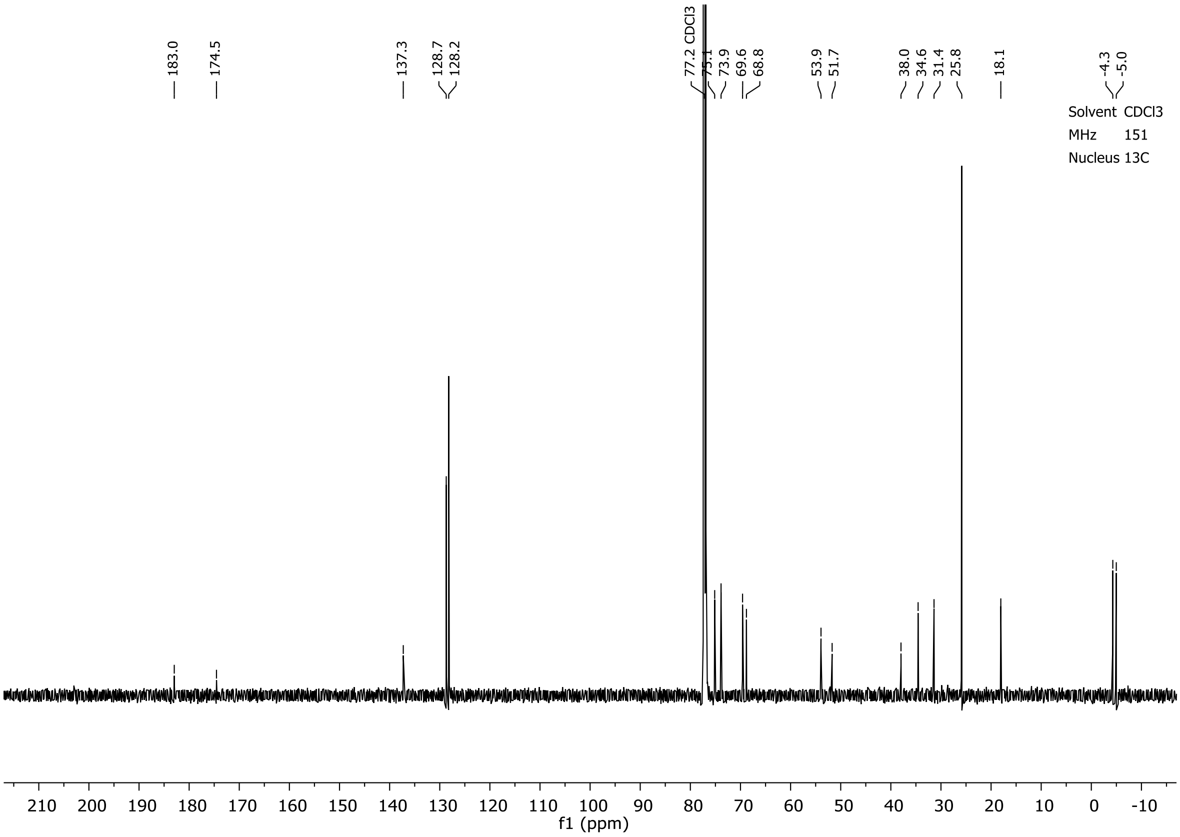
183.0
174.5
137.3
128.7
128.2
77.2 CDCl3
75.1
73.9
69.6
68.8
53.9
51.7
38.0
34.6
31.4
25.8
18.1
-4.3
-5.0
Solvent CDCl3
MHz 151
Nucleus 13C
210 200 190 180 170 160 150 140 130 120 110 100 90 80 70 60 50 40 30 20 10 0 -10
f1 (ppm)

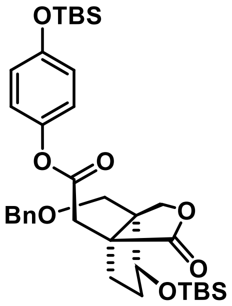
OTBS
O
O
BnO
O
O
OTBS

25

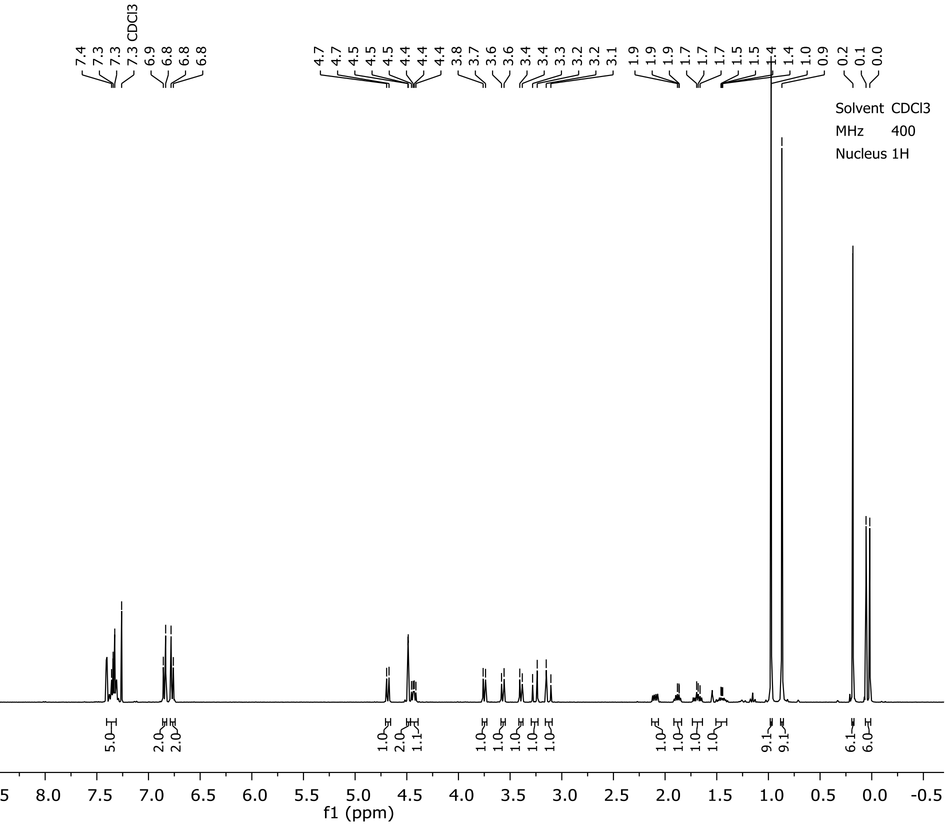
7.4 7.3 7.3 7.3 CDCl3 6.9 6.8 6.8 6.8
4.7 4.7 4.5 4.5 4.5 4.4 4.4 4.4 3.8 3.7 3.6 3.6 3.4 3.4 3.3 3.2 3.2 3.1
1.9 1.9 1.9 1.7 1.7 1.7 1.5 1.5 1.4 1.4 1.0 0.9 0.2 0.1 0.0
Solvent CDCl3
MHz 400
Nucleus 1H
5.0 2.0 2.0 1.0 2.0 1.1 1.0 1.0 1.0 1.0 1.0 1.0 1.0 1.0 1.0 9.1 9.1 6.1 6.0
10.5 10.0 9.5 9.0 8.5 8.0 7.5 7.0 6.5 6.0 5.5 5.0 4.5 4.0 3.5 3.0 2.5 2.0 1.5 1.0 0.5 0.0 -0.5
f1 (ppm)

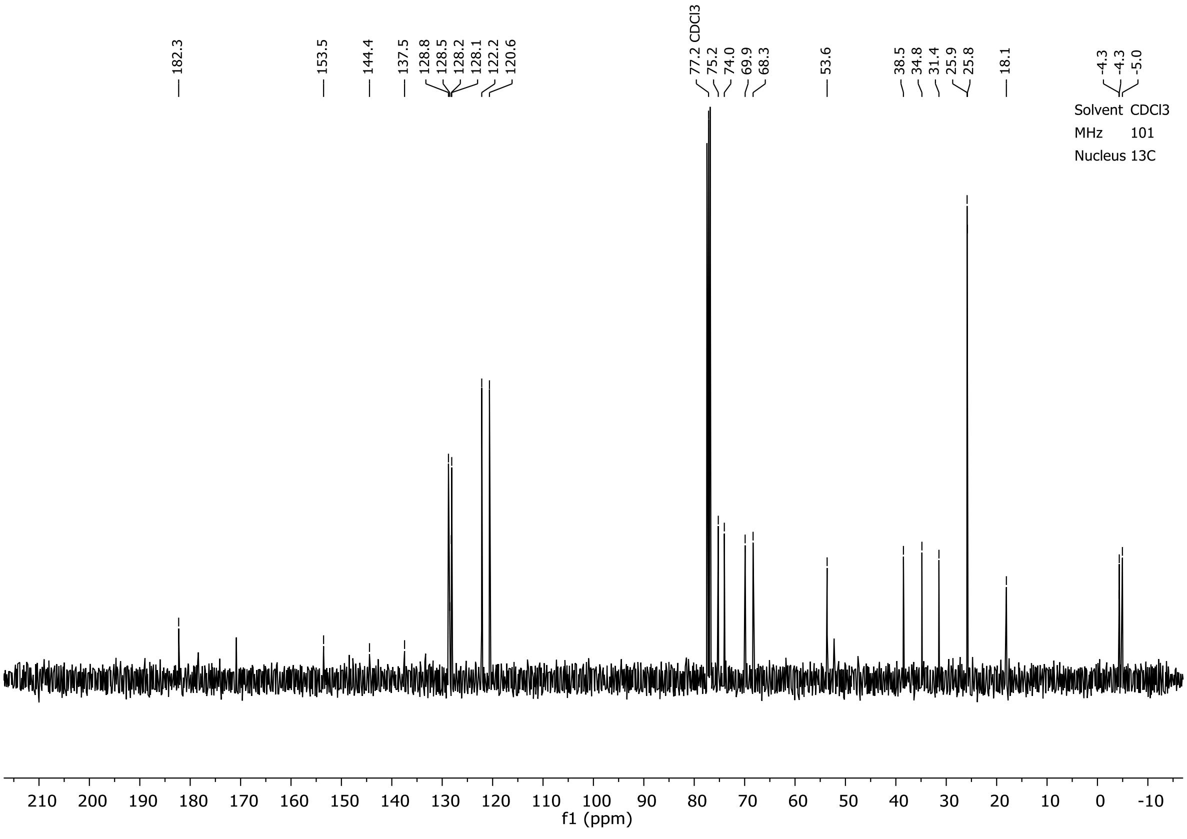
182.3
153.5
144.4
137.5 128.8 128.5 128.2 128.1 122.2 120.6
77.2 CDCl3 75.2 74.0 69.9 68.3
53.6
38.5 34.8 31.4 25.9 25.8
18.1
-4.3 -4.3 -5.0
Solvent CDCl3
MHz 101
Nucleus 13C
210 200 190 180 170 160 150 140 130 120 110 100 90 80 70 60 50 40 30 20 10 0 -10
f1 (ppm)

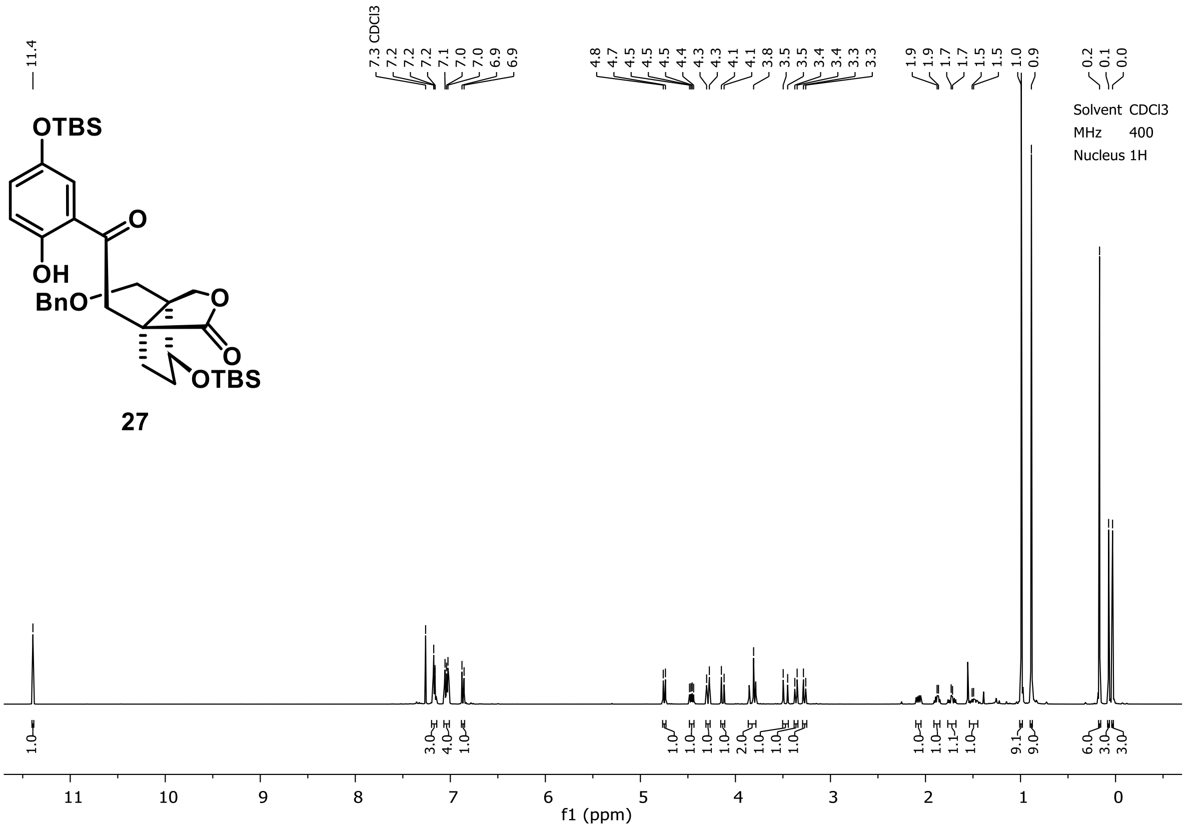
OTBS
O
OH
BnO
O
O
OTBS
27
11.4
7.3 CDCl3
7.2
7.2
7.2
7.2
7.1
7.0
7.0
6.9
6.9
4.8
4.7
4.5
4.5
4.5
4.4
4.3
4.3
4.1
4.1
3.8
3.5
3.5
3.4
3.4
3.3
3.3
1.9
1.9
1.7
1.7
1.5
1.5
1.0
0.9
0.2
0.1
0.0
Solvent CDCl3
MHz 400
Nucleus 1H
1.0
3.0
4.0
1.0
1.0
1.0
1.0
1.0
2.0
1.0
1.0
1.0
1.0
1.0
1.1
1.0
9.1
9.0
6.0
3.0
3.0
11
10
9
8
7
6
5
4
3
2
1
0
f1 (ppm)

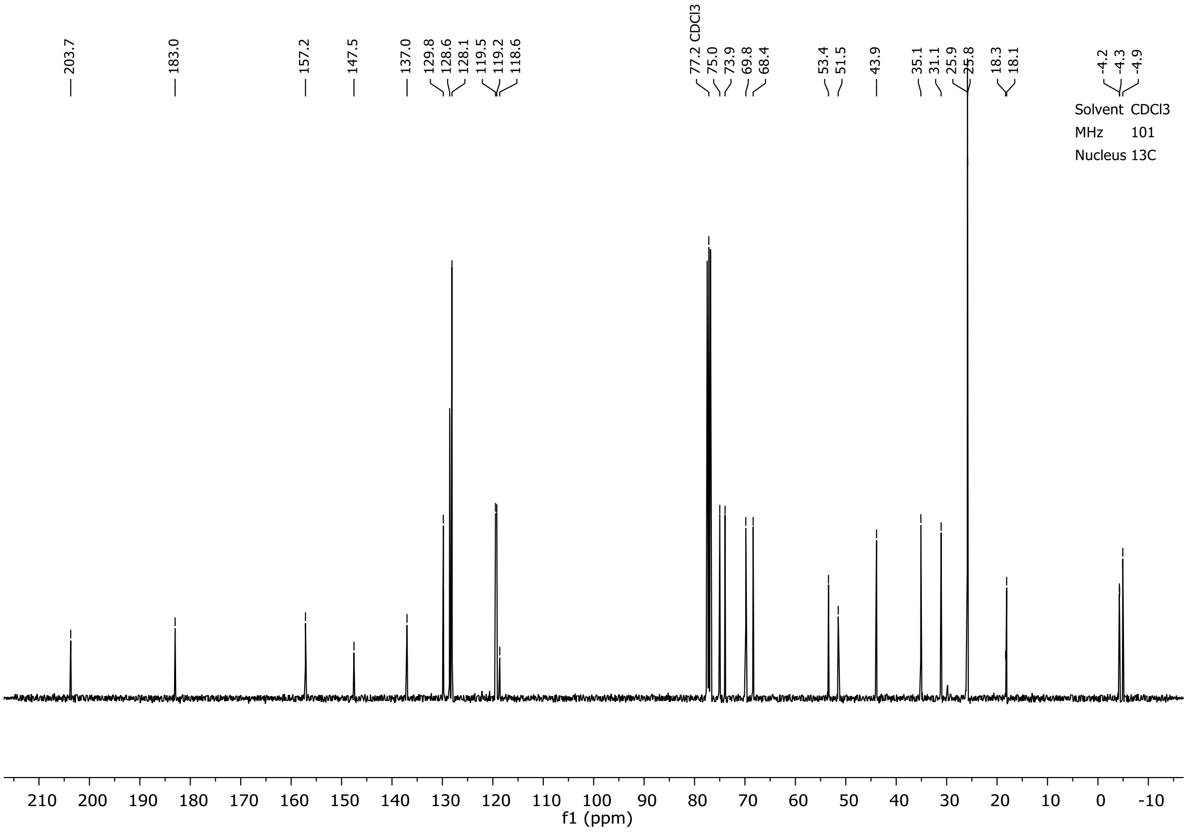
203.7
183.0
157.2
147.5
137.0
129.8
128.6
128.1
119.5
119.2
118.6
77.2 CDCl3
75.0
73.9
69.8
68.4
53.4
51.5
43.9
35.1
31.1
25.9
25.8
18.3
18.1
-4.2
-4.3
-4.9
Solvent CDCl3
MHz 101
Nucleus 13C
210
200
190
180
170
160
150
140
130
120
110
100
90
80
70
60
50
40
30
20
10
0
-10
f1 (ppm)

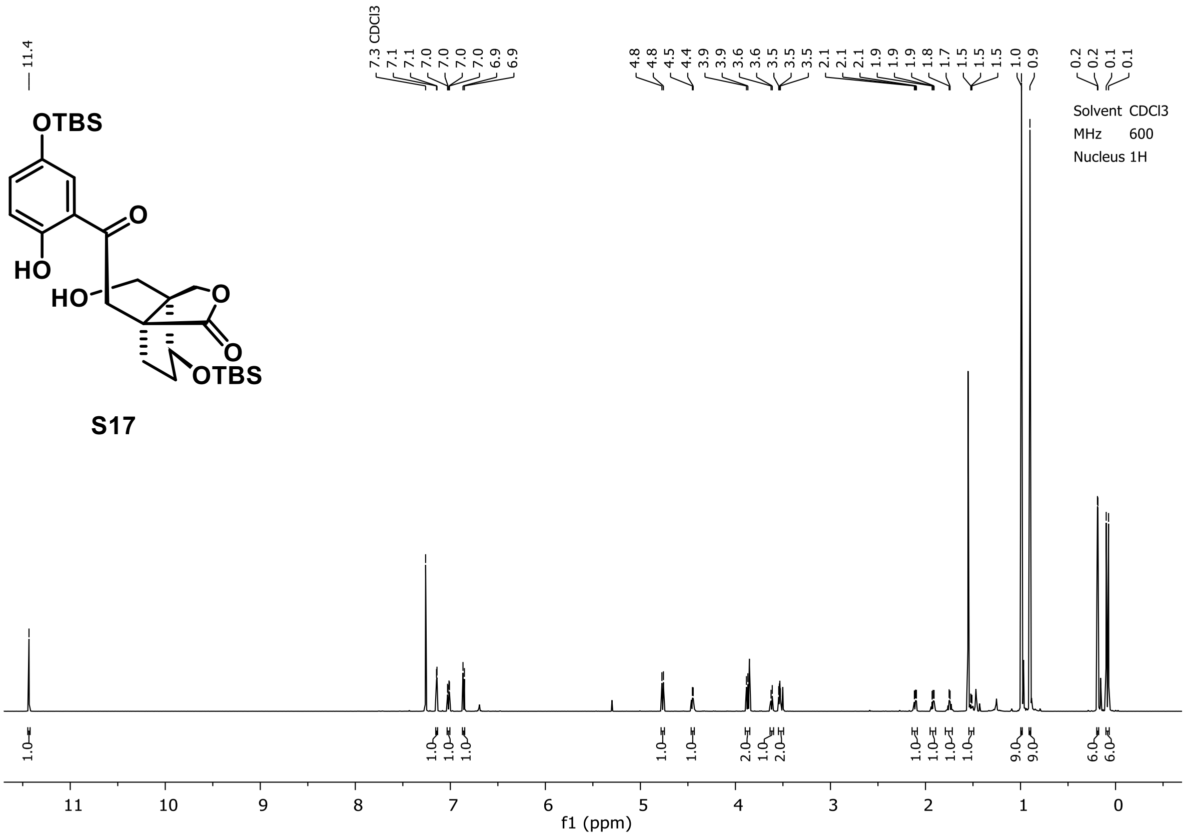

S17

11.4
7.3 CDCl3
7.1
7.1
7.0
7.0
7.0
7.0
6.9
6.9
4.8
4.8
4.5
4.4
3.9
3.9
3.6
3.6
3.5
3.5
3.5
2.1
2.1
2.1
1.9
1.9
1.9
1.8
1.7
1.5
1.5
1.5
1.0
0.9
0.2
0.2
0.1
0.1
Solvent CDCl3
MHz 600
Nucleus 1H
1.0
1.0
1.0
1.0
1.0
1.0
2.0
1.0
2.0
1.0
1.0
1.0
1.0
9.0
9.0
6.0
6.0
f1 (ppm)

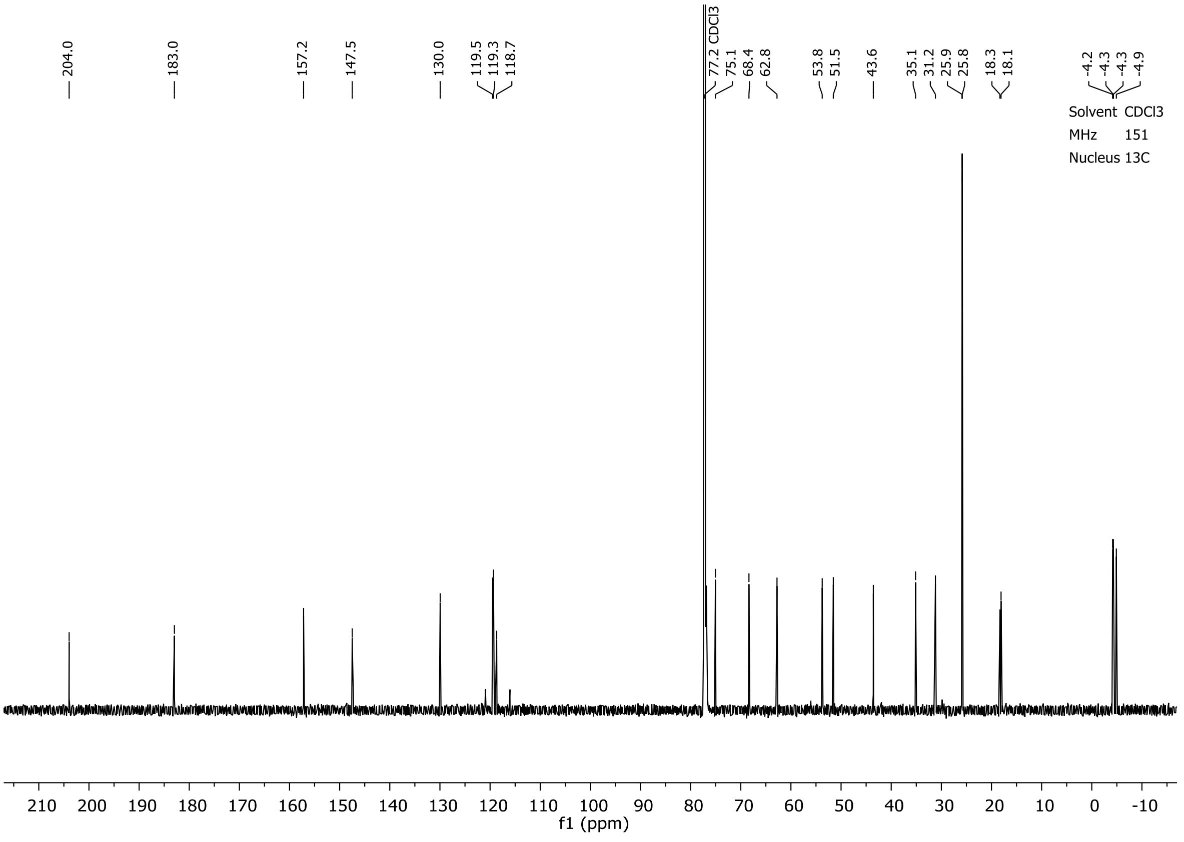
204.0
183.0
157.2
147.5
130.0
119.5
119.3
118.7
77.2 CDCl3
75.1
68.4
62.8
53.8
51.5
43.6
35.1
31.2
25.9
25.8
18.3
18.1
-4.2
-4.3
-4.3
-4.9
Solvent CDCl3
MHz 151
Nucleus 13C
f1 (ppm)

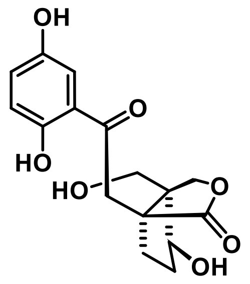

applanatumol H (28)

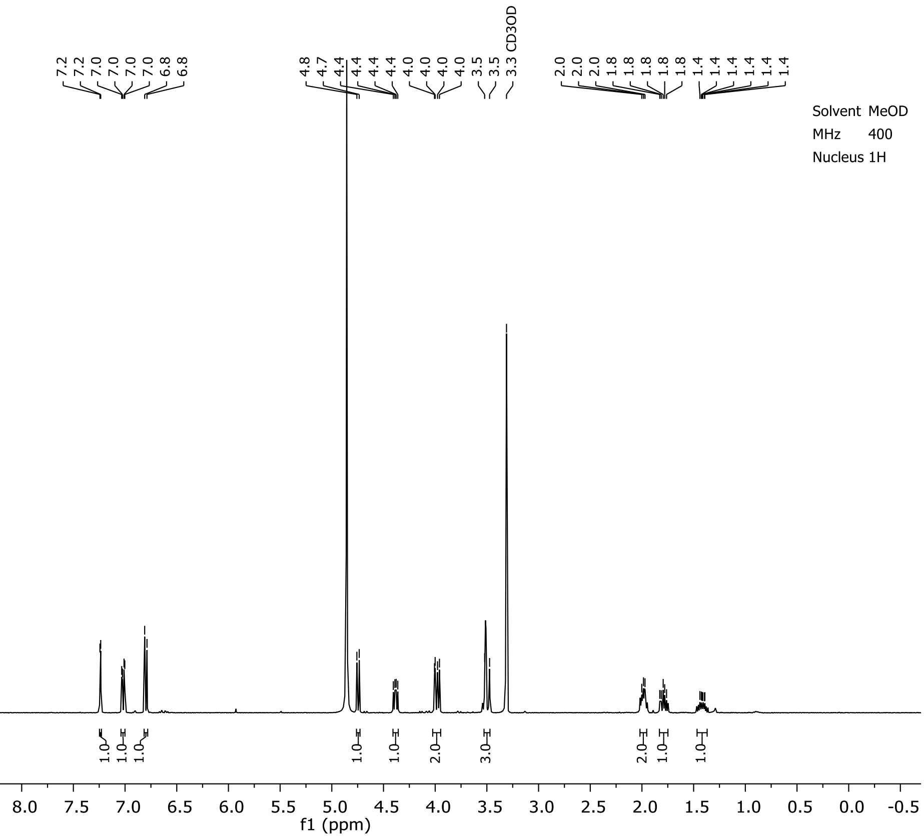

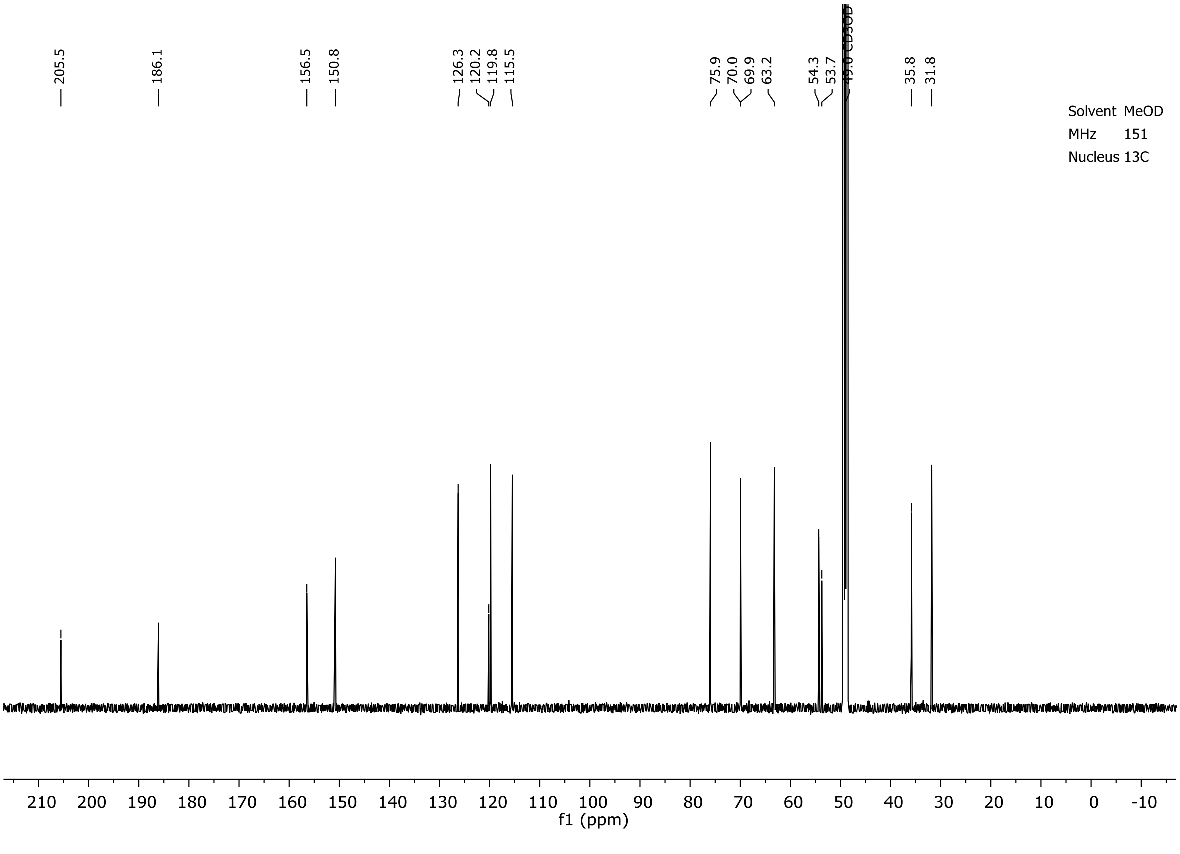

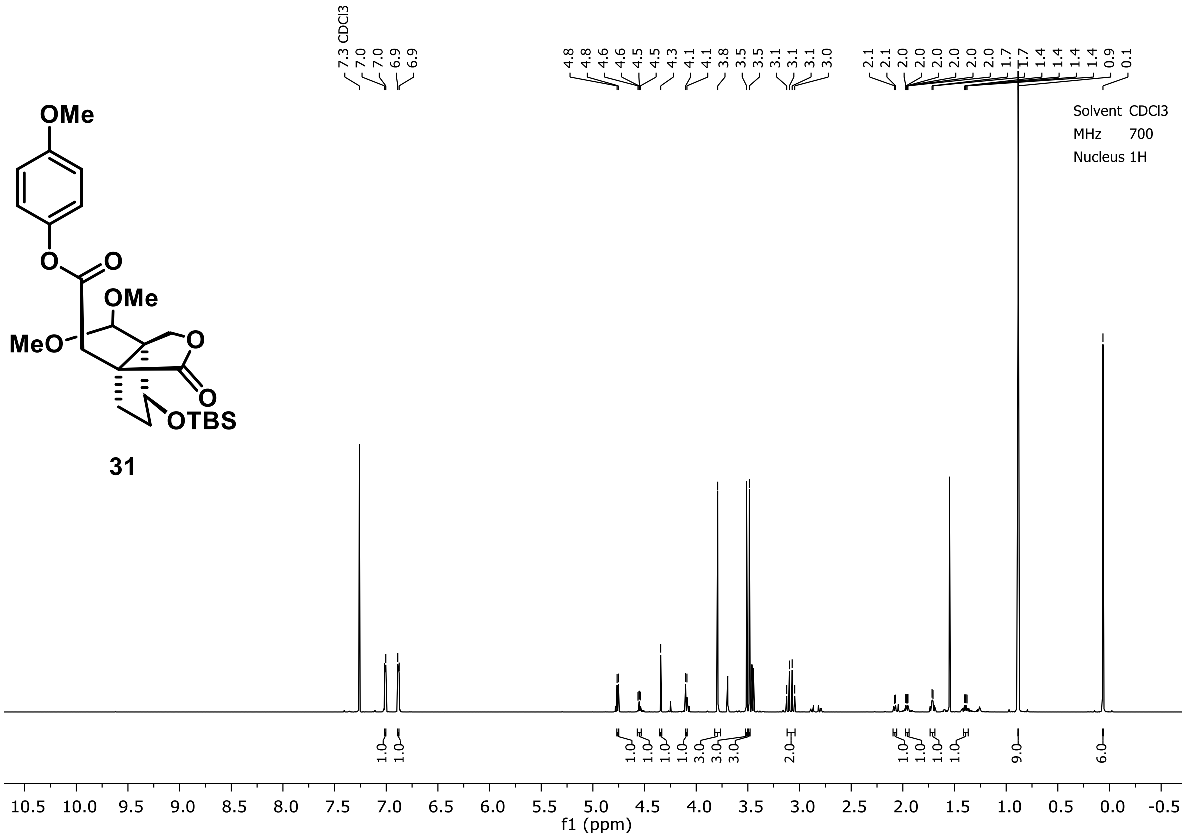
OMe
O
O
OMe
MeO
O
O
OTBS
31
Solvent CDCl3
MHz 700
Nucleus 1H
f1 (ppm)

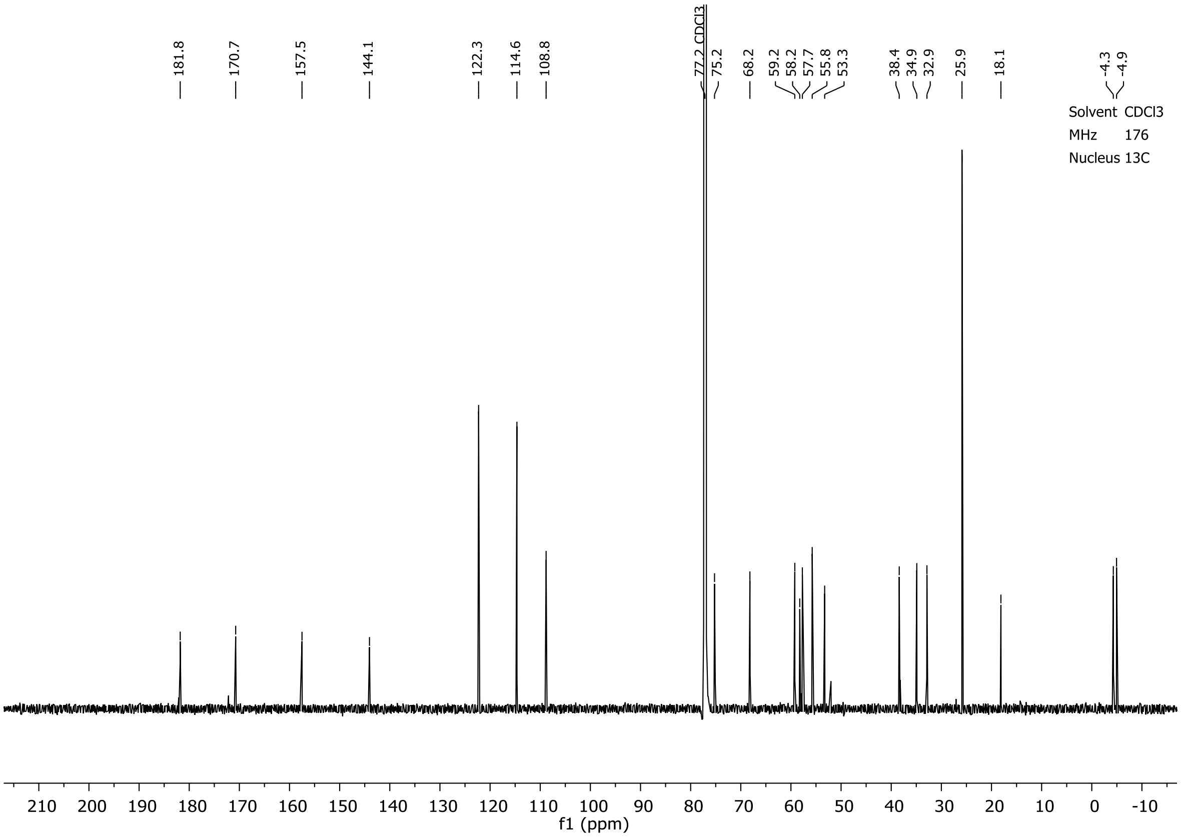
181.8
170.7
157.5
144.1
122.3
114.6
108.8
77.2 CDCl3
75.2
68.2
59.2
58.2
57.7
55.8
53.3
38.4
34.9
32.9
25.9
18.1
-4.3
-4.9
Solvent CDCl3
MHz 176
Nucleus 13C
f1 (ppm)

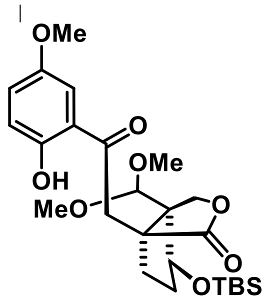
OMe
O
OH
OMe
MeO
O
O
OTBS

32

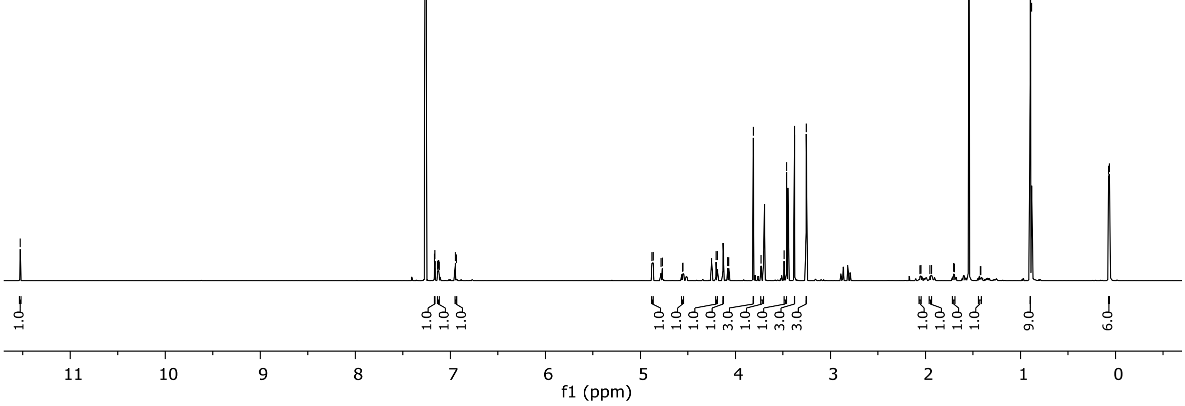
11.5
7.3 CDCl3
Solvent CDCl3
MHz 700
Nucleus 1H
f1 (ppm)

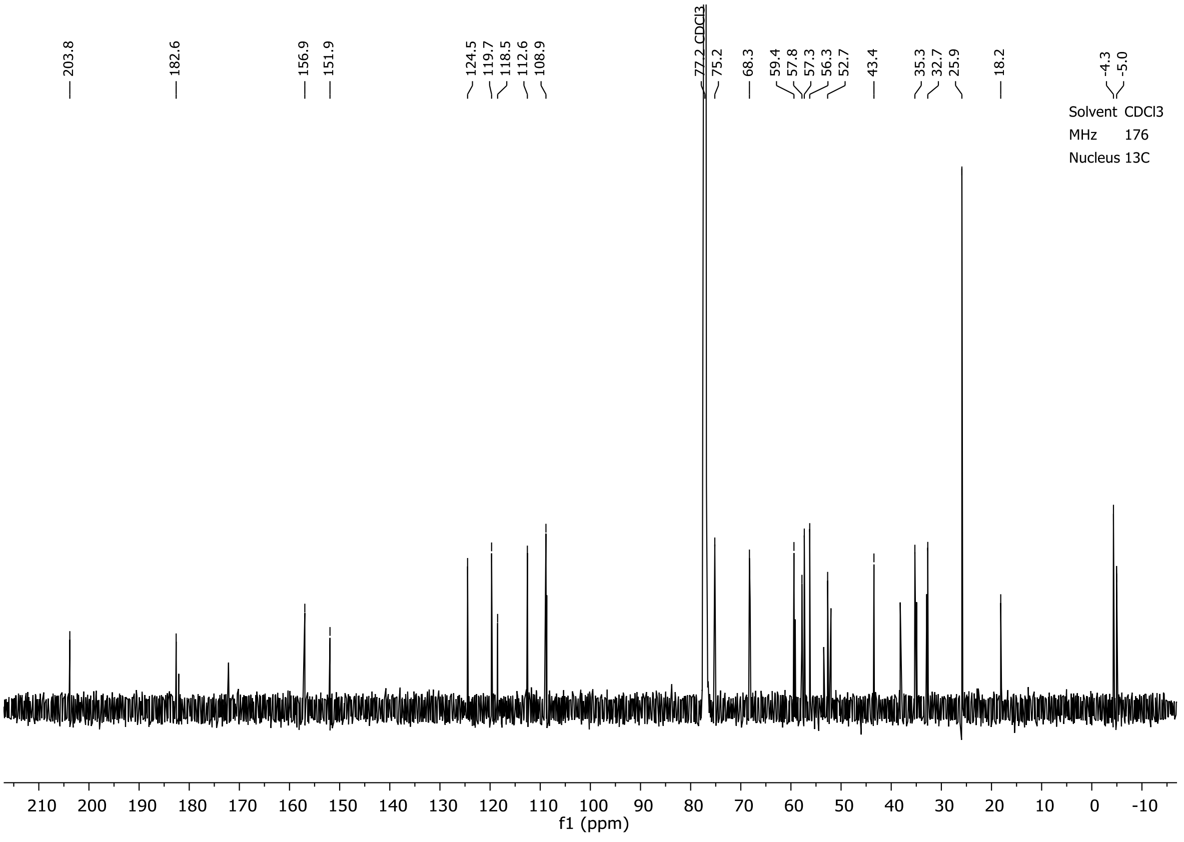
203.8
182.6
156.9
151.9
124.5
119.7
118.5
112.6
108.9
77.2 CDCl3
75.2
68.3
59.4
57.8
57.3
56.3
52.7
43.4
35.3
32.7
25.9
18.2
-4.3
-5.0
Solvent CDCl3
MHz 176
Nucleus 13C
f1 (ppm)

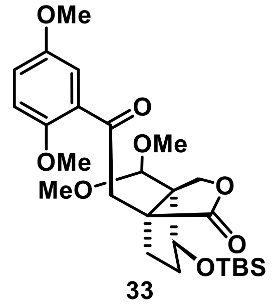
OMe
O
OMe
OMe
MeO
O
O
OTBS

33

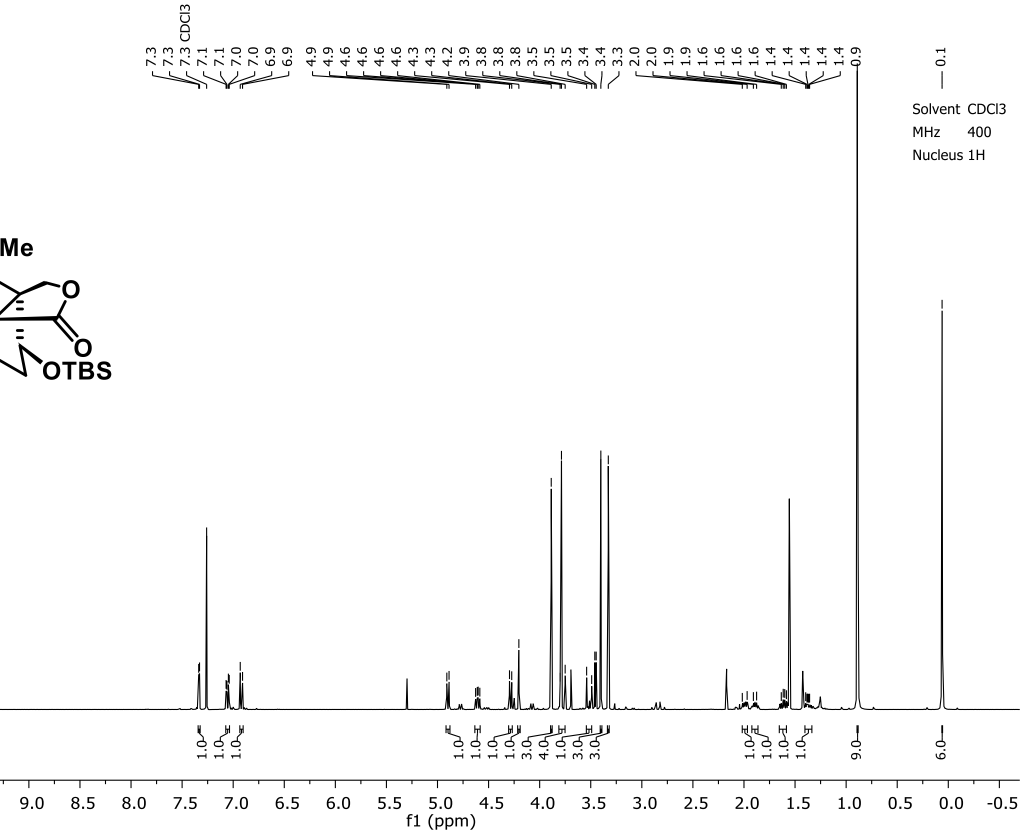
7.3 7.3 7.3 CDCl3 7.1 7.1 7.0 7.0 6.9 6.9 4.9 4.9 4.6 4.6 4.6 4.6 4.3 4.3 4.2 3.9 3.8 3.8 3.8 3.5 3.5 3.5 3.4 3.4 3.3 2.0 2.0 1.9 1.9 1.6 1.6 1.6 1.6 1.4 1.4 1.4 1.4 1.4 0.9 0.1
Solvent CDCl3
MHz 400
Nucleus 1H
1.0 1.0 1.0 1.0 1.0 1.0 3.0 4.0 1.0 3.0 3.0 1.0 1.0 1.0 1.0 9.0 6.0
10.5 10.0 9.5 9.0 8.5 8.0 7.5 7.0 6.5 6.0 5.5 5.0 4.5 4.0 3.5 3.0 2.5 2.0 1.5 1.0 0.5 0.0 -0.5
f1 (ppm)

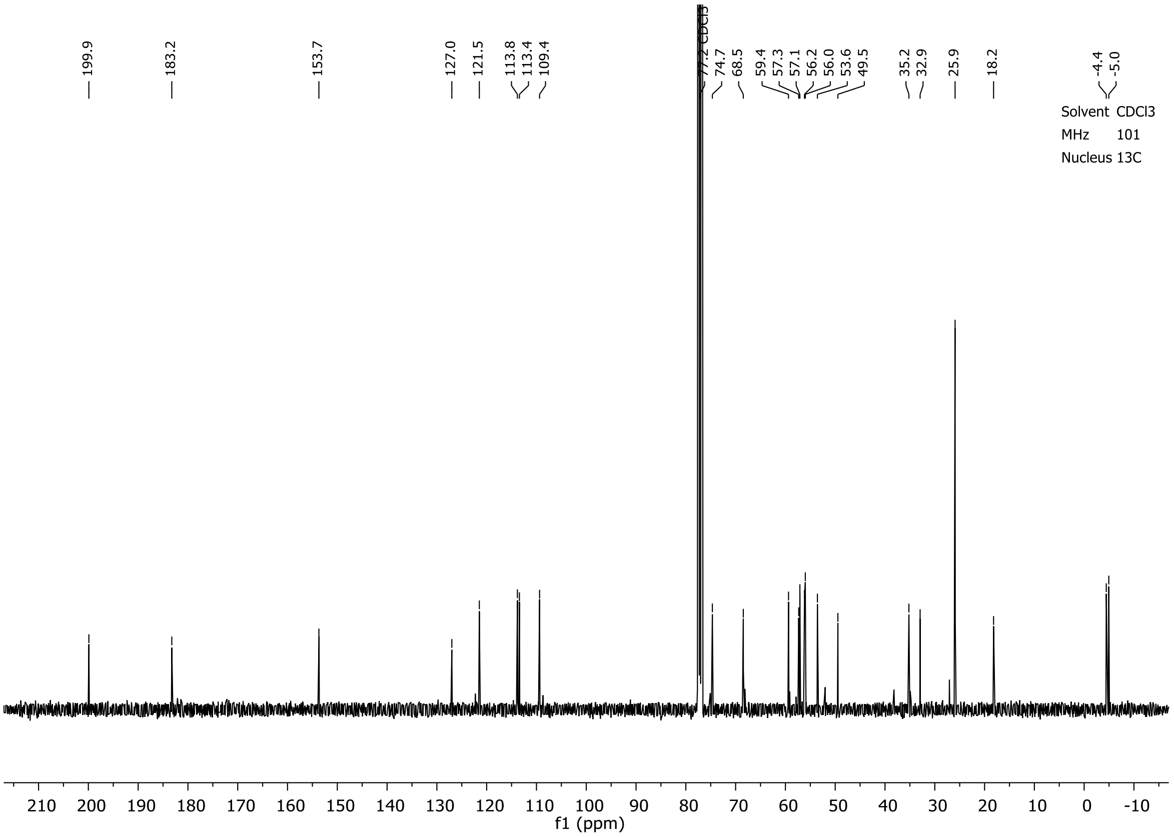
199.9 183.2 153.7 127.0 121.5 113.8 113.4 109.4 77.2 CDCl3 74.7 68.5 59.4 57.3 57.1 56.2 56.0 53.6 49.5 35.2 32.9 25.9 18.2 -4.4 -5.0
Solvent CDCl3
MHz 101
Nucleus 13C
210 200 190 180 170 160 150 140 130 120 110 100 90 80 70 60 50 40 30 20 10 0 -10
f1 (ppm)

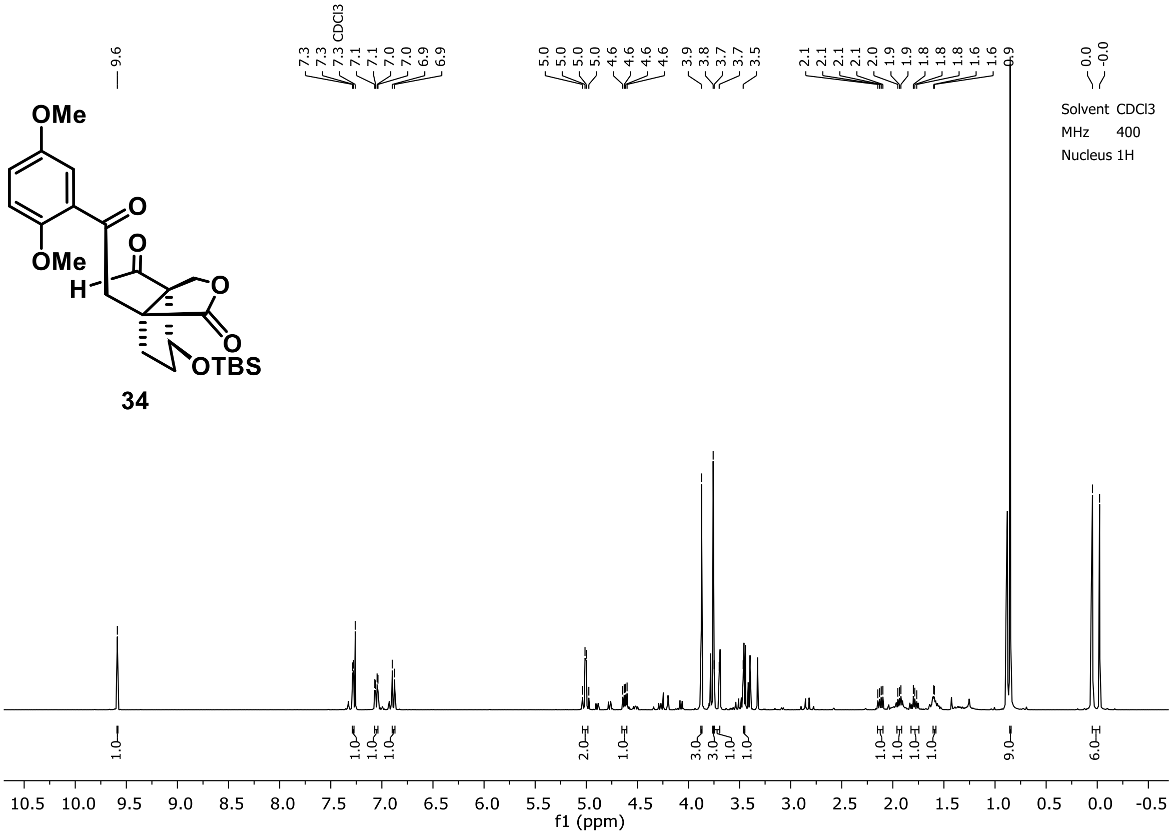
OMe
O
O
OMe
H
O
O
OTBS
34
9.6
7.3
7.3
7.3 CDCl3
7.1
7.1
7.0
7.0
6.9
6.9
5.0
5.0
5.0
5.0
4.6
4.6
4.6
4.6
3.9
3.8
3.7
3.7
3.5
2.1
2.1
2.1
2.1
2.0
1.9
1.9
1.8
1.8
1.8
1.6
1.6
0.9
0.0
-0.0
Solvent CDCl3
MHz 400
Nucleus 1H
1.0
1.0
1.0
1.0
2.0
1.0
3.0
3.0
1.0
1.0
1.0
1.0
1.0
1.0
9.0
6.0
10.5 10.0 9.5 9.0 8.5 8.0 7.5 7.0 6.5 6.0 5.5 5.0 4.5 4.0 3.5 3.0 2.5 2.0 1.5 1.0 0.5 0.0 -0.5
f1 (ppm)

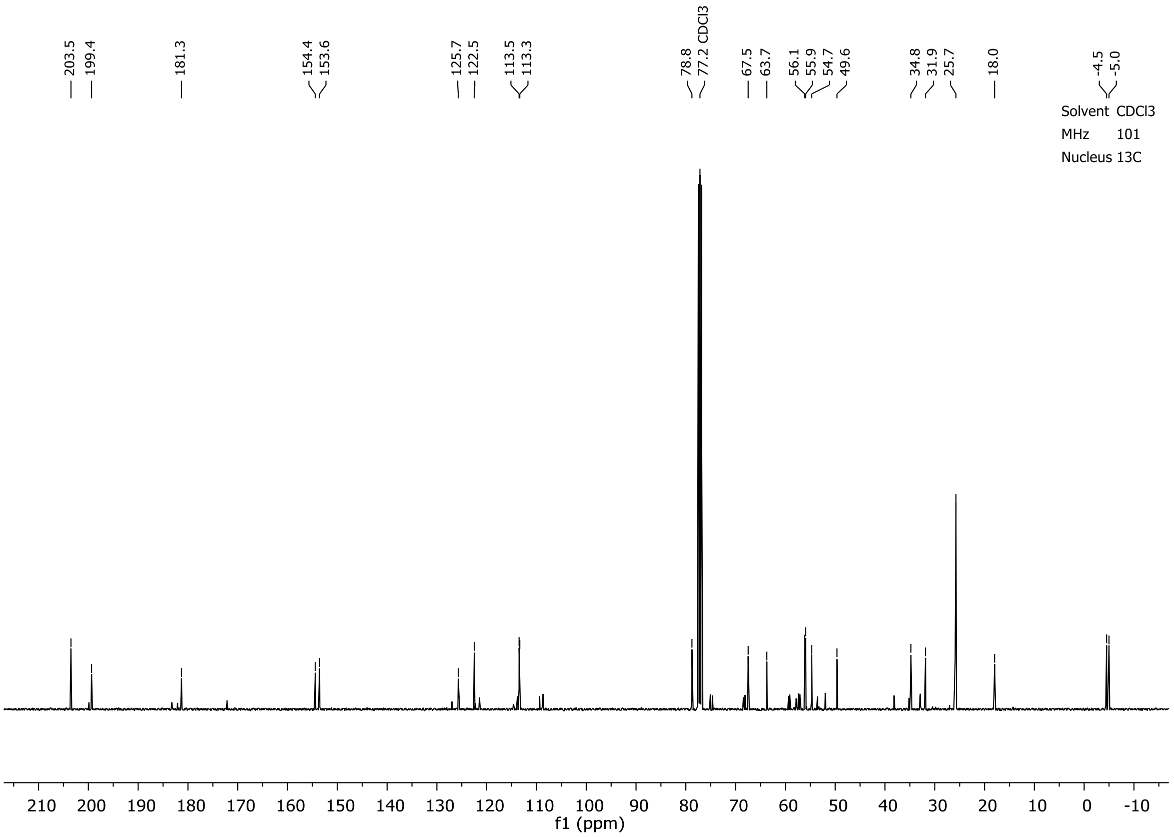
203.5
199.4
181.3
154.4
153.6
125.7
122.5
113.5
113.3
78.8
77.2 CDCl3
67.5
63.7
56.1
55.9
54.7
49.6
34.8
31.9
25.7
18.0
-4.5
-5.0
Solvent CDCl3
MHz 101
Nucleus 13C
210 200 190 180 170 160 150 140 130 120 110 100 90 80 70 60 50 40 30 20 10 0 -10
f1 (ppm)

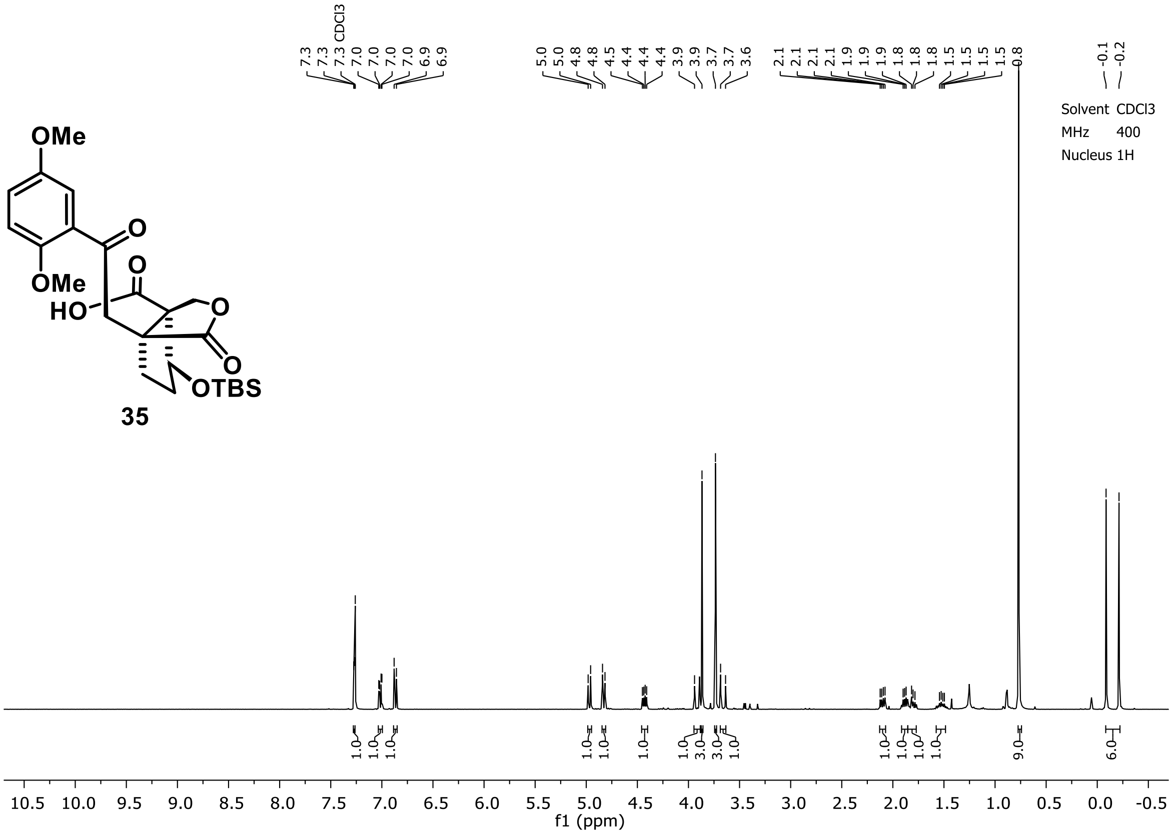
35
Solvent CDCl3
MHz 400
Nucleus 1H
f1 (ppm)

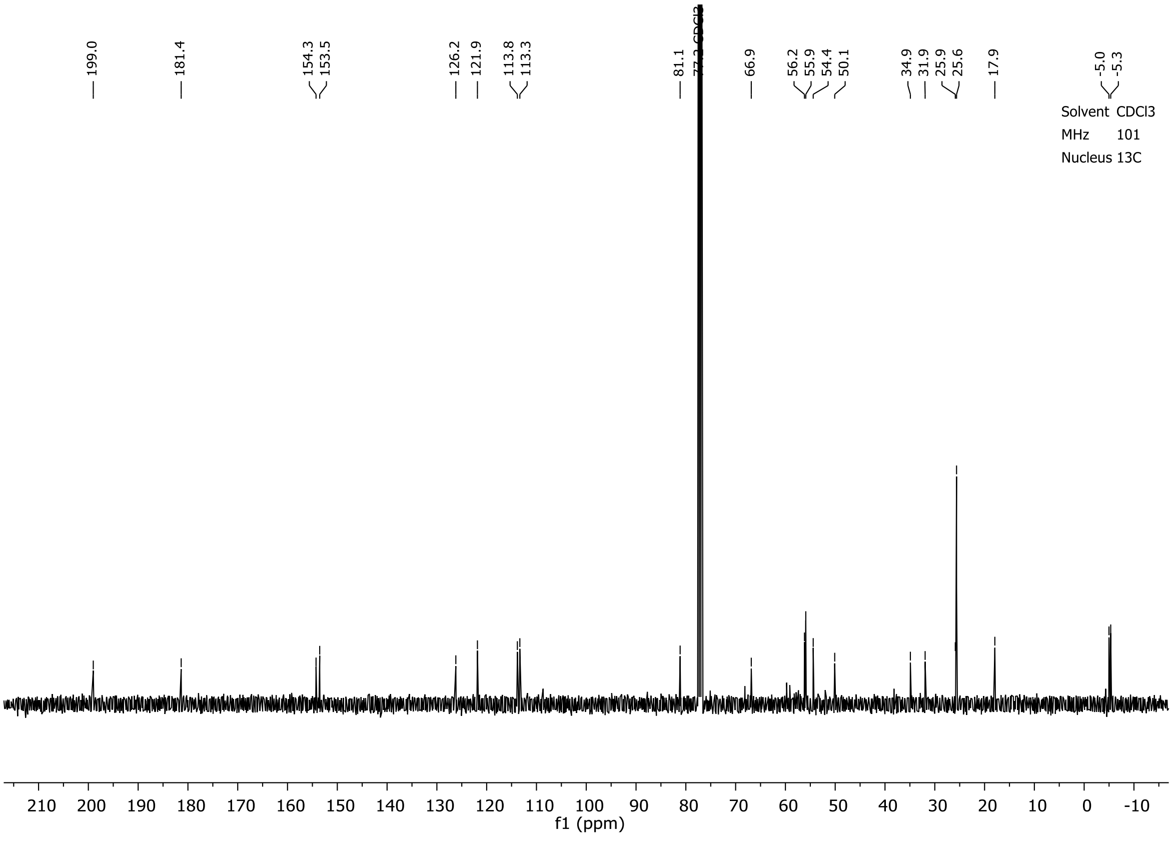
Solvent CDCl3
MHz 101
Nucleus 13C
f1 (ppm)

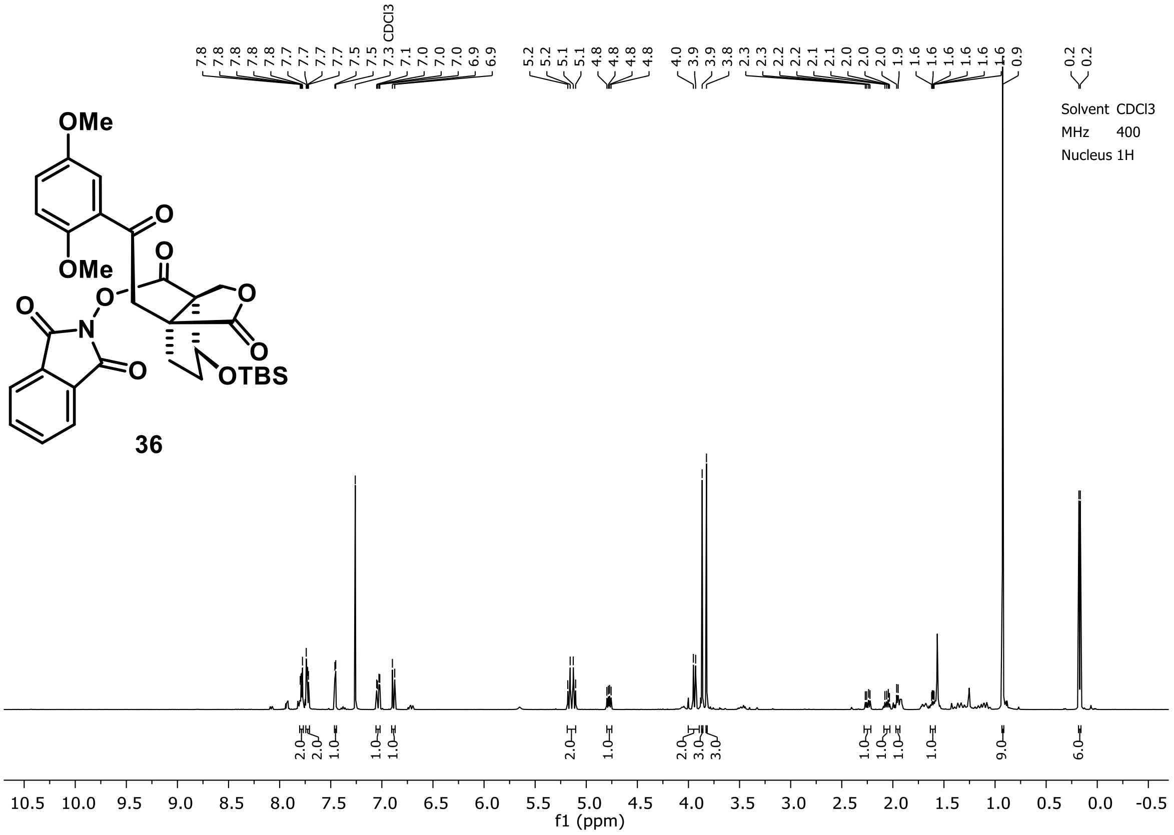
OMe
O
O
OMe
O
N
O
O
O
O
OTBS
36
7.8 7.8 7.8 7.8 7.8 7.7 7.7 7.7 7.7 7.5 7.5 7.3 CDCl3 7.1 7.0 7.0 7.0 7.0 6.9 6.9
5.2 5.2 5.1 5.1 4.8 4.8 4.8 4.8 4.8
4.0 3.9 3.9 3.8 2.3 2.3 2.2 2.2 2.2 2.1 2.1 2.0 2.0 2.0 2.0 1.9 1.6 1.6 1.6 1.6 1.6 1.6 0.9
0.2 0.2
Solvent CDCl3
MHz 400
Nucleus 1H
2.0 2.0 1.0 1.0 1.0 2.0 1.0 2.0 3.0 3.0 1.0 1.0 1.0 1.0 9.0 6.0
10.5 10.0 9.5 9.0 8.5 8.0 7.5 7.0 6.5 6.0 5.5 5.0 4.5 4.0 3.5 3.0 2.5 2.0 1.5 1.0 0.5 0.0 -0.5
f1 (ppm)

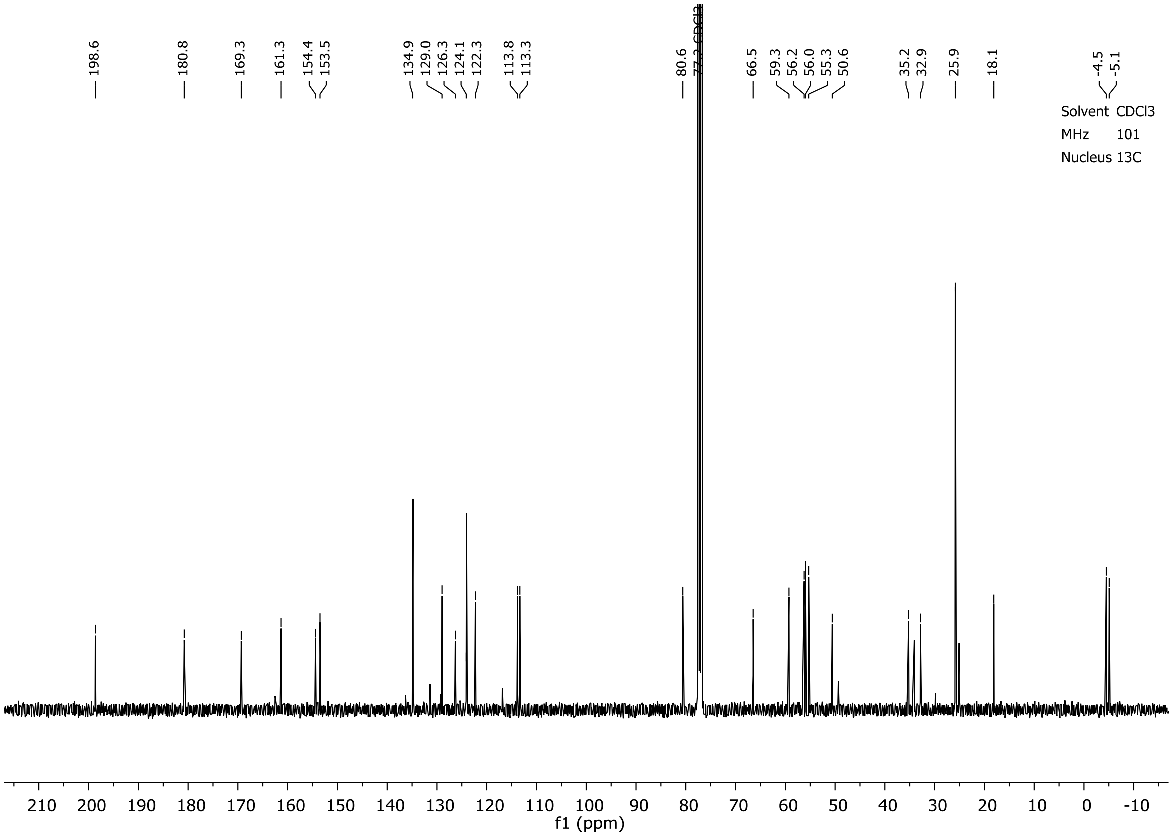
198.6
180.8
169.3
161.3
154.4 153.5
134.9 129.0 126.3 124.1 122.3
113.8 113.3
80.6
77.2 CDCl3
66.5
59.3 56.2 56.0 55.3 50.6
35.2 32.9
25.9
18.1
-4.5 -5.1
Solvent CDCl3
MHz 101
Nucleus 13C
210 200 190 180 170 160 150 140 130 120 110 100 90 80 70 60 50 40 30 20 10 0 -10
f1 (ppm)

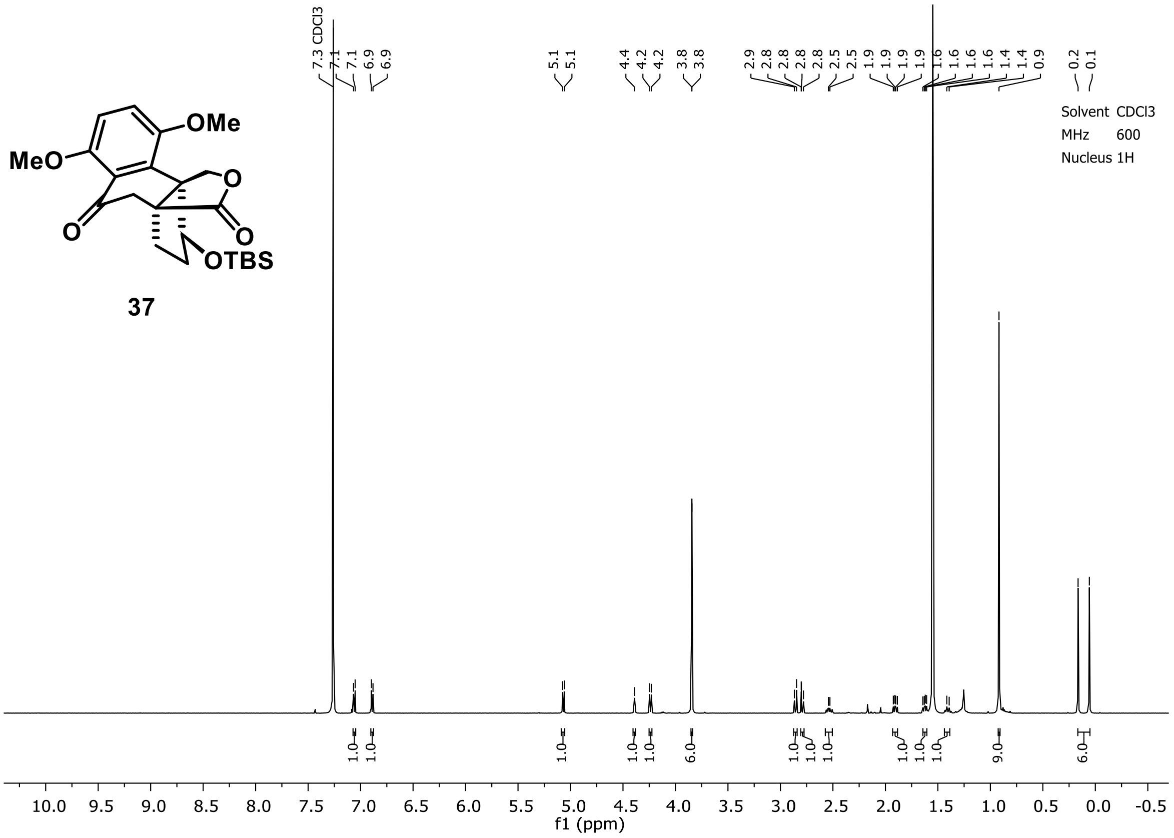
MeO
OMe
O
O
O
OTBS
7.3 CDCl3
7.1
7.1
6.9
6.9
5.1
5.1
4.4
4.2
4.2
3.8
3.8
2.9
2.8
2.8
2.8
2.8
2.5
2.5
1.9
1.9
1.9
1.9
1.6
1.6
1.6
1.4
1.4
0.9
0.2
0.1
Solvent CDCl3
MHz 600
Nucleus 1H
1.0
1.0
1.0
1.0
1.0
6.0
1.0
1.0
1.0
1.0
1.0
1.0
9.0
6.0
10.0 9.5 9.0 8.5 8.0 7.5 7.0 6.5 6.0 5.5 5.0 4.5 4.0 3.5 3.0 2.5 2.0 1.5 1.0 0.5 0.0 -0.5
f1 (ppm)

37

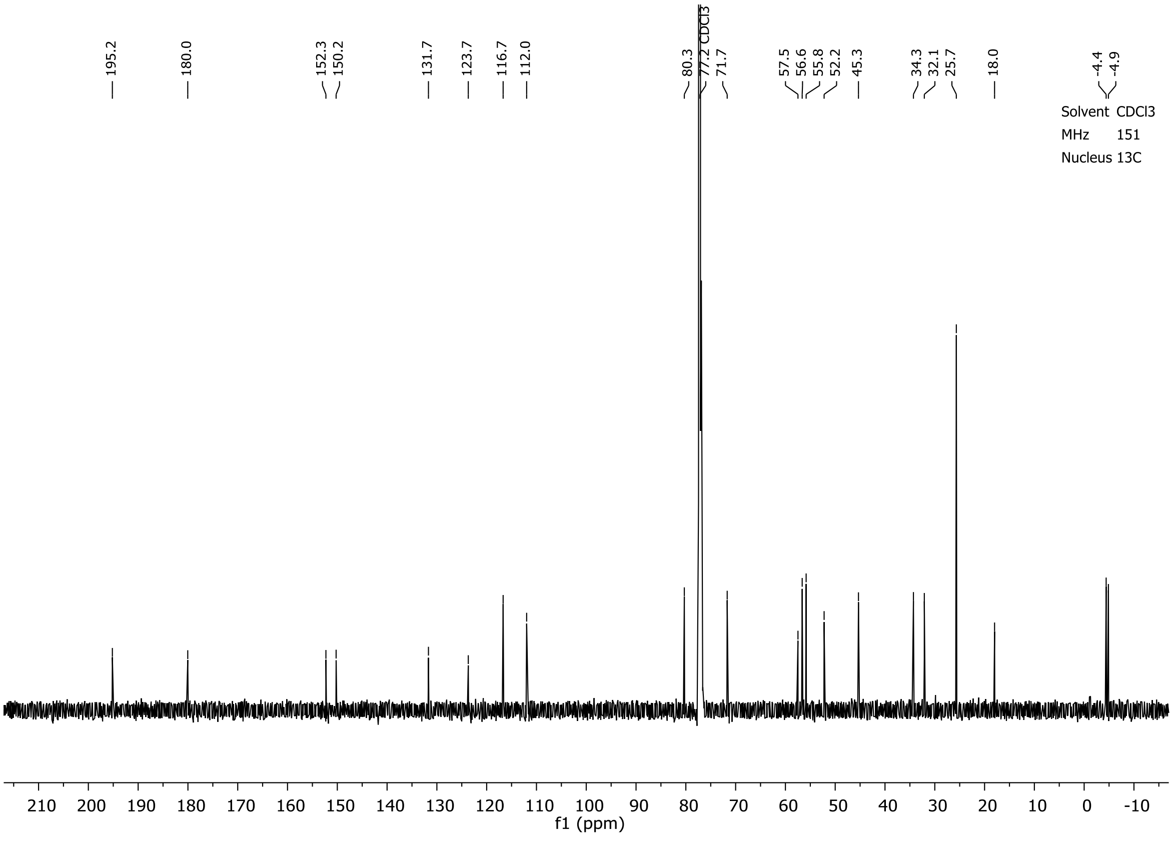
195.2
180.0
152.3
150.2
131.7
123.7
116.7
112.0
80.3
77.2 CDCl3
71.7
57.5
56.6
55.8
52.2
45.3
34.3
32.1
25.7
18.0
-4.4
-4.9
Solvent CDCl3
MHz 151
Nucleus 13C
210 200 190 180 170 160 150 140 130 120 110 100 90 80 70 60 50 40 30 20 10 0 -10
f1 (ppm)

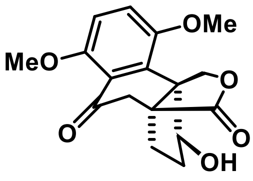
OMe
MeO
O
O
O
OH

S18

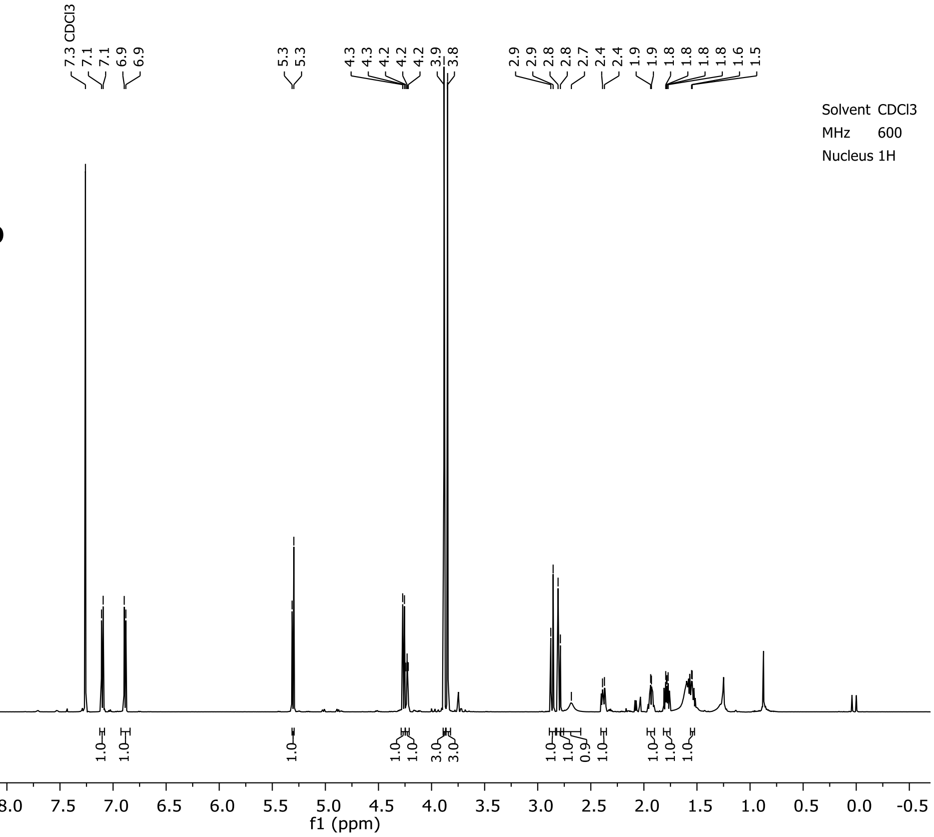
7.3 CDCl3
7.1
7.1
6.9
6.9
5.3
5.3
4.3
4.3
4.2
4.2
4.2
3.9
3.8
2.9
2.9
2.8
2.8
2.7
2.4
2.4
1.9
1.9
1.8
1.8
1.8
1.8
1.6
1.5
Solvent CDCl3
MHz 600
Nucleus 1H
1.0
1.0
1.0
1.0
1.0
3.0
3.0
1.0
1.0
0.9
1.0
1.0
1.0
1.0
10.0 9.5 9.0 8.5 8.0 7.5 7.0 6.5 6.0 5.5 5.0 4.5 4.0 3.5 3.0 2.5 2.0 1.5 1.0 0.5 0.0 -0.5
f1 (ppm)

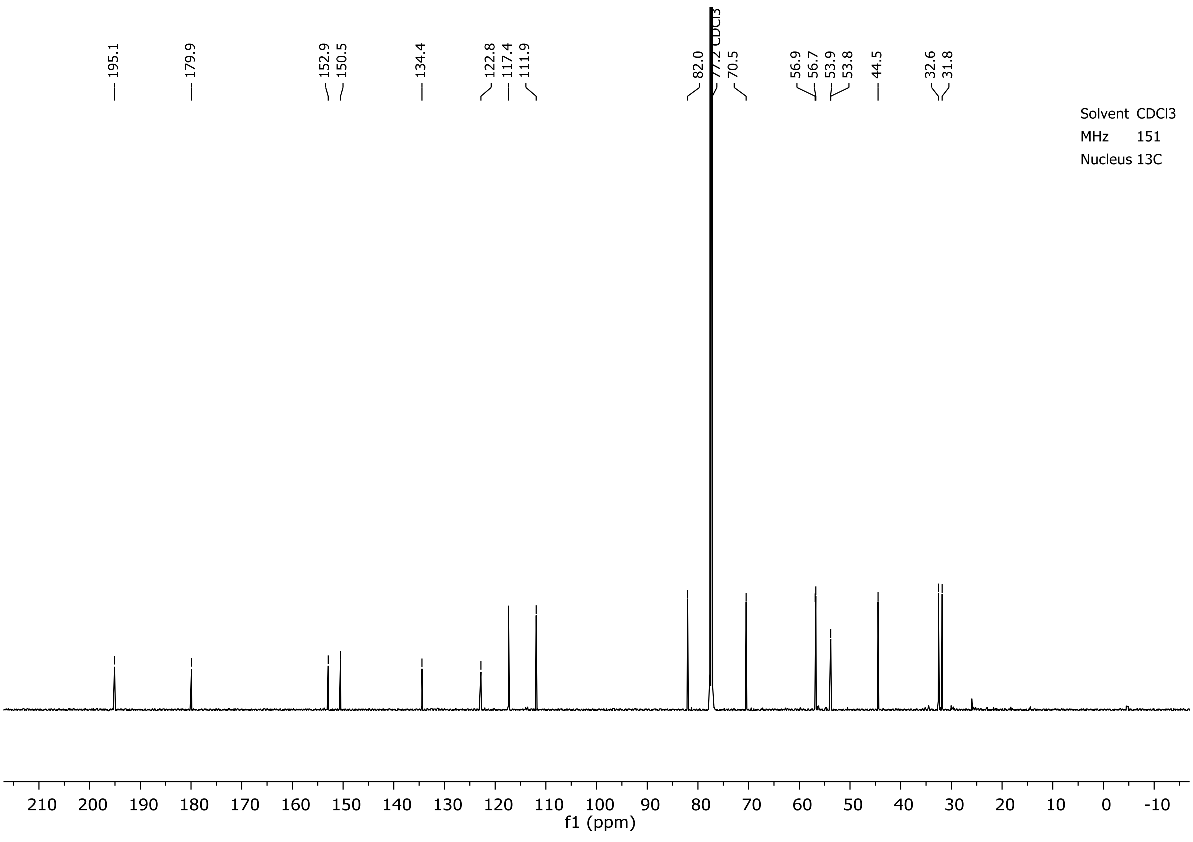
195.1
179.9
152.9
150.5
134.4
122.8
117.4
111.9
82.0
77.2 CDCl3
70.5
56.9
56.7
53.9
53.8
44.5
32.6
31.8
Solvent CDCl3
MHz 151
Nucleus 13C
210 200 190 180 170 160 150 140 130 120 110 100 90 80 70 60 50 40 30 20 10 0 -10
f1 (ppm)

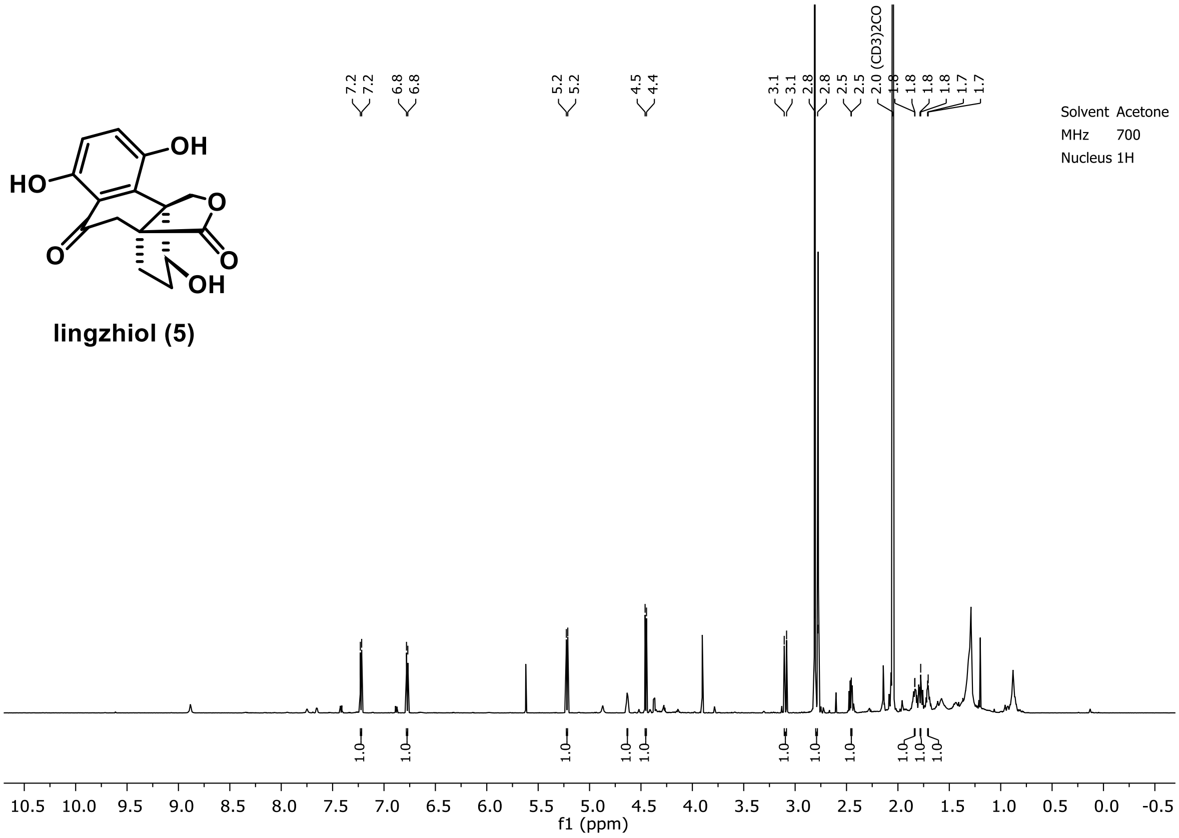
OH
HO
O
O
O
OH
7.2
7.2
6.8
6.8
5.2
5.2
4.5
4.4
3.1
3.1
2.8
2.8
2.5
2.5
2.0 (CD3)2CO
1.8
1.8
1.8
1.7
1.7
Solvent Acetone
MHz 700
Nucleus 1H
1.0
1.0
1.0
1.0
1.0
1.0
1.0
1.0
1.0
1.0
1.0
10.5 10.0 9.5 9.0 8.5 8.0 7.5 7.0 6.5 6.0 5.5 5.0 4.5 4.0 3.5 3.0 2.5 2.0 1.5 1.0 0.5 0.0 -0.5
f1 (ppm)

lingzhiol (5)

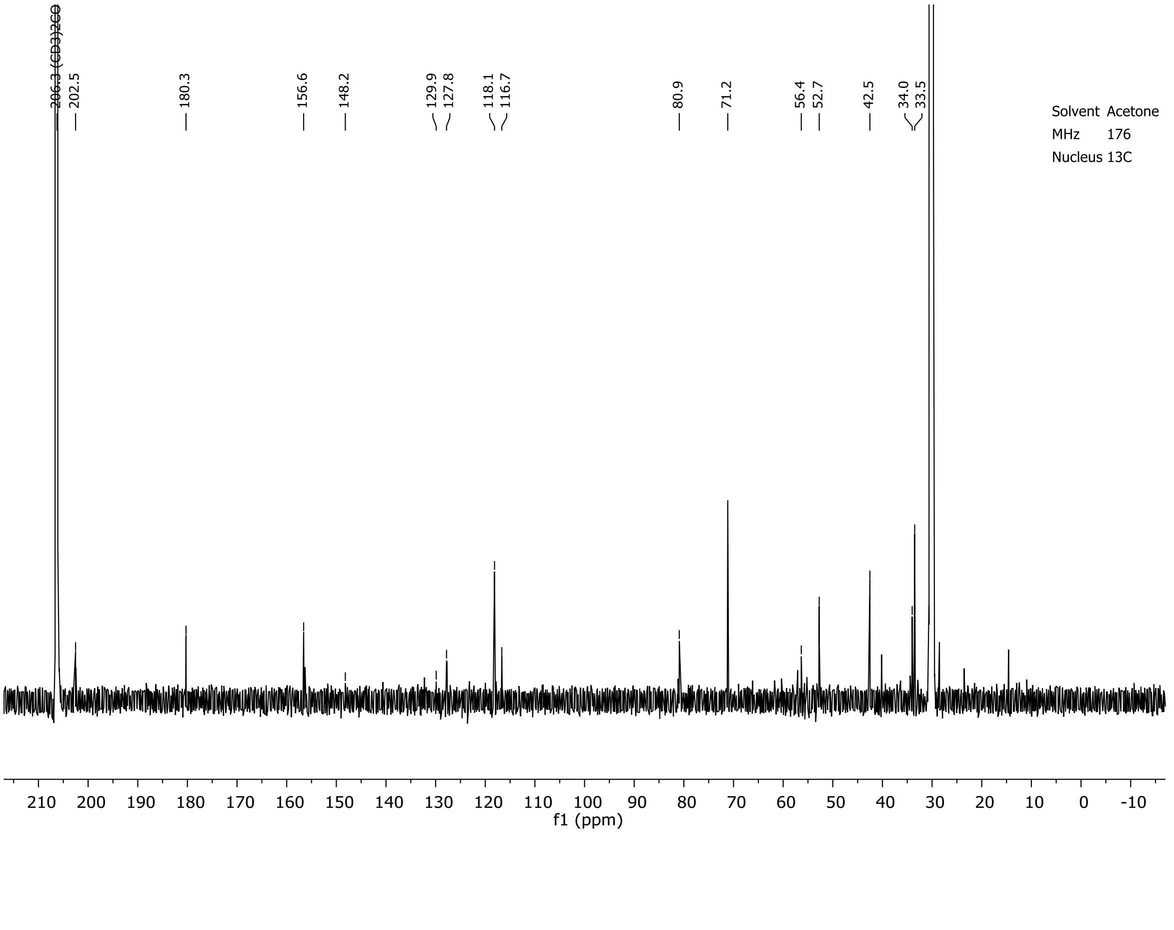
206.3 (CD3)2CO
202.5
180.3
156.6
148.2
129.9
127.8
118.1
116.7
80.9
71.2
56.4
52.7
42.5
34.0
33.5
Solvent Acetone
MHz 176
Nucleus 13C
210 200 190 180 170 160 150 140 130 120 110 100 90 80 70 60 50 40 30 20 10 0 -10
f1 (ppm)

4. X-Ray Data

4.1. Ferrocene ester S15

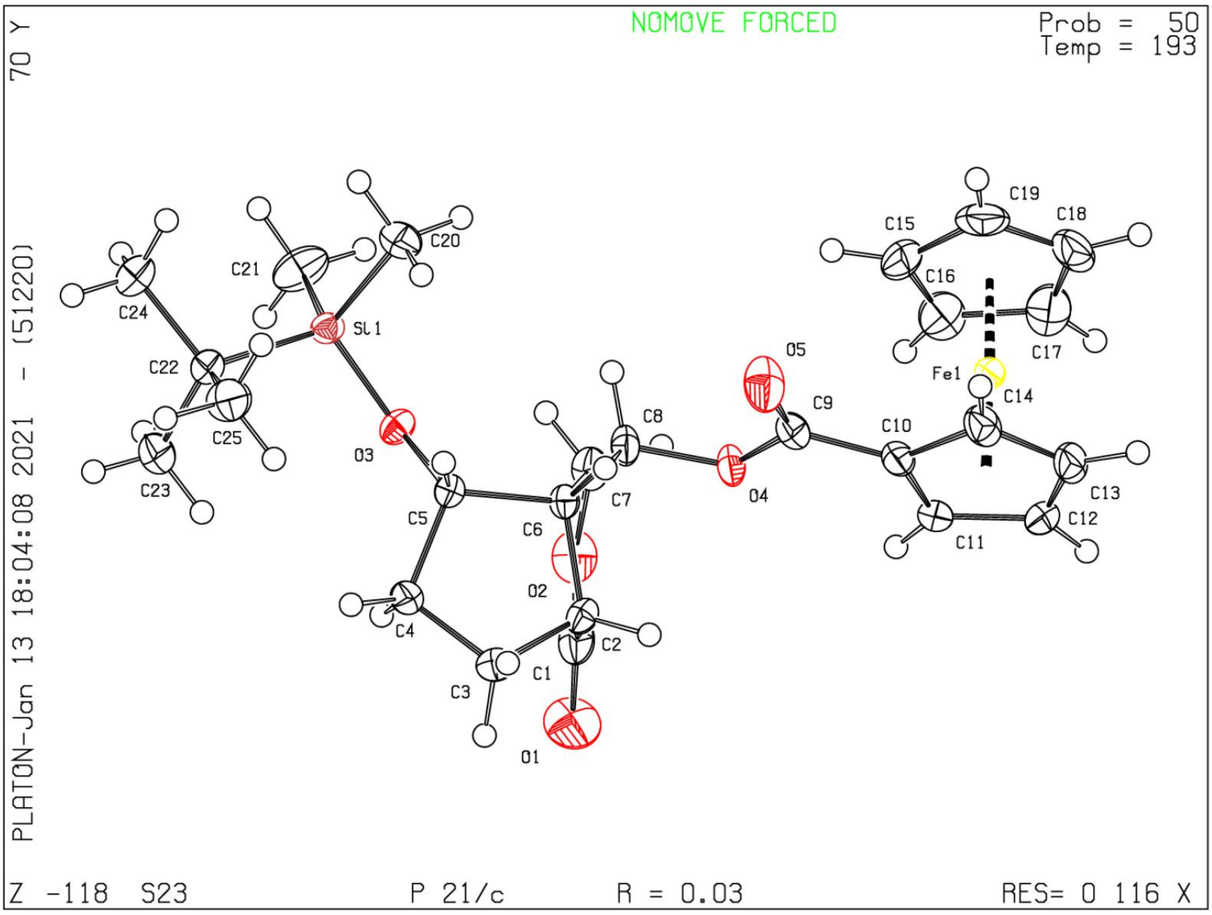

Table 15: Thermal ellipsoid plot (50% ellipsoid contour percent probability) of ferrocene ester **S15**.

Empirical formula	$C_{25}H_{34}FeO_5Si$	
Formula weight	262.29	
Temperature	193(2) K	
Wavelength	0.71073 Å	
Crystal system	Monoclinic	
Space group	$P2_1/c$ (no. 14)	
Unit cell dimensions	a = 6.3861(4) Å	α = 90°
	b = 12.7167(8) Å	β = 95.450(2)°
	c = 29.8799(18) Å	γ = 90°
Volume	2415.6(3) $Å^3$	
Z	4	
Density (calculated)	1.371 mg/m^3	
Absorption coefficient	0.707 mm^{-1}	
F(000)	1056	
Crystal size	0.160 x 0.110 x 0.060 mm^3	
Theta range for data collection	2.107 to 25.999°	
Index ranges	-7<=h<=7, -15<=k<=15, -36<=l<=36	
Reflections collected	30466	
Independent reflections	4752 [R(int) = 0.0260]	

Completeness to theta = 25.242°	100.0%
Absorption correction	Semi-empirical from equivalents
Max. and min. transmission	0.942 and 0.896
Refinement method	Full-matrix least-squares on F2
Data / restraints / parameters	4752 / 0 / 289
Goodness-of-fit on F^2	1.075
Final R indices [I>2sigma(I)]	R1 = 0.0280, wR2 = 0.0682
R indices (all data)	R1 = 0.0321, wR2 = 0.0700
Extinction coefficient	n/a
Largest diff. peak and hole	0.267 and -0.360 e.$Å^{-3}$
